# Neutralizing the Impact of the Virulence Factor LecA from *Pseudomonas aeruginosa* on Human Cells with New Glycomimetic Inhibitors

**DOI:** 10.1002/anie.202215535

**Published:** 2023-01-10

**Authors:** Eva Zahorska, Francesca Rosato, Kai Stober, Sakonwan Kuhaudomlarp, Joscha Meiers, Dirk Hauck, Dorina Reith, Emilie Gillon, Katharina Rox, Anne Imberty, Winfried Römer, Alexander Titz

**Affiliations:** ^1^ Chemical Biology of Carbohydrates (CBCH) Helmholtz Institute for Pharmaceutical Research Saarland (HIPS) Helmholtz Centre for Infection Research 66123 Saarbrücken Germany; ^2^ Department of Chemistry Saarland University 66123 Saarbrücken Germany; ^3^ Deutsches Zentrum für Infektionsforschung (DZIF), Standort Hannover- Braunschweig Germany; ^4^ Faculty of Biology University of Freiburg 79104 Freiburg Germany; ^5^ Signalling Research Centres BIOSS and CIBSS University of Freiburg 79104 Freiburg Germany; ^6^ Université Grenoble Alpes CNRS, CERMAV 38000 Grenoble France; ^7^ Department of Biochemistry Faculty of Science Mahidol University Bangkok 10400 Thailand; ^8^ Center for Excellence in Protein and Enzyme Technology Faculty of Science Mahidol University Bangkok 10400 Thailand; ^9^ Department of Chemical Biology (CBIO) Helmholtz Centre for Infection Research (HZI) 38124 Braunschweig Germany; ^10^ Freiburg Institute for Advanced Studies (FRIAS) University of Freiburg 79104 Freiburg Germany

**Keywords:** Glycomimetics, Lectin, Pathoblocker, Pseudomonas Aeruginosa, Virulence

## Abstract

Bacterial adhesion, biofilm formation and host cell invasion of the ESKAPE pathogen *Pseudomonas aeruginosa* require the tetravalent lectins LecA and LecB, which are therefore drug targets to fight these infections. Recently, we have reported highly potent divalent galactosides as specific LecA inhibitors. However, they suffered from very low solubility and an intrinsic chemical instability due to two acylhydrazone motifs, which precluded further biological evaluation. Here, we isosterically substituted the acylhydrazones and systematically varied linker identity and length between the two galactosides necessary for LecA binding. The optimized divalent LecA ligands showed improved stability and were up to 1000‐fold more soluble. Importantly, these properties now enabled their biological characterization. The lead compound **L2** potently inhibited LecA binding to lung epithelial cells, restored wound closure in a scratch assay and reduced the invasiveness of *P. aeruginosa* into host cells.

## Graphical abstract

Inhibition of the bacterial lectin LecA is an alternative option to treat infections with the ESKAPE pathogen Pseudomonas aeruginosa. Optimization of divalent LecA ligands yielded ligands with good drug‐like properties and high potency. Compound evaluation in functional cellular assays showed their ability to inhibit LecA‐mediated virulence, demonstrating their suitability as pathoblockers.

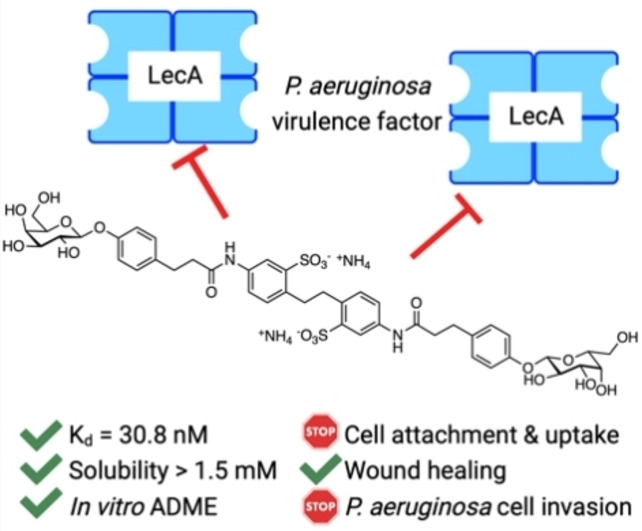

## Introduction

Carbohydrate‐protein interactions are essential recognition codes in many biological processes, including bacterial and viral infections. Therefore, lectins of pathogenic origin involved in host‐cell recognition, adhesion and/or biofilm formation are being recognized as new therapeutic targets.[^1^, ^2^] *Pseudomonas aeruginosa* is a Gram‐negative opportunistic bacterium that belongs to the group of highly drug resistant ESKAPE pathogens.^[3]^ According to the World Health Organization (WHO), the pathogen ranks as critical priority 1 due to its high level of resistance to antibiotics and the lack of new treatment options to combat it. Consequently, several new therapeutic strategies aim at an anti‐virulence therapy which circumvents the selection pressure of antibiotic treatment, thus preventing the emergence of new resistances.^[4]^ Antimicrobial resistance of *P. aeruginosa* is further enhanced by the bacterium′s ability to grow in biofilms—the causative mechanism for chronic infections.^[5]^ To this end, *P. aeruginosa* adhesion, biofilm formation and virulence depend on the tetravalent lectins LecA and LecB, both of which are encoded in its conserved core genome and are functionally conserved across clinical isolates.[^6^, ^7^, ^8^, ^9^] Furthermore, it was shown that LecA acts as a lipid zipper upon binding to its cellular receptor,[^10^, ^11^] the glycosphingolipid globotriaosylceramide (Gb3), and induces the formation of a plasma membrane nanodomain,[^12^, ^13^] cellular signaling^[14]^ and ultimately leads to bacterial invasion.^[10]^ Thus, the inhibition of LecA and LecB is a promising path forward to counteract *P. aeruginosa*′s pathogenicity and break its antimicrobial resistance.^[4]^ Encouragingly, it was also demonstrated that the inhalation of aerosols containing d‐galactose and l‐fucose, the monosaccharide ligands of LecA and LecB, respectively, reduced the bacterial burden and improved the clinical situation in cystic fibrosis patients.^[15]^

Consequently, numerous glycomimetics have been developed based on d‐galactose (*K*_d_=88 μM)^[16]^ for LecA and l‐fucose/d‐mannose (*K*_d_=430 nM for methyl α‐l‐fucoside, *K*_d_=71 μM methyl α‐d‐mannoside)^[17]^ for LecB.[^2^, ^4^, ^18^] Recently, we reported small molecules based on C‐glycosides targeting LecB which showed potent inhibition of LecB in the low micromolar range, prevented biofilm formation and exhibited beneficial pharmacokinetic properties.[^19^, ^20^, ^21^] For LecA, numerous monovalent glycomimetic inhibitors have been reported,[^22^, ^23^, ^24^] demonstrating the importance of an aromatic aglycon attached to the β‐galactoside to reach lower micromolar affinity. To alter the galactoside moiety itself, we have recently introduced the first covalent lectin inhibitor targeting a surface exposed cysteine residue in LecA.^[25]^ Furthermore, we also identified the first non‐carbohydrate lectin inhibitor for a bacterial lectin.^[26]^ Despite numerous attempts to increase potency, all monovalent LecA inhibitors reached at best only moderate potencies in the 5 to 50 μM range.

In contrast to LecB, the quaternary structure of LecA^[27]^ displays two adjacent binding sites that are optimally oriented in space for the simultaneous binding of two galactose moieties of a single divalent ligand, which can result in an increased binding affinity. Notably, Pieters and co‐workers developed low nanomolar LecA inhibitors with *K*_d_s down to 12 nM by connecting two galactosides through a linker containing several copies of rigid glucose‐triazole linkers.[^28^, ^29^] Despite the efficient click chemistry applied for the final assembly of the divalent ligands, in total 17 synthetic steps were required to prepare the azide and alkyne building blocks and final assembly. Replacing one of the glucose‐bistriazolyl spacers with a cyclohexyl bisthiourea moiety in a 9‐step synthesis yielded one compound with 30 nM affinity.^[30]^ Similarly, more complex divalent LecA ligands with peptide‐based linkers were reported by Novoa et al. (*K*_d_=82 nM) and Huang et al. (*K*_d_=71 nM).[^31^, ^32^]

Inspired by Pieters′ approach, we recently reported a series of highly active divalent LecA inhibitors with acylhydrazone based linkers (*K*_d_=11–81 nM) that simplified the synthesis to only four chemical steps.^[33]^ While these molecules showed the highest potency among reported divalent LecA inhibitors, they suffer from their intrinsic hydrolytic lability of the acylhydrazone bond and a very low aqueous solubility, despite the presence of the two hydrophilic carbohydrates. Furthermore, acylhydrazones undergo hydrolysis already at weak acidic pH resulting in toxic aldehyde and hydrazide degradation products that may also be formed in vivo.

Here, we report the optimization of these divalent molecules by replacing the acylhydrazone motif and varying the linker to balance potency, solubility and stability. The lead compound was then successfully analyzed in in vitro assays and showed excellent properties to block LecA‐mediated host cell binding, restore wound healing and decrease bacterial invasion into human cells.

## Results and Discussion

**Figure 1:**
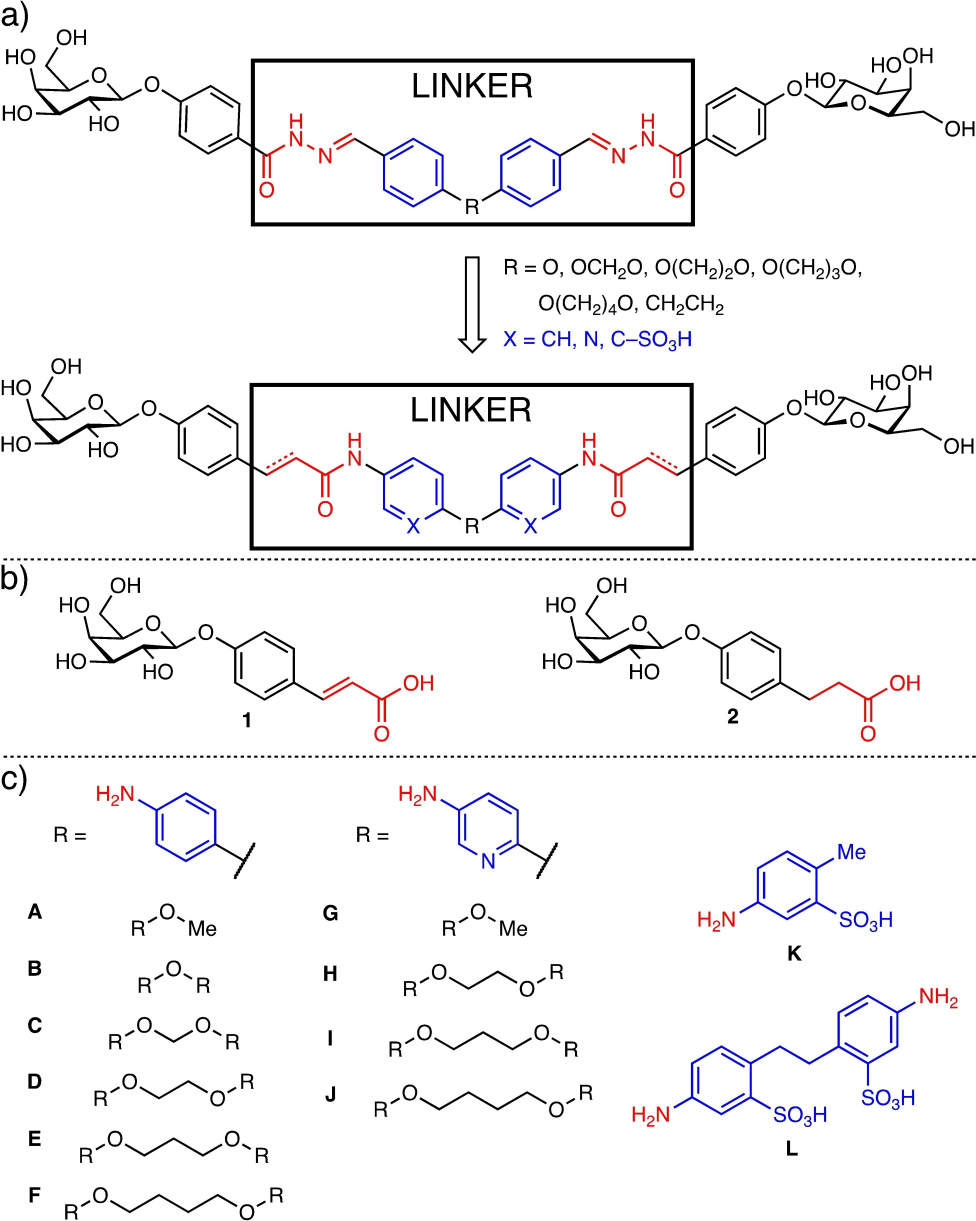
Divalent precision LecA ligands: a) parent bisacylhydrazone LecA inhibitors^[33]^ (top) and new generation optimized bioisosters (bottom). Proposed chemical modifications are highlighted: amide linkage as acylhydrazone bioisoster in red and linker derivatizations in blue. b) Galactoside building blocks with terminal α,β‐unsaturated carboxylate **1** and its saturated analogue **2**. c) Linker moieties: anilines **B**–**F**, aminopyridines **H**–**J** and sulfonated linker **L**, and their monovalent controls **A**, **G**, and **K**.

We substituted the acylhydrazone motif present in our previous compounds with a more stable amide bond and varied linker identity and length in order to increase solubility and stability (Figure 1a). Two galactoside building blocks bearing coumaric acid (**1**, Figure 1b) or hydroxyphenyl propionic acid (**2**) as aglycons were selected to investigate the effect of the rigid olefin in **1**, comparable to the parent acylhydrazones, versus the flexible alkyl motif in **2**. These galactosylated carboxylic acids were then coupled to various bisanilines to yield the desired divalent LecA inhibitors. Since an optimal length and geometry of the divalent ligand is important to simultaneously bind to the two neighboring LecA sites, linker length was varied by stepwise introduction of methylene units. The aromatic moieties of the linker and its length were also varied and bisaniline linkers **B**–**F** and their monovalent control **A** (Figure 1c) were used to mimic the previously used bisbenzaldehyde structures.^[33]^ Furthermore, our rational solubility optimization included the introduction of hydrogen‐bonding polar groups or ionizable moieties into the divalent ligands using bisaminopyridine linkers **H**–**J** or sulfonated linker **L** and their monovalent controls **G** and **K**, respectively.

**Scheme 1:**
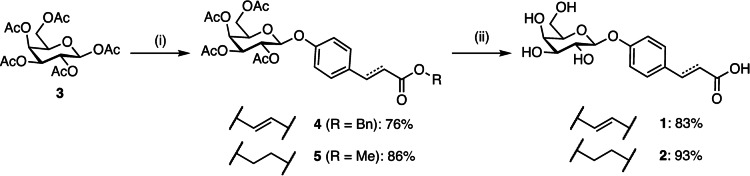
Synthesis of galactoside building blocks **1** and **2**. Reagents and conditions: (i) benzyl *p*‐coumarate/methyl 3‐(4‐hydroxyphenyl)‐propanoate, BF_3_⋅Et_2_O, CHCl_3_ for **4** and CH_2_Cl_2_ for **5**, 0 °C–r.t., overnight; (ii) NaOH, H_2_O/MeOH (1 : 1), 50 °C for **1** and r.t. for **2**, 1–2 h.

Synthesis of the two galactoside building blocks **1** and **2** started with β‐selective glycosylation of benzyl coumarate or methyl 3‐(4‐hydroxyphenyl)propanoate with β‐d‐galactose pentaacetate (**3**) under Lewis acid catalysis (Scheme 1). β‐Glycosides **4** and **5** were obtained in 76 % and 86 % yields, respectively. Saponification of the esters with aqueous NaOH resulted in galactosides **1** and **2** in good yields. Synthesis of coumarate **1** was initially attempted using the methyl ester under identical glycosylation conditions as for compound **2**, but this transformation was unsuccessful most probably due to its poor solubility in dichloromethane and only poor yields were achieved using more polar chloroform as a solvent instead. Changing from methyl to benzyl coumarate improved solubility in those solvents and the glycosylation gave compound **4** in good yield (76 %).

**Scheme 2:**
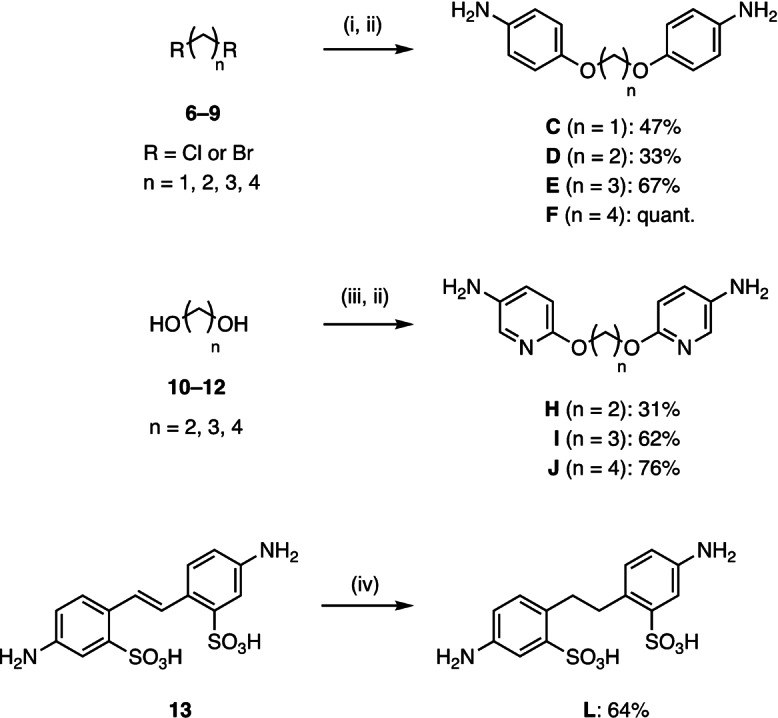
Synthesis of benzene, pyridine and phenylsulfonate linkers. Reagents and conditions: (i) 4‐nitrophenol, K_2_CO_3_, DMF, 70 °C, microwave, 11 h–4 d (for **C** 10 d, no irradiation); (ii) H_2_, Pd/C, CH_2_Cl_2_/MeOH, r.t., 3 h–o.n.; (iii) 2‐chloro‐5‐nitropyridine, NaH, r.t., DMF, 1 h–2 d (for **H** K_2_CO_3_, 65 °C, DMF, 5 d); (iv) Raney Ni, H_2_, r.t., H_2_O, 6 d.

The linkers were synthesized or purchased: while anilines **A** and **B**, aminopyridine **G** and sulfonated linker **K** were commercially available, linkers **C**–**F** and bis‐aminopyridine linkers **H**–**J** were prepared in two steps: a double nucleophilic substitution of the α,ω‐alkyldihalides (**6**–**9**) with 4‐nitrophenol or two nucleophilic aromatic substitutions using α,ω‐alkyldiols (**10**–**12**) with 2‐chloro‐5‐nitro‐pyridine. In both cases, a palladium‐catalyzed hydrogenation followed to give the desired bis‐anilines or bis‐aminopyridines, respectively (Scheme 2). Ethyl‐spaced bissulfonated linker **L** was obtained by reduction of 4,4′‐diaminostilbene‐2,2′‐disulfonic acid (**13**) with hydrogen on Raney nickel.

**Scheme 3:**
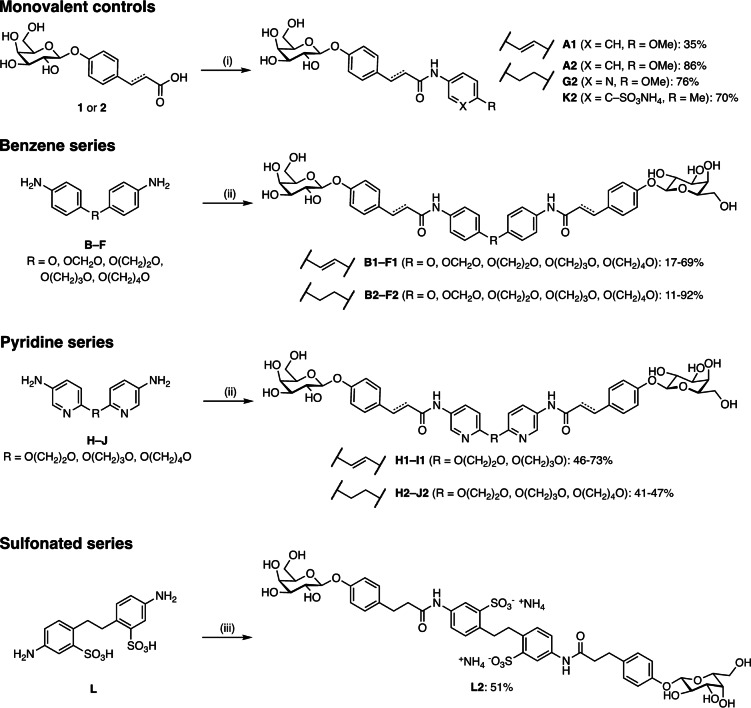
Synthesis of divalent LecA ligands and their monovalent analogues as controls. Reagents and conditions: (i) for **A** and **G**: HBTU, DIPEA, DMF, r.t., 1 h–overnight, for **K**: PyBOP, N‐methylmorpholine, DMF, r.t., overnight; (ii) galactoside **1** or **2**, HBTU, DIPEA, DMF, r.t., 2 h–2 d, (iii) galactoside **2**, PyBOP, N‐methylmorpholine, DMF, r.t., overnight.

Final assembly of the divalent LecA inhibitors was achieved by coupling of the amino‐substituted linkers **A**–**L** with carboxylate‐containing galactosides **1** or **2** using HBTU or PyBOP as peptide coupling reagents (Scheme 3). High turnovers were observed for all coupling reactions, but purification difficulties caused varying yields: the lower solubility was responsible for isolated yields in the benzene series (**A1**–**F1** and **A2**–**F2**), whereas side product formation was observed in the pyridine series (**H1**–**I1** and **G2**–**J2**). The sulfonated ligands **K2** and **L2** were obtained as ammonium salts since their chromatography required buffered eluents to avoid acid‐catalyzed self‐hydrolysis of the glyosidic linkage upon solvent removal and concentration.

Next, we quantified the solubility of at least one representative of each new class and of one parent bisacylhydrazone in aqueous media (Table 1). All tested new derivatives showed improved solubility compared to the previous bisacylhydrazone **14**. The very low kinetic solubility of bisacylhydrazone **14** (*S*=1.6±0.4 μM) was increased twofold in its amide analogues **D1** (*S*=3.3±1.3 μM) and **D2** (*S*=3.1±2.1 μM). Substitution of the benzene ring with a pyridine moiety further increased solubility twofold in case of α,β‐unsaturated amide analogue **H1** (*S*=8.1±2.2 μM) and almost fivefold in the saturated analogue **H2** (*S*=14.8±1.5 μM) compared to **D1** and **D2**, respectively. Surprisingly, another fourfold increase in solubility was observed for **I2** (*S*=63.3±4.2 μM) compared to **H2**, despite only a small structural difference of one additional methylene in the linker. The longest pyridine ligand **J2** showed again almost identical solubility to **H2**. Hence, the linker has a strong impact on solubility in the pyridine amide series, which could result from the conformational preferences of an ethylene glycol compared to alkyl chains with different numbers of methylene units that might affect ligand self‐aggregation and thus their solubility. However, excellent aqueous solubility was finally achieved with sulfonated divalent ligand **L2**, which was fully dissolved from its solid form in an aqueous buffer (*S*>1.5 mM).

**Table 1:**
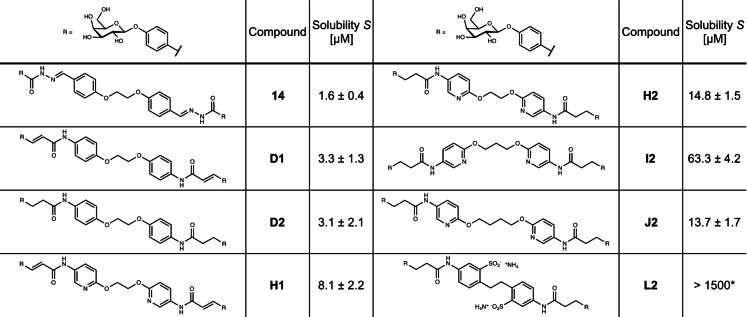
Aqueous solubility of selected LecA inhibitors. Kinetic solubility determined in TBS/Ca^2+^ buffer (pH 7.4) with 1 % DMSO (N≥3). *Thermodynamic solubility in TBS/Ca^2+^ buffer (w/o DMSO, N=1).

We then determined the in vitro metabolic stability in plasma and liver microsomes as well as plasma protein binding for a selected subset of the synthesized LecA inhibitors, i.e. **D1**, **D2**, **H1**, **H2** and **L2** and compared them to the parent bisacylhydrazone **14** (Table 2). Stability tests revealed a low intrinsic clearance (CL_int_) by mouse liver microsomes (MLM) for most tested compounds (CL_int_=6.8–23 μL min^−1^/mg protein), with a slightly elevated clearance for **D2** (CL_int_=29.6 μL min^−1^/mg protein) and sulfonated ligand **L2** (CL_int_=29.5 μL min^−1^/mg protein). The metabolic stability of the compounds differed in presence of human liver microsomes (HLM). Introduction of a pyridine ring decreased stability (**H1** CL_int_=28.6 μL min^−1^/mg protein, **H2** CL_int_=32.5 μL min^−1^/mg protein) compared to the benzene analogues (**D1** CL_int_=21.9 μL min^−1^/mg protein, **D2** CL_int_=21.0 μL min^−1^/mg protein) and to the parent acylhydrazone **14** (CL_int_=19.6 μL min^−1^/mg protein). Finally, sulfonated **L2** showed the highest stability in presence of HLM (CL_int_=9.2 μL min^−1^/mg protein).

**Table 2:**
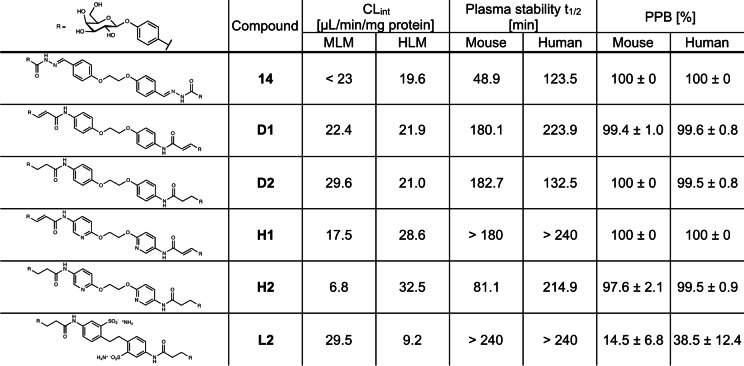
Plasma and metabolic stability in mouse and human liver microsomes (MLM, HLM) and plasma protein binding (PPB) of selected LecA inhibitors (N=3).

Then, we analyzed the stability of the compounds in plasma. Acylhydrazone **14** was degraded faster (*t*_1/2_=48.9 min) in mouse plasma whereas a higher stability was observed in human plasma (*t*_1/2_=123.5 min). The tested representatives of the new ligands exhibited high stability in both mouse and human plasma (*t*_1/2_≥180 min), with exception of the coumarates: pyridine **H2** was only moderately stable in mouse plasma (*t*_1/2_=81.1 min) and benzene **D2** showed a slow degradation in human plasma (*t*_1/2_=132.5 min). The observed increase in plasma stability of the tested amide derivatives compared to bisacylhydrazone **14** supports our design hypothesis for the isosteric replacement of the bisacylhydrazone linking motif. In addition, all tested compounds showed very high mouse and human plasma protein binding (PPB) with lowest PPB of 97 % for saturated pyridyl amide **H2**. In contrast, sulfonated **L2** showed a remarkably low PPB in both species (mouse 14 %, human 38 %).

All synthesized galactosides were then evaluated for LecA inhibition using a competitive binding assay based on fluorescence polarization (Figure S1).^[24]^ Monovalent galactosides **A1**, **A2**, **G2** and **K2** showed similar IC_50_ values between 14–19 μM. The monovalent acrylamide **A1** (IC_50_=18.8±6.6 μM) was equipotent to its saturated and more flexible propanamide analogue **A2** (IC_50_=18.9±5.5 μM). Replacement of the benzene ring with a pyridine in **G2** (IC_50_=14.3±7.2 μM) or addition of the sulfonate in **K2** (IC_50_=14.4±3.6 μM) had only minor effects on LecA inhibition. In contrast to the monovalent controls and similar to our previous observations for the bisacylhydrazones, titrations with all divalent LecA ligands exhibited very steep Hill slopes with fitted IC_50_ values between 3.2–8.0 μM. These data are indicative for the high potency of divalent compounds and suggest that the lower assay limit was reached.^[33]^

**Figure 2:**
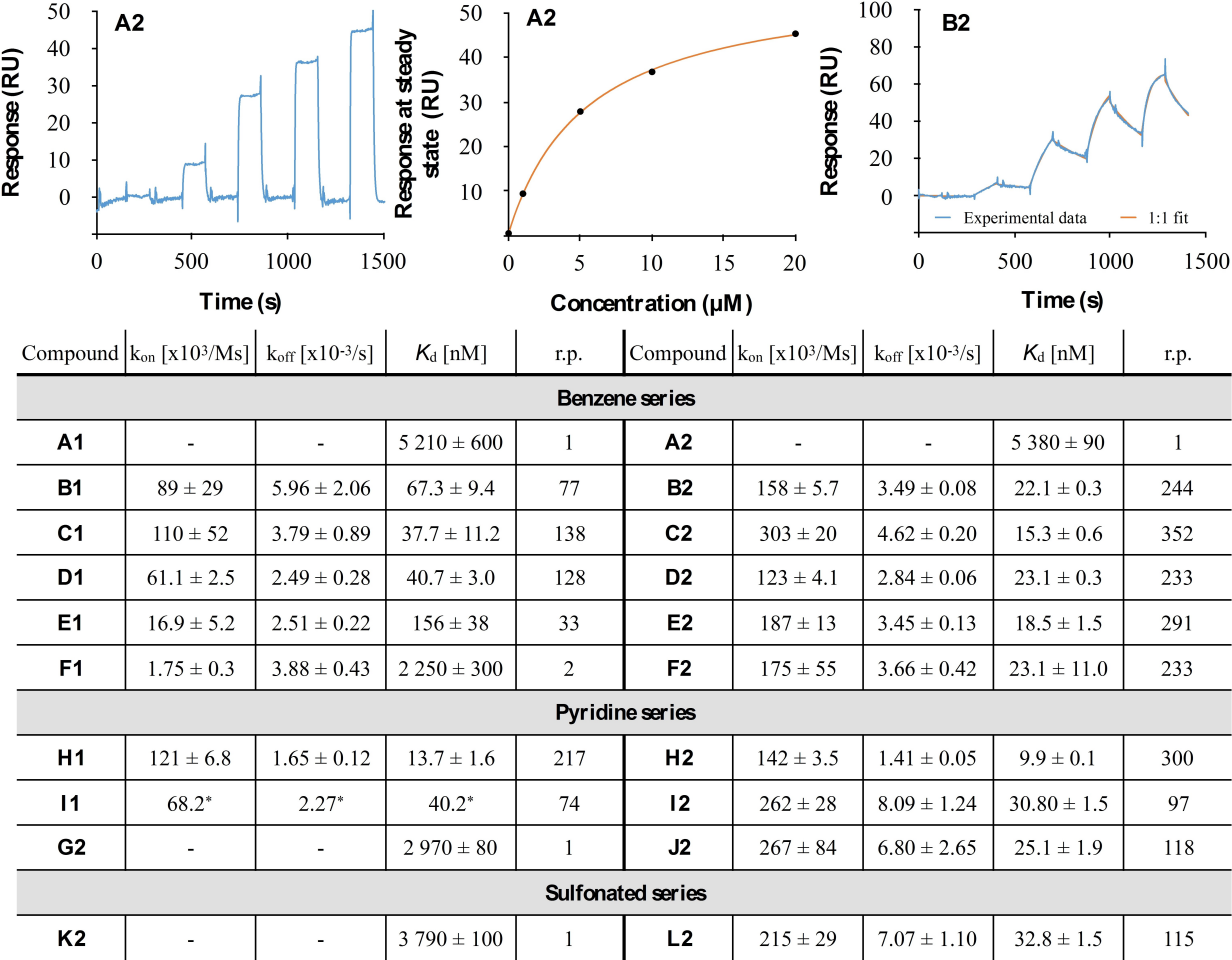
Direct binding of LecA ligands determined by SPR (N=3). Sensorgram of monovalent **A2** (left) with affinity analysis (center) and sensorgram of divalent **B2** (right) from single‐cycle kinetics experiments (injections of 0, 10, 50, 100, 200 nM for **B2** or 0, 1, 2.5, 5, 10 μM for **A2**) are shown. Relative potencies (r.p.) were calculated compared to respective monovalent compound in each series and are valency‐normalized. *N=1 due to sample aggregation.

To overcome the assay limit for these highly potent inhibitors, direct binding to immobilized LecA was quantified using surface plasmon resonance (SPR, Figure 2, Figures S2–S4). In agreement with the competitive binding assay, the binding affinity determined by SPR for the monovalent acrylamide ligand **A1** (*K*_d_=5.21±0.60 μM) and propanamide ligand **A2** (*K*_d_=5.38±0.09 μM) were comparable to their IC_50_ values. Interestingly, a striking difference was observed among divalent inhibitors. Within the benzene series, the acrylamides **B1**–**F1** showed *K*_d_ values in the nanomolar to micromolar range while their propanamide analogues **B2**–**F2** were two‐ to threefold more active when attached to the shorter linkers **B**–**D** and 100‐ to 200‐fold more potent for the longest linkers **E** and **F**. With respect to linker length, in the acrylamide series linker **C** containing one methylene unit exhibited strongest binding affinity (**C1** *K*_d_=37.7±11 nM). Increasing to four methylene units in **F1** led to a complete loss of divalent binding affinity boost (*K*_d_=2.25±0.3 μM). In contrast, all divalent ligands carrying the propanamide motif (**B2**–**F2)** showed very high binding affinity to LecA between 15 and 23 nM. *K*_d_ values were oscillating with the number of methylene units present in the linker, presumably linked to the zig‐zag geometry of the hydrocarbon chain. These observations could be explained by an increased rigidity of the acrylamides, that does not allow the ligand to simultaneously bind to two adjacent binding sites when the linker is too long.

Monovalent ligands from the pyridine series (**G2**) and sulfonated **K2** showed slightly enhanced binding affinity to LecA (**G2** *K*_d_=2.97±0.08 μM, **K2** *K*_d_=3.79±0.10 μM) when compared to **A2**. Substitution of the benzene ring with a pyridine was also favored for divalent ligands: three‐ and four‐fold increase in binding affinity was observed for acrylamide‐based ligand **H1** (*K*_d_=13.7±1.6 nM) and **I1** (*K*_d_=40.2 nM) when compared to their benzene analogues **D1** and **E1**, respectively. Among the propanamide derivatives, the increase in binding affinity was less pronounced: **H2** (*K*_d_=9.9±0.1 nM) and **I2** (*K*_d_=30.8±1.5 nM) were twice more active, while the longest pyridine ligand **J2** (*K*_d_=25.1±1.9 nM) was equipotent to its benzene analog **F2**. The observed affinity increase could result from additional interactions of the pyridine rings with the protein surface. Furthermore, divalent sulfonate **L2** (*K*_d_=32.8±1.5 nM) also reached low nanomolar affinity for LecA, indicating that an ether function in the linkers is not essential and that the negatively charged sulfonate solubility tags were tolerated. In general, divalent ligands containing propanamides (**B2**–**F2**, **H2**–**J2** and **L2**) showed faster association kinetics (*k*_on_) compared to their acrylamide analogues (**B1**–**F1**, **H1**–**I1**), indicating the importance of a certain degree of flexibility to allow simultaneous binding to two LecA binding sites.

**Figure 3:**
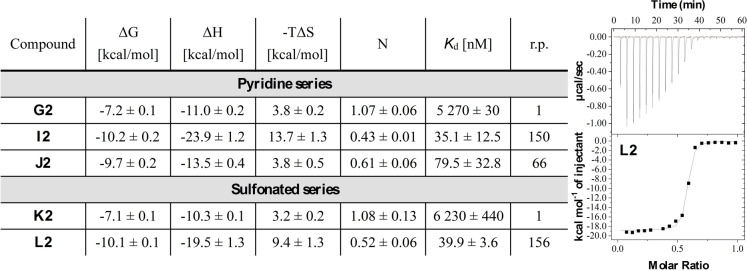
Direct binding of selected ligands to LecA by ITC (N=3). Titration of LecA (50 μM) with divalent sulfonated ligand **L2** (250 μM) is depicted. Relative potencies (r.p.) were calculated compared to respective monovalent compound in each series and are valency‐normalized.

Due to the high solubility of the propanamides in the pyridine series and the sulfonated ligand **L2**, we determined binding thermodynamics with LecA using isothermal titration microcalorimetry (ITC, Figure 3, Figures S5–S9). *K*_d_ values for monovalent **G2** and **K2** by ITC were in the low micromolar range (**G2**: *K*_d_=5.27±0.03 μM, **K2**: *K*_d_=6.23±0.44 μM), nearly twofold higher than those obtained by SPR. Divalent pyridine **I2** (*K*_d_=35.1±12.5 nM) and divalent sulfonated ligand **L2** (*K*_d_=39.9±3.6 nM) exhibited binding affinities in the low nanomolar range consistent with SPR. In both cases, the enthalpy of binding increased approximately twofold (**I2**: Δ*H*=−23.9±1.2 kcal mol^−1^, **L2**: Δ*H*=−19.5±1.3 kcal mol^−1^) compared to their monovalent analogues (**G2**: Δ*H*=−11.0±0.2 kcal mol^−1^, **K2**: Δ*H*=−10.3±0.1 kcal mol^−1^), while the binding entropy was increased approximately threefold (**G2**: −*T*Δ*S*=3.8±0.2 kcal mol^−1^ vs. **I2**: −*T*Δ*S*=13.7±1.3 kcal mol^−1^, **K2**: −*T*Δ*S*=3.2±0.2 kcal mol^−1^ vs. **L2**: −*T*Δ*S*=9.4±1.3 kcal mol^−1^). The divalent ligand with the longest linker, **J2**, was less potent, yet remaining in the nanomolar range (*K*_d_=79.5±32.8 nM). However, this compound showed a decreased binding enthalpy (Δ*H*=−13.5±0.4 kcal mol^−1^) but also lower entropy costs (−*T*Δ*S*=3.8±0.5 kcal mol^−1^), suggesting an alternative binding mode for **J2**.

Next, we investigated the two most promising compounds **H2** and **L2** for their efficacy to inhibit LecA binding to H1299 human lung epithelial cells using flow cytometry and confocal microscopy. To this end, LecA was fluorescently labelled with Alexa Fluor 488 NHS ester (LecA‐AF488), incubated with varying concentrations of the inhibitors (100 nM to 10 μM) and subsequently added to the cells.

**Figure 4:**
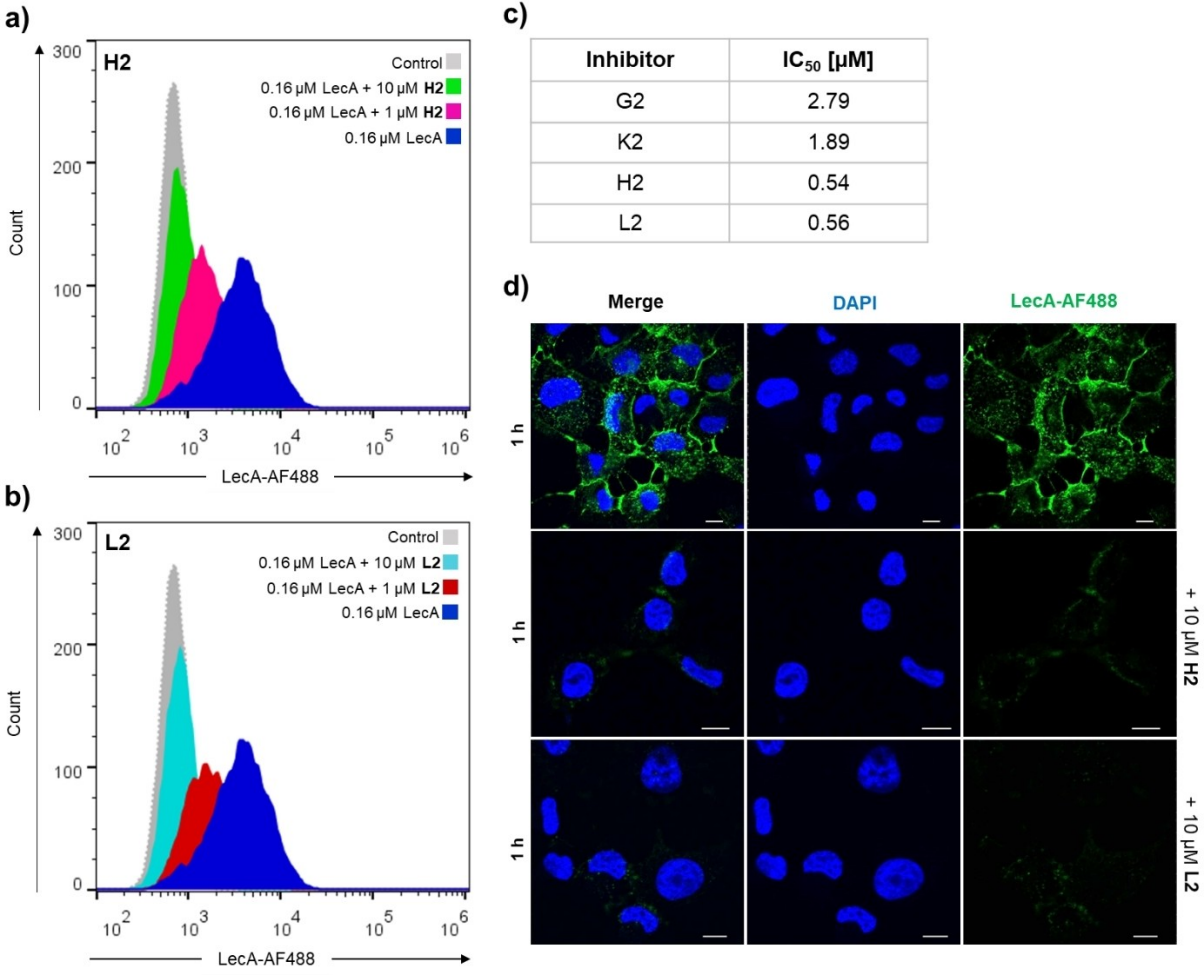
Divalent inhibitors **H2** and **L2** decreased LecA binding and uptake to lung cells (N=3). Histograms of fluorescence intensity of gated live H1299 cells incubated with 0.16 μM of LecA‐AF488 in presence of a) **H2** or b) **L2**. H1299 cells (without LecA, **H2** or **L2**) served as a negative control (grey). c) IC_50_ values for inhibition of LecA binding to H1299 cells of monovalent inhibitors **G2** and **K2**, divalent inhibitors **H2** and **L2** following titrations in flow cytometry assays. d) Confocal imaging of H1299 cells incubated with 0.5 μM LecA‐AF488 (in green) or 0.5 μM LecA‐AF488 which was preincubated with 10 μM **H2** or **L2**. Nuclei were counterstained with DAPI (blue). Scale bars=10 μm.

For flow cytometry analysis, inhibitors were preincubated with 0.16 μM LecA prior addition to the H1299 cells. After incubation for 30 min, fluorescence intensity was recorded at the flow cytometer. The sulfonated divalent inhibitor **L2** decreased LecA binding to H1299 cells by more than 30 % at 250 nM, whereas divalent pyridine **H2** only showed 16 % inhibition (Figure 4a, b). On the other hand, **H2** was slightly more potent at the concentration range from 0.5 to 2.5 μM, consistent with its higher affinity measured by SPR. At 1 μM, **H2** and **L2** decreased LecA binding to the host cells by 50 % and at 7.5 μM and above, a complete inhibition of lectin binding to the cells was achieved. In contrast, 10 μM of the corresponding monovalent ligands **G2** and **K2** were required for 50 % inhibition (Figure S10). Thus, both divalent inhibitors, **H2** and **L2**, showed high efficacy to prevent LecA binding to host cells and revealed a significant increase in potency compared to the monovalent inhibitors **G2** and **K2**. When mean fluorescence intensity (MFI) of samples treated with LecA‐AF488 and divalent inhibitors were normalized to the negative control and plotted as a function of inhibitor concentration (Figure S11), **H2** and **L2** demonstrated a dose‐dependent inhibition of lectin binding to the host cells and determined IC_50_ values were 0.54 μM for **H2** and 0.56 μM for **L2** (Figure 4c). On the other hand, the IC_50_ values of the monovalent ligands were in the micromolar range (**G2** IC_50_=2.8 μM, **K2** IC_50_=1.9 μM), thus further confirming the benefit of divalent ligands.

The ability of **H2** and **L2** to diminish LecA binding to host receptors at the plasma membrane and its subsequent internalization into H1299 cells was further analyzed by confocal fluorescence microscopy. Here, the applied LecA concentration was increased to improve signal to noise and consequently image quality and inhibitors were preincubated with 0.5 μM LecA before addition to the cells. After 60 min at 37 °C, the cells were imaged: in inhibitor‐treated cells, a remarkably low fluorescence signal for LecA was observed at 10 μM divalent ligands compared to those without inhibitor (Figure 4d). Sulfonated inhibitor **L2** showed a stronger inhibition of lectin binding than **H2** from the pyridine series. A complete inhibition of LecA binding and cellular uptake in the presence of divalent ligands was achieved at 7.5 μM, corresponding to a 10‐fold potency increase compared to the monovalent inhibitors **G2** and **K2**, which required 75 μM to block LecA binding (Figure S11). Confocal microscopy images thus confirmed that the divalent inhibitors can prevent LecA binding to the cell surface and reduce lectin uptake in a concentration‐dependent manner. Overall, these data suggest that the divalent inhibitors **H2** and **L2** have a high potential to block LecA and prevent recognition of the glycosphingolipid Gb3 on host cells, possibly impairing the invasiveness of *P. aeruginosa*.

**Figure 5:**
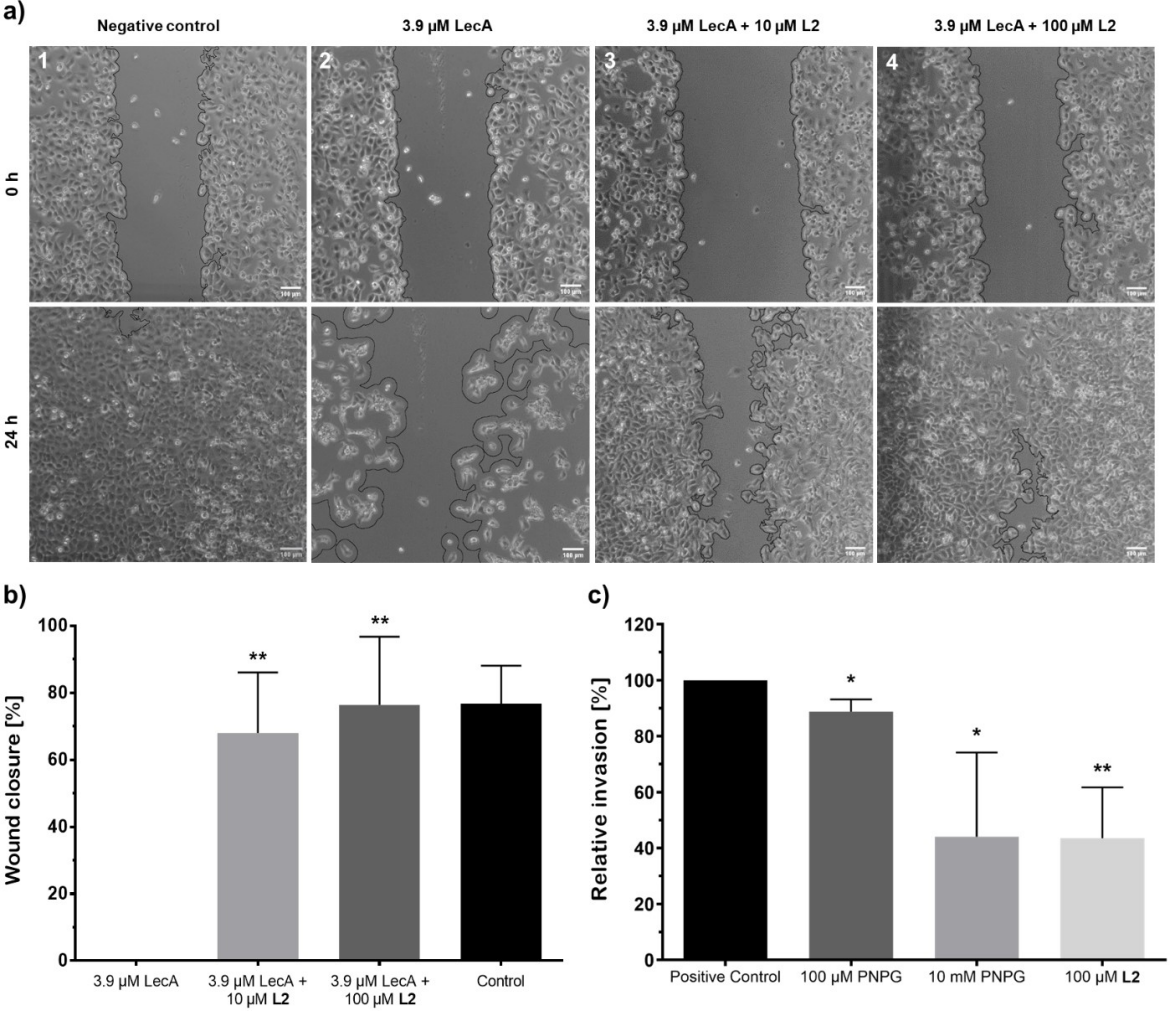
Divalent inhibitor **L2** restored wound healing in LecA‐treated cells and reduced *P. aeruginosa* PAO1 invasiveness into host cells. Wound healing (N=3): a) Light microscopy images of scratched H1299 cells at 0 h and 24 h post‐treatment with (1) PBS, (2) 3.9 μM LecA, (3) 3.9 μM LecA preincubated with 10 μM **L2**, or (4) 3.9 μM LecA preincubated with 100 μM **L2**. Scale bars=100 μm. b) Quantification of wound closure after 24 h, control in absence of LecA. Bacterial Invasion (N≥4): c) Incubation of *P. aeruginosa* PAO1 with 100 μM PNPG, 10 mM PNPG or 100 μM **L2** for 30 min reduced bacterial invasiveness into H1299 cells in comparison to the absence of inhibitors (positive control). For each experiment, conditions were normalized to invasion of untreated bacteria (positive control).

The opportunistic bacterium *P. aeruginosa* depends on favorable circumstances to infect the host, such as a suppressed immunity or wounded skin.[^34^, ^35^] Chronic infection of wounds with *P. aeruginosa* increases the risk of nosocomial spread, are difficult to treat and after systemic dissemination can result in a fatal outcome in patients. In a previous study, we investigated the effect of the fucose‐binding *P. aeruginosa* lectin LecB on epithelial wound healing.^[36]^ Cell migration was found to be strongly inhibited in the presence of LecB in a dose‐dependent manner, and could be fully blocked with 50 μg mL^−1^ (4.3 μM) LecB. Here, we investigated whether LecA also impairs the same physiological process. To this end, H1299 cell monolayers were grown, and after scratching with a pipette tip, wound closure was monitored microscopically (Figure 5a). Cells incubated with 3.9 μM LecA showed a visible decrease in cell migration, compared to full closure of the wound after 24 h in untreated cells. Furthermore, cell detachment in the close proximity of the wound was observed, suggesting that the lectin LecA can significantly hinder cell migration, adhesion and wound closure. The mode of action of LecA in the wound healing process remains elusive. However, in the presence of the monovalent inhibitor **K2** (100 μM), wound healing was partially restored to about 50 % after 24 h compared to samples treated solely with LecA (Figure S13). Importantly, when LecA was added to the cells together with **L2** (10 μM and 100 μM, respectively), the wound healed at a similar rate to the control in absence of LecA. Remarkably, in presence of 10 μM **L2**, cell migration was restored to about 70 % compared to the negative control, and the wound closed by 80 % after 24 h of incubation with 100 μM **L2** (Figure 5b). In conclusion, the detrimental effect of LecA in the wound‐scratch assay could be reversed by addition of our inhibitors and wound healing was restored to a physiological closure rate. Thus, these data demonstrate that LecA binds to host cells to impair cell migration and, furthermore, unravel the potential of these inhibitors in restoring such a process, an important step towards the treatment and eradication of chronic infections.

*P. aeruginosa* is able to invade non‐phagocytic cells via a lipid zipper mechanism.^[10]^ Binding of LecA to the glycosphingolipid Gb3 followed by LecA‐induced receptor clustering plays a crucial role in this process. The LecA ligand 4‐nitrophenyl α‐ d‐galactopyranoside (PNPG) has been previously shown to interfere with these events at a high concentration of 10 mM leading to a reduction of bacterial invasiveness to 30–60 % as compared to untreated bacteria (100 % invasiveness).[^10^, ^31^] For invasion assays, H1299 cells were incubated with *P. aeruginosa* in presence and absence of LecA inhibitors, followed by killing of extracellular bacteria with the antibiotic amikacin. Then, all intracellular bacteria were released by lysis of the human cells and viable bacteria were quantified by plating. To assess the impact of LecA inhibitors on the invasiveness, the influence of the divalent LecA inhibitor **L2** on bacterial invasion into host cells was evaluated and compared to PNPG. Compared to untreated bacteria (100 % invasion), invasiveness was slightly reduced to 89 % in presence of 100 μM PNPG and to 44 % with 10 mM PNPG, which is comparable to literature data.[^10^, ^31^] In contrast, the divalent LecA ligand **L2** achieved an invasion decrease to 44 % already at 100 μM (Figure 5c). **L2** was thus able to reach a similar reduction of invasion at a 100‐fold lower concentration than the one needed for PNPG.

Additionally, the cytotoxicity of sulfonated ligands **K2** and **L2** on H1299 cells was evaluated prior to the invasion assay (Figure S12). **K2** and **L2** did not exhibit significant toxicity during 24 h in ranges from 10–100 μM and cellular viability was preserved. However, >50 % inhibition of cell proliferation was observed for higher inhibitor concentrations (500 and 750 μM), indicating a potential toxicity window for the tested compounds.

**Figure 6:**
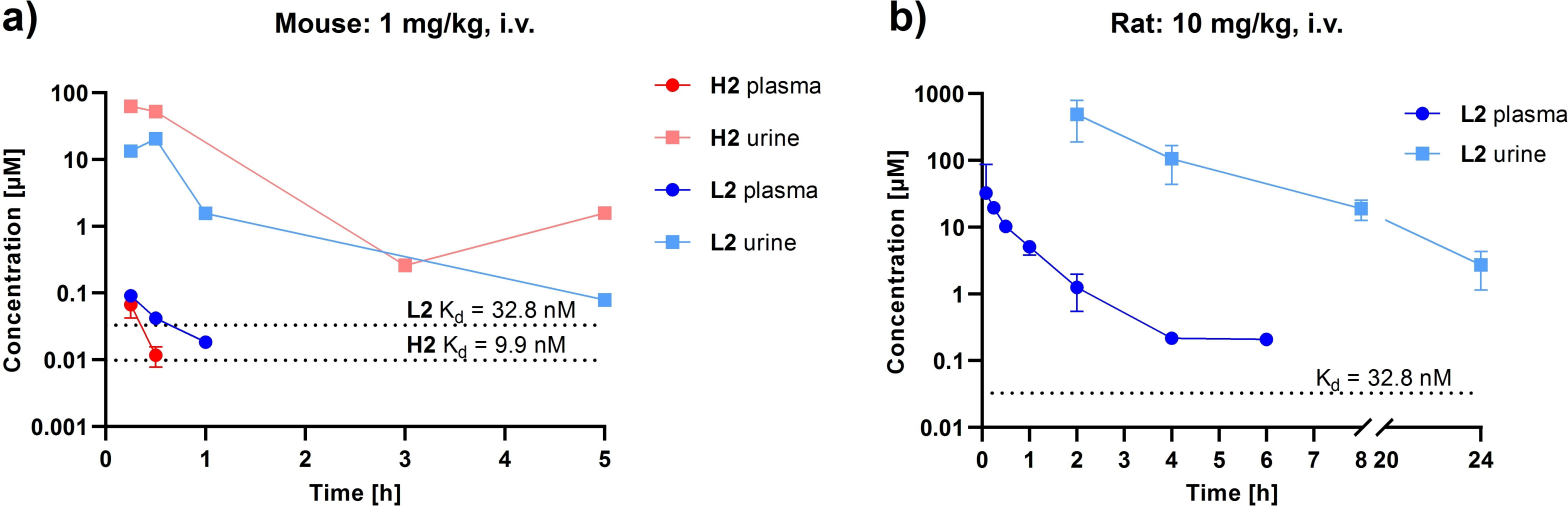
In vivo pharmacokinetics of **H2** and **L2**. Plasma and urine concentrations in a) CD‐1 mice (N=2 per compound) after 1 mg kg^−1^ i. v. dose of **H2** (1.15 μmol kg^−1^) and **L2** (0.97 μmol kg^−1^) and in b) Sprague‐Dawley rats (N=3) after 10 mg kg^−1^ i. v. dose of **L2** (9.73 μmol kg^−1^). Dashed lines represent the in vitro *K*_d_ values.

Encouraged by these promising in vitro results, an in vivo pharmacokinetic study was conducted for **H2** and **L2** in mice (1 mg kg^−1^, i. v.) and in rats for **L2** (10 mg kg^−1^, i. v.). Compound concentrations were monitored in plasma and urine (Figure 6). **H2** and **L2** were cleared from mouse plasma within 1 h. Both compounds rapidly reached high concentrations in urine, several orders of magnitude above their respective in vitro LecA binding affinity. In fact, concentrations above the *K*_d_s were detected over the entire observation period of 5 h. The overall lower urine exposure of **L2** compared to **H2** ($c_{max}^{urine}$ =63.0 μM and 13.4 μM for **H2** and **L2**, respectively) could result from first pass metabolism and a lower hepatic stability of **L2** in vitro (Table 2). Interestingly, **H2** showed higher renal elimination at 5 h than at 3 h, possibly a result of redistribution from another compartment. In rats, higher amounts of **L2** were detected both in plasma and urine, not solely corresponding to the 10‐fold increased dose. **L2** showed a half‐life of 37±12 min, a clearance of 9.84±5.0 mL kg^−1^ min^−1^ and a mean residence time 41±18 min in rat plasma in vivo, exceeding the concentration of its on‐target affinity for at least 6 h. As a result, the overall drug exposure in rat plasma was higher than in mice with an area under the curve (AUC) of 19.7±12.8 μM h. In agreement with its strong hydrophilicity, **L2** showed a rather low distribution volume (*V*_D_=0.46 L kg^−1^). Importantly, high levels of **L2** were present in rat urine even 24 h after administration.

## Conclusion

We previously reported highly potent LecA inhibitors which unfortunately suffered from very low aqueous solubility and limited stability, both precluding thermodynamic and biological analysis of these bisacylhydrazones.^[33]^ Here, we replaced this disadvantageous linker motif using bioisosters, systematic linker variation and solubilization tags. We now succeeded to increase solubility over 1000‐fold for the sulfonated ligand **L2** and also increased stability while maintaining the nanomolar on target activity for LecA. The pyridine‐based ligand **H2** showed a further increased affinity of 9.9 nM by SPR. It is worth to note that low nanomolar binding affinities associated with a strong divalent potency boost were achieved for all synthesized compounds, with a single exception of the longest acrylamide‐based ligand **F1**. The strongly increased solubility finally allowed to analyze the thermodynamic binding profile of selected ligands with LecA by microcalorimetry and complement the binding kinetics by SPR.

Importantly, we could now analyze divalent ligands **H2** and **L2** and their monovalent analogs **G2** and **K2** in cellular assays using flow cytometry and confocal microscopy to determine their efficacy to block LecA binding to host cell receptors. Binding of LecA to H1299 cells was remarkably decreased in presence of 10 μM **H2** and **L2**. Moreover, we demonstrated for the first time that LecA impairs cell migration of human epithelial cells, similarly to the fucose‐binding lectin LecB.^[36]^ Divalent sulfonate **L2** restored the migration of injured epithelial cells to a physiological rate in a scratch‐wound assay. Finally, we also analyzed the effect of **L2** on the invasion of live bacteria into human host cells, since *P. aeruginosa* can invade non‐phagocytic cells. In these infection experiments, we found that **L2** efficiently decreased LecA‐mediated bacterial invasiveness, significantly better than galactoside PNPG.

Taken together, the novel divalent LecA inhibitor **L2** is a promising lead molecule to combat *P. aeruginosa* virulence, such as host cell binding, bacterial invasion and the detrimental effects of LecA on wound healing. Thus, the inhibition of LecA‐mediated virulence provides an alternative treatment option for the highly problematic infections with *P. aeruginosa*. The pharmacokinetics study revealed renal excretion and long‐lasting presence of **H2** and **L2** in urine, opening further treatment options of urinary tract infections.

## Conflict of interest

The authors declare no conflict of interest.

1

## Supporting information

As a service to our authors and readers, this journal provides supporting information supplied by the authors. Such materials are peer reviewed and may be re‐organized for online delivery, but are not copy‐edited or typeset. Technical support issues arising from supporting information (other than missing files) should be addressed to the authors.

Supporting Information

## Data Availability

The data that support the findings of this study are available in the Supporting Information of this article.
