# Supplementary material for: Neutralizing the Impact of the Virulence Factor LecA from Pseudomonas aeruginosa on Human Cells with New Glycomimetic Inhibitors
Source: Angew Chem Int Ed Engl. 2023 Jan 10;62(7):e202215535. doi: 10.1002/anie.202215535 (PMC10107299; doi:10.1002/anie.202215535)
Supplement: Supplementary file 1 — Supporting Information [file ANIE-62-0-s001.pdf]

Angewandte Chemie
Eine Zeitschrift der Gesellschaft Deutscher Chemiker

Supporting Information

Neutralizing the Impact of the Virulence Factor LecA from *Pseudomonas aeruginosa* on Human Cells with New Glycomimetic Inhibitors

E. Zahorska, F. Rosato, K. Stober, S. Kuhaudomlarp, J. Meiers, D. Hauck, D. Reith, E. Gillon, K. Rox, A. Imberty, W. Römer, A. Titz**

General experimental details

Commercial chemicals and solvents were used without further purification.

Thin layer chromatography (TLC) was performed using silica gel 60 aluminum plates containing fluorescence indicator (Merck KGaA, Darmstadt, Germany) and developed under UV light (254 nm) and using a molybdate solution (0.02 M solution of $(NH_4)_4Ce(SO_4)_4 \cdot 2\ H_2O$ and $(NH_4)_6Mo_7O_{24} \cdot 4\ H_2O$ in aqueous 10% H_2SO_4) or a potassium permanganate solution (3 g of $KMnO_4$, 20 g of K_2CO_3 in 5 mL of 5% NaOH and 300 mL of water) with heating.

Medium pressure liquid chromatography (MPLC) was performed on a Teledyne Isco Combiflash Rf200 system using normal phase self-packed silica gel columns (60 Å, 400 mesh particle size, Fluka) or reverse-phase pre-packed silica gel 60 Å columns from Macherey-Nagel (C_{18} ec, endcapped). Preparative high-pressure liquid chromatography (HPLC) was performed on Waters 2545 Binary Gradient Module with a Waters 2489 UV/Vis detector using a RP-18 column (250/21 Nucleodur C18 Gravity SB, 5 µm from Macherey-Nagel, Germany).

Analytical HPLC-MS was performed on a Thermo Dionex Ultimate 3000 HPLC coupled to a Bruker amaZon SL mass spectrometer, with UV detection at 254 nm using a RP-18 column (100/2 Nucleoshell RP18plus, 2.7 µm from Macherey-Nagel, Germany) as stationary phase. High resolution mass spectrometry (HRMS) was performed on an Ultimate 3000 UPLC system coupled to a Q Exactive Focus Orbitrap system with HESI source (Thermo Fisher, Dreieich, Germany). The UPLC was operated with a C18 column (EC 150/2 Nucleodur C18 Pyramid, 3 µm from Macherey-Nagel, Germany).

^{1}H-NMR and ^{13}C-NMR spectra were recorded on a Bruker Avance III 500 UltraShield spectrometer at 500 MHz and 126 MHz. Chemical shifts (δ) are given in ppm and were calibrated on residual solvent peaks: : $CDCl_3$ (^{1}H-NMR δ = 7.26 ppm, ^{13}C-NMR δ = 77.0 ppm), D_2O (^{1}H-NMR δ = 4.79 ppm), MeOH-d_4 (^{1}H-NMR δ = 3.31 ppm, ^{13}C-NMR δ = 49.0 ppm), DMSO-d_6 (^{1}H-NMR δ = 2.50 ppm, ^{13}C-NMR δ = 39.51 ppm).[1] Deuterated solvents were purchased from Eurisotop (Saarbrücken, Germany). Multiplicities are specified as s = singlet, d = doublet, t = triplet, q = quartet, m = multiplet. The spectra were assigned with the help of ^{1}H,^{1}H-COSY; ^{1}H,^{13}C-HSQC and ^{1}H,^{13}C-HMBC experiments.

Compound synthesis

4-O-(2',3',4',6'-tetra-O-acetyl-β-D-galactopyranosyl)trans-*p*-coumaric acid benzyl ester (4)

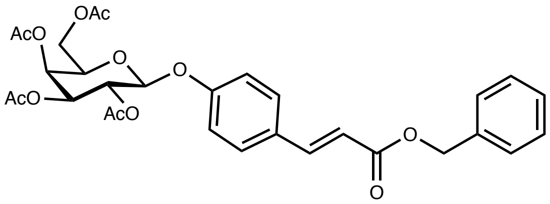

Benzyl *p*-coumarate was synthesised in analogy to Guo *et. al.*.[2] *p*-Coumaric acid (1.0 g, 6.2 mmol) was dissolved in dimethylformamide (30 mL) and Na_2CO_3 (1.5 g, 14.5 mmol) was added. Benzyl bromide (1.5 mL, 12.5 mmol) was added dropwise and stirred overnight. The reaction was concentrated *in vacuo*, diluted with water and the product was extracted into ethyl acetate, washed with half satd. brine, dried over anhydrous Na_2SO_4, filtered and concentrated *in vacuo*. The crude product was purified by normal phase MPLC (petrol ether/ethyl acetate, gradient of 10-40% ethyl acetate) to give pure benzyl *p*-coumarate (1.3 g, 5.0 mmol, 80%).

β-D-galactopyranose pentaacetate (**3**, 1.0 g, 2.6 mmol) and benzyl *p*-coumarate (1.3 g, 5.0 mmol) were dissolved in dry chloroform (20 mL) in a round bottom flask with powdered activated molecular sieve (3 Å). The reaction mixture was cooled to 0 °C and $BF_3{\cdot}OEt_2$ (1.9 mL, 15.2 mmol) was added dropwise. The mixture was allowed to warm to room temperature (r. t.) and stirred overnight. The reaction was poured over ice cold satd. $NaHCO_3$ solution and diluted with dichloromethane. The separated organic phase was washed with satd. $NaHCO_3$ solution and half satd. brine, dried over anhydrous Na_2SO_4, filtered and concentrated *in vacuo*. The product **4** was purified by normal phase MPLC (petrol ether/ethyl acetate, gradient of 20-40% ethyl acetate). Compound **4** (1.1 g, 2.0 mmol, 76%) was obtained as a white solid.

^{1}H NMR (500 MHz, $CDCl_3$) δ 7.68 (d, *J* = 16.0 Hz, 1H, C<u>H</u>=CHCOOBn), 7.50 – 7.44 (m, 2H, ArH), 7.44 – 7.32 (m, 5H, Bn), 7.02 – 6.97 (m, 2H, ArH), 6.39 (d, *J* = 16.0 Hz, 1H, CH=C<u>H</u>COOBn), 5.50 (dd, *J* = 10.5, 7.9 Hz, 1H, H-2), 5.46 (dd, *J* = 3.5, 1.1 Hz, 1H, H-4), 5.24 (s, 2H, Bn), 5.11 (dd, *J* = 10.4, 3.4 Hz, 1H, H-3), 5.08 (d, *J* = 7.9 Hz, 1H, H-1), 4.26 – 4.12 (m, 2H, H-6), 4.08 (ddd, *J* = 7.2, 6.2, 1.2 Hz, 1H, H-5), 2.19 (s, 3H, CH_3), 2.06 (s, 6H, CH_3), 2.02 (s, 3H, CH_3).

^{13}C NMR (126 MHz, $CDCl_3$) δ 170.51 (1C, C=O), 170.37 (1C, C=O), 170.28 (1C, C=O), 169.52 (1C, C=O), 167.01 (1C, C=O), 158.43 (1C, ArC), 144.35 (1C, <u>C</u>H=CHCOOBn), 136.14 (1C, Bn), 129.79 (2C, ArCH), 129.63 (1C, ArC), 128.75 (2C, Bn), 128.47 (2C, Bn), 128.43 (1C, Bn), 117.12 (2C, ArCH), 116.88 (1C, CH=<u>C</u>HCOOBn), 99.16 (1C, C-1), 71.26 (1C, C-

5), 70.86 (1C, C-3), 68.57 (1C, C-2), 66.89 (1C, C-4), 66.51 (1C, Bn), 61.47 (1C, C-6), 20.89 (1C, CH_3), 20.83 (2C, CH_3), 20.75 (1C, CH_3).

HPLC-MS: $[C_{30}H_{32}O_{12} + NH_4]^+$ calcd. 602.22, found 602.17.

HRMS: $[C_{30}H_{32}O_{12} + NH_4]^+$ calcd. 602.2232, found 602.2234.

4-O-β-D-galactopyranosyl trans-*p*-coumaric acid (1)

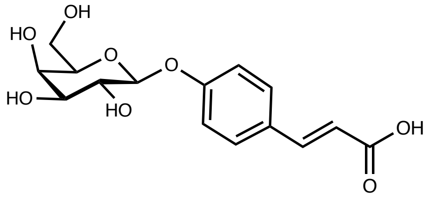

Compound **4** (1.1 g, 2.0 mmol) was suspended in methanol (48 mL). Water (32 mL) and sodium hydroxide solution (16 mL, 1 M, 16 mmol) were added. The reaction mixture was heated to 50 °C and stirred for 2 h when the reaction was neutralised with Amberlite IR 120/H^+ and concentrated *in vacuo*. After purification by normal phase MPLC (dichloromethane/methanol with 1% formic acid, gradient of 1-20% methanol), compound **1** (554.2 mg, 1.7 mmol, 76%) was obtained as a white solid. Synthesis of compound **1** was first described by Takada *et. al.*.[3]

^{1}H NMR (500 MHz, MeOH-d_4) δ 7.61 (d, *J* = 15.9 Hz, 1H, C$\underline{H}$=CHCOOH), 7.57 – 7.51 (m, 2H, ArCH), 7.16 – 7.11 (m, 2H, ArCH), 6.37 (d, *J* = 16.0 Hz, 1H, CH=C$\underline{H}$COOH), 4.92 (d, *J* = 7.8 Hz, 1H, H-1), 3.93 – 3.90 (m, 1H, H-4), 3.84 – 3.69 (m, 4H. H-2, H-5, H-6), 3.59 (dd, *J* = 9.7, 3.4 Hz, 1H, H-3).

^{13}C NMR (126 MHz, MeOH-d_4) δ 171.24 (1C, COOH), δ 160.82 (1C, ArC), 145.43 (1C, $\underline{C}$H=CHCOOH), 130.63 (2C, ArCH), 130.05 (1C, ArC), 118.00 (3C, ArCH, CH=$\underline{C}$HCOOH), 102.49 (1C, C-1), 77.07 (1C, C-5), 74.81 (1C, C-3), 72.18 (1C, C-2), 70.20 (1C, C-4), 62.40 (1C, C-6).

HPLC-MS: $[C_{15}H_{18}O_8 + HCOO]^-$ calcd. 371.10, found 370.94.

HRMS: $[C_{15}H_{18}O_8 + HCOO]^-$ calcd. 371.0984, found 371.0982.

Methyl 4-((2',3',4',6'-tetra-O-acetyl)-β-D-galactopyranosyl)oxy) 3-phenylpropanoate (5)

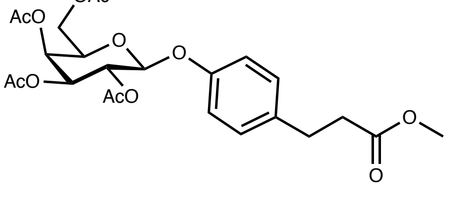

β-D-galactopyranose pentaacetate (**3**, 1.5 g, 3.9 mmol) and methyl 3-(4-hydroxyphenyl)propanoate (1.4 g, 7.8 mmol) were dissolved in dry dichloromethane (20 mL)

in a round bottom flask with powdered activated molecular sieves (3 Å). The reaction mixture was cooled to 0 °C and $BF_3 \cdot OEt_2$ (2.4 mL, 19.5 mmol) was added dropwise, it was then allowed to warm to room temperature and stirred was continued overnight. The reaction was poured over ice cold satd. $NaHCO_3$ solution and diluted with dichloromethane. The organic phase was separated and washed with satd. $NaHCO_3$ solution and half satd. brine, dried over anhydrous Na_2SO_4, filtered and concentrated *in vacuo*. After purification by normal phase MPLC (petrol ether/ethyl acetate, gradient of 15-50% ethyl acetate), compound **5** (1.8 g, 3.4 mmol, 86%) was obtained as a white solid.

^1H NMR (500 MHz, $CDCl_3$) δ 7.15 – 7.08 (m, 2H, ArH), 6.95 – 6.89 (m, 2H, ArH), 5.50 – 5.43 (m, 2H, H-2, H-4), 5.09 (dd, *J* = 10.5, 3.4 Hz, 1H, H-3), 5.00 (d, *J* = 8.0 Hz, 1H, H-1), 4.26 – 4.12 (m, 2H, H-6), 4.04 (td, *J* = 6.7, 1.2 Hz, 1H, H-5), 3.66 (s, 3H, $CH_2CH_2COOC\underline{H}_3$), 2.90 (t, *J* = 7.7 Hz, 2H, $C\underline{H_2}CH_2COOCH_3$), 2.60 (dd, *J* = 8.2, 7.2 Hz, 2H, $CH_2C\underline{H_2}COOCH_3$), 2.18 (s, 3H, CH_3), 2.06 (s, 3H, CH_3), 2.06 (s, 3H, CH_3), 2.01 (s, 3H, CH_3).

^{13}C NMR (126 MHz, $CDCl_3$) δ 173.37 (1C, C=O), 170.50 (1C, C=O), 170.40 (1C, C=O), 170.29 (1C, C=O), 169.53 (1C, C=O), 155.60 (1C, ArC), 135.63 (1C, ArC), 129.50 (2C, ArCH), 117.20 (2C, ArCH), 99.97 (1C, C-1), 71.10 (1C, C-5), 70.99 (1C, C-3), 68.79 (1C, C-2), 67.01 (1C, C-4), 61.48 (1C, C-6), 51.78 (1C, $CH_2CH_2COO\underline{C}H_3$), 35.92 (1C, $CH_2\underline{C}H_2COOCH_3$), 30.25 (1C, $\underline{C}H_2CH_2COOCH_3$), 20.88 (1C, CH_3), 20.81 (2C, CH_3), 20.73 (1C, CH_3).

HPLC-MS: $[C_{24}H_{30}O_{12} + NH_4]^+$ calcd. 528.21, found 528.21.

HRMS: $[C_{24}H_{30}O_{12} + NH_4]^+$ calcd. 528.2074, found 528.2080.

4-(β-D-galactopyranosyloxy)phenylpropanoic acid (2)

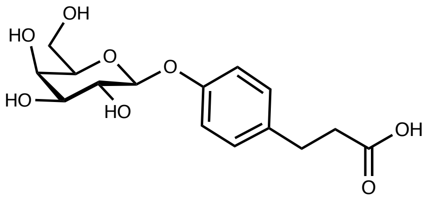

Compound **5** (179.8 mg, 0.35 mmol) was dissolved in methanol (8.5 mL). Water (5.6 mL) and sodium hydroxide solution (2.8 mL, 1 M, 2.82 mmol) were added. The reaction mixture was stirred for 1 h at r.t, then neutralised with Amberlite IR 120/H^+ and concentrated *in vacuo*. After purification by normal phase MPLC (dichloromethane/methanol with 1% formic acid, gradient of 1-20% methanol), compound **2** (107.5 mg, 0.33 mmol, 93%) was obtained as a white solid.

^1H NMR (500 MHz, DMSO-d_6) δ, 7.15 – 7.09 (m, 2H, ArH), 6.95 – 6.89 (m, 2H, ArH), 4.75 (d, *J* = 7.6 Hz, 1H, H-1), 3.69 (d, *J* = 3.3 Hz, 1H, H-4), 3.57 – 3.44 (m, 4H, H-2, H-5, H-6),

3.38 (dd, J = 9.5, 3.3 Hz, 1H, H-3), 2.75 (t, J = 7.6 Hz, 2H, C<u>H</u>$_2$CH$_2$COOCH$_3$), 2.47 (t, J = 7.6 Hz, 2H, CH$_2$C<u>H</u>$_2$COOCH$_3$).

^{13}C NMR (126 MHz, DMSO-d_6) δ 174.09 (1C, C=O), 155.85 (1C, ArC), 134.16 (1C, ArC), 129.05 (2C, ArCH), 116.15 (2C, ArCH), 101.17 (1C, C-1), 75.46 (1C, C-5), 73.33 (1C, C-3), 70.30 (1C, C-2), 68.13 (1C, C-4), 60.38 (1C, C-6), 35.80 (1C, CH$_2$<u>C</u>H$_2$COOH), 29.69 (1C, <u>C</u>H$_2$CH$_2$COOH).

HPLC-MS: [$C_{15}H_{20}O_8$ - H]$^-$ calcd. 327.11, found 326.95.

HRMS: [$C_{15}H_{20}O_8$ - H]$^-$ calcd. 327.1085, found 327.1087.

<u>General procedure for the synthesis of bis-anilines **C–F**</u>

Corresponding di-halogenated hydrocarbons (1 eq.), 4-nitrophenol (4 eq.) and potassium carbonate (3 eq.) were dissolved in dry dimethylformamide in a microwave reaction vial. The vial was sealed and the mixture was irradiated in a Discover SP Sequential Microwave Synthesis System (CEM Corporation, North Carolina, USA) with maximum power 300 W at 70 °C for 11 h – 4 days (for **C** 10 days in oil bath, no irradiation). After cooling, the reaction was diluted with ethyl acetate and washed with satd. aqueous $NaHCO_3$, dried over anhydrous Na_2SO_4, filtered and concentrated *in vacuo*. Bis-nitro intermediates were purified by normal phase MPLC (toluene with 1% ethyl acetate and 1% triethylamine, isocratic) or in case of **E** by C18 column MPLC chromatography (water/acetonitrile with 0.1% formic acid, gradient of 40-75% acetonitrile). Pure bis-nitro intermediate was then dissolved in dichloromethane/methanol (3:1, for **C** 2:1) and 0.1 eq Pd/C (10 wt-%) was added. After three vacuum/H_2 cycles the reaction was stirred under H_2 atmosphere (1 atm) overnight. The reaction was filtered over celite and concentrated *in vacuo*. Pure products were obtained without further purification. Analytical data of compounds **C–F** match the literature.[4–6]

Bis(4-aminophenoxy) methane (C)

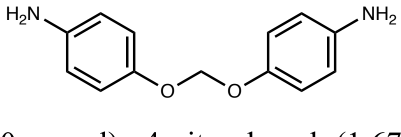

Dichloromethane (192 µL, 3.0 mmol), 4-nitrophenol (1.67 g, 12 mmol) and potassium carbonate (1.24 g, 9.0 mmol) in 9 mL dimethylformamide were used following the general procedure for synthesis of bis-anilines to give compound **C** (326 mg, 1.42 mmol, 47% over two steps) as a light-brown solid.

^{1}H NMR (500 MHz, MeOH-d$_4$) δ 6.91 – 6.84 (m, 4H, ArH), 6.74 – 6.68 (m, 4H, ArH), 5.52 (s, 2H, CH$_2$).

^{13}C NMR (126 MHz, MeOH-d4) δ 151.77 (2C, ArC), 142.80 (2C, ArC), 118.98 (4C, ArCH), 118.07 (4C, ArCH), 94.31 (1C, CH_2).
HPLC-MS: [C13$H_{14}N_2O_2$ + H]$^+$ calcd. 231.11, found 231.04.
HRMS: [$C_{13}H_{14}N_2O_2$ + H]$^+$ calcd. 231.1128, found 231.1125.

1,2-bis(4-aminophenoxy) ethane (D)

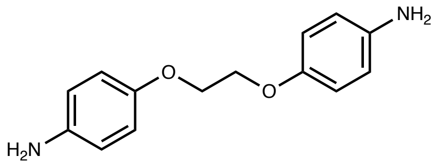

1,2-Dichloroethane (181 µL, 2.3 mmol), 4-nitrophenol (1.11 g, 8 mmol) and potassium carbonate (829 mg, 6.0 mmol) in 6 mL dimethylformamide were used following the general procedure for synthesis of bis-anilines to give compound **D** (182 mg, 0.75 mmol, 33% over two steps) as a brown-gray solid.
^1H NMR (500 MHz, DMSO-d_6) δ 6.81 – 6.71 (m, 4H, ArH), 6.70 – 6.54 (m, 4H, ArH), 4.12 (s, 4H, CH_2).
^{13}C NMR (126 MHz, DMSO-d_6) δ 150.95 (2C, ArC), 139.66 (2C, ArC), 116.45 (4C, ArCH), 115.44, (4C, ArCH) 66.93 (2C, CH_2).
HPLC-MS: [$C_{14}H_{16}N_2O_2$ + H]$^+$ calcd. 245.13, found 245.05.
HRMS: [$C_{14}H_{16}N_2O_2$ + H]$^+$ calcd. 245.1285, found 245.1281.

1,3-bis(4-aminophenoxy) propane (E)

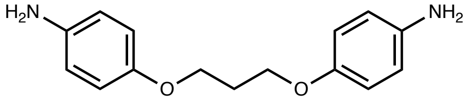

1,3-Dichloropropane (190 µL, 2.0 mmol), 4-nitrophenol (1.11 g, 8 mmol) and potassium carbonate (829 mg, 6.0 mmol) in 6 mL dimethylformamide were used following the general procedure for synthesis of bis-anilines to give compound **E** (342 mg, 1.32 mmol, 67% over two steps) as a brown-gray solid.
^1H NMR (500 MHz, MeOH-d4) δ 6.80 – 6.70 (m, 8H, ArH), 4.06 (t, *J* = 6.2 Hz, 4H, $C\underline{H}_2CH_2C\underline{H}_2$), 2.19 – 2.09 (m, 2H, $CH_2C\underline{H}_2CH_2$).
^{13}C NMR (126 MHz, MeOH-d4) δ 154.21 (2C, ArC), 140.39 (2C, ArC), 118.73 (4C, ArCH), 116.67 (4C, ArCH), 66.38 (2C, $\underline{C}H_2CH_2\underline{C}H_2$), 30.67 (1C, $CH_2\underline{C}H_2CH_2$).
HPLC-MS: [$C_{15}H_{18}N_2O_2$ + H]$^+$ calcd. 259.14, found 259.06.
HRMS: [$C_{15}H_{18}N_2O_2$ + H]$^+$ calcd. 259.1441, found 259.1438.

1,4-bis(4-aminophenoxy) butane (F)

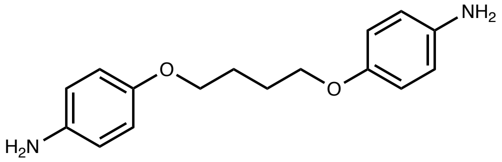

1,4-Dibromobutane (236 µL, 2.0 mmol), 4-nitrophenol (1.11 g, 8 mmol) and potassium carbonate (829 mg, 6.0 mmol) in 6 mL dimethylformamide were used following the general procedure for synthesis of bis-anilines to give compound **F** (562 mg, 2.1 mmol, quant. over two steps) as a brown-gray solid.

^{1}H NMR (500 MHz, MeOH-d_4) δ 6.77 (s, 8H, ArH), 4.00 – 3.92 (m, 4H, $C\underline{H}_2CH_2CH_2C\underline{H}_2$), 1.95 – 1.84 (m, 4H, $CH_2C\underline{H}_2C\underline{H}_2CH_2$).

^{13}C NMR (126 MHz, MeOH-d_4) δ 154.75 (2C, ArC), 139.21 (2C, ArC), 119.17 (4C, ArCH), 116.63 (4C, ArCH), 69.36 (2C, $\underline{C}H_2CH_2CH_2\underline{C}H_2$), 27.26 (2C, $CH_2\underline{C}H_2\underline{C}H_2CH_2$).

HPLC-MS: $[C_{16}H_{20}N_2O_2 + H]^+$ calcd. 273.16, found 273.07.

HRMS: $[C_{16}H_{20}N_2O_2 + H]^+$ calcd. 273.1598, found 273.1594.

<u>General procedure for the synthesis of bis-aminopyridines **H–J**</u>

Corresponding diol (1 eq.), 2-chloro-5-nitropyridine (2-3 eq.) and sodium hydride (3 eq., 60% in mineral oil) were dissolved in dry dimethylformamide and stirred at room temperature for 1 h – 2 d (for **H** 3.5 eq. of potassium carbonate was used, T = 65 °C for 5 d). The reaction was diluted with ice cold water and dichloromethane. Organic phase was separated and washed with half satd. brine, dried over anhydrous Na_2SO_4, filtered and concentrated *in vacuo*. Bis-nitro intermediate was purified by normal phase MPLC (petrol ether/ethyl acetate, gradient of 5-20% ethyl acetate). Pure bis-nitro intermediate was then dissolved in dichloromethane/methanol (2:1) and 0.1 eq Pd/C (10 wt-%) was added. After three vacuum/H_2 cycles the reaction was stirred under H_2 atmosphere (1 atm) for 3–4 h. The reaction was filtered over celite and concentrated *in vacuo*. Pure products were obtained without further purification.

1,2-bis((5-aminopyridin-2-yl)oxy)ethane (H)

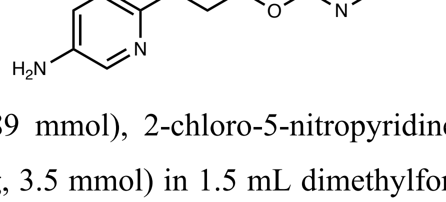

Ethylene glycol (50 µL, 0.89 mmol), 2-chloro-5-nitropyridine (436 mg, 2.75 mmol) and potassium carbonate (438 mg, 3.5 mmol) in 1.5 mL dimethylformamide were used following

the general procedure for synthesis of bis-aminopyridines to give compound **H** (69.0 mg, 0.28 mmol, 31% over two steps) as a light-brown solid.

^{1}H NMR (500 MHz, MeOH-d$_4$) δ 7.61 (dd, *J* = 2.9, 0.7 Hz, 2H ArH), 7.17 (dd, *J* = 8.7, 2.9 Hz, 2H, ArH), 6.65 (dd, *J* = 8.7, 0.7 Hz, 2H, ArH), 4.44 (s, 4H, CH_2).

^{13}C NMR (126 MHz, MeOH-d$_4$) δ 158.46 (2C, ArC), 139.63 (2C, ArC), 133.72 (2C, ArCH), 129.40 (2C, ArCH), 111.82 (2C, ArCH), 65.88 (2C, CH_2).

HPLC-MS: $[C_{12}H_{14}N_4O_2 + Na]^+$ calcd. 269.10, found 269.05.

HRMS: $[C_{12}H_{14}N_4O_2 + H]^+$ calcd. 247.1190, found 247.1187.

1,3-bis((5-aminopyridin-2-yl)oxy)propane (I)

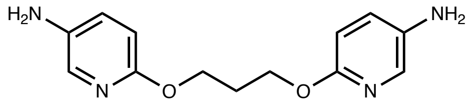

Propane-1,3-diol (50 µL, 0.89 mmol), 2-chloro-5-nitropyridine (279 mg, 1.76 mmol) and sodium hydride (83 mg, 2.1 mmol) in 1.5 mL dimethylformamide were used following the general procedure for synthesis of bis-aminopyridines to give compound **I** (111.4 mg, 0.43 mmol, 62% over two steps) as brown solid.

^{1}H NMR (500 MHz, MeOH-d$_4$) δ 7.60 (d, *J* = 2.8 Hz, 2H, ArH), 7.17 (dd, *J* = 8.7, 2.9 Hz, 2H, ArH), 6.64 (d, *J* = 8.7 Hz, 2H, ArH), 4.29 (t, *J* = 6.3 Hz, 4H, $C\underline{H}_2CH_2C\underline{H}_2$), 2.16 (p, *J* = 6.3 Hz, 2H, $CH_2C\underline{H}_2CH_2$).

^{13}C NMR (126 MHz, MeOH-d$_4$) δ 158.76 (2C, ArC), 139.35 (2C, ArC), 133.81 (2C, ArCH), 129.48 (2C, ArCH), 111.65 (2C, ArCH), 64.18 (2C, $\underline{C}H_2CH_2\underline{C}H_2$), 30.21 (1C, $CH_2\underline{C}H_2CH_2$).

HPLC-MS: $[C_{13}H_{16}N_4O_2 + H]^+$ calcd. 261.13, found 261.07.

HRMS: $[C_{13}H_{16}N_4O_2 + H]^+$ calcd. 261.1346, found 261.1340.

1,4-bis((5-aminopyridin-2-yl)oxy)butane (J)

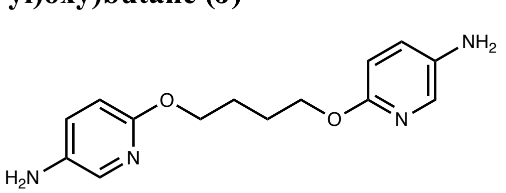

Butane-1,4-diol (50 µL, 0.56 mmol), 2-chloro-5-nitropyridine (284 mg, 1.79 mmol) and sodium hydride (72 mg, 1.9 mmol) in 1.5 mL dimethylformamide were used following the general procedure for synthesis of bis-aminopyridines to give the bis-nitro intermediate (143 mg, 0.43 mmol, 76%). The bis-nitro intermediate (125 mg, 0.38 mmol) was reduced to give pure compound **J** (104 mg, 0.38 mmol, quant.) as a brown solid.

^{1}H NMR (500 MHz, MeOH-d$_4$) δ 7.60 (d, *J* = 2.9 Hz, 2H, ArH), 7.17 (dd, *J* = 8.7, 2.9 Hz, 2H, ArH), 6.62 (d, *J* = 8.7 Hz, 2H, ArH), 4.22 – 4.16 (m, 4H, $C\underline{H}_2CH_2CH_2C\underline{H}_2$), 1.93 – 1.85 (m, 4H, $CH_2C\underline{H}_2C\underline{H}_2CH_2$).
^{13}C NMR (126 MHz, MeOH-d$_4$) δ 158.86 (2C, ArC), 139.36 (2C, ArC), 133.78 (2C, ArCH), 129.47 (2C, ArCH), 111.62 (2C, ArCH), 67.09 (2C, $\underline{C}H_2CH_2CH_2\underline{C}H_2$), 27.05 (2C, $CH_2\underline{C}H_2\underline{C}H_2CH_2$).
HPLC-MS: $[C_{14}H_{18}N_4O_2 + H]^+$ calcd. 275.15, found 275.10.
HRMS: $[C_{14}H_{18}N_4O_2 + H]^+$ calcd. 275.1503, found 275.1497.

6,6'-(ethane-1,2-diyl)bis(3-aminobenzenesulfonic acid) (L)

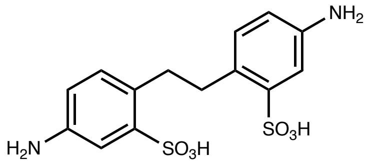

Compound **L** was synthesized according to Bazanova *et. al.*.[7] 4,4'-Diaminostilbene-2,2'-disulfonic acid (**13**, 235.5 mg, 0.64 mmol) was completely dissolved in water (10 mL) after addition of 1 drop of satd. NaOH aqueous solution, and then Raney Ni slurry was added. After three vacuum/H_2 cycles the reaction was stirred under H_2 atmosphere (1 atm) for 4 days. The reaction was filtered over celite and lyophilized. Because conversion was incomplete, the reaction crude was re-dissolved in in water (5 mL) and 1 drop of satd. NaOH aqueous solution. Raney Ni slurry was added and after three vacuum/H_2 cycles the reaction was stirred under H_2 atmosphere (1 atm) for 2 additional days. The reaction was filtered over celite and lyophilized. Reaction crude was re-dissolved in water (10 mL) and pure product **L** (150.6 mg, 0.40 mmol, 64%) was precipitated after acidification with HCl (2 M) as a white solid.
^{1}H NMR (500 MHz, D_2O) δ 7.28 (dd, *J* = 2.4, 0.9 Hz, 2H, ArH), 7.13 (d, *J* = 8.0 Hz, 2H, ArH), 6.85 (ddd, *J* = 8.1, 2.5, 0.9 Hz, 2H, ArH), 3.15 (s, 4H, CH_2).
^{13}C NMR (126 MHz, D_2O) δ 144.12 (2C, ArC), 141.07 (2C, ArC), 132.63 (2C, ArCH), 130.22 (2C, ArC), 119.20 (2C, ArCH), 114.50 (2C, ArCH), 33.46 (2C, CH_2).
HPLC-MS: $[C_{14}H_{16}N_2O_6S_2 - H]^-$ calcd. 371.0377, found 370.93.

General procedure for synthesis of amides

Corresponding anilines **A–F** or aminopyridines **G–J** (1 eq.) and carboxylate-bearing galactosides **1** or **2** (2.5 eq.) were dissolved in dry dimethylformamide. HBTU (2.5 eq.) and DIPEA (5 eq.) were added. Reactions were stirred at r.t. until completion, then diluted with

water and lyophilized. The products were purified by MPLC or preparative HPLC (C18, water/acetonitrile with 0.1% formic acid).

Monovalent ligand A1

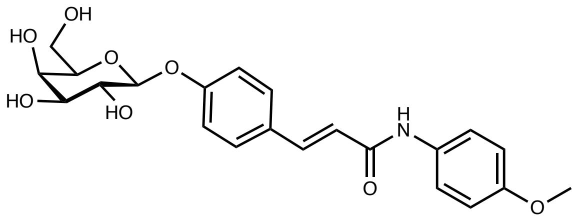

4-methoxyaniline (**A**, 20.8 mg, 169 µmol), coumarate **1** (33.9 mg, 104 µmol) and HBTU (51.4 mg, 136 µmol) were dissolved in dimethylformamide (1 mL) and DIPEA (30 µL, 172 µmol) was added. The reaction was stirred at r.t. for 1 h, then diluted with water and lyophilized. The product was purified by preparative reverse-phase HPLC (water/acetonitrile with 0.1% formic acid, gradient of 15-40% acetonitrile) and compound **A1** (15.9 mg, 37 µmol, 35%) was obtained as a white solid.

^{1}H NMR (500 MHz, DMSO-d_6) δ 10.00 (s, 1H, NH), 7.63 – 7.58 (m, 2H, ArH), 7.58 – 7.53 (m, 2H, ArH), 7.51 (d, J = 15.7 Hz, 1H, CH=CHCONH), 7.11 – 7.05 (m, 2H, ArH), 6.94 – 6.86 (m, 2H, ArH), 6.66 (d, J = 15.7 Hz, 1H, CH=CHCONH), 4.89 (d, J = 7.7 Hz, 1H, H-1), 3.73 (s, 3H, OCH_3), 3.71 (d, J = 3.0 Hz, 1H, H-4), 3.63 – 3.47 (m, 4H, H-2, H-5, H-6), 3.44 – 3.40 (m, 1H, H-3).

^{13}C NMR (126 MHz, DMSO-d_6) δ 163.37 (1C, C=O), 158.65 (1C, ArC), 155.23 (1C, ArC), 139.31 (1C, CH=CHCONH), 132.58 (1C, ArC), 129.11 (2C, ArCH), 128.43 (1C, ArC), 120.65 (2C, ArCH), 120.34 (1C, CH=CHCONH), 116.61 (2C, ArCH), 113.95 (2C, ArCH), 100.69 (1C, C-1), 75.59 (1C, C-5), 73.28 (1C, C-3), 70.23 (1C, C-2), 68.13 (1C, C-4), 60.37 (1C, C-6), 55.19 (1C, OCH_3).

HPLC-MS: $[C_{22}H_{25}NO_8 + H]^+$ calcd. 432.17, found 432.13.

HRMS: $[C_{22}H_{25}NO_8 + H]^+$ calcd. 432.1653, found 432.1652.

Divalent Ligand B1

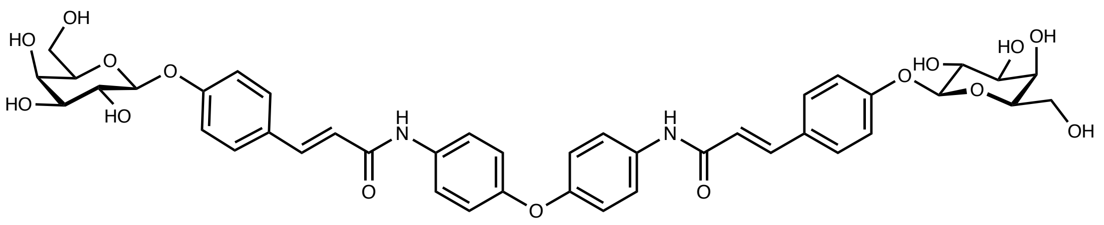

4,4'-Oxydianiline **B** (10.1 mg, 50.4 µmol), compound **1** (38.2 mg, 117 µmol) and HBTU (53.1 mg, 140 µmol) were dissolved in dimethylformamide (1 mL) and DIPEA (50 µL, 287 µmol)

was added. The reaction was stirred at r.t. overnight, then dried *in vacuo*. The product was purified by preparative reverse-phase HPLC (water/acetonitrile with 0.1% formic acid, gradient of 20-35% acetonitrile) and compound **B1** (28.3 mg, 34.6 μmol, 69%) was obtained as pale-yellow solid.

^{1}H NMR (500 MHz, DMSO-d_6) δ 10.18 (s, 2H, NH), 7.73 – 7.67 (m, 4H, ArH), 7.60 – 7.50 (m, 6H, ArH, C<u>H</u>=CHCONH), 7.11 – 7.04 (m, 4H, ArH), 7.02 – 6.95 (m, 4H, ArH), 6.68 (d, *J* = 15.7 Hz, 2H, CH=C<u>H</u>CONH), 4.90 (d, *J* = 7.7 Hz, 2H, H-1), 3.81 – 3.35 (m, 12H, H-2, H-3, H-4, H-5, H-6).

^{13}C NMR (126 MHz, DMSO-d_6) δ 163.64 (2C, C=O), 158.75 (2C, ArC), 152.55 (2C, ArC), 139.74 (2C, <u>C</u>H=CHCONH), 135.00 (2C, ArC), 129.24 (4C, ArCH), 128.36 (2C, ArC), 120.82 (4C, ArCH), 120.15 (2C, CH=<u>C</u>HCONH), 118.85 (4C, ArCH), 116.63 (4C, ArCH), 100.66 (2C, C-1), 75.62 (2C, C-5), 73.29 (2C, C-3), 70.24 (2C, C-2), 68.15 (2C, C-4), 60.38 (2C, C-6).

HPLC-MS: $[C_{42}H_{44}N_2O_{15} + H]^+$ calcd. 817.28, found 817.33.

HRMS: $[C_{42}H_{44}N_2O_{15} + H]^+$ calcd. 817.2814, found 817.2805.

Divalent Ligand C1

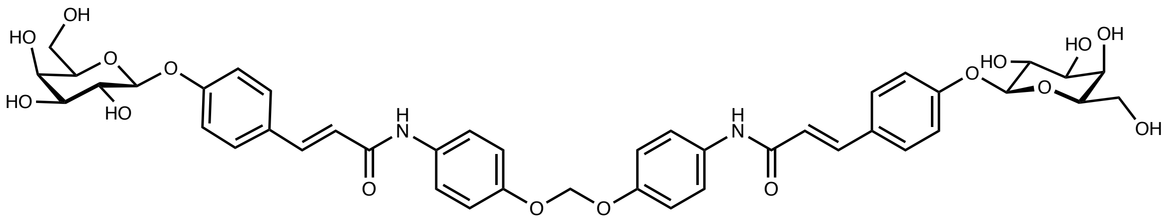

Bis(4-aminophenoxy)methane **C** (7.9 mg, 34 μmol), compound **1** (25.3 mg, 76 μmol) and HBTU (32.7 mg, 86 μmol) were dissolved in dimethylformamide (3 mL) and DIPEA (9 μL, 52 μmol) was added. The reaction was stirred at r.t. for 2 days, then dried *in vacuo*. The product was purified by preparative reverse-phase HPLC (water/acetonitrile with 0.1% formic acid, gradient of 25-45% acetonitrile) and compound **C1** (6.9 mg, 8.1 μmol, 24%) was obtained as a white solid.

^{1}H NMR (500 MHz, DMSO-d_6) δ 10.07 (s, 2H, NH), 7.67 – 7.60 (m, 4H, ArH), 7.59 – 7.54 (m, 4H, ArH), 7.52 (d, *J* = 15.6 Hz, 2H, C<u>H</u>=CHCONH), 7.12 – 7.02 (m, 8H, ArH), 6.67 (d, *J* = 15.6 Hz, 2H, CH=C<u>H</u>CONH), 5.77 (s, 2H, CH_2), 5.19 (d, *J* = 5.2 Hz, 2H, OH-2), 4.92 – 4.84 (m, 4H, H-1, OH-3), 4.66 (t, *J* = 5.5 Hz, 2H, OH-6), 4.52 (d, *J* = 4.6 Hz, 2H, OH-4), 3.71 (t, *J* = 4.1 Hz, 2H, H-4), 3.64 – 3.45 (m, 8H, H-2, H-5, H-6), 3.42 (ddd, *J* = 9.3, 5.3, 3.2 Hz, 2H, H-3).

^{13}C NMR (126 MHz, DMSO-d_6) δ 163.49 (2C, C=O), 158.68 (2C, ArC), 152.19 (2C, ArC), 139.52 (2C, <u>C</u>H=CHCONH), 134.09 (2C, ArC), 129.14 (4C, ArCH), 128.38 (2C, ArC), 120.59

(4C, ArCH), 120.23 (2C, CH=$\underline{C}$HCONH), 116.60 (4C, ArCH), 116.59 (4C, ArCH), 100.68 (2C, C-1), 90.81 (1C, CH_2), 75.58 (2C, C-5), 73.27 (2C, C-3), 70.22 (2C, C-2), 68.12 (2C, C-4), 60.36 (2C, C-6).

HPLC-MS: $[C_{43}H_{46}N_2O_{16} + H]^+$ calcd. 847.29, found 847.33

HRMS: $[C_{43}H_{46}N_2O_{16} + H]^+$ calcd. 847.2920, found 847.2924.

Divalent Ligand D1

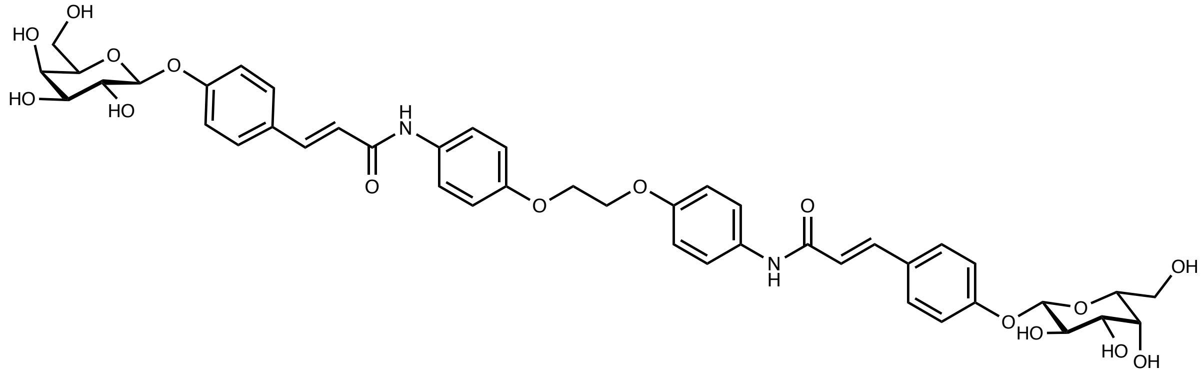

Bis(4-aminophenoxy)ethane **D** (7.3 mg, 0.03 mmol), compound **1** (21.6 mg, 66 µmol) and HBTU (25.2 mg, 66 µmol) were dissolved in dimethylformamide (4 mL) and DIPEA (7 µL, 40 µmol) was added. The reaction was stirred at r.t. for 2 days, then dried *in vacuo*. The product was purified by preparative reverse-phase HPLC (water/acetonitrile supplemented with 0.1% formic acid, gradient of 25-45% acetonitrile) and compound **D1** (4.5 mg, 5.2 µmol, 17%) was obtained as a white solid.

^{1}H NMR (500 MHz, DMSO-d_6) δ 10.04 (s, 2H, NH), 7.65 – 7.60 (m, 4H, ArH), 7.58 – 7.54 (m, 4H, ArH), 7.52 (d, J = 15.5 Hz, 2H, C$\underline{H}$=CHCONH), 7.08 (d, J = 8.7 Hz, 4H, ArH), 7.01 – 6.91 (m, 4H, ArH), 6.67 (d, J = 15.7 Hz, 2H, CH=C$\underline{H}$CONH), 4.89 (d, J = 7.7 Hz, 2H, H-1), 4.28 (s, 4H, CH_2), 3.70 (d, J = 3.3 Hz, 2H, H-4), 3.63 – 3.46 (m, 8H, H-2, H-5, H-6), 3.42 (dd, J = 9.5, 3.3 Hz, 4H, H-3).

^{13}C NMR (126 MHz, DMSO-d_6) δ 163.41 (2C, C=O), 158.67 (2C, ArC), 154.29 (2C, ArC), 139.39 (2C, $\underline{C}$H=CHCONH), 132.85 (2C, ArC), 129.16 (4C, ArCH), 128.43 (2C, ArC), 120.64 (4C, ArCH), 120.32 (2C, CH=$\underline{C}$HCONH), 116.61 (4C, ArCH), 114.65 (4C, ArCH), 100.67 (2C, C-1), 75.60 (2C, C-5), 73.28 (2C, C-3), 70.23 (2C, C-2), 68.14 (2C, C-4), 66.51 (2C, CH_2), 60.37 (2C, C-6).

HPLC-MS: $[C_{44}H_{48}N_2O_{16} + H]^+$ calcd. 861.31, found 861.36.

HRMS: $[C_{44}H_{48}N_2O_{16} + H]^+$ calcd. 861.3077, found 861.3083.

Divalent Ligand E1

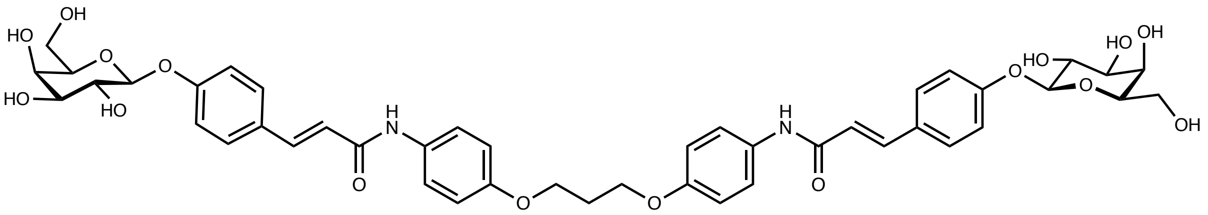

Bis(4-aminophenoxy)propane **E** (11.2 mg, 43.3 μmol), compound **1** (30.8 mg, 94.4 μmol) and HBTU (38.1 mg, 100 μmol) were dissolved in dimethylformamide (1 mL) and DIPEA (20 μL, 115 μmol) was added. The reaction was stirred at r.t. overnight, then dried *in vacuo*. The product was purified by preparative reverse-phase HPLC (water/acetonitrile with 0.1% formic acid, gradient of 20-50% acetonitrile) and compound **E1** (25.9 mg, 29.6 μmol, 68%) was obtained as a pale-yellow solid.

^{1}H NMR (500 MHz, DMSO-d_6) δ 10.02 (s, 2H, NH), 7.63 – 7.58 (m, 4H, ArH), 7.58 – 7.53 (m, 4H, ArH), 7.51 (d, *J* = 15.6 Hz, 2H, C<u>H</u>=CHCONH), 7.10 – 7.05 (m, 4H, ArH), 6.96 – 6.91 (m, 4H, ArH), 6.66 (d, *J* = 15.7 Hz, 2H, CH=C<u>H</u>CONH), 5.21 (s, 2H, OH-2), 4.89 (d, *J* = 7.7 Hz, 4H, H-1, OH-3), 4.68 (s, 2H, OH-6), 4.55 (s, 2H, OH-4), 4.10 (t, *J* = 6.2 Hz, 4H, C$\underline{H}_2$CH$_2$C$\underline{H}_2$), 3.70 (d, *J* = 3.2 Hz, 2H, H-4), 3.63 – 3.46 (m, 8H, H-2, H-5, H-6), 3.41 (dd, *J* = 9.5, 3.3 Hz, 2H, H-3), 2.20 – 2.11 (m, 2H, CH$_2$C$\underline{H}_2$CH$_2$).

^{13}C NMR (126 MHz, DMSO-d_6) δ 163.39 (2C, C=O), 158.67 (2C, ArC), 154.47 (2C, ArC), 139.36 (2C, <u>C</u>H=CHCONH), 132.70 (2C, ArC), 129.16 (4C, ArCH), 128.43 (2C, ArC), 120.63 (4C, ArCH), 120.34 (2C, CH=<u>C</u>HCONH), 116.61 (4C, ArCH), 114.60 (4C, ArCH), 100.67 (2C, C-1), 75.61 (2C, C-5), 73.28 (2C, C-3), 70.23 (2C, C-2), 68.15 (2C, C-4), 64.37 (2C, <u>C</u>H$_2$CH$_2$<u>C</u>H$_2$), 60.38 (2C, C-6), 28.77 (1C, CH$_2$<u>C</u>H$_2$CH$_2$).

HPLC-MS: $[C_{45}H_{50}N_2O_{16} + H]^+$ calcd. 875.32, found 875.40.

HRMS: $[C_{45}H_{50}N_2O_{16} + H]^+$ calcd. 875.3233, found 875.3223.

Divalent Ligand F1

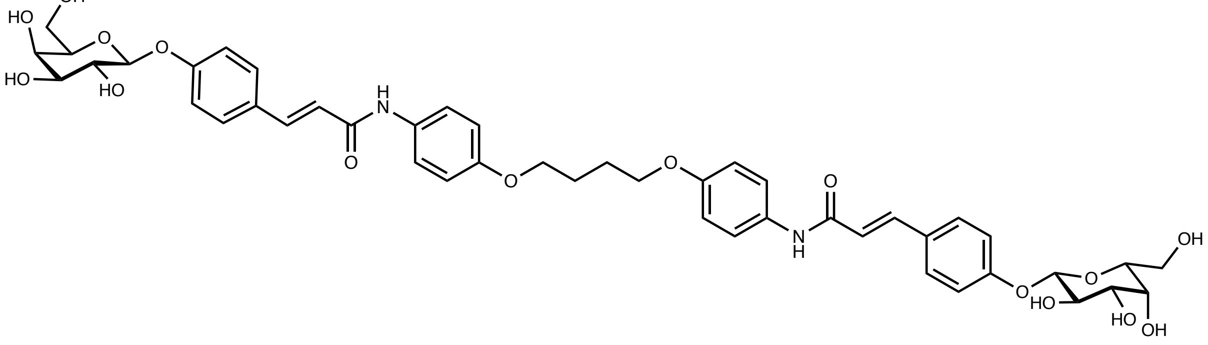

Bis(4-aminophenoxy)butane **F** (11.7 mg, 43.0 μmol), compound **1** (33.3 mg, 102 μmol) and HBTU (37.1 mg, 97.8 μmol) were dissolved in dimethylformamide (1 mL) and DIPEA (20 μL, 115 μmol) was added. The reaction was stirred at r.t. overnight, then dried *in vacuo*. The product was purified by preparative reverse-phase HPLC (water/acetonitrile with 0.1% formic acid, gradient of 25-45% acetonitrile) and compound **F1** (22.8 mg, 25.6 μmol, 60%) was obtained as a pale-yellow solid.

^{1}H NMR (500 MHz, DMSO-d_6) δ 10.00 (s, 2H, NH), 7.62 – 7.58 (m, 4H, ArH), 7.58 – 7.54 (m, 4H, ArH), 7.51 (d, *J* = 15.6 Hz, 2H, $C\underline{H}$=CHCONH), 7.08 (d, *J* = 8.7 Hz, 4H, ArH), 6.94 – 6.89 (m, 4H, ArH), 6.67 (d, *J* = 15.7 Hz, 2H, CH=$C\underline{H}$CONH), 5.19 (d, *J* = 5.2 Hz, 2H, OH-2), 4.93 – 4.84 (m, 4H, H-1, OH-3), 4.67 (t, *J* = 5.5 Hz, 2H, OH-6), 4.52 (d, *J* = 4.6 Hz, 2H, OH-4), 4.00 (d, *J* = 5.7 Hz, 4H, $C\underline{H}_2CH_2CH_2C\underline{H}_2$), 3.71 (t, *J* = 4.2 Hz, 2H, H-4), 3.63 – 3.46 (m, 8H, H-2, H-5, H-6), 3.45 – 3.39 (m, 2H, H-3), 1.90 – 1.80 (m, 4H, $CH_2C\underline{H}_2C\underline{H}_2CH_2$).

^{13}C NMR (126 MHz, DMSO-d_6) δ 163.36 (2C, C=O), 158.65 (2C, ArC), 154.61 (2C, ArC), 139.31 (2C, $\underline{C}$H=CHCONH), 132.55 (2C, ArC), 129.11 (4C, ArCH), 128.44 (2C, ArC), 120.64 (2C, ArC), 120.36 (2C, CH=$\underline{C}$HCONH), 116.61 (4C, ArCH), 114.57 (4C, ArCH), 100.69 (2C, C-1), 75.59 (2C, C-5), 73.28 (2C, C-3), 70.23 (2C, C-2), 68.14 (2C, C-4), 67.29 (2C, $\underline{C}H_2CH_2CH_2\underline{C}H_2$), 60.37 (2C, C-6), 25.50 (2C, $CH_2\underline{C}H_2\underline{C}H_2CH_2$).

HPLC-MS: $[C_{46}H_{52}N_2O_{16} + H]^+$ calcd. 889.34, found 889.42.

HRMS: $[C_{46}H_{52}N_2O_{16} + H]^+$ calcd. 889.3423, found 889.3383.

Divalent Ligand H1

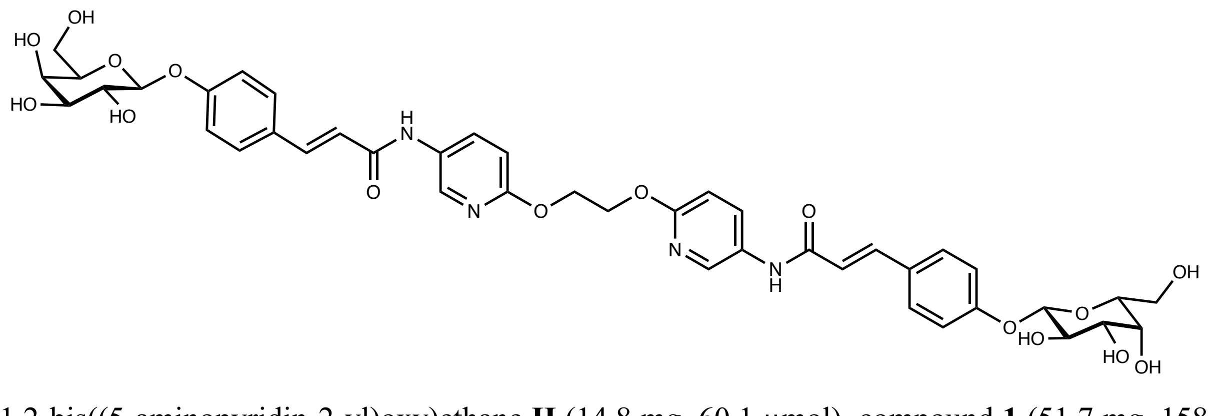

1,2-bis((5-aminopyridin-2-yl)oxy)ethane **H** (14.8 mg, 60.1 μmol), compound **1** (51.7 mg, 158 μmol) and HBTU (60.1 mg, 158 μmol) were dissolved in dimethylformamide (1.5 mL) and DIPEA (55 μL, 316 μmol) was added. The reaction was stirred at r.t. overnight, then lyophilized. The product was purified by preparative reverse-phase HPLC (water/acetonitrile supplemented with 0.1% formic acid, gradient of 15-40% acetonitrile) and compound **H1** (37.9 mg, 43.9 μmol, 73%) was obtained as a pale-yellow solid.

^{1}H NMR (500 MHz, DMSO-d_6) δ 10.19 (s, 2H, NH), 8.46 (d, *J* = 2.9 Hz, 2H, ArH), 8.02 (dd, *J* = 8.9, 2.7 Hz, 2H, ArH), 7.60 – 7.52 (m, 6H, ArH, C<u>H</u>=CHCONH), 7.11 – 7.05 (m, 4H, ArH), 6.87 (d, *J* = 8.9 Hz, 2H, ArH), 6.66 (d, *J* = 15.7 Hz, 2H, CH=C<u>H</u>CONH), 4.90 (d, *J* = 7.7 Hz, 2H, H-1), 4.56 (s, 4H, CH_2), 3.71 (d, *J* = 3.8 Hz, 2H, H-4), 3.63 – 3.46 (m, 8H, H-2, H-5, H-6), 3.42 (dd, *J* = 9.5, 3.3 Hz, 2H, H-3).

^{13}C NMR (126 MHz, DMSO-d_6) δ 163.85 (2C, C=O), 158.99 (2C, ArC), 158.78 (2C, ArC), 140.02 (2C, <u>C</u>H=CHCONH), 137.36 (2C, ArCH), 131.42 (2C, ArCH), 130.53 (2C, ArC), 129.25 (4C, ArCH), 128.25 (2C, ArC), 119.61 (2C, CH=<u>C</u>HCONH), 116.62 (4C, ArCH), 110.48 (2C, ArCH), 100.67 (2C, C-1), 75.59 (2C, C-5), 73.27 (2C, C-3), 70.22 (2C, C-2), 68.13 (2C, C-4), 64.11 (2C, CH_2), 60.36 (2C, C-6).

HPLC-MS: $[C_{42}H_{46}N_4O_{16} + H]^+$ calcd. 863.30, found 863.34.

HRMS: $[C_{42}H_{46}N_4O_{16} + H]^+$ calcd. 863.2982, found 863.2977.

Divalent Ligand I1

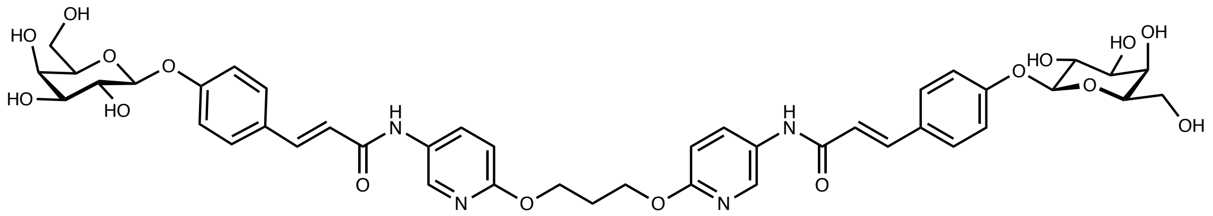

1,3-bis((5-aminopyridin-2-yl)oxy)propane **I** (14.8 mg, 56.9 µmol), compound **1** (40.4 mg, 124 µmol) and HBTU (51.4 mg, 136 µmol) were dissolved in dimethylformamide (1.5 mL) and DIPEA (50 µL, 287 µmol) was added. The reaction was stirred at r.t. overnight, then lyophilized. The product was purified by preparative reverse-phase HPLC (water/acetonitrile supplemented with 0.1% formic acid, gradient of 15-40% acetonitrile) and compound **I1** (22.7 mg, 25.9 µmol, 46%) was obtained as a white solid.

^{1}H NMR (500 MHz, DMSO-d_6) δ 10.18 (s, 2H, NH), 8.44 (d, *J* = 2.6 Hz, 2H, ArH), 8.00 (dd, *J* = 8.9, 2.7 Hz, 2H, ArH), 7.61 – 7.50 (m, 6H, ArH, C<u>H</u>=CHCONH), 7.08 (d, *J* = 8.5 Hz, 4H, ArH), 6.83 (d, *J* = 8.8 Hz, 2H, ArH), 6.66 (d, *J* = 15.7 Hz, 2H, CH=C<u>H</u>CONH), 5.20 (s, 2H, OH-2), 4.95 – 4.82 (m, 4H, H-1, OH-3), 4.67 (s, 2H, OH-6), 4.53 (s, 2H, OH-4), 4.37 (t, *J* = 6.4 Hz, 4H, C<u>H</u>$_2$CH$_2$C<u>H</u>$_2$), 3.71 (d, *J* = 3.0 Hz, 2H, H-4), 3.64 – 3.45 (m, 8H, H-2, H-5, H-6), 3.45 – 3.39 (m, 2H, H-3), 2.16 (p, *J* = 6.4 Hz, 2H, CH$_2$C<u>H</u>$_2$CH$_2$).

^{13}C NMR (126 MHz, DMSO-d_6) δ 163.82 (2C, C=O), 159.26 (2C, ArC), 158.77 (2C, ArC), 139.97 (2C, <u>C</u>H=CHCONH), 137.51 (2C, ArCH), 131.32 (2C, ArCH), 130.35 (2C, ArC), 129.24 (4C, ArCH), 128.26 (2C, ArC), 119.65 (2C, CH=<u>C</u>HCONH), 116.62 (2C, ArCH),

110.35 (2C, ArCH), 100.67 (2C, C-1), 75.60 (2C, C-5), 73.27 (2C, C-3), 70.22 (2C, C-2), 68.13 (2C, C-4), 62.54 (2C, $\underline{C}H_2CH_2\underline{C}H_2$), 60.36 (2C, C-6), 28.38 (1C, $CH_2\underline{C}H_2CH_2$).

HPLC-MS: $[C_{43}H_{48}N_4O_{16} + H]^+$ calcd. 877.31, found 877.32.

HRMS: $[C_{43}H_{48}N_4O_{16} + H]^+$ calcd. 877.3138, found 877.3138.

Monovalent ligand A2

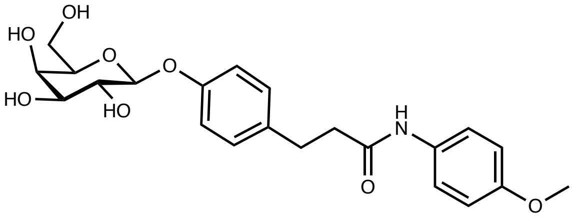

4-methoxyaniline **A** (12.1 mg, 98.1 µmol), compound **2** (36.4 mg, 111 µmol) and HBTU (47.1 mg, 124 µmol) were dissolved in dimethylformamide (1.5 mL) and DIPEA (20 µL, 115 µmol) was added. The reaction was stirred at r.t. for 3 h, then dried *in vacuo*. The product was purified by preparative reverse-phase HPLC (water/acetonitrile supplemented with 0.1% formic acid, gradient of 15-40% acetonitrile) and compound **A2** (36.7 mg, 84.7 µmol, 86%) was obtained as a white solid.

^{1}H NMR (500 MHz, DMSO-d_6) δ 9.73 (s, 1H, NH), 7.50 – 7.44 (m, 2H, ArH), 7.18 – 7.11 (m, 2H, ArH), 6.96 – 6.91 (m, 2H, ArH), 6.88 – 6.82 (m, 2H, ArH), 4.75 (d, J = 7.7 Hz, 1H, H-1), 3.71 (s, 3H, CH_3), 3.68 (d, J = 3.3 Hz, 1H, H-4), 3.57 – 3.43 (m, 4H, H-2, H-5, H-6), 3.38 (dd, J = 9.5, 3.3 Hz, 2H, H-3), 2.83 (t, J = 7.7 Hz, 2H, $C\underline{H}_2CH_2CONH$), 2.54 (dd, J = 8.6, 6.8 Hz, 2H, $CH_2C\underline{H}_2CONH$).

^{13}C NMR (126 MHz, DMSO-d_6) δ 169.91 (1C, C=O), 155.84 (1C, ArC), 155.04 (1C, ArC), 134.39 (1C, ArC), 132.42 (1C, ArC), 129.05 (2C, ArCH), 120.62 (2C, ArCH), 116.18 (2C, ArCH), 113.80 (2C, ArCH), 101.14 (1C, C-1), 75.44 (1C, C-5), 73.31 (1C, C-3), 70.30 (1C, C-2), 68.15 (1C, C-4), 60.40 (1C, C-6), 55.14 (1C, CH_3), 38.14 (1C, $CH_2\underline{C}H_2CONH$), 30.15 (1C, $\underline{C}H_2CH_2CONH$).

HPLC-MS: $[C_{22}H_{27}NO_8 + H]^+$ calcd. 434.18, found 434.08.

HRMS: $[C_{22}H_{27}NO_8 + H]^+$ calcd. 434.1809, found 434.1809.

Divalent ligand B2

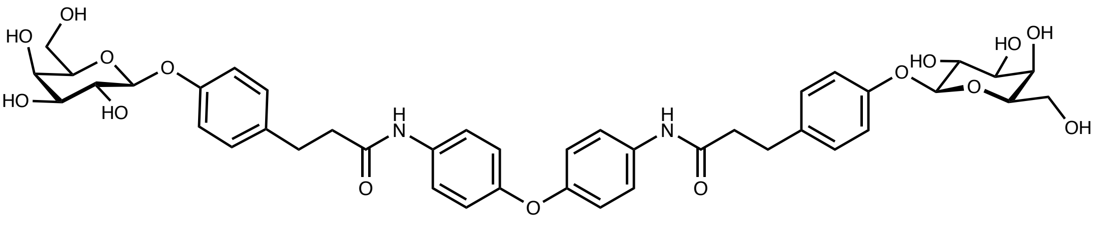

4,4’-Oxydianiline **B** (10.2 mg, 50.9 µmol), compound **2** (40.1 mg, 122 µmol) and HBTU (50.9 mg, 134 µmol) were dissolved in dimethylformamide (1 mL) and DIPEA (25 µL, 144 µmol) was added. The reaction was stirred at r.t. for 3 h, then dried *in vacuo*. The product was purified by preparative reverse-phase HPLC (water/acetonitrile with 0.1% formic acid, gradient of 15-40% acetonitrile) and compound **B2** (10.2 mg, 12.4 µmol, 24%) was obtained as a white solid.
^{1}H NMR (500 MHz, DMSO-d_6) δ 9.91 (s, 2H, NH), 7.58 – 7.52 (m, 4H, ArH), 7.17 – 7.12 (m, 4H, ArH), 6.96 – 6.88 (m, 8H, ArH), 4.76 (d, J = 7.7 Hz, 2H, H-1), 3.68 (d, J = 3.3 Hz, 2H, H-4), 3.56 – 3.44 (m, 8H, H-2, H-5, H-6), 3.38 (dd, J = 9.5, 3.3 Hz, 2H, H-3), 2.84 (t, J = 7.6 Hz, 4H, $C\underline{H}_2CH_2CONH$), 2.56 (t, J = 7.7 Hz, 4H, $CH_2C\underline{H}_2CONH$).
^{13}C NMR (126 MHz, DMSO-d_6) δ 170.23 (2C, C=O), 155.88 (2C, ArC), 152.38 (2C, ArC), 134.80 (2C, ArC), 134.34 (2C, ArC), 129.11 (4C, ArCH), 120.71 (4C, ArCH), 118.70 (4C, ArCH), 116.19 (4C, ArCH), 101.11 (2C, C-1), 75.47 (2C, C-5), 73.32 (2C, C-3), 70.31 (2C, C-2), 68.18 (2C, C-4), 60.42 (2C, C-6), 38.21 (2C, $CH_2\underline{C}H_2CONH$), 30.12 (2C, $\underline{C}H_2CH_2CONH$).
HPLC-MS: $[C_{42}H_{48}N_2O_{15} + H]^+$ calcd. 821.31, found 821.30.
HRMS: $[C_{42}H_{48}N_2O_{15} - H]^-$ calcd. 819.2982, found 819.2981.

Divalent ligand C2

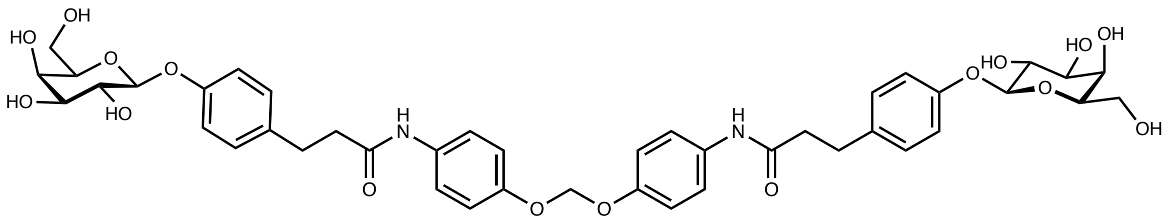

Bis(4-aminophenoxy)methane **C** (11.6 mg, 50.4 µmol), compound **2** (41.5 mg, 126 µmol) and HBTU (48.7 mg, 128 µmol) were dissolved in dimethylformamide (1 mL) and DIPEA (25 µL, 144 µmol) was added. The reaction was stirred at r.t. for 3 h, then dried *in vacuo*. The product was purified by preparative reverse-phase HPLC (water/acetonitrile with 0.1% formic acid, 15-40% acetonitrile) and compound **C2** (39.3 mg, 46.2 µmol, 92%) was obtained as a white solid.
^{1}H NMR (500 MHz, DMSO-d_6) δ 9.83 (s, 2H, NH), 7.54 – 7.47 (m, 4H, ArH), 7.17 – 7.11 (m, 4H, ArH), 7.04 – 6.96 (m, 4H, ArH), 6.96 – 6.89 (m, 4H, ArH), 5.72 (s, 2H, OCH_2O), 4.76 (d, J = 7.7 Hz, 2H, H-1), 3.68 (d, J = 3.3 Hz, 2H, H-4), 3.57 – 3.43 (m, 8H, H-2, H-5, H-6), 3.38 (dd, J = 9.5, 3.3 Hz, 2H, H-3), 2.83 (t, J = 7.7 Hz, 4H, $C\underline{H}_2CH_2CONH$), 2.58 – 2.52 (m, 4H, $CH_2C\underline{H}_2CONH$).
^{13}C NMR (126 MHz, DMSO-d_6) δ 170.11 (2C, C=O), 155.87 (2C, ArC), 152.03 (2C, ArC), 134.38 (2C, ArC), 133.97 (2C, ArC), 129.10 (4C, ArCH), 120.53 (4C, ArCH), 116.48 (4C, ArCH), 116.19 (4C, ArCH), 101.12 (2C, C-1), 90.78 (1C, OCH_2O), 75.47 (2C, C-5), 73.32 (2C,

C-3), 70.31 (2C, C-2), 68.18 (2C, C-4), 60.42 (2C, C-6), 38.20 (2C, $CH_2\underline{C}H_2CONH$), 30.14 (2C, $\underline{C}H_2CH_2CONH$).

HPLC-MS: $[C_{43}H_{50}N_2O_{16} + H]^+$ calcd. 851.32, found 851.32.

HRMS: $[C_{43}H_{50}N_2O_{16} - H]^-$ calcd. 849.3088, found 849.3085.

Divalent ligand D2

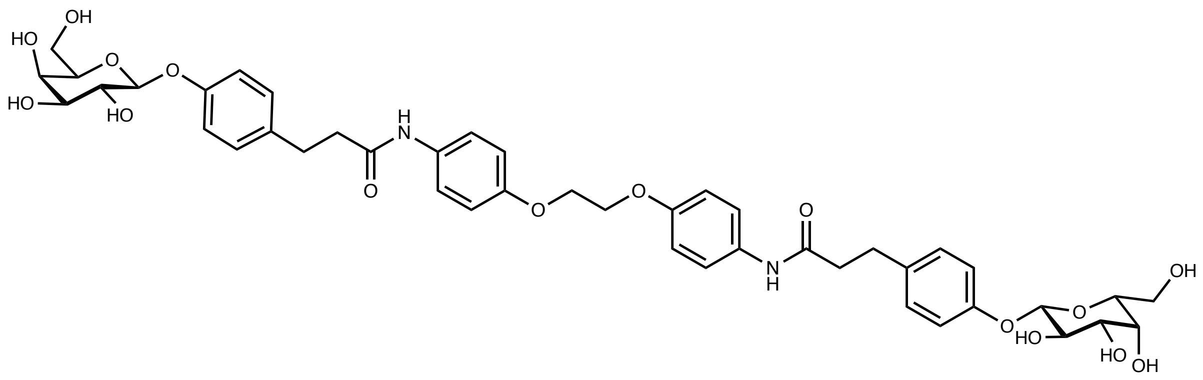

1,2-bis(4-aminophenoxy)ethane **D** (14.6 mg, 59.8 µmol), compound **2** (47.2 mg, 144 µmol) and HBTU (34.9 mg, 92.0 µmol) were dissolved in dimethylformamide (1 mL) and DIPEA (16 µL, 91.9 µmol) was added. The reaction was stirred at r.t. for 2 days, then dried *in vacuo*. The product was purified by preparative reverse-phase HPLC (water/acetonitrile with 0.1% formic acid, gradient of 25-45% acetonitrile) and compound **D2** (6.1 mg, 7.1 µmol, 12%) was obtained as a white solid.

^{1}H NMR (500 MHz, DMSO-d_6) δ 9.76 (s, 2H, NH), 7.51 – 7.45 (m, 4H, ArH), 7.18 – 7.11 (m, 4H, ArH), 6.96 – 6.86 (m, 8H, ArH), 4.76 (d, J = 7.7 Hz, 2H, H-1), 4.24 (s, 4H, CH_2), 3.68 (d, J = 3.3 Hz, 2H, H-4), 3.56 – 3.44 (m, 8H, H-2, H-5, H-6), 3.38 (dd, J = 9.5, 3.3 Hz, 2H, H-3), 2.84 (t, J = 7.6 Hz, 4H, $C\underline{H}_2CH_2CONH$), 2.58 – 2.52 (m, 4H, $CH_2C\underline{H}_2CONH$).

^{13}C NMR (126 MHz, DMSO-d_6) δ 169.94 (2C, C=O), 155.84 (2C, ArC), 154.08 (2C, ArC), 134.39 (2C, ArC), 132.67 (2C, ArC), 129.06 (4C, ArCH), 120.59 (4C, ArCH), 116.19 (4C, ArCH), 114.50 (4C, ArCH), 101.14 (2C, C-1), 75.45 (2C, C-5), 73.31 (2C, C-3), 70.30 (2C, C-2), 68.16 (2C, C-4), 66.48 (2C, CH_2), 60.40 (2C, C-6), 38.16 (2C, $CH_2\underline{C}H_2CONH$), 30.14 (2C, $\underline{C}H_2CH_2CONH$).

HPLC-MS: $[C_{44}H_{52}N_2O_{16} + H]^+$ calcd. 865.34, found 865.37.

HRMS: $[C_{44}H_{52}N_2O_{16} + H]^+$ calcd. 865.3390, found 865.3389.

Divalent ligand E2

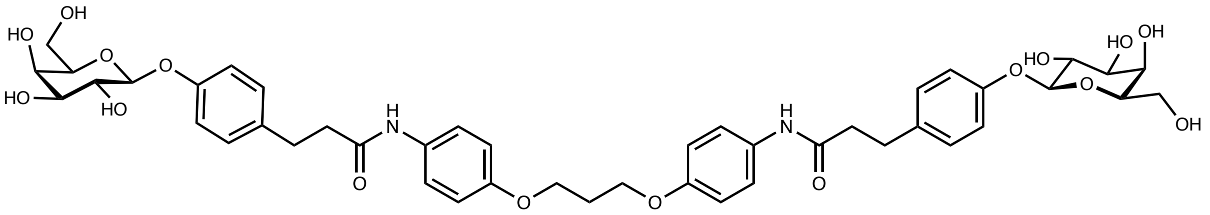

1,3-bis(4-aminophenoxy)propane **E** (11.7 mg, 45.3 μmol), compound **2** (34.3 mg, 104 μmol) and HBTU (46.1 mg, 122 μmol) were dissolved in dimethylformamide (1 mL) and DIPEA (40 μL, 227 μmol) was added. The reaction was stirred at r.t. for 24 h, then dried *in vacuo*. The product was purified by preparative reverse-phase HPLC (water/acetonitrile with 0.1% formic acid, gradient of 20-45% acetonitrile) and compound **E2** (16.6 mg, 18.9 μmol, 42%) was obtained as a white solid.

^{1}H NMR (500 MHz, DMSO-d_6) δ 9.74 (s, 2H, NH), 7.49 – 7.44 (m, 4H, ArH), 7.17 – 7.12 (m, 4H, ArH), 6.96 – 6.91 (m, 4H, ArH), 6.90 – 6.85 (m, 4H, ArH), 5.11 (d, *J* = 5.1 Hz, 2H, OH-2), 4.83 (d, *J* = 5.6 Hz, 2H, OH-3), 4.75 (d, *J* = 7.7 Hz, 2H, H-1), 4.63 (t, *J* = 5.3 Hz, 2H, OH-6), 4.47 (d, *J* = 4.6 Hz, 2H, OH-4), 4.07 (t, *J* = 6.3 Hz, 4H, $C\underline{H}_2CH_2C\underline{H}_2$), 3.68 (t, *J* = 4.1 Hz, 2H, H-4), 3.57 – 3.43 (m, 8H, H-2, H-5, H-6), 3.41 – 3.36 (m, 2H, H-3), 2.83 (t, *J* = 7.7 Hz, 4H, $C\underline{H}_2CH_2CONH$), 2.56 – 2.51 (m, 4H, $CH_2C\underline{H}_2CONH$), 2.16 – 2.08 (m, 2H, $CH_2C\underline{H}_2CH_2$).
^{13}C NMR (126 MHz, DMSO-d_6) δ 169.91 (2C, C=O), 155.83 (2C, ArC), 154.25 (2C, ArC), 134.39 (2C, ArC), 132.52 (2C, ArC), 129.05 (4C, ArCH), 120.59 (4C, ArCH), 116.18 (4C, ArCH), 114.45 (4C, ArCH), 101.14 (2C, C-1), 75.44 (2C, C-5), 73.31 (2C, C-3), 70.29 (2C, C-2), 68.15 (2C, C-4), 64.34 (2C, $\underline{C}H_2CH_2\underline{C}H_2$), 60.39 (2C, C-6), 38.15 (2C, $CH_2\underline{C}H_2CONH$), 30.14 (2C, $\underline{C}H_2CH_2CONH$), 28.74 (1C, $CH_2\underline{C}H_2CH_2$).
HPLC-MS: $[C_{45}H_{54}N_2O_{16} + H]^+$ calcd. 879.35, found 879.39.
HRMS: $[C_{45}H_{54}N_2O_{16} + H]^+$ calcd. 879.3546, found 879.3538.

Divalent ligand F2

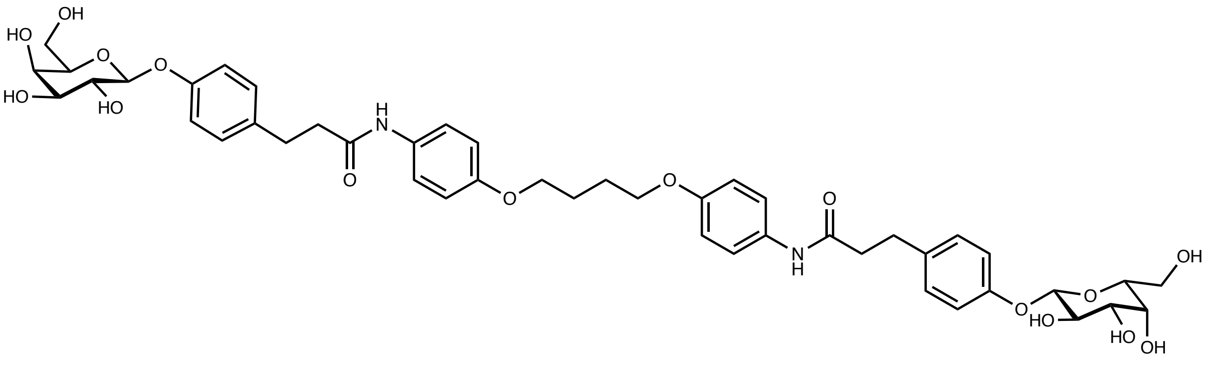

1,4-bis(4-aminophenoxy)butane **F** (36.5 mg, 134 µmol), compound **2** (104 mg, 317 µmol) and HBTU (142 mg, 375 µmol) were dissolved in dimethylformamide (3 mL) and DIPEA (60 µL, 344 µmol) was added. The reaction was stirred at r.t. for 2 h, then dried *in vacuo*. The product was purified by reverse-phase MPLC (water/acetonitrile with 0.1% formic acid, gradient of 20-35% acetonitrile) and compound **F2** (13.7 mg, 15.3 µmol, 11%) was obtained as a white solid.

^{1}H NMR (500 MHz, DMSO-d_6) δ 9.77 (s, 2H, NH), 7.49 – 7.43 (m, 4H, ArH), 7.18 – 7.11 (m, 4H, ArH), 6.95 – 6.89 (m, 4H, ArH), 6.89 – 6.82 (m, 4H, ArH), 5.16 (d, *J* = 5.2 Hz, 2H, OH-2), 4.91 (d, *J* = 5.5 Hz, 2H, OH-3), 4.76 (d, *J* = 7.7 Hz, 2H, H-1), 4.68 (t, *J* = 5.3 Hz, 2H, OH-6), 4.54 (d, *J* = 4.6 Hz, 2H, OH-4), 3.97 (d, *J* = 5.4 Hz, 4H, $C\underline{H}_2CH_2CH_2C\underline{H}_2$), 3.68 (t, *J* = 4.0 Hz, 2H, H-4), 3.57 – 3.42 (m, 8H, H-2, H-5, H-6), 3.41 – 3.37 (m, 2H, H-3), 2.83 (t, *J* = 7.7 Hz, 4H, $C\underline{H}_2CH_2CONH$), 2.56 – 2.51 (m, 4H, $CH_2C\underline{H}_2CONH$), 1.88 – 1.78 (m, 4H, $CH_2C\underline{H}_2C\underline{H}_2CH_2$).

^{13}C NMR (126 MHz, DMSO-d_6) δ 169.93 (2C, C=O), 155.86 (2C, ArC), 154.41 (2C, ArC), 134.40 (2C, ArC), 132.43(2C, ArC), 129.10 (4C, ArCH), 120.60 (4C, ArCH), 116.18 (4C, ArCH), 114.43 (4C, ArCH), 101.12 (2C, C-1), 75.47 (2C, C-5), 73.33 (2C, C-3), 70.31 (2C, C-2), 68.15 (2C, C-4), 67.24 (2C, $\underline{C}H_2CH_2CH_2\underline{C}H_2$), 60.40 (2C, C-6), 38.20 (2C, $CH_2\underline{C}H_2CONH$), 30.19 (2C, $\underline{C}H_2CH_2CONH$), 25.52 (2C, $CH_2\underline{C}H_2CH_2\underline{C}H_2$).

HPLC-MS: $[C_{46}H_{56}N_2O_{16} + H]^+$ calcd. 893.37, found 893.43.

HRMS: $[C_{46}H_{56}N_2O_{16} + H]^+$ calcd. 893.3736, found 893.3691.

Monovalent Ligand G2

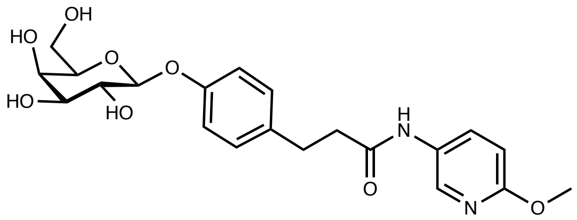

5-amino-2-methoxypyridine **G** (7.4 mg, 59.6 µmol), compound **2** (15.0 mg, 45.7 µmol) and HBTU (23.0 mg, 60.6 µmol) were dissolved in dimethylformamide (1.5 mL) and DIPEA (20 µL, 118 µmol) was added. The reaction was stirred at r.t. overnight, then lyophilized. The product was purified by preparative reverse-phase HPLC (water/acetonitrile supplemented with 0.1% formic acid, gradient of 15-30% acetonitrile) and compound **G2** (15.0 mg, 34.5 µmol, 76%) was obtained as a white solid.

^{1}H NMR (500 MHz, DMSO-d_6) δ 9.94 (s, 1H, NH), 8.31 (dd, *J* = 2.7, 0.6 Hz, 1H, ArH), 7.87 (dd, *J* = 8.9, 2.7 Hz, 1H, ArH), 7.17 – 7.12 (m, 2H, ArH), 6.96 – 6.91 (m, 2H, ArH), 6.78 (d, *J* = 8.8 Hz, 1H, ArH), 5.15 (d, *J* = 5.2 Hz, 1H, OH-2), 4.88 (d, *J* = 5.6 Hz, 1H, OH-3), 4.76 (d, *J*

= 7.7 Hz, 1H, H-1), 4.65 (t, J = 5.3 Hz, 1H, OH-6), 4.51 (d, J = 4.6 Hz, 1H, OH-4), 3.80 (s, 3H, CH_3), 3.68 (t, J = 4.1 Hz, 1H, H-4), 3.57 – 3.43 (m, 4H, H-2, H-5, H-6), 3.41 – 3.37 (m, 1H, H-3), 2.84 (t, J = 7.7 Hz, 2H, $C\underline{H}_2CH_2CONH$), 2.60 – 2.53 (m, 2H, $CH_2C\underline{H}_2CONH$).

^{13}C NMR (126 MHz, DMSO-d_6) δ 170.49 (1C, C=O), 159.50 (1C, ArC), 155.89 (1C, ArC), 137.47 (1C, ArCH), 134.26 (1C, ArC), 131.42 (1C, ArCH), 130.17 (1C, ArC), 129.12 (2C, ArCH), 116.20 (2C, ArCH), 110.13 (1C, ArCH), 101.10 (1C, C-1), 75.47 (1C, C-5), 73.33 (1C, C-3), 70.31 (1C, C-2), 68.17 (1C, C-4), 60.41 (1C, C-6), 53.17 (1C, CH_3), 38.01 (1C, $CH_2\underline{C}H_2CONH$), 30.07 (1C, $\underline{C}H_2CH_2CONH$).

HPLC-MS: $[C_{21}H_{26}N_2O_8 + H]^+$ calcd. 435.18, found 435.17.

HRMS: $[C_{21}H_{26}N_2O_8 + H]^+$ calcd. 435.1762, found 435.1756.

Divalent Ligand H2

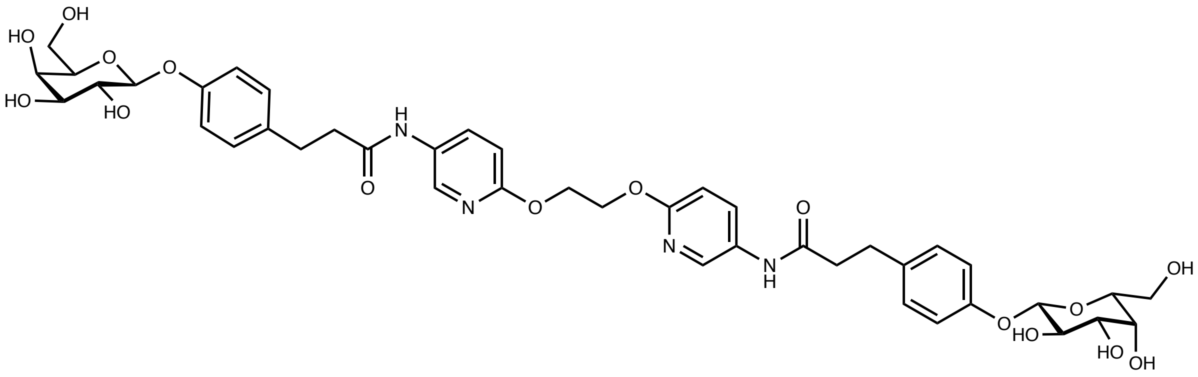

1,2-bis((5-aminopyridin-2-yl)oxy)ethane **H** (12.1 mg, 49.1 µmol), compound **2** (46.4 mg, 141 µmol) and HBTU (60.2 mg, 159 µmol) were dissolved in dimethylformamide (1.5 mL) and DIPEA (50 µL, 287 µmol) was added. The reaction was stirred at r.t. overnight, then lyophilized. The product was purified by preparative reverse-phase HPLC (water/acetonitrile supplemented with 0.1% formic acid, gradient of 15-30% acetonitrile) and compound **H2** (19.6 mg, 22.6 µmol, 46%) was obtained as a pale-yellow solid.

^{1}H NMR (500 MHz, DMSO-d_6) δ 9.93 (s, 2H, NH), 8.32 (d, J = 2.6 Hz, 2H, ArH), 7.88 (dd, J = 8.9, 2.7 Hz, 2H, ArH), 7.18 – 7.12 (m, 4H, ArH), 6.97 – 6.91 (m, 4H, ArH), 6.81 (d, J = 8.9 Hz, 2H, ArH), 4.76 (d, J = 7.7 Hz, 2H, H-1), 4.51 (s, 4H, CH_2), 3.68 (d, J = 3.3 Hz, 2H, H-4), 3.57 – 3.44 (m, 8H, H-2, H-5, H-6), 3.38 (dd, J = 9.5, 3.3 Hz, 2H, H-3), 2.85 (t, J = 7.7 Hz, 4H, $C\underline{H}_2CH_2CONH$), 2.57 (t, J = 7.7 Hz, 4H, $CH_2C\underline{H}_2CONH$).

^{13}C NMR (126 MHz, DMSO-d_6) δ 170.47 (2C, C=O), 158.88 (2C, ArC), 155.86 (2C, ArC), 137.33 (2C, ArCH), 134.24 (2C, ArC), 131.48 (2C, ArCH), 130.30 (2C, ArC), 129.06 (4C, ArCH), 116.20 (4C, ArCH), 110.35 (2C, ArCH), 101.12 (2C, C-1), 75.45 (2C, C-5), 73.31 (2C,

C-3), 70.30 (2C, C-2), 68.16 (2C, C-4), 64.05 (2C, CH_2), 60.40 (2C, C-6), 37.98 (2C, $CH_2\underline{C}H_2CONH$), 30.03 (2C, $\underline{C}H_2CH_2CONH$).

HPLC-MS: $[C_{42}H_{50}N_4O_{16} + H]^+$ calcd. 867.33, found 867.35.

HRMS: $[C_{42}H_{50}N_4O_{16} + H]^+$ calcd. 867.3295, found 867.3296.

Divalent Ligand I2

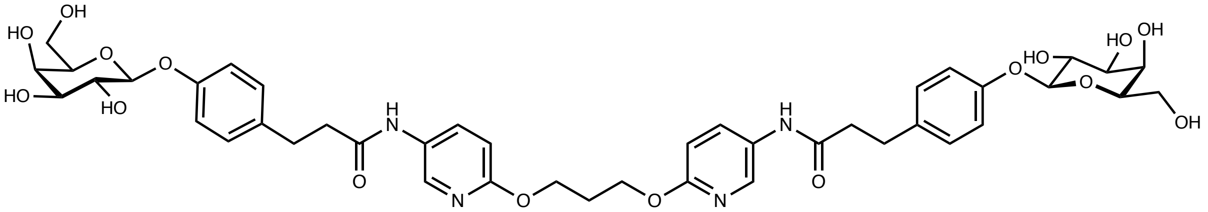

1,3-bis((5-aminopyridin-2-yl)oxy)propane **I** (14.7 mg, 56.5 μmol), compound **2** (40.7 mg, 124 μmol) and HBTU (49.8 mg, 131 μmol) were dissolved in dimethylformamide (1.5 mL) and DIPEA (50 μL, 287 μmol) was added. The reaction was stirred at r.t. overnight, then lyophilized. The product was purified by preparative reverse-phase HPLC (water/acetonitrile supplemented with 0.1% formic acid, gradient of 15-40% acetonitrile) and compound **I2** (20.2 mg, 22.9 μmol, 41%) was obtained as a white solid.

^{1}H NMR (500 MHz, DMSO-d_6) δ 9.91 (s, 2H, NH), 8.30 (d, J = 2.7 Hz, 2H, ArH), 7.86 (dd, J = 8.9, 2.7 Hz, 2H, ArH), 7.15 (d, J = 8.3 Hz, 4H, ArH), 6.99 – 6.89 (m, 4H, ArH), 6.78 (d, J = 8.9 Hz, 2H, ArH), 5.12 (d, J = 5.2 Hz, 2H, OH-2), 4.84 (d, J = 5.6 Hz, 2H, OH-3), 4.76 (d, J = 7.7 Hz, 2H, H-1), 4.63 (t, J = 5.2 Hz, 2H, OH-6), 4.48 (d, J = 4.6 Hz, 2H, OH-4), 4.33 (t, J = 6.3 Hz, 4H, $C\underline{H}_2CH_2C\underline{H}_2$), 3.68 (t, J = 4.0 Hz, 2H, H-4), 3.60 – 3.43 (m, 8H, H-2, H-5, H-6), 3.41 – 3.37 (m, 2H, H-3), 2.84 (t, J = 7.6 Hz, 4H, $C\underline{H}_2CH_2CONH$), 2.57 (t, J = 7.7 Hz, 4H, $CH_2C\underline{H}_2CONH$), 2.12 (p, J = 6.3 Hz, 2H, $CH_2C\underline{H}_2CH_2$).

^{13}C NMR (126 MHz, DMSO-d_6) δ 170.45 (2C, C=O), 159.14 (2C, ArC), 155.87 (2C, ArC), 137.48 (2C, ArCH), 134.25 (2C, ArC), 131.40 (2C, ArCH), 130.12 (2C, ArC), 129.07 (4C, ArCH), 116.20 (4C, ArCH), 110.23 (2C, ArCH), 101.13 (2C, C-1), 75.45 (2C, C-5), 73.32 (2C, C-3), 70.30 (2C, C-2), 68.15 (2C, C-4), 62.49 (2C, $\underline{C}H_2CH_2\underline{C}H_2$), 60.40 (2C, C-6), 37.97 (2C, $CH_2\underline{C}H_2CONH$), 30.05 (2C, $\underline{C}H_2CH_2CONH$), 28.35 (1C, $CH_2\underline{C}H_2CH_2$).

HPLC-MS: $[C_{43}H_{52}N_4O_{16} + H]^+$ calcd. 881.35, found 881.33.

HRMS: $[C_{43}H_{52}N_4O_{16} + H]^+$ calcd. 881.3451, found 881.3446.

Divalent Ligand J2

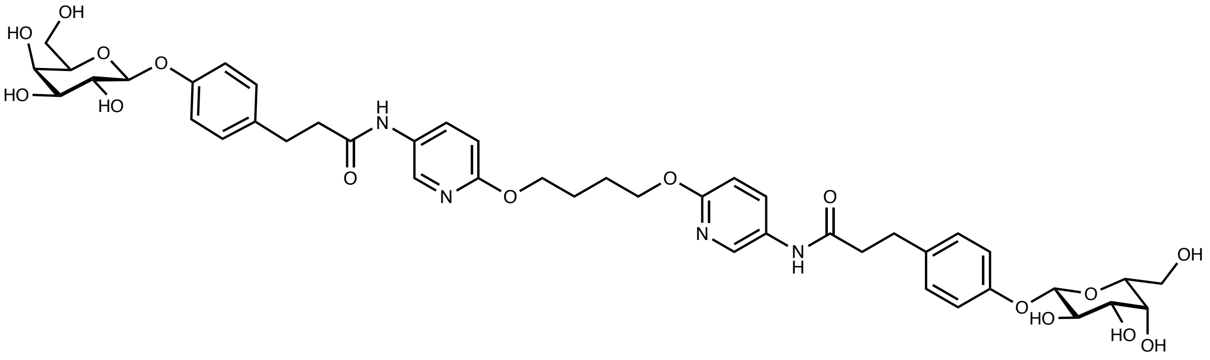

1,4-bis((5-aminopyridin-2-yl)oxy)butane **J** (10.5 mg, 38.3 µmol), compound **2** (46.5 mg, 142 µmol) and HBTU (45.5 mg, 120 µmol) were dissolved in dimethylformamide (1.5 mL) and DIPEA (40 µL, 230 µmol) was added. The reaction was stirred at r.t. overnight, then lyophilized. The product was purified by preparative reverse-phase HPLC (water/acetonitrile supplemented with 0.1% formic acid, gradient of 15-45% acetonitrile) and compound **J2** (16.2 mg, 18.1 µmol, 47%) was obtained as a white solid.

^{1}H NMR (500 MHz, DMSO-d_6) δ 9.90 (s, 2H, NH), 8.29 (d, J = 2.7 Hz, 2H, ArH), 7.86 (dd, J = 8.9, 2.7 Hz, 2H, ArH), 7.17 – 7.12 (m, 4H, ArH), 6.96 – 6.91 (m, 4H, ArH), 6.76 (d, J = 8.8 Hz, 2H, ArH), 5.11 (d, J = 5.1 Hz, 2H, OH-2), 4.83 (d, J = 5.6 Hz, 2H, OH-3), 4.76 (d, J = 7.7 Hz, 2H, H-1), 4.63 (t, J = 5.3 Hz, 2H, OH-6), 4.47 (d, J = 4.6 Hz, 2H, OH-4), 4.24 (q, J = 4.4, 3.0 Hz, 4H, $C\underline{H}_2CH_2CH_2C\underline{H}_2$), 3.68 (t, J = 4.1 Hz, 2H, H-4), 3.57 – 3.43 (m, 8H, H-2, H-5, H-6), 3.42 – 3.36 (m, 2H, H-3), 2.84 (t, J = 7.7 Hz, 4H, $C\underline{H}_2CH_2CONH$), 2.60 – 2.54 (m, 4H, $CH_2C\underline{H}_2CONH$), 1.84 – 1.77 (m, 4H, $CH_2C\underline{H}_2C\underline{H}_2CH_2$).

^{13}C NMR (126 MHz, DMSO-d_6) δ 170.43 (2C, C=O), 159.28 (2C, ArC), 155.86 (2C, ArC), 137.51 (2C, ArCH), 134.25 (2C, ArC), 131.39 (2C, ArCH), 130.01 (2C, ArC), 129.06 (4C, ArCH), 116.19 (4C, ArCH), 110.17 (2C, ArCH), 101.12 (2C, C-1), 75.45 (2C, C-5), 73.32 (2C, C-3), 70.30 (2C, C-2), 68.15 (2C, C-4), 65.14 (2C, $\underline{C}H_2CH_2CH_2\underline{C}H_2$), 60.40 (2C, C-6), 37.97 (2C, $CH_2\underline{C}H_2CONH$), 30.04 (2C, $\underline{C}H_2CH_2CONH$), 25.38 (2C, $CH_2\underline{C}H_2\underline{C}H_2CH_2$).

HPLC-MS: $[C_{44}H_{54}N_4O_{16} + H]^+$ calcd. 895.36, found 895.36.

HRMS: $[C_{44}H_{54}N_4O_{16} + H]^+$ calcd. 895.3608, found 895.3601.

Monovalent ligand K2

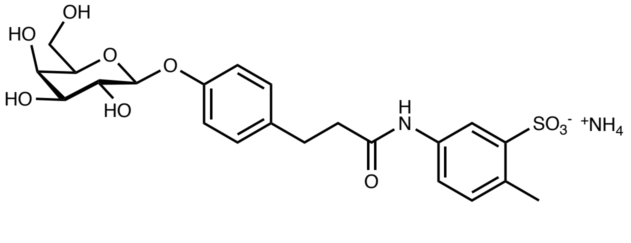

5-Amino-2-methylbenzenesulfonic acid **K** (11.0 mg, 58.8 µmol), galactoside **2** (15.0 mg, 45.7 µmol) and PyBOP (31.0 mg, 59.6 µmol) were dissolved in dimethylformamide (1.5 mL) and N-methylmorpholine (20 µL, 178 µmol) was added. The reaction was stirred at r.t. overnight, then lyophilized. The product was purified by preparative reverse-phase HPLC (5 mM ammonium bicarbonate at pH 7/acetonitrile, gradient of 5-20% acetonitrile) and compound **K2** (16.0 mg, 32.2 µmol, 70%) was obtained as a white solid.

^{1}H NMR (500 MHz, DMSO-d_6) δ 9.88 (s, 1H, NH), 7.78 (d, *J* = 2.4 Hz, 1H, ArH), 7.63 (dd, *J* = 8.2, 2.4 Hz, 1H, ArH), 7.14 (d, *J* = 8.6 Hz, 2H, ArH), 7.09 (s, 3H, ammonia), 7.02 (d, *J* = 8.2 Hz, 1H, ArH), 6.95 – 6.91 (m, 2H, ArH), 5.13 (d, *J* = 5.2 Hz, 1H, OH-2), 4.86 (d, *J* = 5.6 Hz, 1H, OH-3), 4.76 (d, *J* = 7.7 Hz, 1H, H-1), 4.66 (t, *J* = 5.2 Hz, 1H. OH-6), 4.49 (d, *J* = 4.7 Hz, 1H, OH-4), 3.67 (t, *J* = 4.0 Hz, 1H, H-4), 3.57 – 3.43 (m, 4H, H-2, H-5, H-6), 3.40 – 3.37 (m, 1H, H-3), 2.83 (t, *J* = 7.7 Hz, 2H, C$\underline{H}_2$CH$_2$CONH), 2.58 – 2.52 (m, 2H, CH$_2$C$\underline{H}_2$CONH), 2.43 (s, 3H, CH$_3$).

^{13}C NMR (126 MHz, DMSO) δ 170.28 (1C, C=O), 155.87 (1C, ArC), 146.50 (1C, ArC), 136.15 (1C, ArC), 134.41 (1C, ArC), 130.78 (1C, ArCH), 129.90 (1C, ArC), 129.10 (2C, ArCH), 119.11 (1C, ArCH), 117.80 (1C, ArCH), 116.21 (2C, ArCH), 101.15 (1C, C-1), 75.45 (1C, C-5), 73.31 (1C, C-3), 70.33 (1C, C-2), 68.19 (1C, C-4), 60.40 (1C, C-6), 38.29 (1C, CH$_2$$\underline{C}H_2$CONH), 30.20 (1C, $\underline{C}$H$_2$CH$_2$CONH), 19.53 (1C, CH$_3$).

HPLC-MS: [$C_{22}H_{30}N_2O_{10}S$ - H]$^-$ calcd. 496.13, found 496.12.

HRMS: [$C_{22}H_{30}N_2O_{10}S$ - H]$^-$ calcd. 496.1283, found 496.1289.

Divalent ligand L2

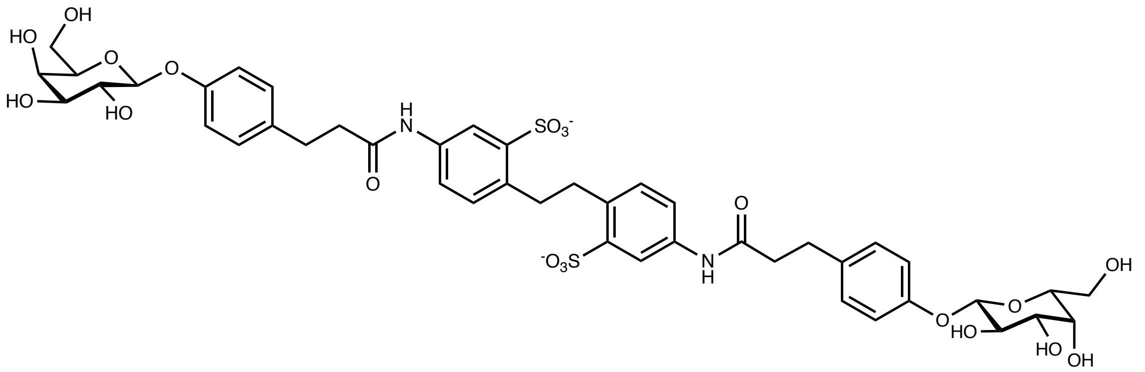

Sulfonated linker **L** (21.6 mg, 58.0 µmol), galactoside **2** (59.0 mg, 198 µmol) and PyBOP (74.3 mg, 143 µmol) were suspended in dimethylformamide (1.5 mL) and N-methylmorpholine (50 µL, 455 µmol) was added. The reaction was stirred at r.t. overnight, then lyophilized. The product was purified by preparative reverse-phase HPLC (5 mM ammonium bicarbonate at pH

7/acetonitrile, gradient of 5-20% acetonitrile) and compound **L2** (30.4 mg, 29.6 µmol, 51%) was obtained as a white solid.

^{1}H NMR (500 MHz, DMSO-*d*$_6$) δ 9.88 (s, 2H, NH), 7.85 (d, *J* = 2.3 Hz, 2H, ArH), 7.59 (dd, *J* = 8.2, 2.3 Hz, 2H, ArH), 7.19 (d, *J* = 8.3 Hz, 2H, ArH), 7.17 – 7.13 (m, 4H, ArH), 6.98 – 6.89 (m, 4H, ArH), 4.77 (d, *J* = 7.7 Hz, 2H, H-1), 3.67 (d, *J* = 3.3 Hz, 2H, H-4), 3.57 – 3.44 (m, 8H, H-2, H-5, H-6), 3.38 (dd, *J* = 9.8, 3.6 Hz, 2H, H-3), 3.18 (s, 4H, ArCH$_2$CH$_2$Ar), 2.84 (t, *J* = 7.7 Hz, 4H, C<u>H</u>$_2$CH$_2$CONH), 2.55 (t, *J* = 7.6 Hz, 4H, CH$_2$C<u>H</u>$_2$CONH).

^{13}C NMR (126 MHz, DMSO-*d*$_6$) δ 170.26 (2C, C=O), 155.88 (2C, ArC), 146.15 (2C, ArC), 135.89 (2C, ArC), 135.24 (2C, ArC), 134.48 (2C, ArC), 130.49 (2C, ArCH), 129.13 (4C, ArCH), 119.37 (2C, ArCH), 117.98 (2C, ArCH), 116.24 (4C, ArCH), 101.17 (2C, C-1), 75.46 (2C, C-5), 73.32 (2C, C-3), 70.36 (2C, C-2), 68.21 (2C, C-4), 60.41 (2C, C-6), 38.34 (2C, CH$_2$<u>C</u>H$_2$CONH), 33.77 (2C, ArCH$_2$CH$_2$Ar), 30.24 (2C, <u>C</u>H$_2$CH$_2$CONH).

HPLC-MS: $[C_{44}H_{52}N_2O_{20}S_2 - 2H]^{2-}$ calcd. 495.12, found 495.11.

HRMS: $[C_{44}H_{52}N_2O_{20}S_2 - H]^{-}$ calcd. 991.2482, found 991.2531.

Synthesis of compound **14** was described by Zahorska *et. al.*.[8]

Biophysical evaluation

Expression and purification of LecA as well as competitive binding by fluorescence polarization was performed as described by Joachim *et al.*.[9] The assay was performed in TBS/Ca^{2+} buffer (20 mM Tris, 137 mM NaCl, 2.6 mM KCl at pH 7.4 supplemented with 1 mM $CaCl_2$) in presence of 25% DMSO. Averages and standard deviations were calculated from at least three independent experiments.

Isothermal titration calorimetry was performed on an iTC200 (Malvern Panalytical) and the data were analyzed using Microcal Origin software (Malvern Panalytical). LecA (50 - 180 µM) in the cell was titrated with ligand (0.25 - 1.5 mM) at 25 °C in TBS/Ca^{2+} buffer for **G2**, **K2** and **L2** and TBS/Ca^{2+} buffer supplemented with 5% DMSO for **I2** and **J2**. Steep titration slopes achieved with the divalent ligands were the result of high amount of protein present and the high binding affinity of the ligand (high 'value of c').[10] However, LecA protein concentration lower than 50 µM resulted in poor signal to noise ratio as a consequence of the low heat released upon binding.

Surface plasmon resonance experiments were performed on a BIACORE X100 instrument (GE Healthcare) at 25 °C as described by Zahorska *et. al.*.[8] Averages and standard deviations were calculated from three independent experiments.

Kinetic solubility was determined on the analytical HPLC-MS using Compass QuantAnalysis quantification software (Bruker). Samples were prepared from DMSO stock solutions by dilution with TBS/Ca^{2+} buffer to 100 µM with 1% DMSO present. Samples were incubated on a shaker for 1 h at r.t., centrifuged (21380 g, 20 min), supernatants were diluted with DMSO (1:3), analyzed by HPLC-MS and fitted to the individual calibration curves.

ADME assays

Every experiment was repeated independently at least three times.

Plasma stability assay

Each compound dissolved in DMSO was added to mouse plasma (pH 7.4, 37 °C) or to human plasma (pH 7.4, 37 °C) to yield a final concentration of 1 µM. In addition, procaine and procainamide (dissolved in DMSO) were added to mouse plasma or to human plasma (pH 7.4, 37 °C) to yield a final concentration of 1 µM. Procaine served as positive control as it is unstable in mouse plasma. Procainamide served as negative control as it is stable in mouse plasma. The samples were incubated for 0 min, 15 min, 30 min, 60 min, 90 min, 120 min and 240 min at 37 °C. At each time point, 10 µl of the respective sample was extracted with 90 µL acetonitrile containing 12.5 ng/mL caffeine as internal standard for 5 min at 2000 rpm on a MixMate vortex

mixer (Eppendorf). Acetonitrile and caffeine were dispensed using a Mantis Formulatrix. Then samples were centrifuged for 20 min at 2.270 x *g* at 4 °C and the supernatants were transferred to 96-well Greiner V-bottom plates. Peak areas of each compound and of the internal standard were analyzed using the MultiQuant 3.0 software (AB Sciex). Peak areas of the respective compound were normalized to the internal standard peak area and to the respective peak areas at time point 0 min: (C/D)/(A/B) with A: peak area of the compound at time point 0 min, B: peak area of the internal standard at time point 0 min, C: peak area of the compound at the respective time point, D: peak area of the internal standard at the respective time point.

In vitro metabolic stability assay

Liver microsomes (mouse and human, Thermo Fisher) were thawed slowly on ice. 20 mg/mL of microsomes, 2 µL of a 100 µM solution of every compound and 183 µL of 100 mM phosphate buffer were incubated 5 min at 37 °C in a water bath. Reactions were initiated using 10 µL of 20 mM NADPH (CarlRoth). Samples were incubated in three replicates at 37 °C under gentle agitation at 150 rpm. At 0, 5, 15, 30, and 60 min, reactions were terminated by the addition of 180 µL acetonitrile using a Mantis Formulatrix dispenser. Samples were vortexed for 5 min using an Eppendorf MixMate vortex mixer and centrifuged at 2.270 x *g* for 20 min at 4 °C. The supernatants were transferred to 96-well Greiner V-bottom plates, sealed and analyzed according to the section HPLC-MS analysis. Peak areas of the respective time point of the compounds were normalized to the peak area at time point 0 min. Then half-life was calculated using linear regression (Microsoft Excel). Cl_{int} [µL/min/mg protein] was calculated using the following formula:

$Cl_{int} = 0.693/(0.005 \text{ x } t_{1/2})$

Assessment of plasma protein binding

Plasma protein binding was assessed using the rapid equilibrium device (RED) system from ThermoFisher. Compounds were dissolved in DMSO. Naproxene served as control as it shows high plasma protein binding. Compounds were diluted in murine plasma (from CD-1 mice, pooled) or in human plasma (human donors, both genders, pooled) to a final concentration of 1 µM. Dialysis buffer and plasma samples were added to the respective chambers according the manufacturer's protocol. The RED plate was sealed with a tape and incubated at 37 °C for 2 hours at 800 rpm on an Eppendorf MixMate vortex-mixer. Then samples were withdrawn from the respective chambers. To 25 µL of each dialysis sample, 25 µL of plasma and to 25 µL of plasma sample, 25 µL of dialysis buffer was added. Then 150 µL ice-cold extraction solvent (ACN/H_2O (90:10) containing 12.5 ng/mL caffeine as internal standard) was added. Samples were incubated for 30 min on ice. Then samples were centrifuged at 4 °C at 2270 x *g* for 10 min.

Supernatants were transferred to Greiner V-bottom 96-well plates and sealed with a tape. The percentage of bound compound was calculated as follows:

(1) % free = (concentration buffer chamber / concentration plasma chamber) *100

% bound = 100 % - % free

HPLC-MS analysis

Samples were analyzed using an Agilent 1290 Infinity II HPLC system coupled to an AB Sciex QTrap 6500plus mass spectrometer. LC conditions were as follows: column: Agilent Zorbax Eclipse Plus C18, 50x2.1 mm, 1.8 µm; temperature: 30 °C; injection volume: 5 µL per sample; flow rate: 700 µL/min. Samples were run under acidic and buffered conditions. Solvents for acidic conditions: A1: water + 0.1% formic acid; solvent B1: 95% acetonitrile/5% H_2O + 0.1% formic acid; solvents for buffered conditions: A2: 95% water + 5% acetonitrile + 5 mM ammonium acetate + 40 µL/L acetic acid; B2: 95% acetonitrile + 5% water + 5 mM ammonium acetate + 40 µL/L acetic acid. The same gradient was applied for acidic and buffered conditions: 99% A at 0 min, 99% A until 1 min, 99% - 0% A from 1 min to 4.0 min, 0% A until 5.0 min. Mass transitions for controls and compounds are depicted in the following table.

	Q1 mass	Q3 mass	DP [volts]	CE [volts]	CXP [volts]
Caffeine	195.024	138.0	130.0	25.0	14.0
Caffeine	195.024	110.0	130.0	31.0	18.0
**14**	861.223	699.1	-125.0	-24.0	-39.0
**14**	861.223	417.0	-125.0	-54.0	-19.0
**H2**	865.252	541.1	-145.0	-48.0	-25.0
**H2**	865.252	703.2	-145.0	-30.0	-35.0
**D2**	863.289	539.0	-145.0	-50.0	-23.0
**D2**	863.289	701.1	-145.0	-30.0	-35.0
**D1**	859.229	697.1	-115.0	-24.0	-37.0
**D1**	859.220	415.0	-115.0	-54.0	-19.0
**H1**	861.239	699.1	-160.0	-26.0	-35.0
**H1**	861.239	537.1	-160.0	-40.0	-27.0
**L2**	511.394	255.1	-35.0	-14.0	-23.0
**L2**	511.304	283.1	-35.0	-20.0	-17.0
**L2**	495.068	333.0	-55.0	-40.0	-35.0
**L2**	495.068	413.9	-55.0	-30.0	-17.0

Pharmacokinetic studies

Pharmacokinetic studies in mice

For pharmacokinetic experiments, outbred male CD-1 mice (Charles River, Germany), 4-weeks-old, were used. The animal studies were conducted in accordance with the recommendations of the European Community (Directive 86/609/EEC, 24 November 1986). All animal procedures were performed in strict accordance with the German regulations of the Society for Laboratory Animal Science (GV-SOLAS) and the European Health Law of the Federation of Laboratory Animal Science Associations (FELASA). Animals were excluded from further analysis if sacrifice was necessary according to the humane endpoints established by the ethical board. All experiments were approved by the ethical board of the Niedersächsisches Landesamt für Verbraucherschutz und Lebensmittelsicherheit, Oldenburg, Germany (33.19-42502-04-20/3522). **L2** and **H2** were dissolved in water and administered in individual PK studies (N = 2 per study) at 1 mg/kg intravenously. Up to 25 µl blood was collected from the lateral tail vein and time points t = 0.25, 0.5, 1 and 3 hours. At t = 5 hours animals were euthanized to collect blood. At time points t = 0.25, 0.5, 1, 3 and 5 hours spontaneous urine was collected as well. Whole blood was collected into Eppendorf tubes coated with 0.5 M EDTA and immediately spun down at 15870 x *g* for 10 min at 4 °C. Then, plasma was transferred into a new Eppendorf tube and stored at -80°C until analysis.

Pharmacokinetic studies in rat

The in vivo part of the study was performed by the CRO Pharmacelsus (Saarbrücken, Germany). For rat *in vivo* pharmacokinetic studies, male Sprague-Dawley rats (Janvier-Labs, France) with 350 g - 400 g body weight were used. All experimental procedures were approved by and conducted in accordance with the regulations of the local Animal Welfare authorities (Landesamt für Gesundheit und Verbraucherschutz, Abteilung Lebensmittel- und Veterinärwesen, Saarbrücken) and any clinical signs of toxicity and any changes in behavior of the animals were recorded. Food and water were provided *ad libitum* throughout the study. Rats were provided with a catheter in the jugular vein 2 - 3 days prior to drug administration and blood sampling. According to national animal welfare guidelines, rats were treated once with Carprofen (5 mg/kg) directly after catheterization. Catheter was rinsed daily with K_3EDTA. **L2** was dissolved in PBS (pH 7.4) and administered *i.v.* in individual PK studies (N = 3) at 10 mg/kg in a volume of 2 mL/kg. At t = 0 h, rats were placed into metabolic cages for drug administration and immediate collection of urine and faeces samples. Further excretion products were collected during 0 - 2 h, 2 - 4 h, 4 - 8 h and 8 - 24 h. Excreta sample volumes were determined and immediately stored at -80 °C until analysis by LC-MS. 80 µL K_3EDTA-treated blood was collected at t = 0.083 h, 0.25 h, 0.5 h, 1 h, 2 h, 4 h, 6 h, 8 h and 24 h post dose and samples were stored on ice until centrifugation (10 minutes at 4500 x g, 4 °C). Plasma was prepared within 45 min after collection, frozen and stored at -80 °C until analysis by LC-MS. Animals showed a normal behavior and there were no clinical signs observed after dosing.

Bioanalysis of pharmacokinetic samples from mice and from rat

First, a calibration curve was prepared by spiking different concentrations of **L2** and **H2** into mouse plasma, rat plasma or mouse urine (matrix for mouse and rat PK samples) from CD-1 mice. Glipizide was used as an internal standard. In addition, quality control samples (QCs) were prepared for **L2** and **H2** in plasma and urine. The following extraction procedures were used: 7.5 µl of a plasma sample or 15 µl of a urine sample (calibration samples, QCs or PK samples) was extracted with 35 µl of methanol containing 12.5 ng/ml of glipizide as internal standard for 15 min at 2000 rpm on an Eppendorf MixMate vortex mixer. Then samples were spun down at 15870 x *g* for 10 min. Supernatants were transferred to standard HPLC-glass vials. Samples from the mouse PK study with **H2** in mice and from the rat PK study with **L2** were analyzed via HPLC-MS/MS as described in the *HPLC-MS analysis* section above. PK samples from the mouse PK study with **L2** were analyzed using an Agilent 1290 Infinity II HPLC system coupled to an AB Sciex QTrap 7500 mass spectrometer. LC conditions were the same as for the analysis of the samples from the mouse PK study with **H2** and from the rat PK study with **L2**. Mass transitions for controls and compounds are depicted in the following table for the AB Sciex Trap 7500 mass spectrometer.

Peaks of PK samples were quantified using the calibration curve. The accuracy of the calibration curve was determined using QCs independently prepared on different days. PK parameters were determined using a non-compartmental analysis with PKSolver.[11]

	Q1 mass	Q3 mass	EP [volts]	CE [volts]	CXP [volts]
L2	1027.3	1010.273	10	18	22
L2	1027.3	521.062	10	62	46
L2	1027.3	669.109	10	43	13

	Compound	IC_{50} [μM]	Compound	IC_{50} [μM]
Controls	Me-α-Gal	89.8 ± 12.4	pNO2-Ph-β-Gal	39.8 ± 12.0
Structure	R = 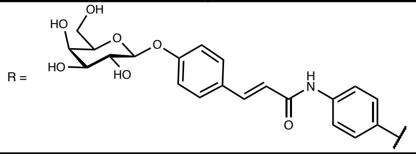		R = 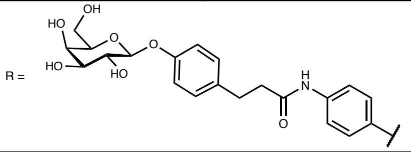	
	**Benzene series**			
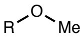	**A1**	18.8 ± 6.6	**A2**	18.9 ± 5.5
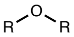	**B1**	6.6 ± 1.5	**B2**	3.6 ± 1.3
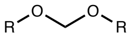	**C1**	4.7 ± 2.6	**C2**	4.2 ± 1.7
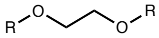	**D1**	5.6 ± 3.1	**D2**	4.5 ± 1.8
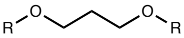	**E1**	4.5 ± 1.6	**E2**	3.5 ± 1.0
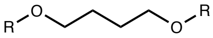	**F1**	7.9 ± 4.8	**F2**	4.4 ± 1.5
Structure	R = 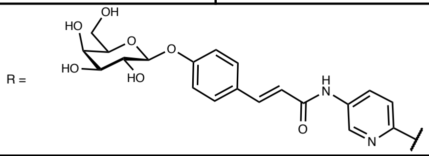		R = 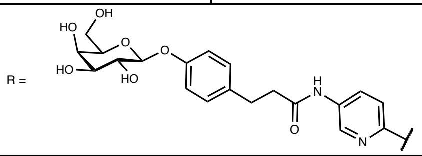	
	**Pyridine series**			
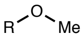	-	-	**G2**	14.3 ± 7.2
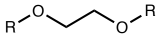	**H1**	8.0 ± 4.3	**H2**	4.8 ± 2.2
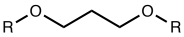	**I1**	6.6 ± 4.0	**I2**	3.2 ± 1.3
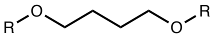	-	-	**J2**	3.4 ± 1.3
Structure	R = 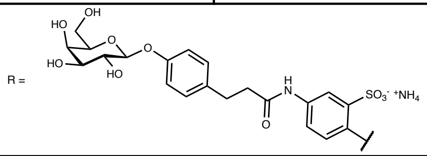			
	**Sulfonated series**			
R—Me	**K2**	14.4 ± 3.6		
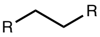	**L2**	4.6 ± 2.2		

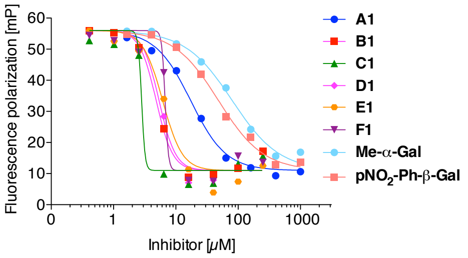

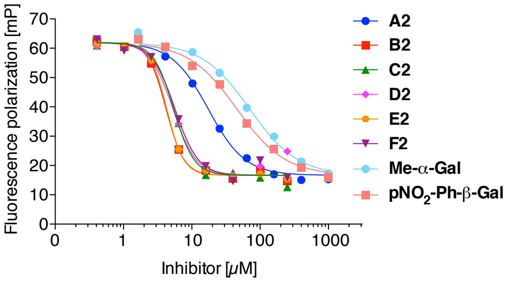

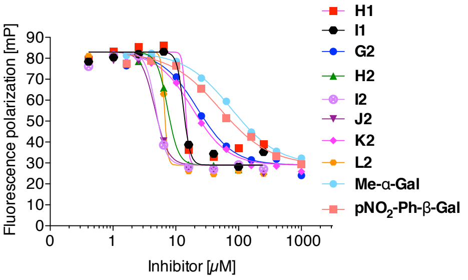

Figure S1: Evaluation of amide divalent LecA ligands in a competitive binding assay based on fluorescence polarization. Divalent ligands showed steep titration slopes indicating the lower assay limit was reached. One representative experiment is shown or each series. Averages and standard deviations from at least three independent titrations of triplicates each.

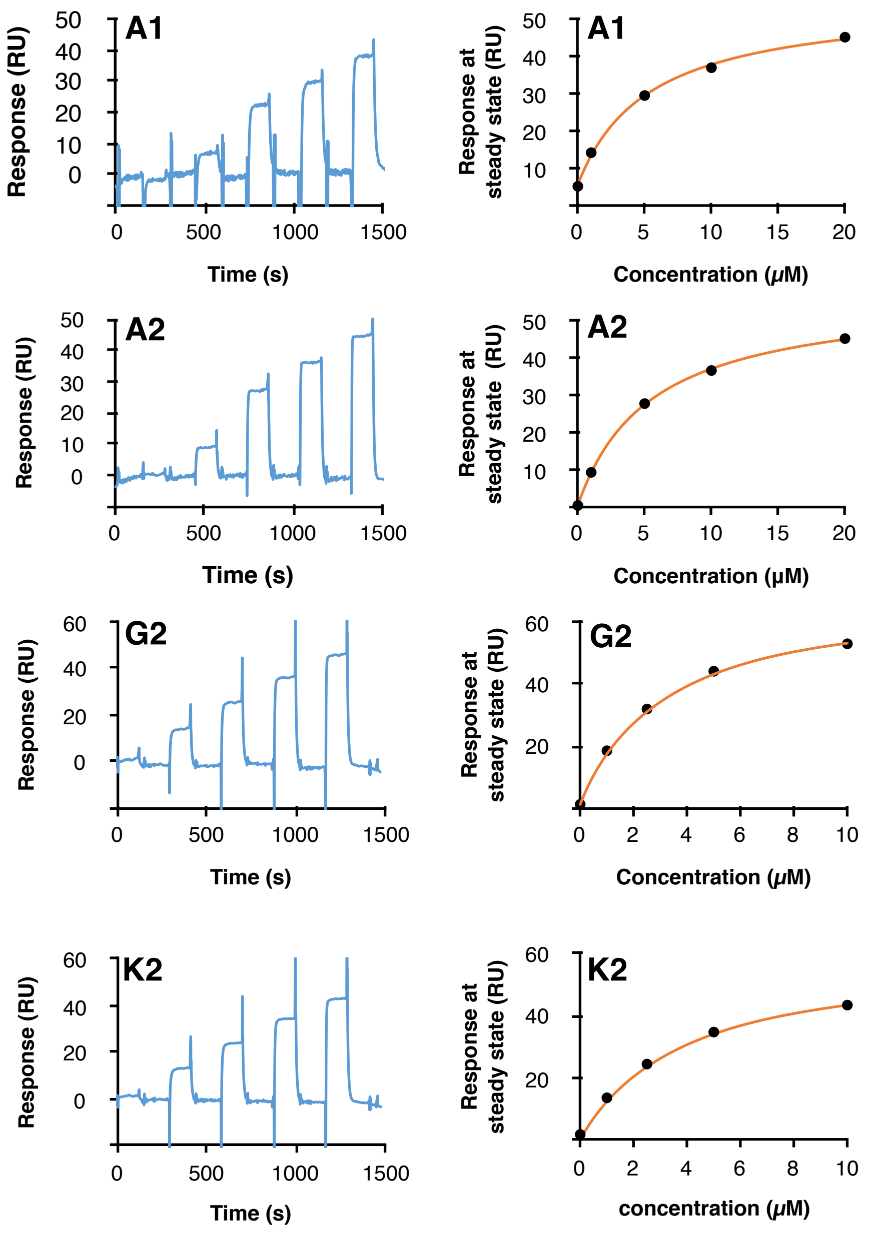

Figure S2: SPR sensograms (left panel) and analyses (right panel) of the monovalent LecA ligands.

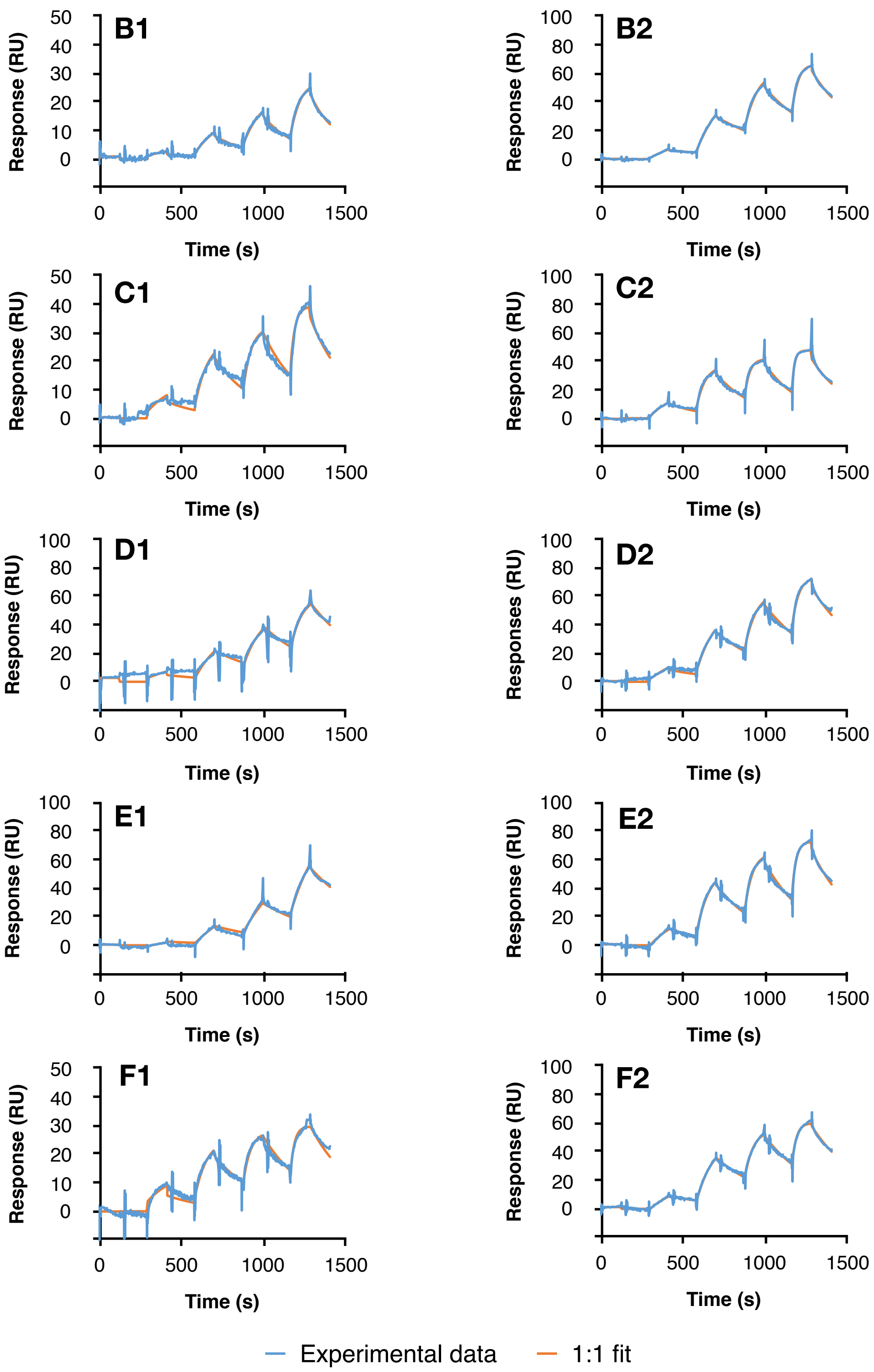

Figure S3: SPR of divalent LecA ligands from benzene series. Sensorgrams obtained from SPR single-cycle kinetics experiments. Five different concentrations of each compound (0, 10, 50, 100, 200 nM) were sequentially injected to obtain the experimental sensorgrams (blue lines), which were then fitted by a 1:1 model (orange) on BIACORE evaluation software.

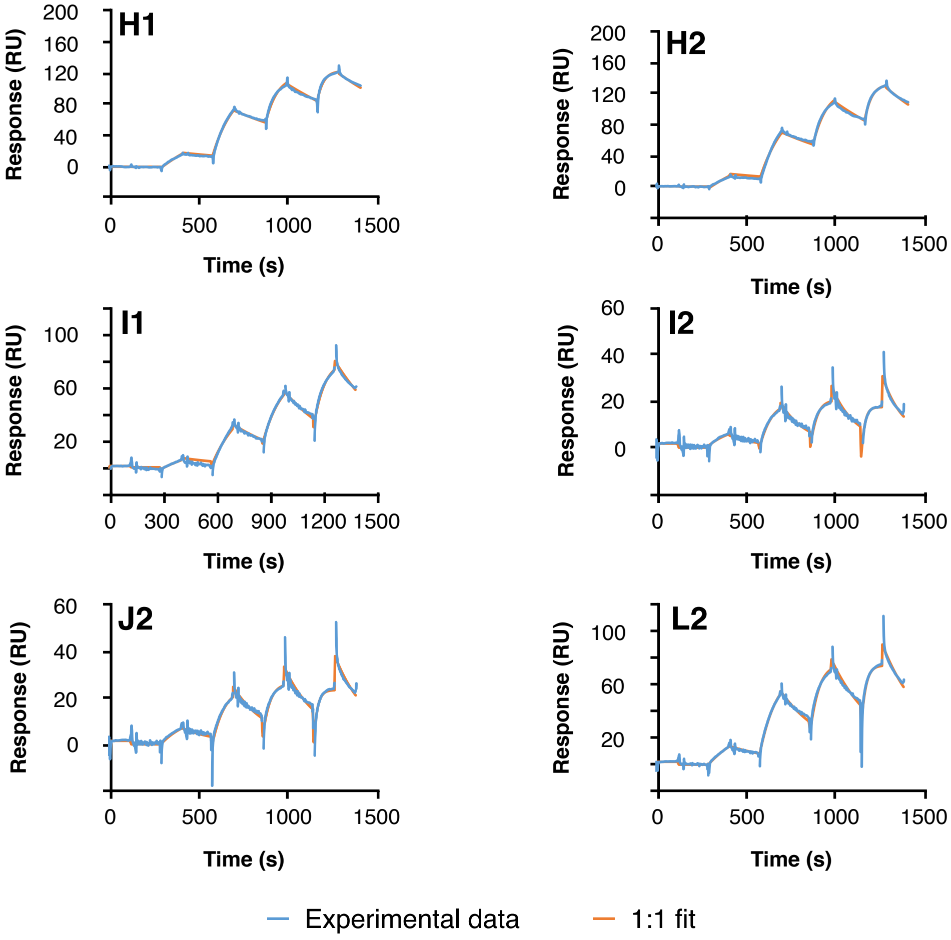

Figure S4: SPR of divalent LecA ligands from pyridine and sulfonated series. Sensorgrams obtained from SPR single-cycle kinetics experiments. Five different concentrations of each compound (0, 10, 50, 100, 200 nM) were sequentially injected to obtain the experimental sensorgrams (blue lines), which were then fitted by a 1:1 model (orange) on BIACORE evaluation software.

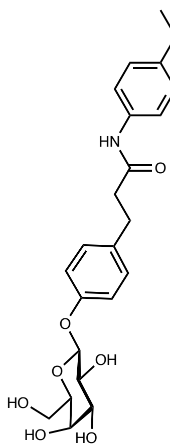

G2

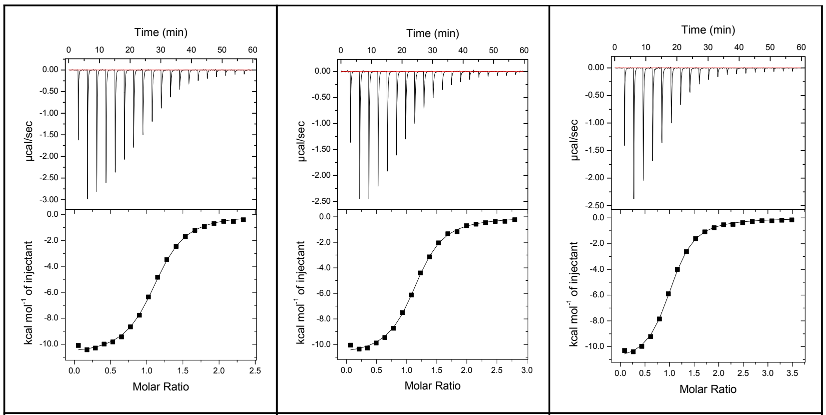

	126 μM LecA	106 μM LecA	85 μM LecA	
	1.5 mM **G2**	1.5 mM **G2**	1.5 mM **G2**	Average ± s.d.
ΔG [kcal/mol]	-7.2	-7.2	-7.2	-7.2 ± 0.01
ΔH [kcal/mol]	-10.9	-10.9	-11.3	-11.0 ± 0.2
ΔS [cal/mol/K]	-12.3	-12.5	-13.7	-12.8 ± 0.6
-TΔS [cal/mol]	3.7	3.7	4.1	3.8 ± 0.2
N	1.10	1.13	0.99	1.07 ± 0.06
K_d [μM]	5.32	5.26	5.24	5.27 ± 0.03

Figure S5: ITC measurements of monovalent ligand **G2** with LecA performed in TBS/Ca^{2+} buffer at 25 °C.

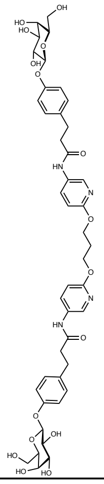

I2

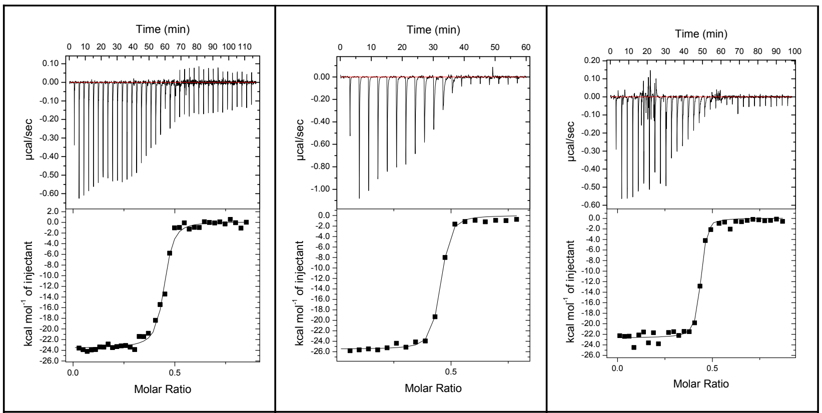

	50 μM LecA	50 μM LecA	40 μM LecA	
	100 μM **I2**	200 μM **I2**	100 μM **I2**	Average ± s.d.
ΔG [kcal/mol]	-10.0	-10.1	-10.6	-10.2 ± 0.2
ΔH [kcal/mol]	-23.6	-25.5	-22.7	-23.9 ± 1.2
ΔS [cal/mol/K]	-45.6	-51.6	-40.6	-45.9 ± 4.5
-TΔS [cal/mol]	13.6	15.4	12.1	13.7 ± 1.3
N	0.44	0.44	0.43	0.43 ± 0.01
K_d [nM]	48.3	38.8	18.3	35.1 ± 12.5

Figure S6: ITC measurements of divalent ligand **I2** with LecA performed in TBS/Ca^{2+} buffer with 5% DMSO at 25 °C.

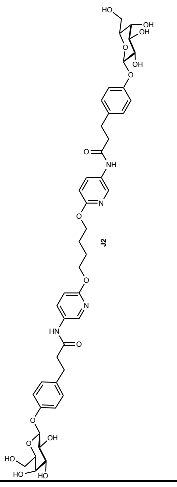

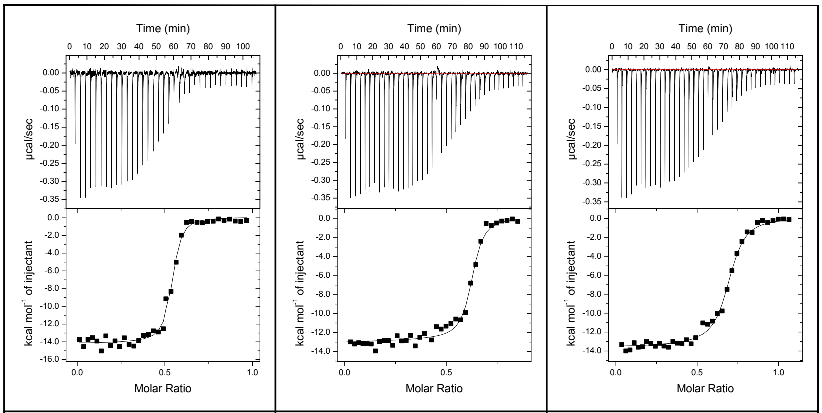

	40 μM LecA<br>100 μM **J2**	50 μM LecA<br>100 μM **J2**	40 μM LecA<br>100 μM **J2**	Average ± s.d.
ΔG [kcal/mol]	-9.9	-9.9	-9.4	-9.7 ± 0.2
ΔH [kcal/mol]	-14.0	-12.9	-13.5	-13.5 ± 0.4
ΔS [cal/mol/K]	-14.0	-10.1	-13.8	-12.6 ± 1.8
-TΔS [cal/mol]	4.2	3.0	4.1	3.8 ± 0.5
N	0.53	0.62	0.69	0.61 ± 0.06
K_d [nM]	59.88	52.91	125.79	79.5 ± 32.8

Figure S7: ITC measurements of divalent ligand **J2** with LecA performed in TBS/Ca^{2+} buffer with 5% DMSO at 25 °C.

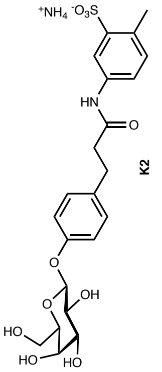

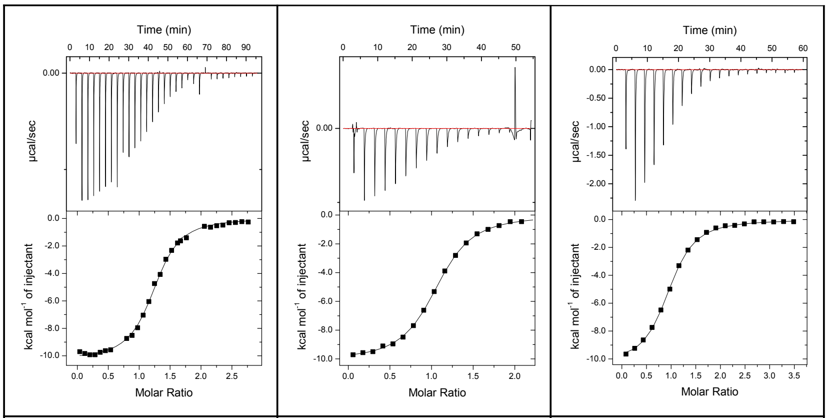

	182 μM LecA<br>1.5 mM **K2**	125 μM LecA<br>1.5 mM **K2**	85 μM LecA<br>1.5 mM **K2**	Average ± s.d.
ΔG [kcal/mol]	-7.1	-7.2	-7.1	-7.1 ± 0.1
ΔH [kcal/mol]	-10.3	-10.1	-10.4	-10.3 ± 0.1
ΔS [cal/mol/K]	-10.7	-10.0	-11.3	-10.7 ± 0.6
-TΔS [cal/mol]	3.2	3.0	3.4	3.2 ± 0.2
N	1.25	1.05	0.94	1.08 ± 0.13
K_d [μM]	6.33	5.65	6.71	6.23 ± 0.44

Figure S8: ITC measurements of monovalent ligand **K2** with LecA performed in TBS/Ca^{2+} buffer at 25 °C.

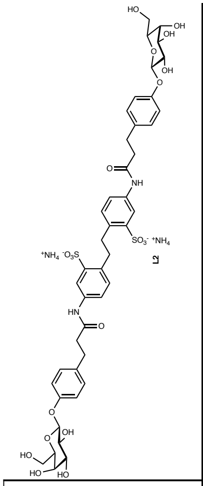

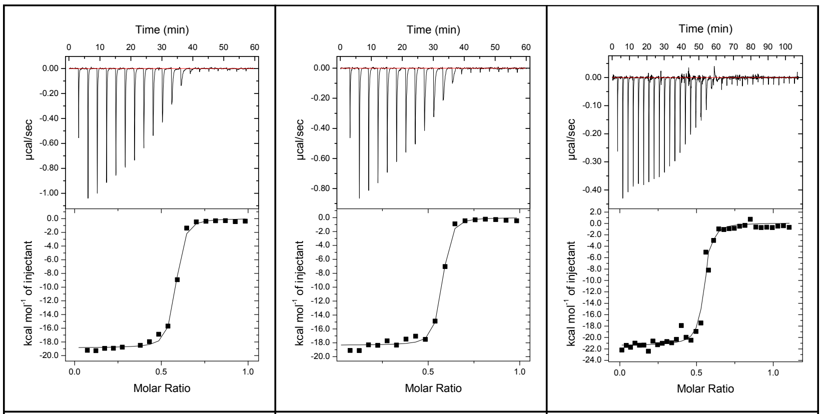

	50 μM LecA<br>250 μM **L2**	50 μM LecA<br>250 μM **L2**	35 μM LecA<br>100 μM **L2**	Average ± s.d.
ΔG [kcal/mol]	-10.0	-10.1	-10.1	-10.1 ± 0.1
ΔH [kcal/mol]	-18.9	-18.3	-21.4	-19.5 ± 1.3
ΔS [cal/mol/K]	-29.6	-27.5	-37.6	-31.6 ± 4.4
-TΔS [cal/mol]	8.8	8.2	11.2	9.4 ± 1.3
N	0.56	0.55	0.44	0.52 ± 0.06
K_d [nM]	44.8	38.0	36.8	39.9 ± 3.6

Figure S9: ITC measurements of **L2** with LecA performed in TBS/Ca^{2+} buffer at 25 °C.

Cell-based assays

Protein labelling

The lectin LecA was expressed in *E. coli* BL21 (DE3), transformed with the plasmid pET25-pa1l and purified as previously published.[12] For fluorescent labelling, Alexa Fluor 488 NHS Ester (Thermo Fisher Scientific Inc., Rockford, IL, USA) was dissolved at a final concentration of 10 mg/mL in DMSO (Carl Roth GmbH & Co. KG, Karlsruhe, Germany), according to the manufacturer´s protocol. For the labelling reaction, 100 µL of lectin (1 mg/mL) in PBS (Capricorn Scientific GmbH, Germany) was supplemented with 10 µL of a 1 M $NaHCO_3$ (pH 9) solution. Hereby, the molar ratio between dye and lectin was set to 5:1, and 0.63 µL of dye were added to the protein. The labelling mixture was incubated at 4 °C for 90 min, and uncoupled dye was removed using Zeba Spin desalting columns (7 kDa MWCO, 0.5 mL, Thermo Fischer Scientific Inc., Rockford, IL, USA). Labelled LecA (LecA-AF488) was stored at 4 °C, protected from light.

Cell culture

The human lung epithelial cell line H1299 (American Type Culture Collection, CRL-5803) was cultured in Roswell Park Memorial Institute (RPMI) 1640 medium supplemented with 10% fetal calf serum (FCS) and 2 mM L-glutamine, at 37 °C and 5% CO_2. Cells were incubated with different concentrations of LecA in the same medium for indicated time points.

Flow cytometry analysis

H1299 cells were detached with 2 mL of 1.5 mM EDTA in PBS, 1.2×10^5 cells were counted and transferred to a U-bottom 96 well plate (Sarstedt AG & Co. KG, Numbrecht, Germany). For flow cytometry assay, the optimal LecA concentration was established following a lectin titration (0.04–0.5 µM LecA-AF488) with H1299 cells. The histogram of fluorescence intensity of cells treated with 0.16 µM LecA exhibited 100% shift compared to the negative control (H1299 cells without any LecA-AF488) and did not reach signal saturation. Therefore, 0.16 µM LecA-AF488 was used in flow cytometry experiments. LecA-AF488 was preincubated with PNPG, **G2**, **K2**, **H2** or **L2** in RPMI medium, for 20 min at room temperature and protected from light. The ligand-LecA solution was then added to the cells and further incubated for 30 min at 4 °C. H1299 cells were resuspended with medium in absence of lectin or inhibitor represented the negative assay control, while cells incubated only with LecA-AF488 (no inhibitor) served as the positive control. Subsequently, cells were centrifuged at 1600 × *g* for 3 min at 4 °C to remove unbound lectin. The samples were then washed twice with ice-cold FACS buffer (PBS (-/-) supplemented with 3% FCS (*v/v*)). After the last washing step, the cells were re-suspended in FACS buffer and transferred to FACS tubes (Kisker Biotech GmbH Co. KG, Steinfurt, Germany) on ice and in absence of light. The fluorescence intensity of treated cells was measured with FACS Gallios from Beckman Coulter. The data were analyzed using FlowJo V.10.5.3.

LecA binding and cellular uptake by fluorescence confocal microscopy

Between 5 and 7×10^4 cells were seeded on a 12 mm glass coverslip to which they adhered overnight, at 37 °C and 5% CO_2. The next day, the cells were incubated with 0.5 µM LecA-AF488 for 30 min at 4 °C and protected from light. Where indicated, LecA was firstly preincubated with PNPG, **G2**, **K2**, **H2** or **L2** in RPMI medium, for 30 min at room temperature and protected from light and then added to the cells for further incubation (30 min, 4 °C). Subsequently, cells were washed with PBS (-/-) to remove the unbound lectin and inhibitors and the samples were incubated for 1 h at 37 °C. After the lectin stimulation, cells were washed twice with ice-cold PBS (-/-), fixed with 4% paraformaldehyde solution for 15 min and quenched with 50 mM ammonium chloride for 10 min. The membrane was permeabilized and cells were blocked by 0.2% Saponin in 3% BSA in PBS (*w/v*) for 30 min. The cell nuclei were counterstained with DAPI (5×10^{-9} g/L), and the samples were mounted on coverslips using Mowiol 4-88 (containing the anti-bleaching reagent DABCO). Samples were visualized with laser scanning confocal microscope system from Nikon (Eclipse Ti-E, A1R), equipped with a 60x oil immersion objective and a numerical aperture (*NA*) of 1.49. The images were analyzed

using NIS-Element Confocal 4.20 from Nikon and ImageJ 1.52a from Laboratory for Optical and Computational Instrumentation.

Cell proliferation (MTT) assay

The MTT assay (MTT Cell Proliferation Kit, Roche Holding AG, Basel, Switzerland) was used to evaluate the cytotoxicity of inhibitors on H1299 cells. 3.5 x 10^4 cells per well were transferred to a 96-well plate with a U-bottom. The cells were centrifuged at 1600 x g for 3 min at room temperature. The cell pellet was resuspended in 100 µL of inhibitor solutions (1–750 µM) and transferred to a 96-well flat-bottomed plate. The cells were incubated for 24 h at 37 °C. Subsequently, 10 µL of MTT labelling solution was added to each well and the cells were incubated for another 4 h at 37 °C. Afterwards, 100 µL of the kit solubilization reagent were added to each well and the plate was incubated at 37 °C overnight. The next day, the absorbance of the samples was measured at 550 nm using a BioTek microplate reader (BioTek Instruments Inc., Winooski, VT, USA). The data were analyzed using Microsoft Excel and GraphPad Prism software.

Scratch-Wound healing assay

2 x 10^5 cells were seeded on 12-well plates and formed a confluent monolayer after 24 h. The next day, a monolayer was scratched with a 200 µL pipette tip to create a wound. The cells were washed with PBS (-/-) and LecA (3.9 µM) or LecA-inhibitor solutions (3.9 µM LecA + 10 µM **L2** or 3.9 µM LecA + 100 µM **L2**) were added. The cells were incubated with the lectin-inhibitor solution for 24 h at 37 °C. In order to depict exactly the same position for each wound, marker lines were drawn on the bottom of the 12-well plates for each well. The samples were imaged with an EVOS microscope (PEQLAB by VWR Life Sciences, Avantor Performance Materials Inc., Center Valley, PA, USA) and analyzed with ImageJ and Microsoft Excel. Measurements were taken at time 0 h and 24 h for each condition in $N = 3$ replicas per time. Experiment was repeated independently three times (N = 3) and evaluated with two tailed, unpaired t-test.

Invasion Assay

A chromosomally GFP-tagged Gentamicin-resistant *Pseudomonas aeruginosa* PAO1 strain was cultured (LB medium supplemented with 60 µg/mL Gentamicin, 37 °C, 180 rpm, 16 h) and invasion assays were performed in analogy to Eierhoff *et al.*.[13] 1.5 x 10^4 H1299 cells per well were seeded one day prior to the invasion assay in a 96-well plate with flat bottom. An

overnight culture of *P. aeruginosa* was centrifuged for 5 min at 3000 x *g*, washed with PBS (-/-) and re-suspended in RPMI 1640 medium. The newly synthesized divalent LecA ligand **L2** was compared to 4-nitrophenyl α-D-galactopyranoside (PNPG; Sigma-Aldrich, Chemie GmbH, Darmstadt, Germany). For 10 mM, PNPG was added as solid directly to the bacterial suspension. For 100 μM, PNPG or **L2** were first dissolved in PBS (-/-) and then added to *P. aeruginosa* culture. Bacterial suspensions were incubated with PNPG or **L2** in RPMI medium for 30 min at room temperature at 600 rpm. H1299 cells were washed once with PBS (-/-) and incubated with bacteria-ligand solutions for 2 h at 37 °C and 5% CO_2. The target multiplicity of infection (MOI) was set to 100, i.e. we aimed to have 100 bacteria per human host cell. Afterwards, H1299 cells were washed three times with PBS (-/-) and extracellular bacteria still attached to the cells were killed off by treatment with 400 μg/mL amikacin disulfate salt (Sigma-Aldrich, Chemie GmbH, Darmstadt, Germany) in RPMI for 2 h at 37 °C. Subsequently, cells were washed three times with DPBS and lysed by incubation with 0.25% (v/v) Triton X-100 (Carl Roth GmbH & Co. KG, Karlsruhe, Germany) for 5 min at 37 °C and 180 rpm. Lysates were diluted, plated on LB agar (Carl Roth GmbH & Co. KG, Karlsruhe, Germany) plates with 60 μg/mL gentamicin (anprotec, Bruckberg, Germany) and incubated at 37 °C overnight. For each condition, two to three dilutions were plated in triplicates. Bacterial colonies were counted the next day. Each condition was tested at least in duplicates per experiment and colony counts were averaged. The invasion rate describes how many bacteria successfully invaded host cells compared to the control (non-treated bacteria). The invasion rate was calculated as the number of intracellularly localized bacteria divided by the total number of non-treated bacteria, bacteria that were not treated with any ligands and neither amikacin, thus the number of *P. aeruginosa* that successfully invaded or stayed attached to the host cells. Experiments were repeated independently at least four times ($N \geq 4$) and evaluated with two tailed, paired t-test.

Statistical analysis

The number of statistical replicates (N) is indicated and the data are presented as mean ± standard deviation (SD). Statistical analysis was performed using Microsoft Excel and GraphPad Prism software. The IC_{50} values were obtained by non-linear regression analysis (GraphPad Prism). Statistical significance in independent, identical samples were determined with a two-tailed, unpaired t-test, if not indicated otherwise. Data with a p-value ≤ 0.05 are considered statistically significant and marked with an asterisk (*). A p-value of ≤ 0.01 is a very significant (**), and ≤ 0.001 is a highly significant result (***). Non-significant results were not highlighted.

Chemical Reagents

The following reagents were obtained from commercial sources: RPMI 1640, FCS, and L-glutamine were all purchased from Gibco (Thermo Fisher Scientific Inc., Waltham, MA, USA). PBS (-/-) was supplied by Capricorn Scientific GmbH, Germany. The following chemicals were obtained from Roth: BSA, DABCO, DAPI, EDTA, Mowiol, NH_4Cl, paraformaldehyde.

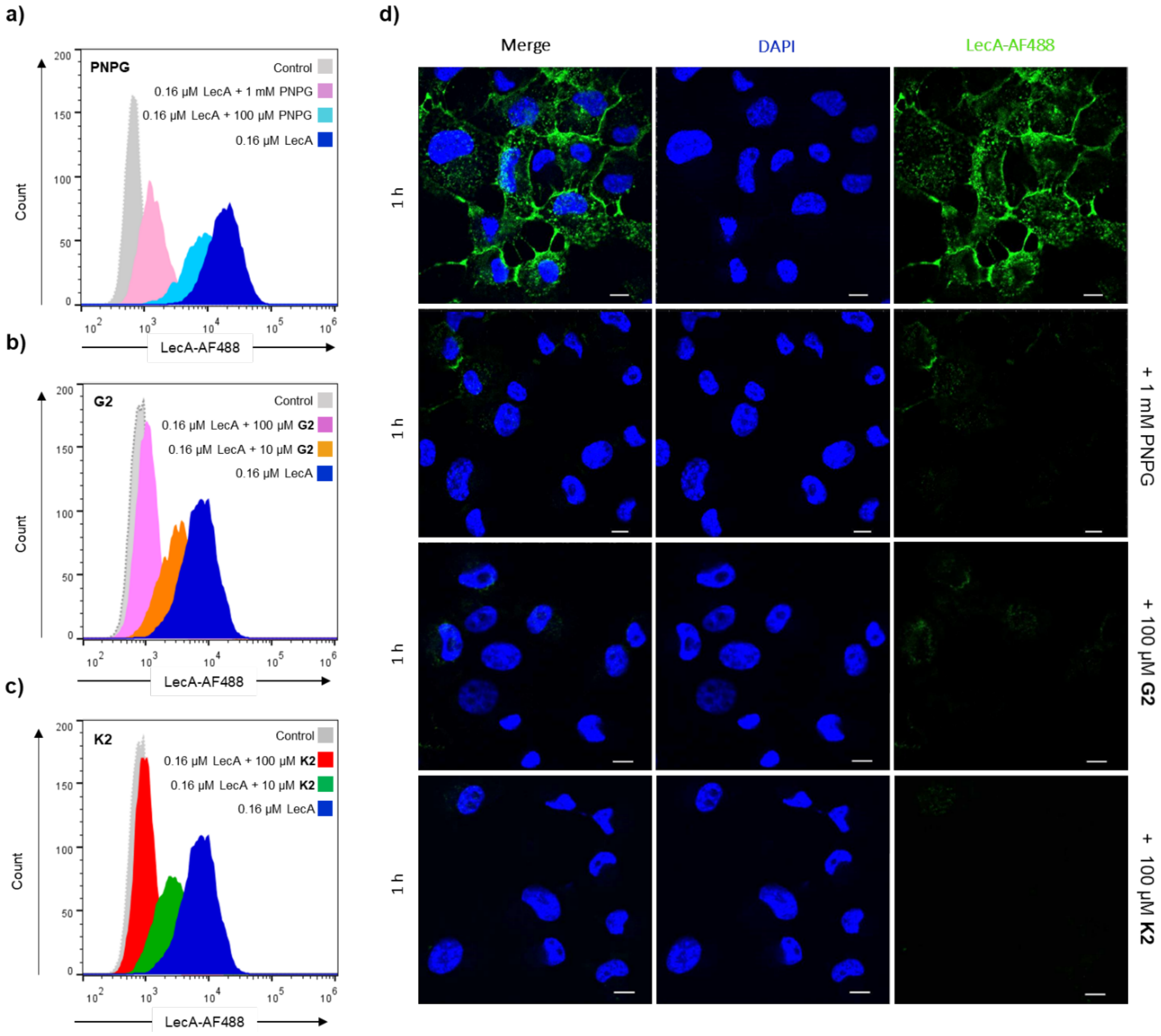

Figure S10: Decreased binding and uptake of LecA to H1299 with PNPG and monovalent inhibitors **G2** and **K2** (N = 3). Representative histograms fluorescence intensity of gated live H1299 cells incubated with 0.16 µM of LecA-AF488 in presence of (a) **PNPG**,(b) **G2** and (c) **K2**. H1299 cells (without LecA, **PNPG**, **G2** or **K2**) served as a negative control (grey). (d) Confocal imaging of H1299 cells incubated with 0.5 µM LecA-AF488 (in green) or LecA-AF488 which was preincubated with inhibitors (**PNPG**, **G2** or **K2**). Nuclei were counterstained by DAPI (in blue). Scale bars = 10 µm.

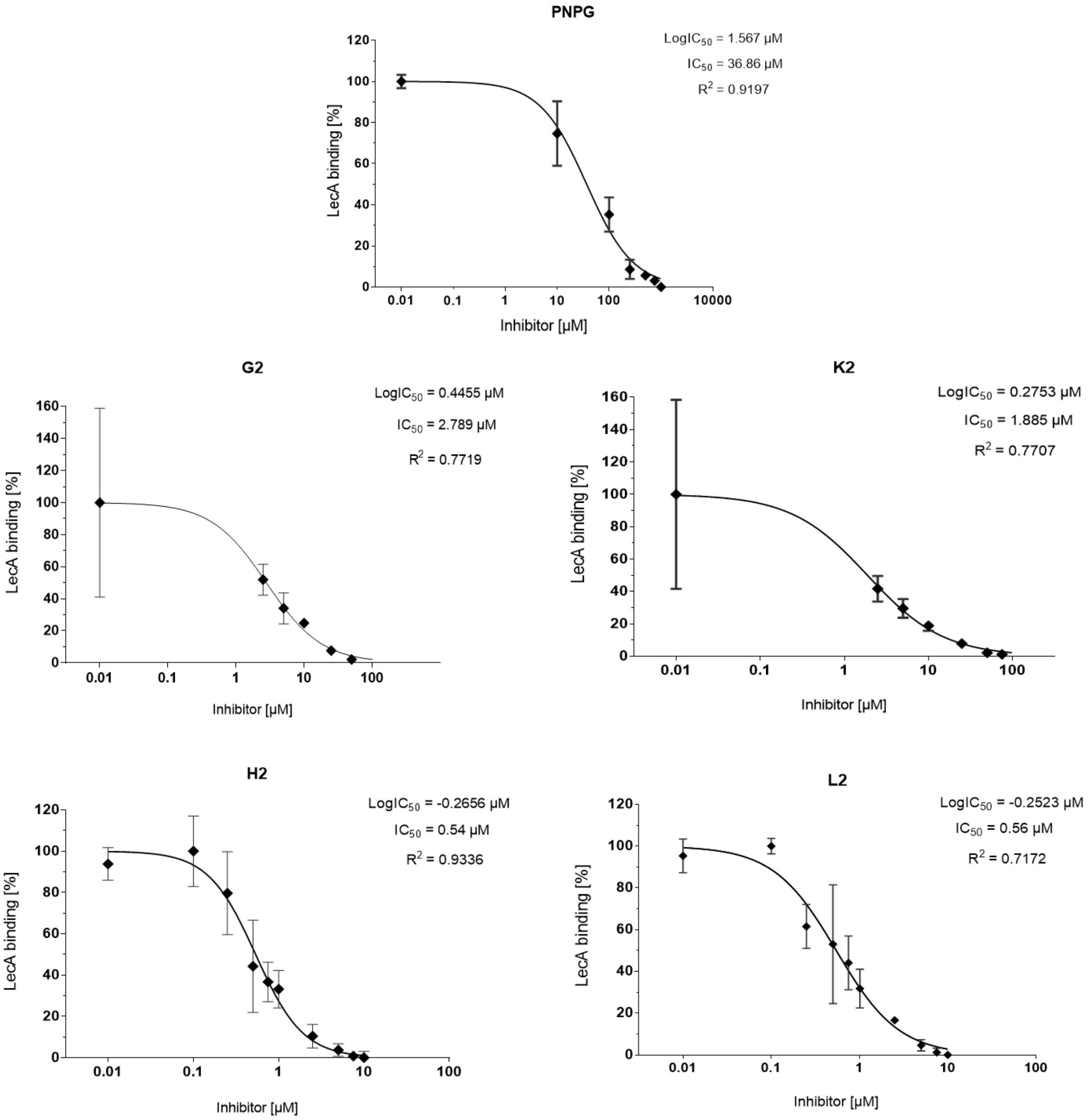

Figure S11: Dose-response curves of LecA-AF488 binding to H1299 cells after preincubation with **PNPG**, monovalent ligands **G2** and **K2**, and divalent ligands **H2** and **L2** (N = 3). Dose-response curves indicate the percentage of LecA binding to H1299 cells in presence of PNPG, monovalent, or divalent inhibitors. Mean Fluorescence Intensity (MFI) of samples treated with LecA and inhibitors were normalized to the negative control (H1299 cells) and plotted as a function of inhibitor concentration. 100% represent LecA binding to H1299 in absence of inhibitors. Error bars represent standard deviation.

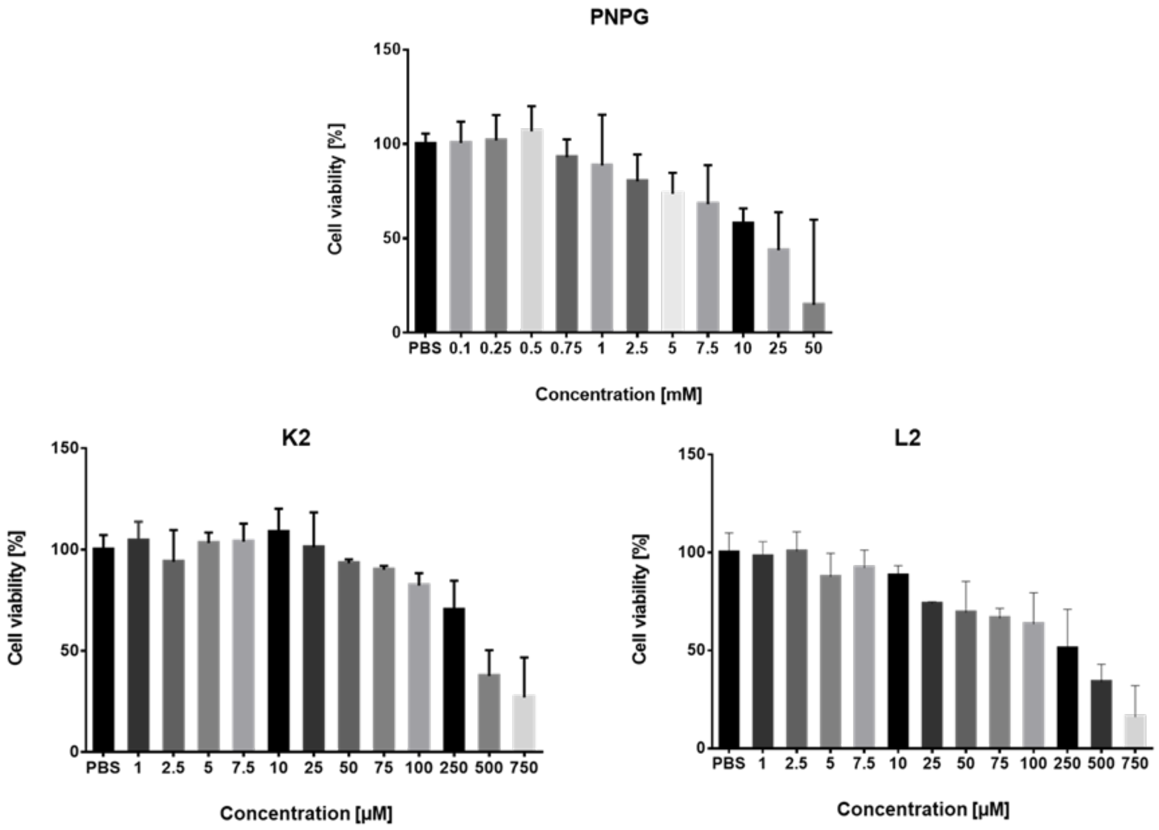

Figure S12: Cytotoxicity assay for selected LecA ligands. Dose-dependent assessment of H1299 cell viability after addition of PNPG, mono- and divalent inhibitors in a standard MTT assay (N = 3). Percentage of cell viability after 24 h treatment with (a) **PNPG** (0.1–50 mM), (b) monovalent **K2** (1–750 µM) and (c) divalent **L2** (1–750 µM). The bars represent the means, while the error bars show the standard deviations of three independent experiments.

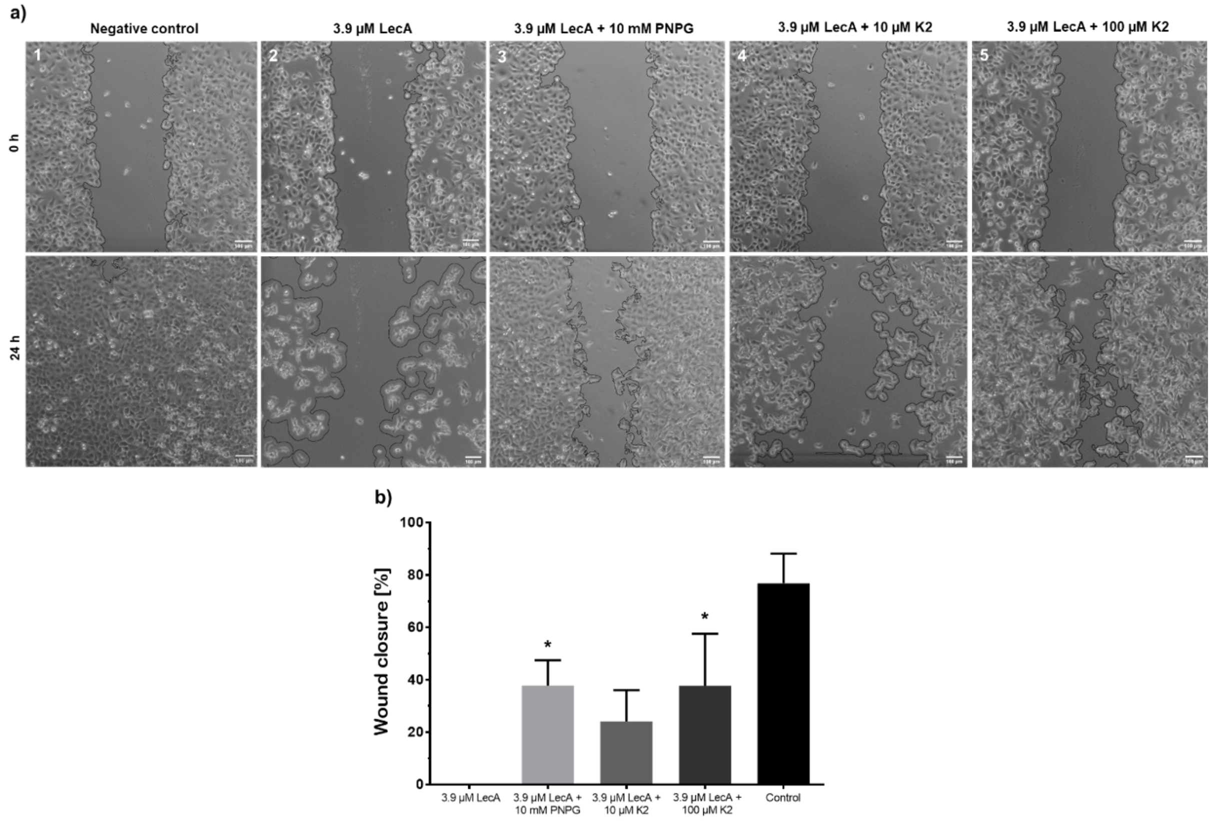

Figure S13: LecA impaired cell migration, but wound healing was restored in presence of **PNPG** and monovalent ligand **K2** (N = 3). (a) Light microscopy image of scratched H1299 cells at 0 h and after 24 h treatment with (1) PBS, (2) 3.9 µM LecA, (3) 3.9 µM LecA preincubated with 10 mM PNPG, (4) 3.9 µM LecA preincubated with 10 µM **K2** and (5) 3.9 µM LecA preincubated with 100 µM **K2**. Scale bars = 100 µm. (b) Quantification of wound closure after 24 h. Measurements were taken at time 0 h and 24 h for each condition. The values represent the means, while the error bars show the standard deviations of three independent experiments. *$p < 0.05$ (two-tailed, unpaired t-test).

HPLC-UV purity chromatograms

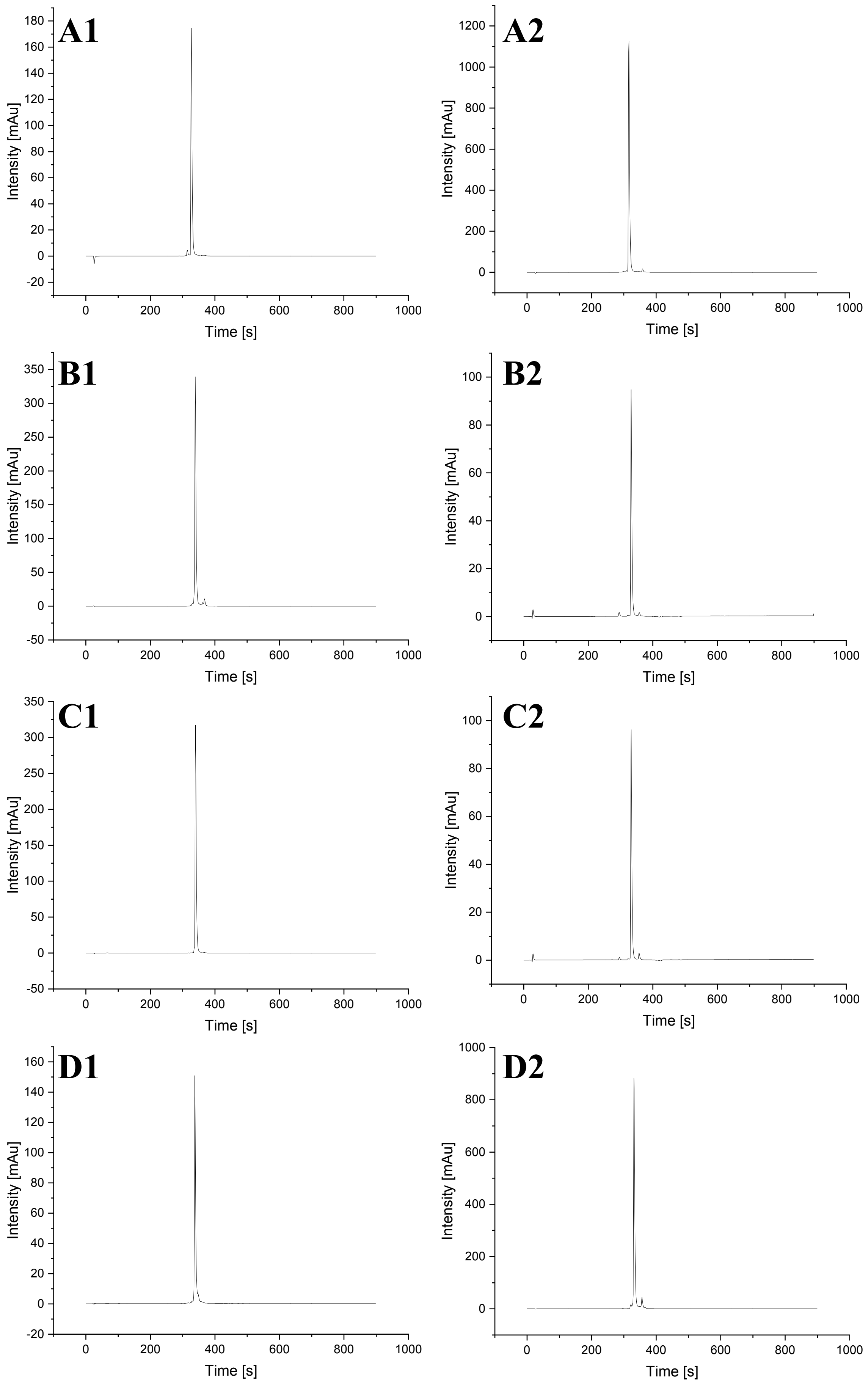

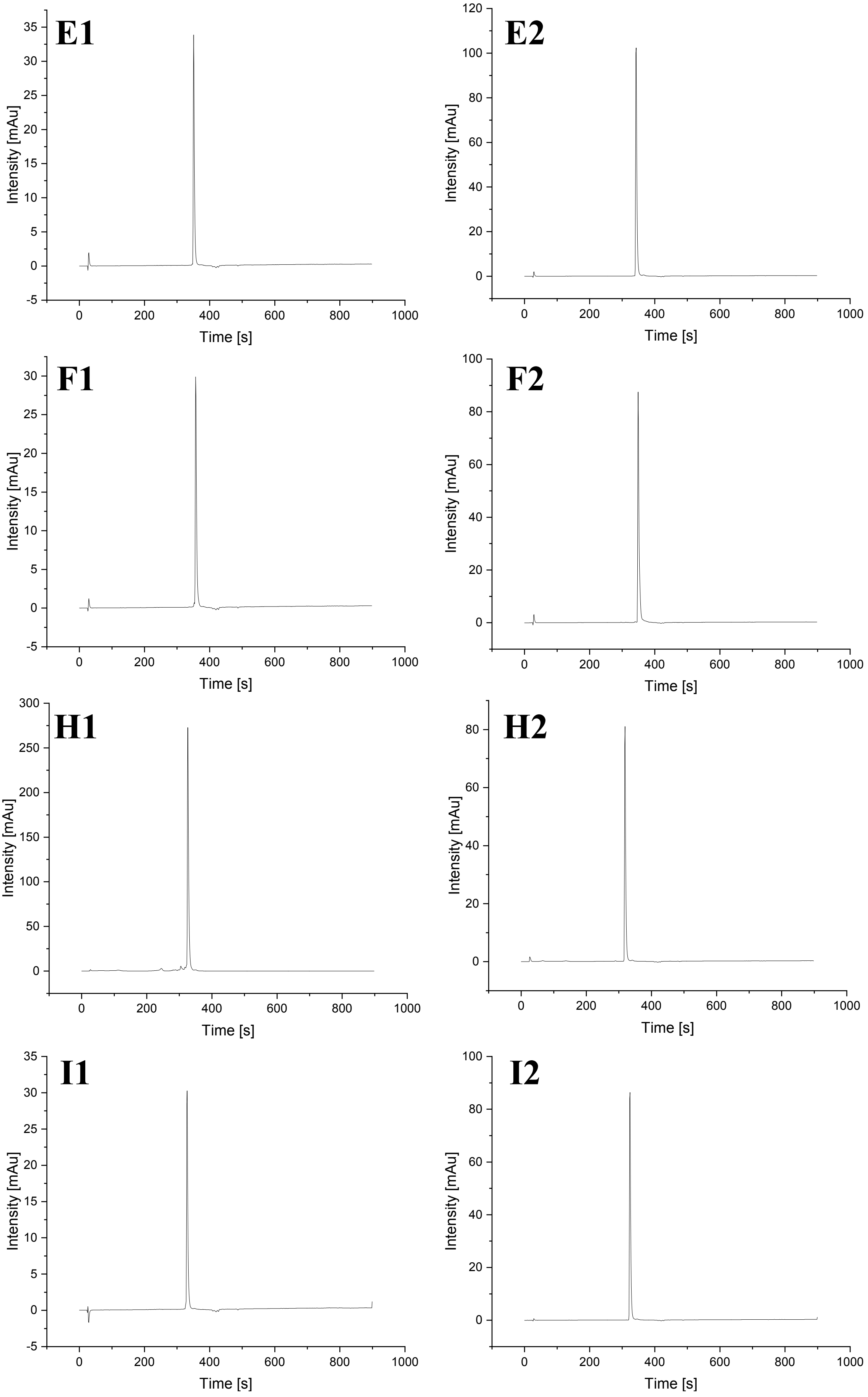
E1
E2
F1
F2
H1
H2
I1
I2
Intensity [mAu]
Time [s]

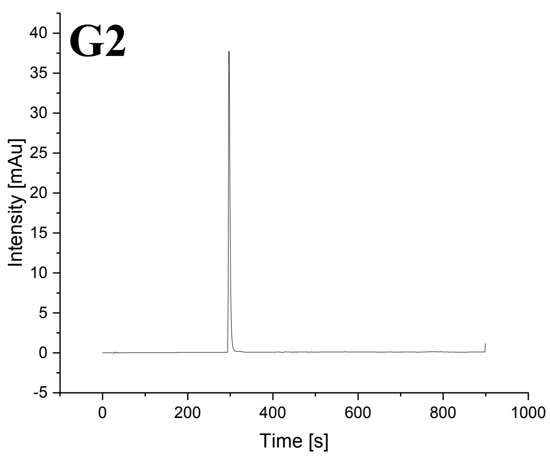
G2
Intensity [mAu]
Time [s]
-5
0
5
10
15
20
25
30
35
40
0
200
400
600
800
1000

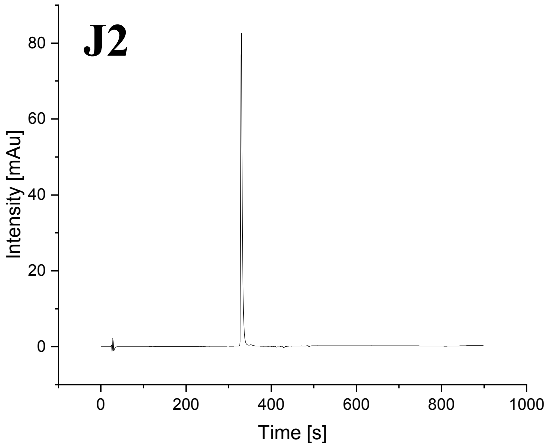
J2
Intensity [mAu]
Time [s]
0
20
40
60
80
0
200
400
600
800
1000

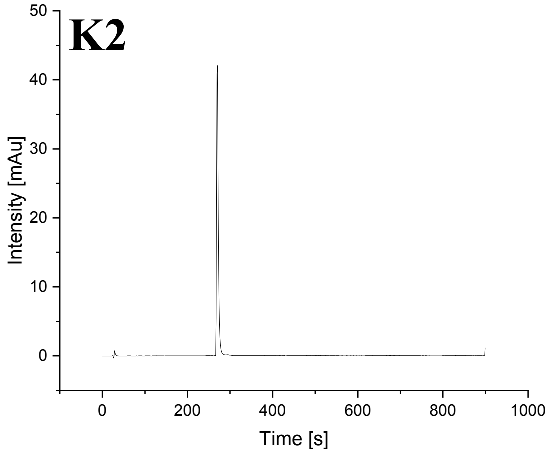
K2
Intensity [mAu]
Time [s]
0
10
20
30
40
50
0
200
400
600
800
1000

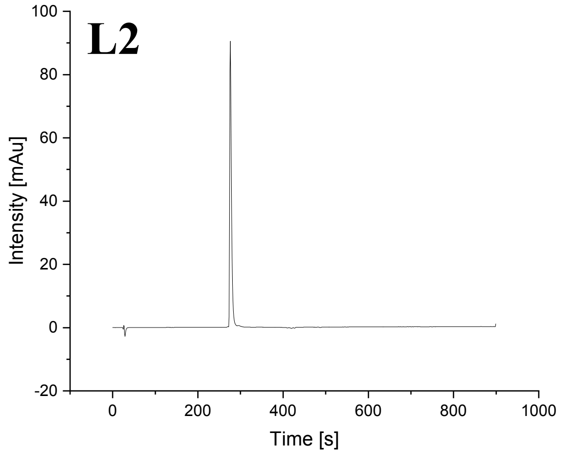
L2
Intensity [mAu]
Time [s]
-20
0
20
40
60
80
100
0
200
400
600
800
1000

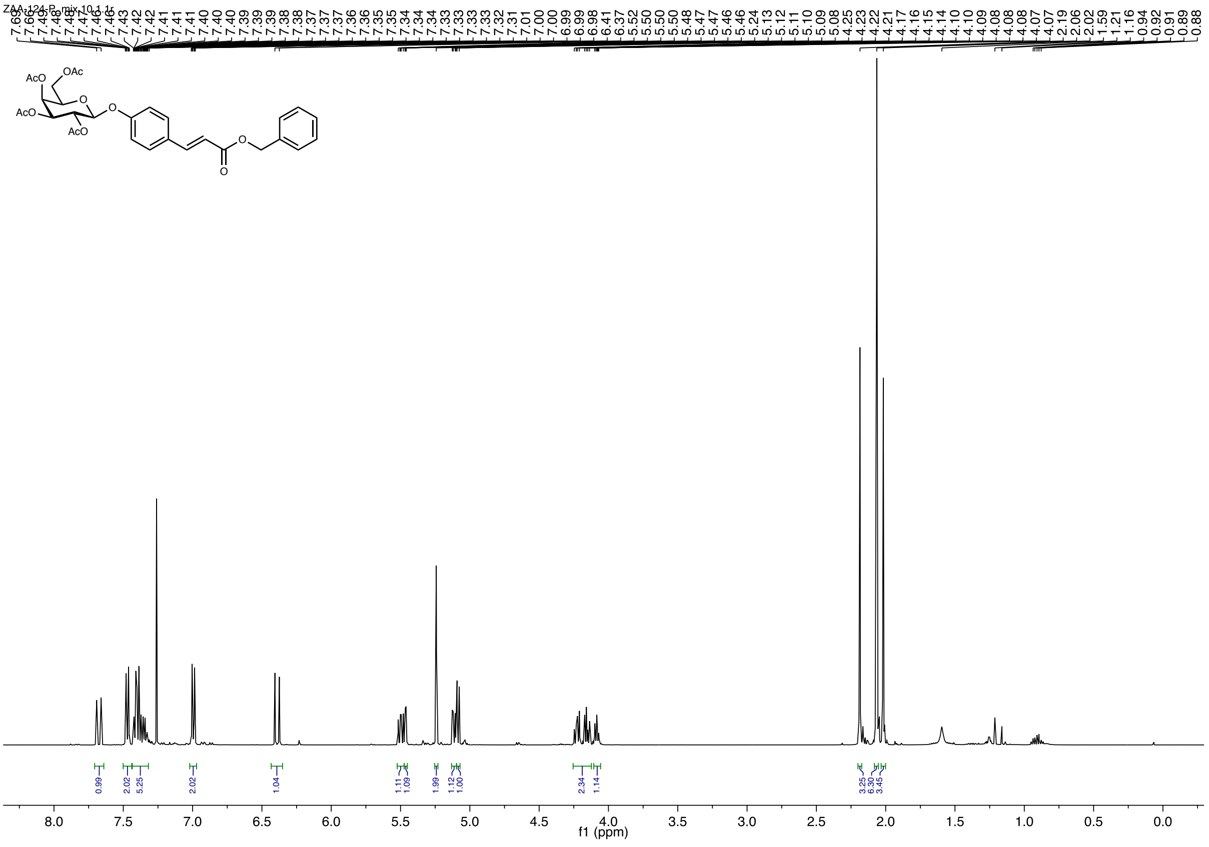

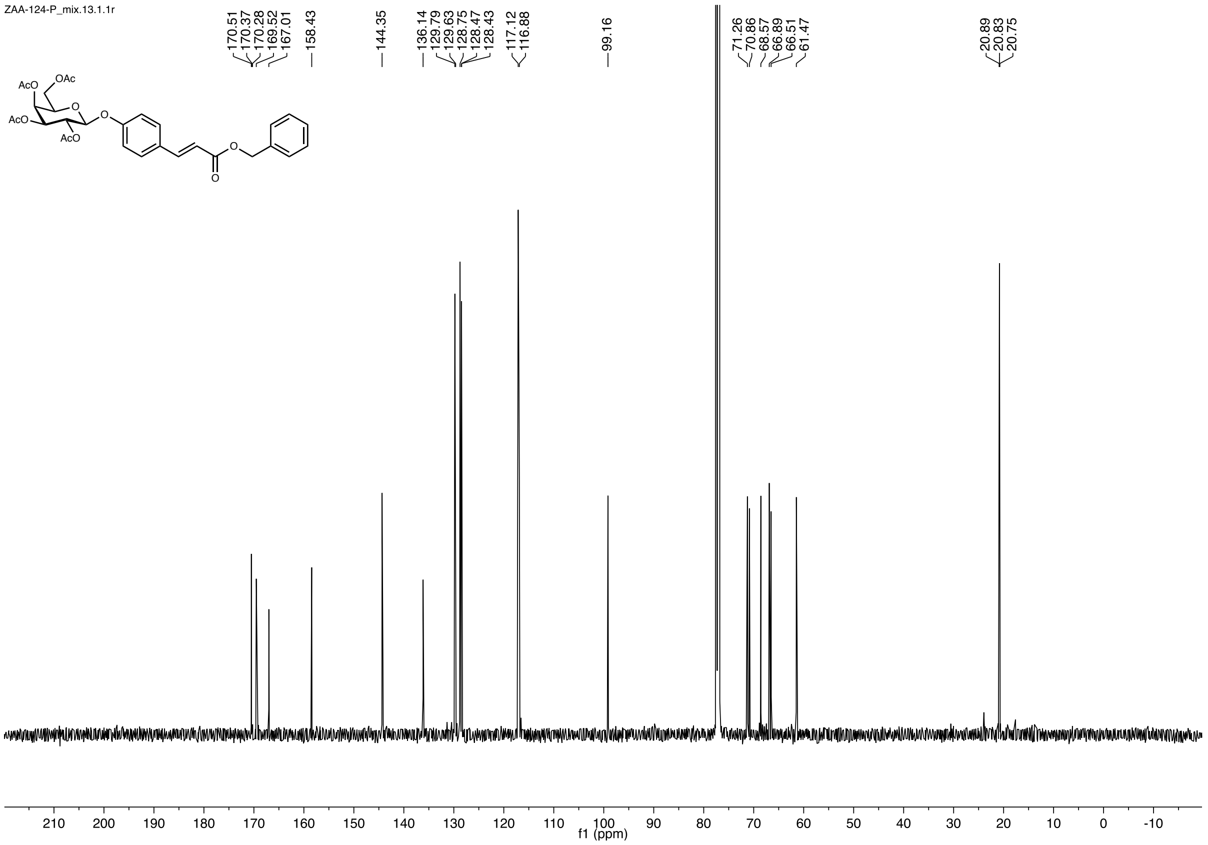

^{1}H and ^{13}C NMR of **4**

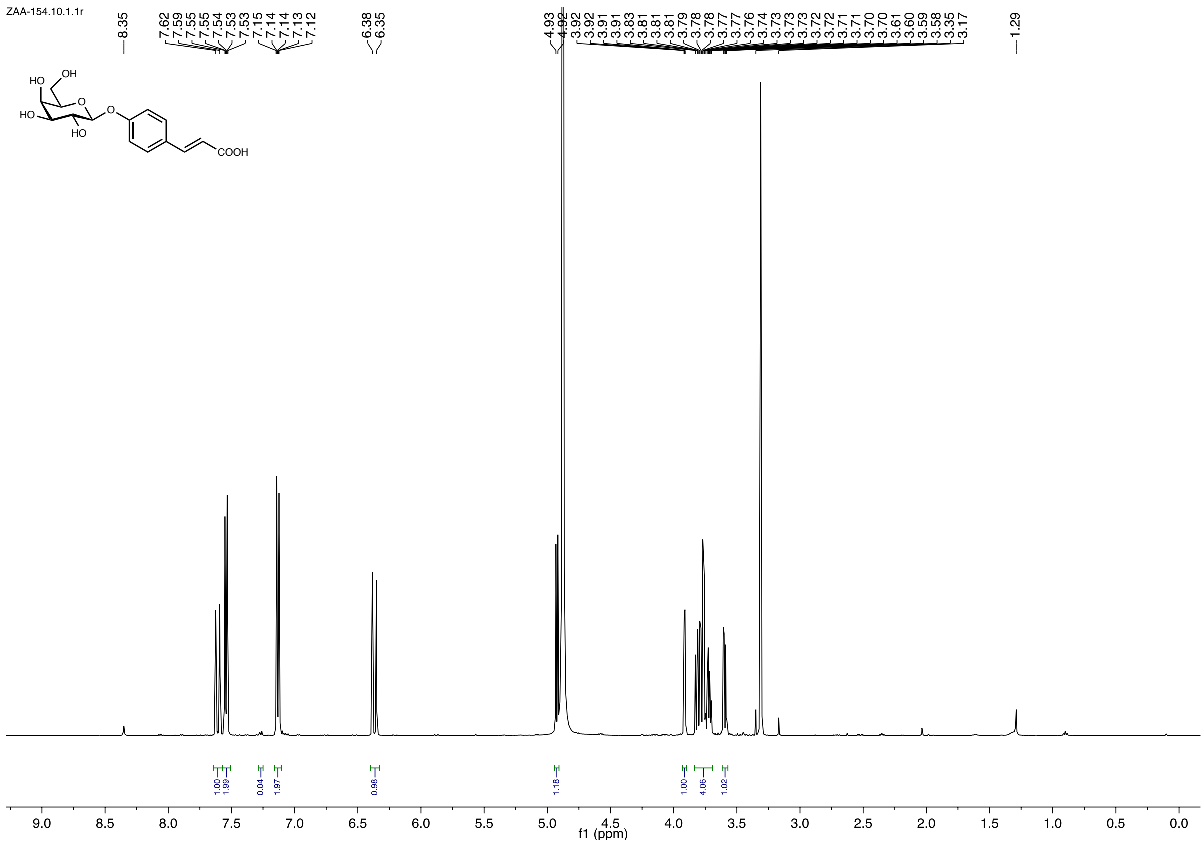

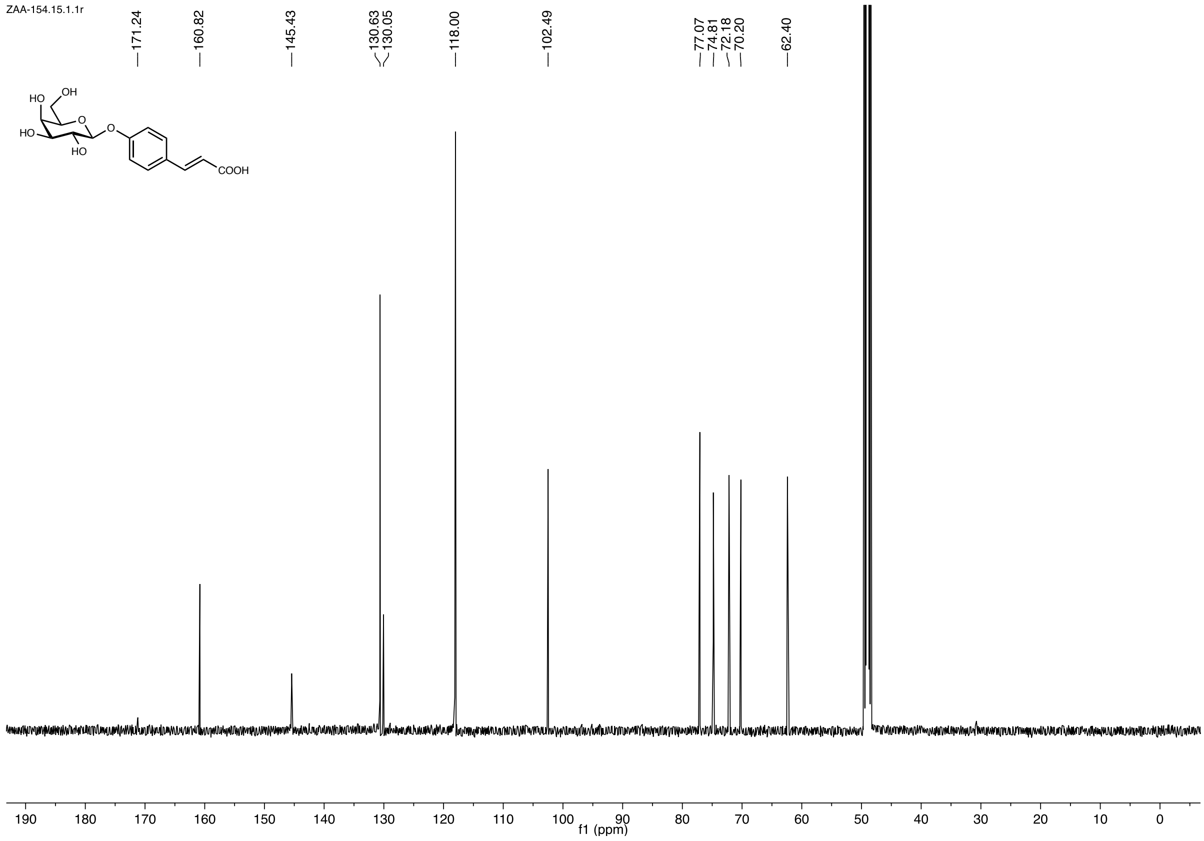

^{1}H and ^{13}C NMR of **1**

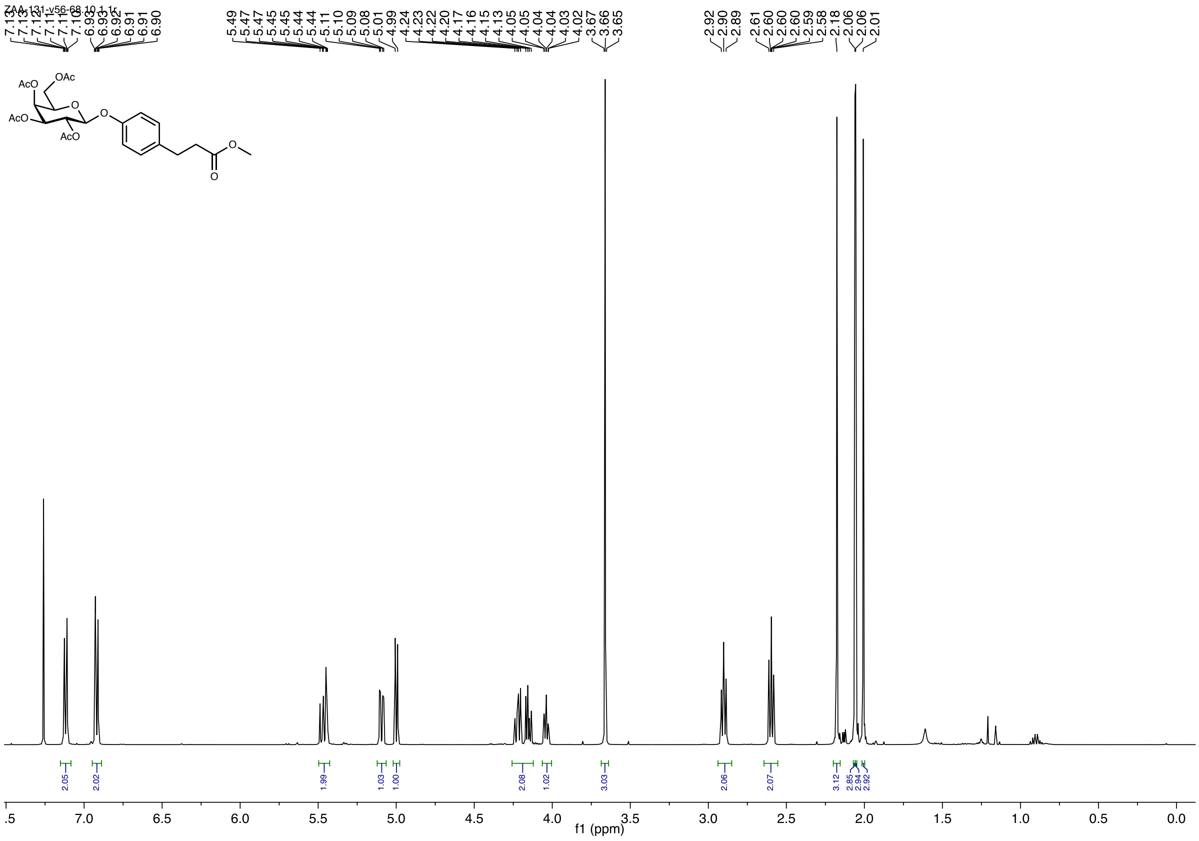

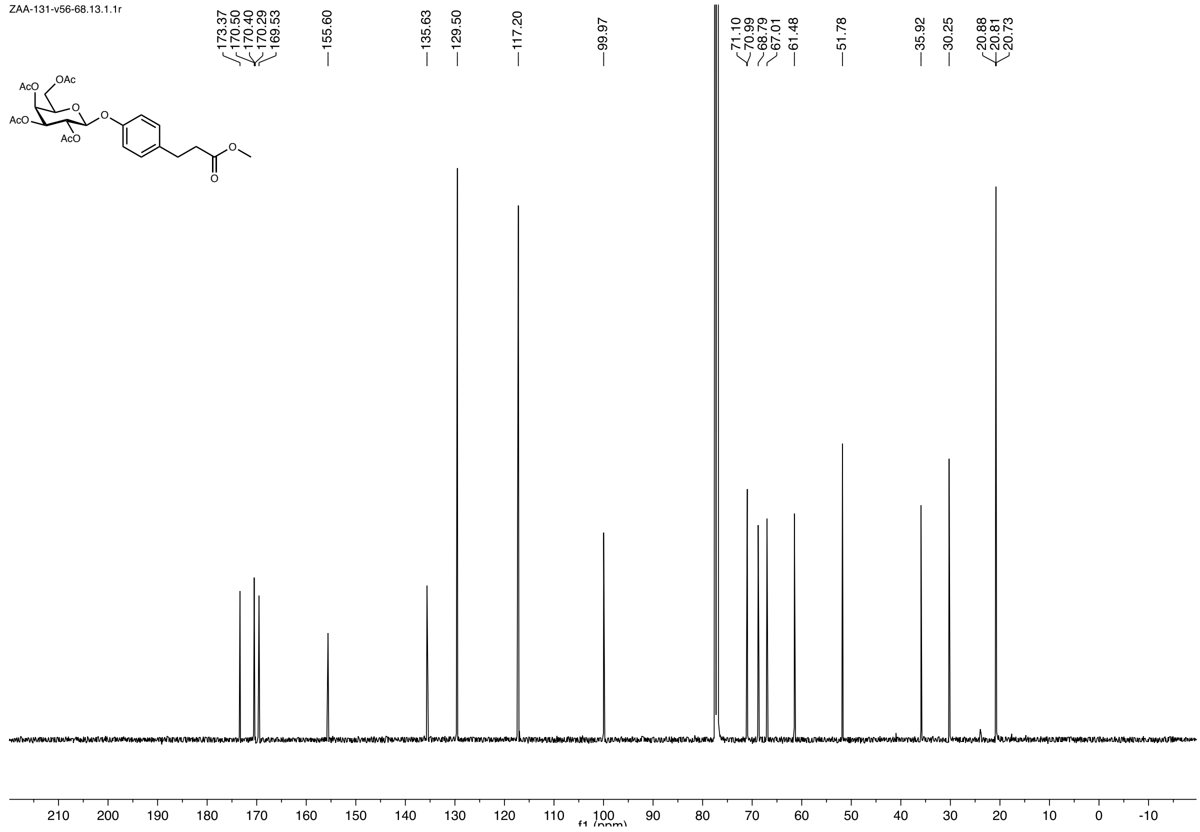

^{1}H and ^{13}C NMR of **5**

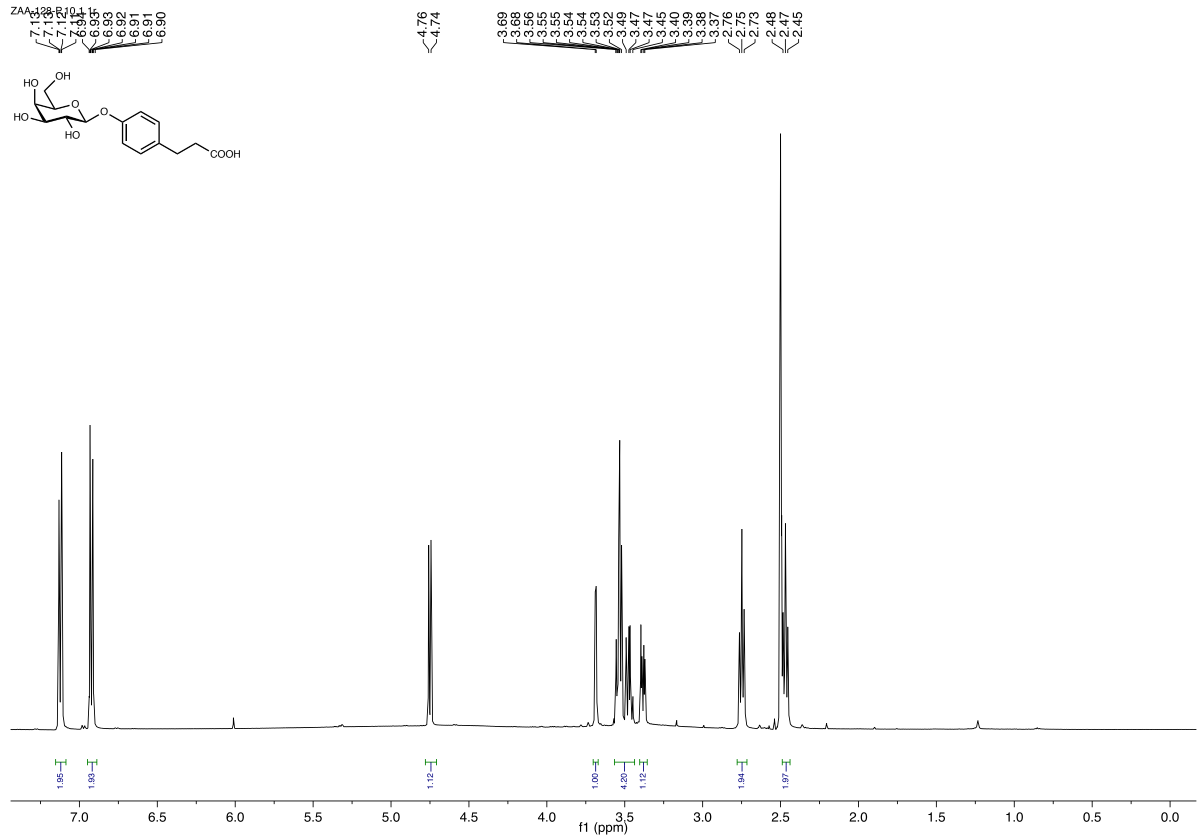

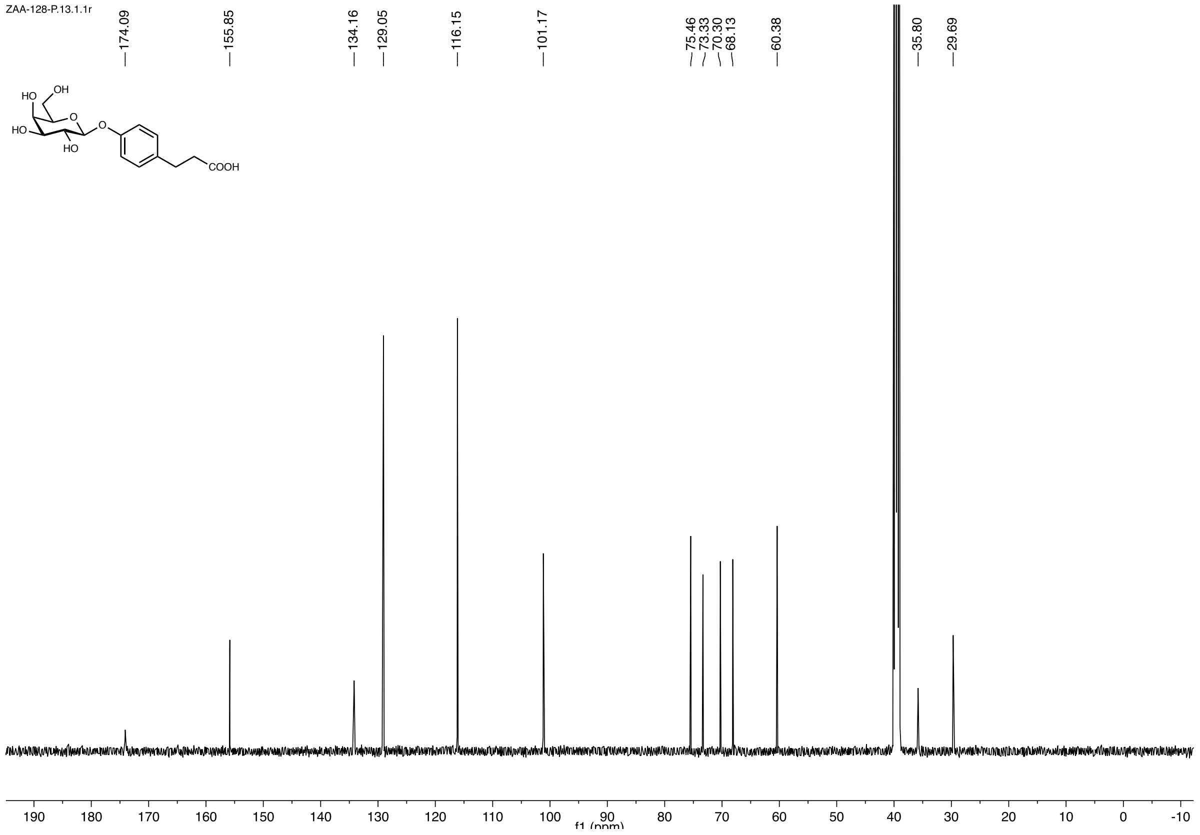

^{1}H and ^{13}C NMR of **2**

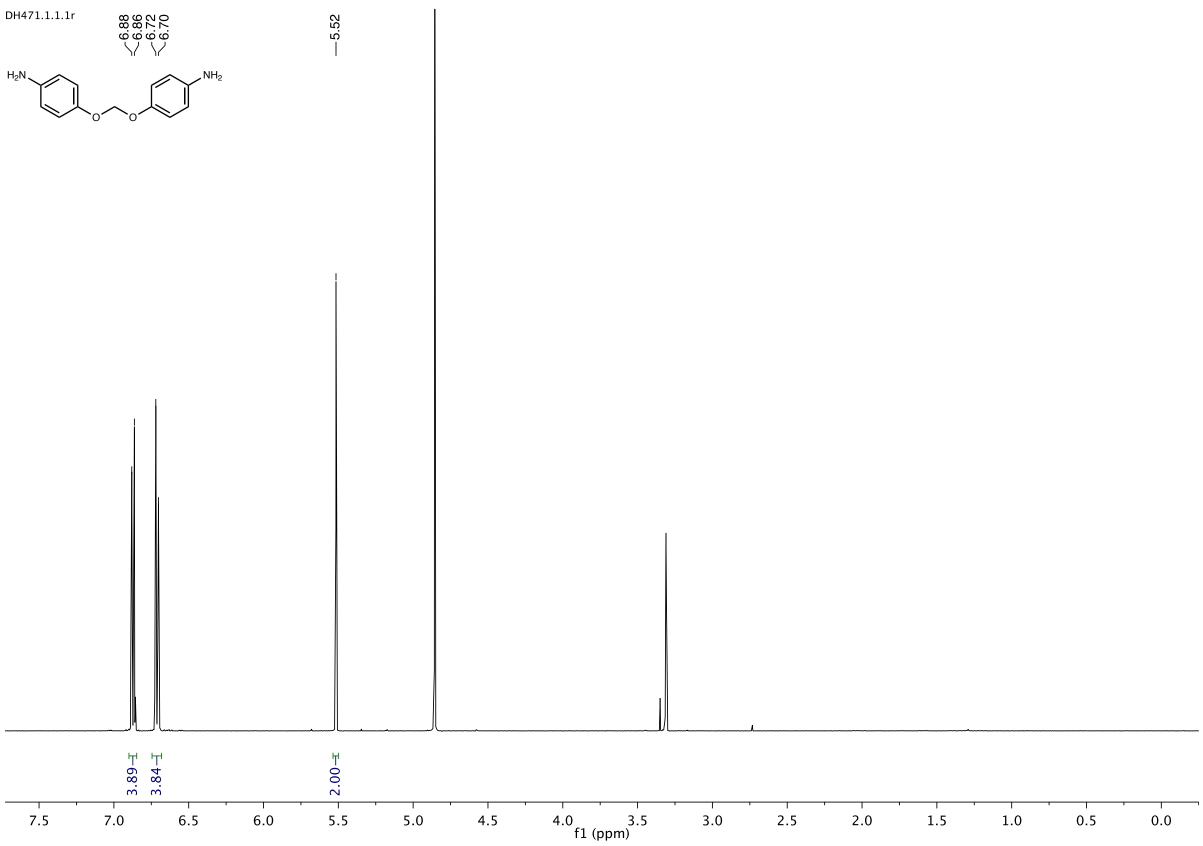

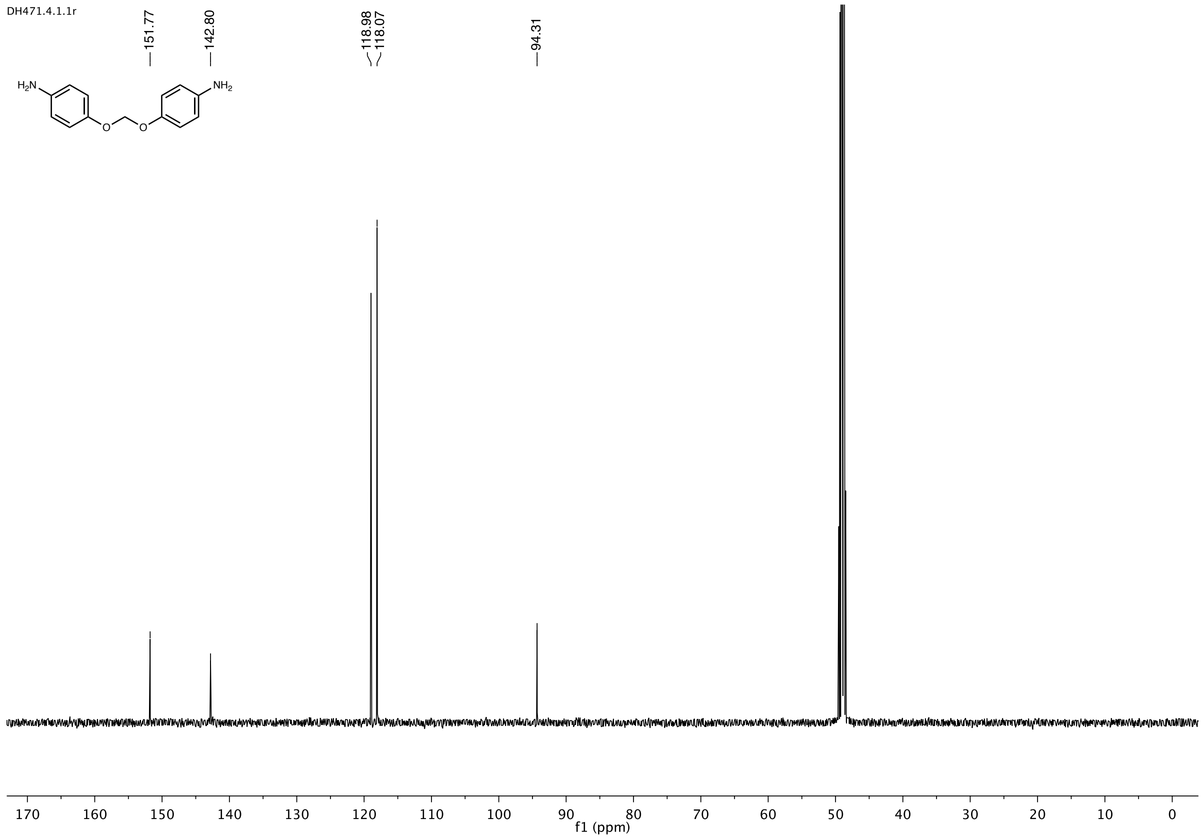

^{1}H and ^{13}C NMR of **C**

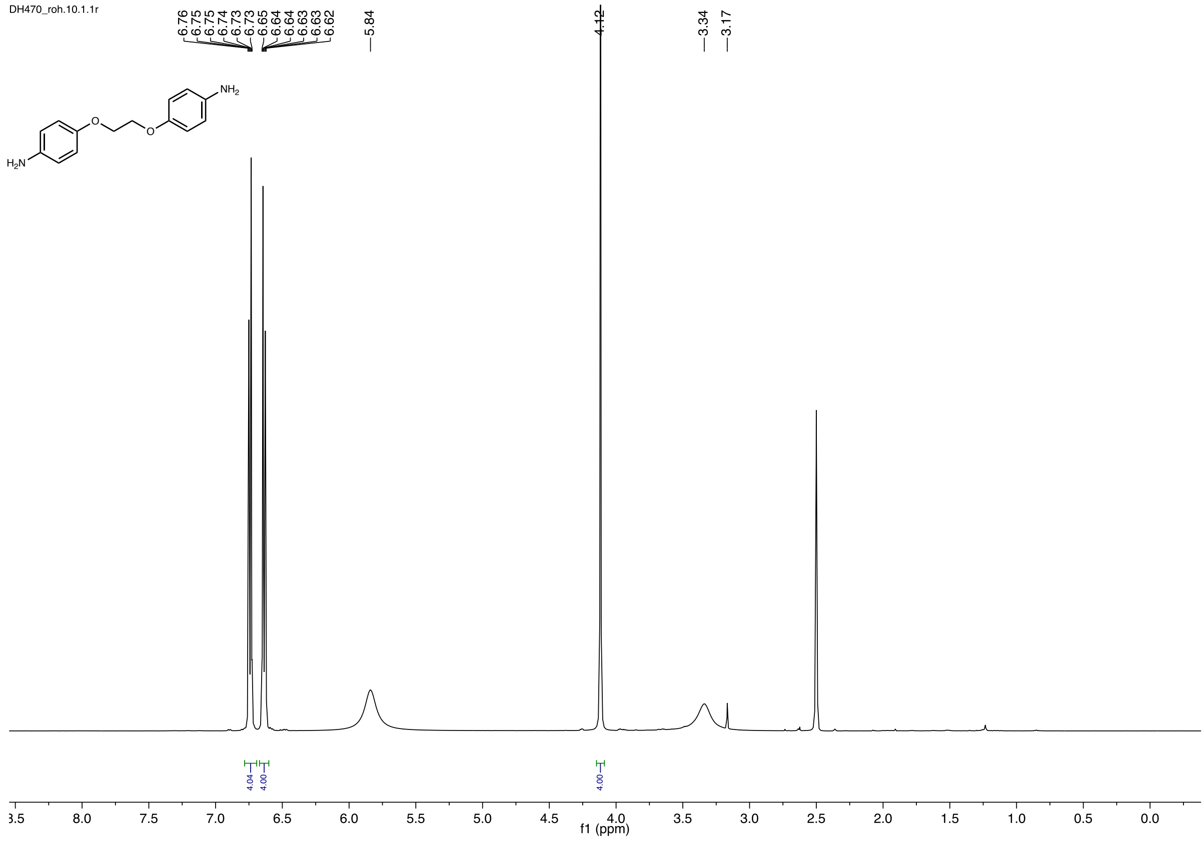

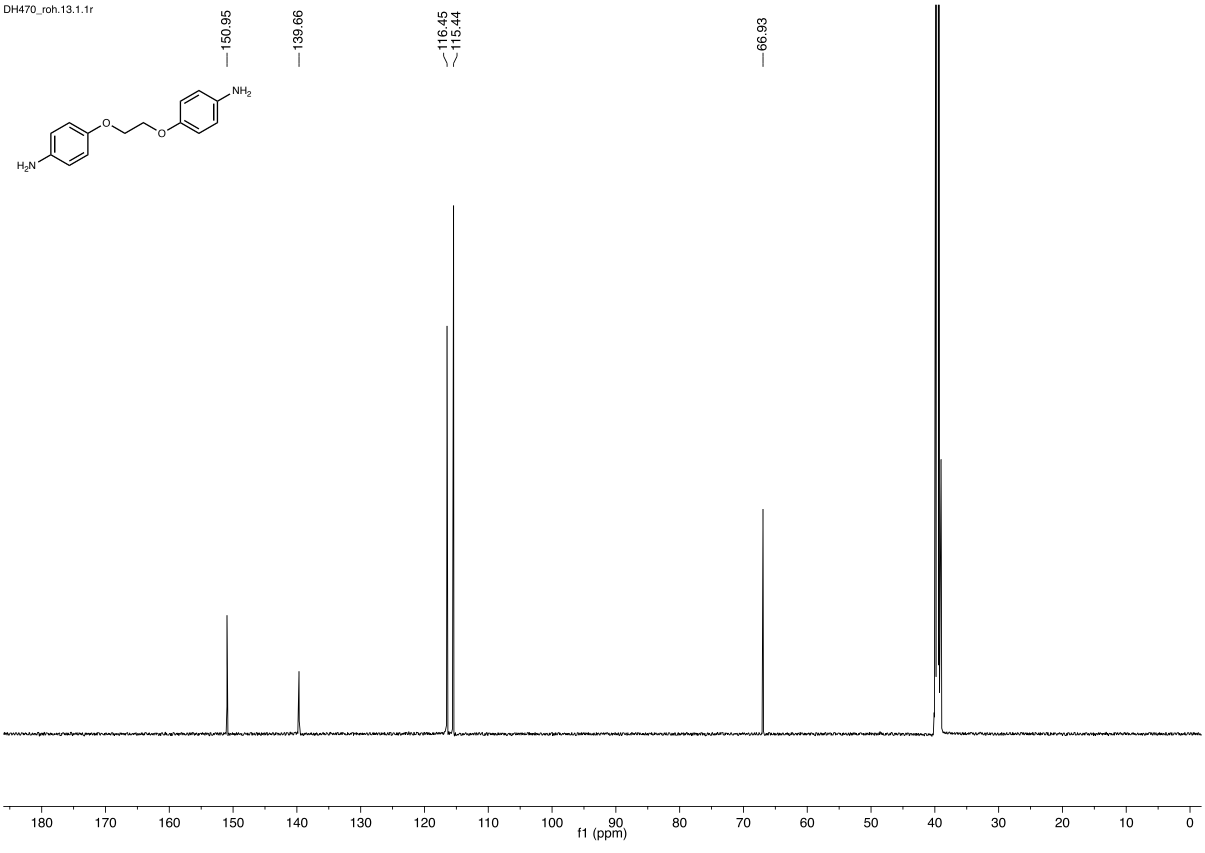

^{1}H and ^{13}C NMR of **D**

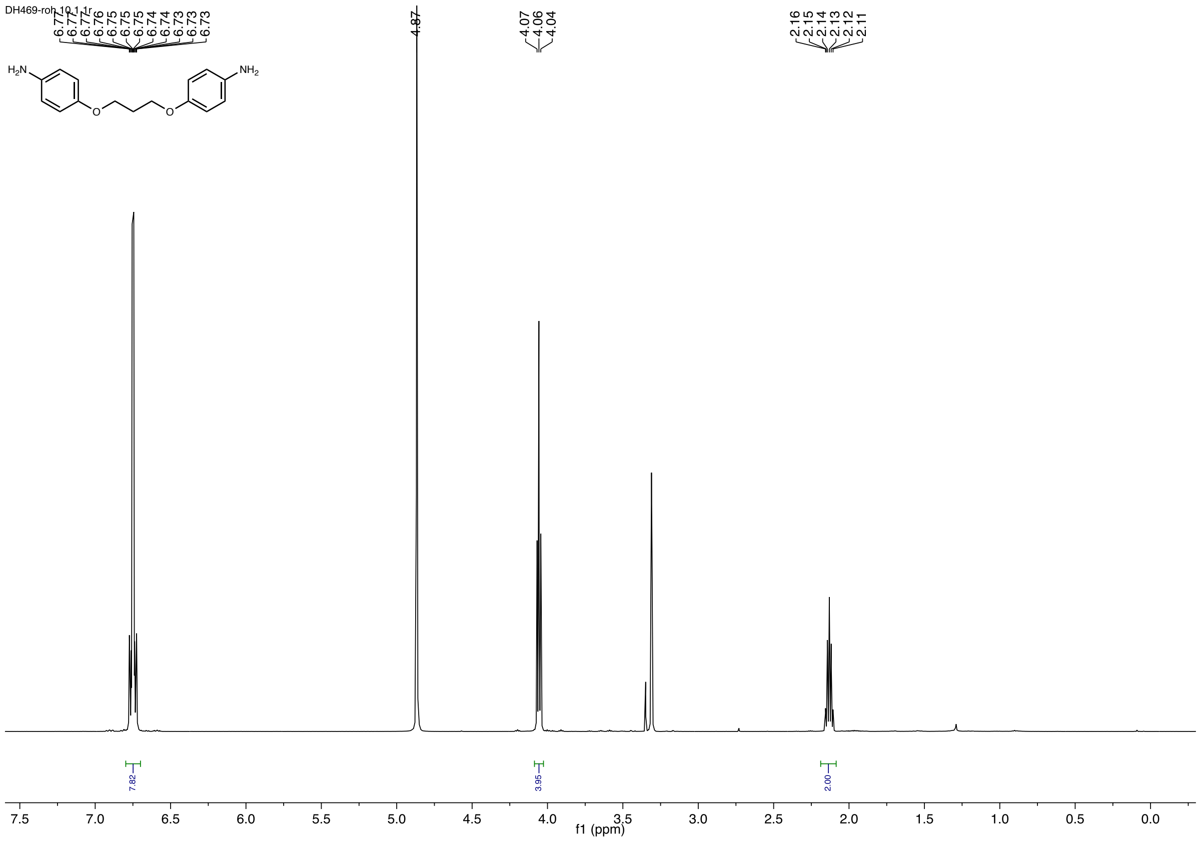

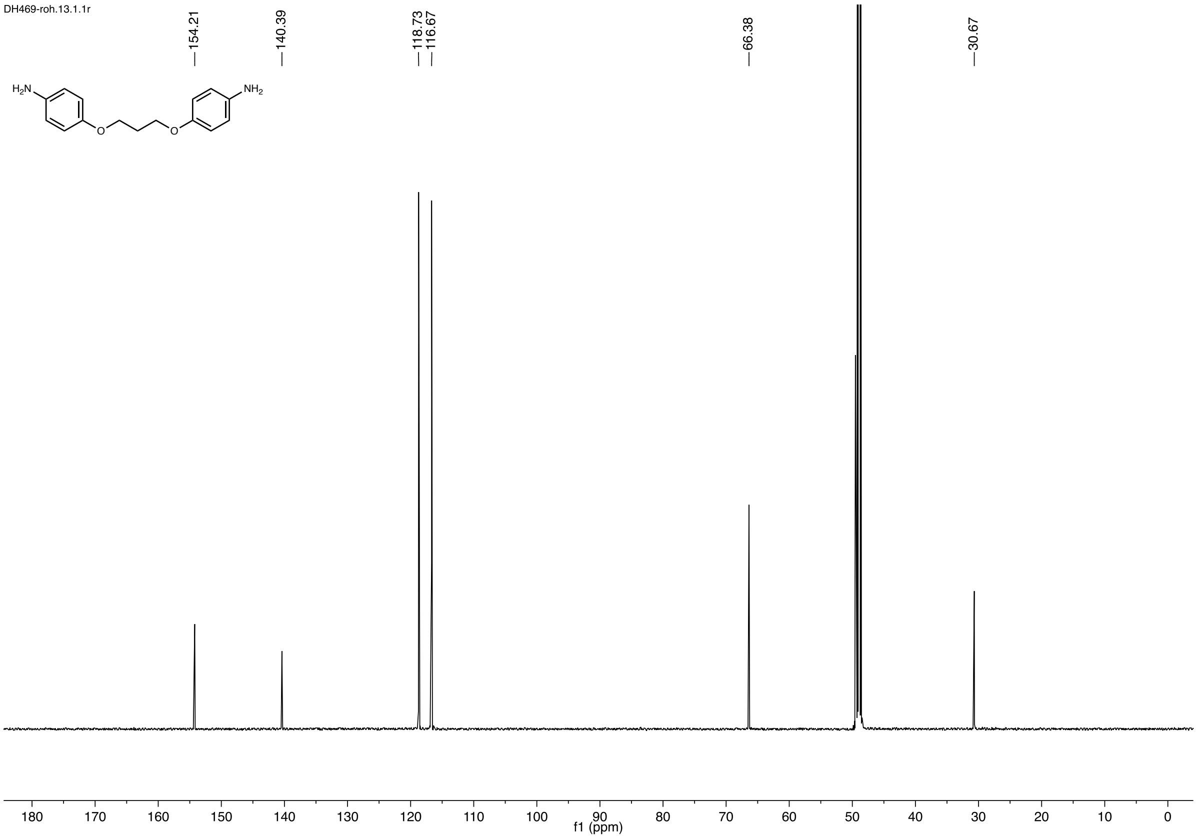

^{1}H and ^{13}C NMR of **E**

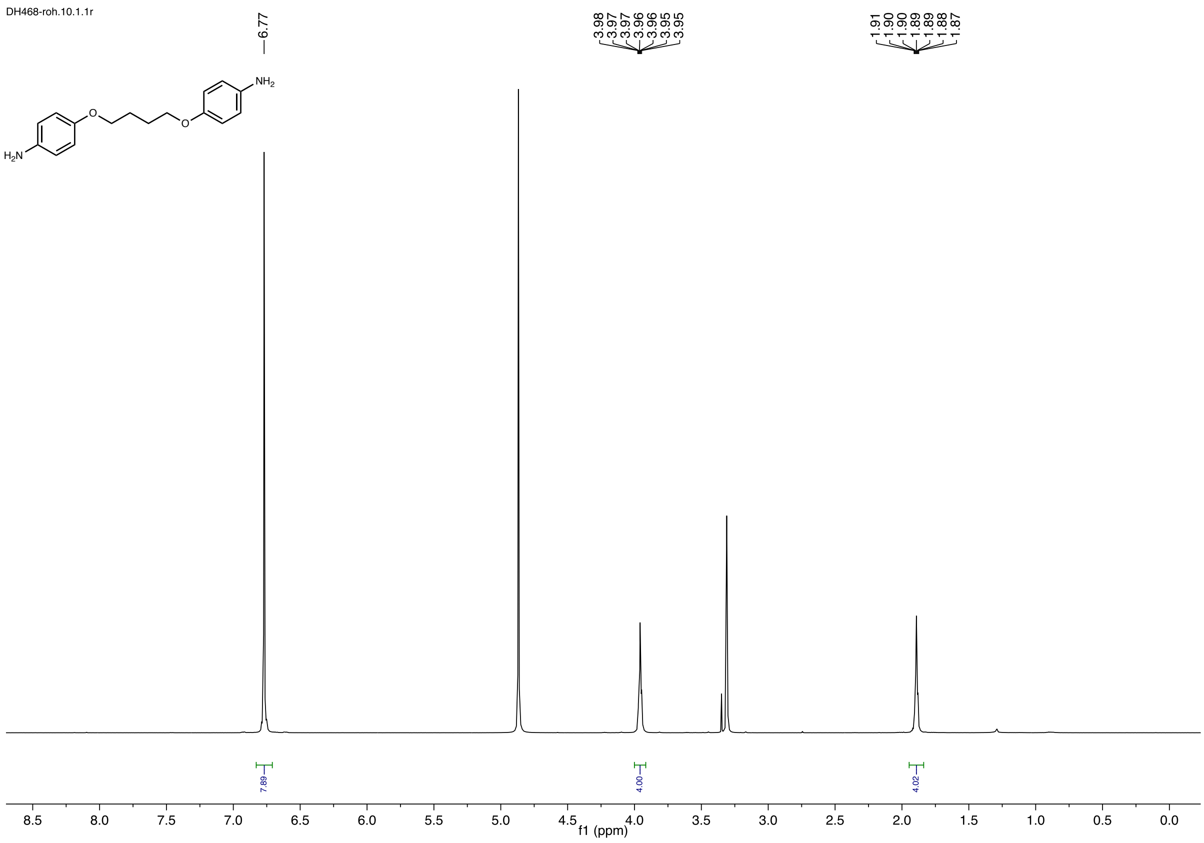

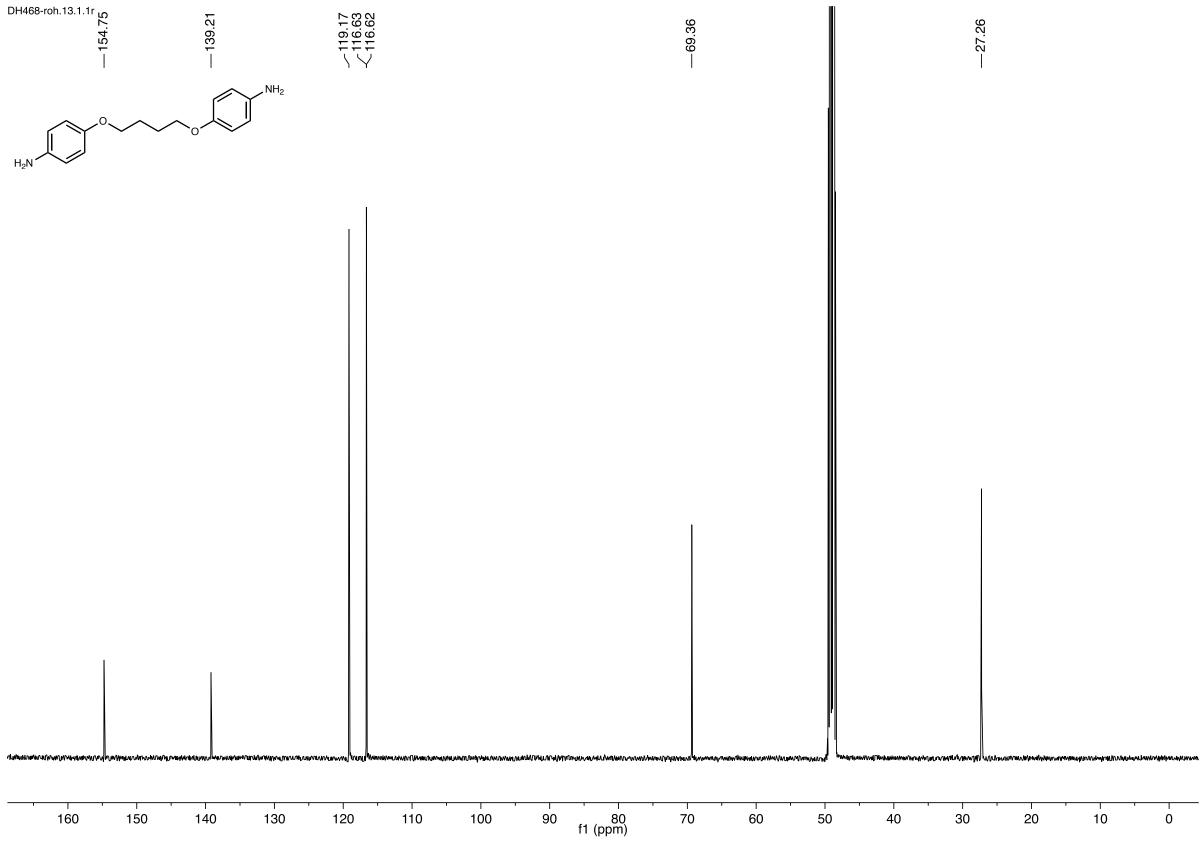

^{1}H and ^{13}C NMR of **F**

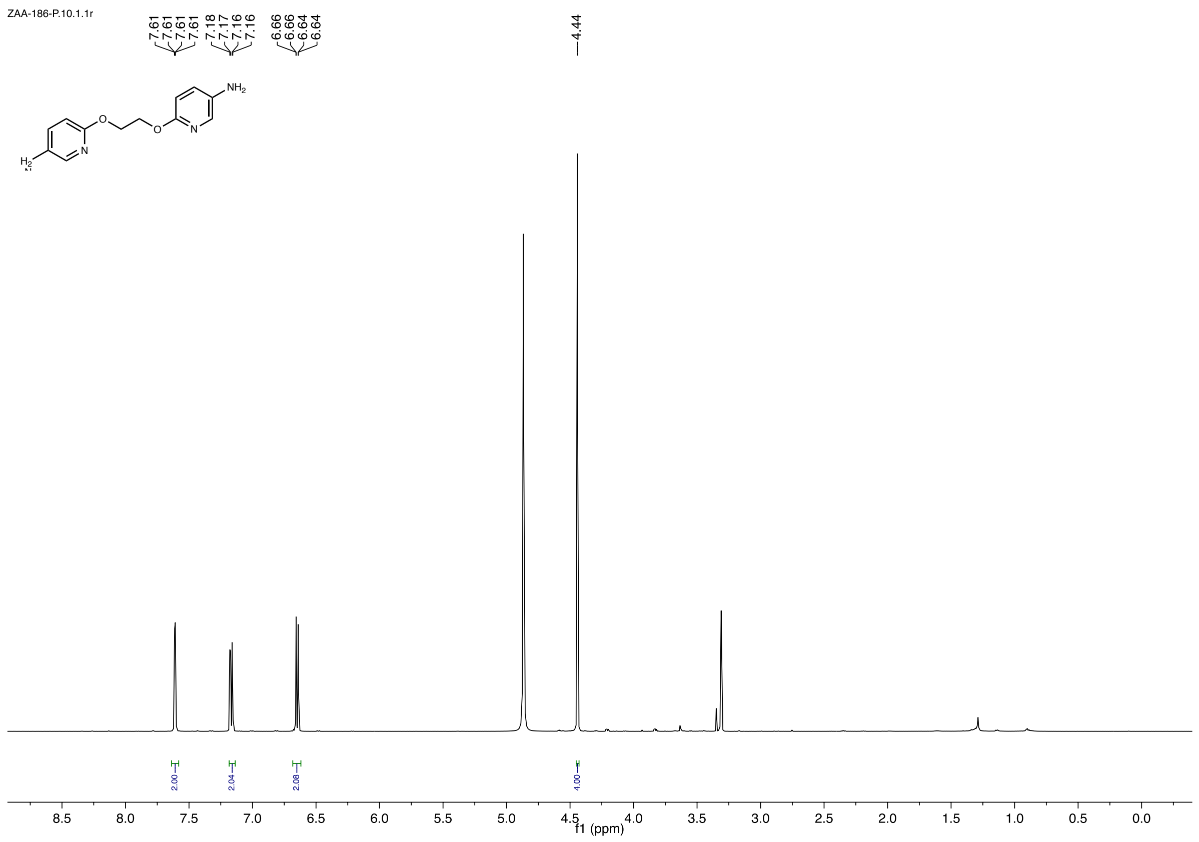

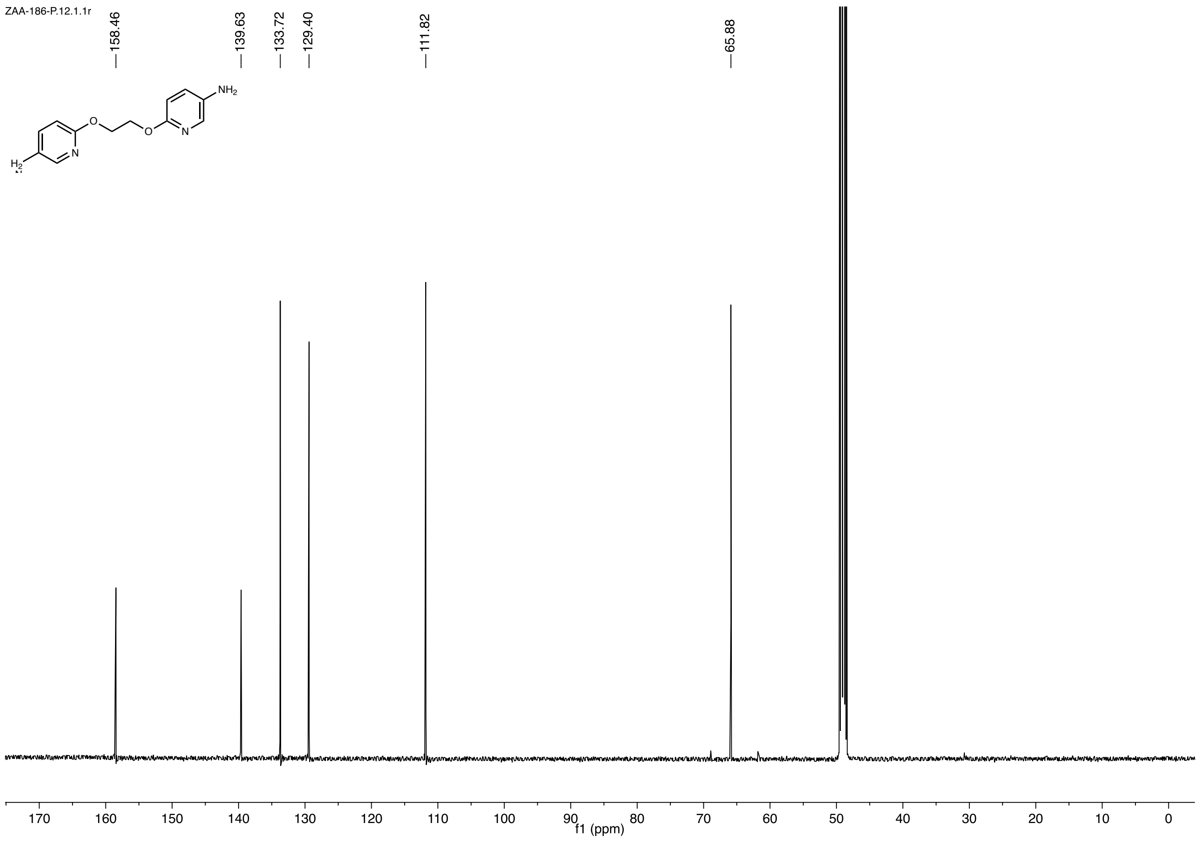

^{1}H and ^{13}C NMR of **H**

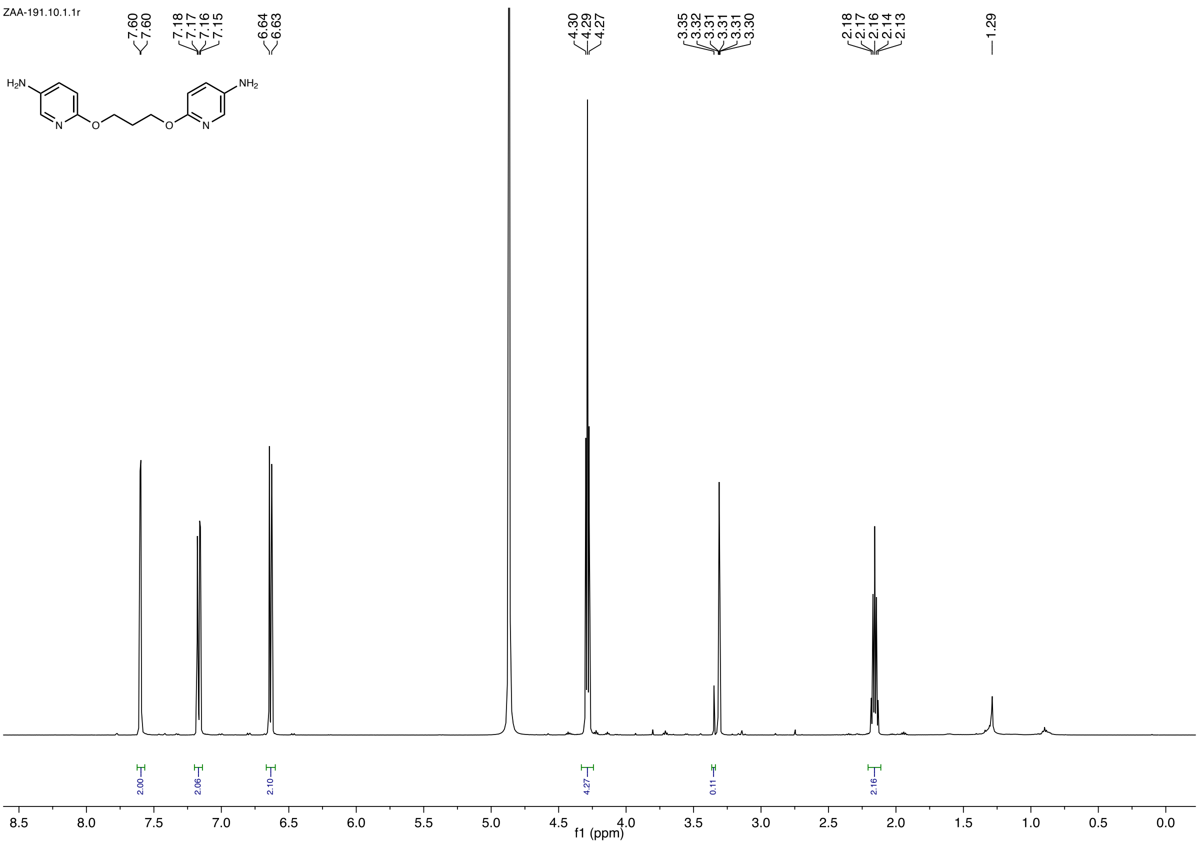

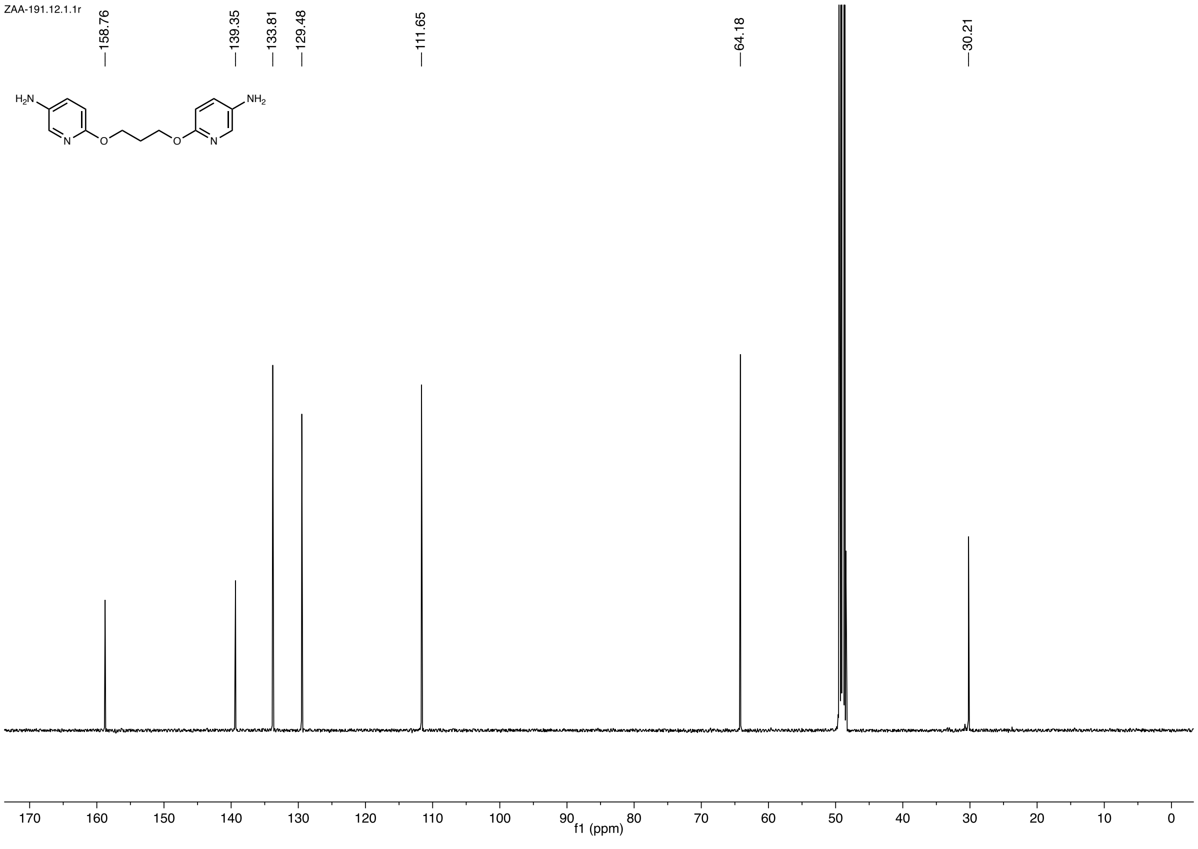

^{1}H and ^{13}C NMR of **I**

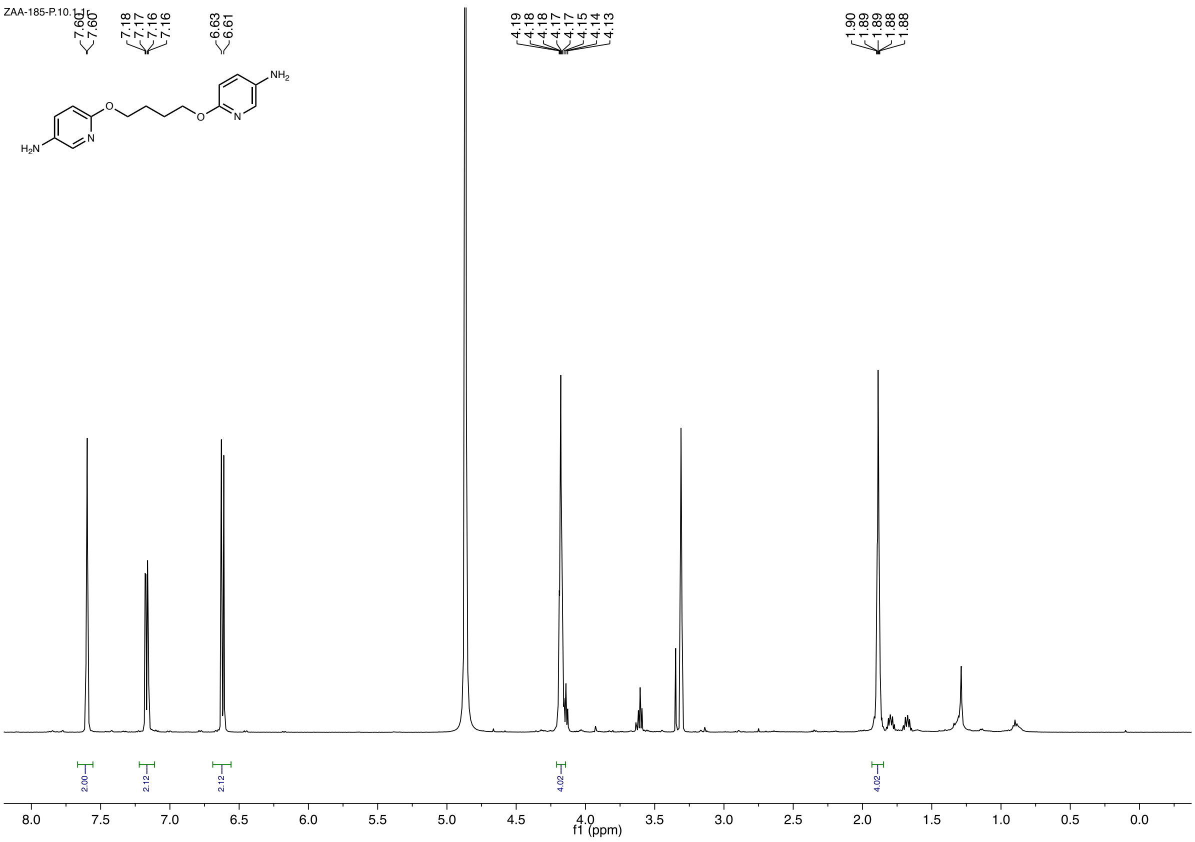

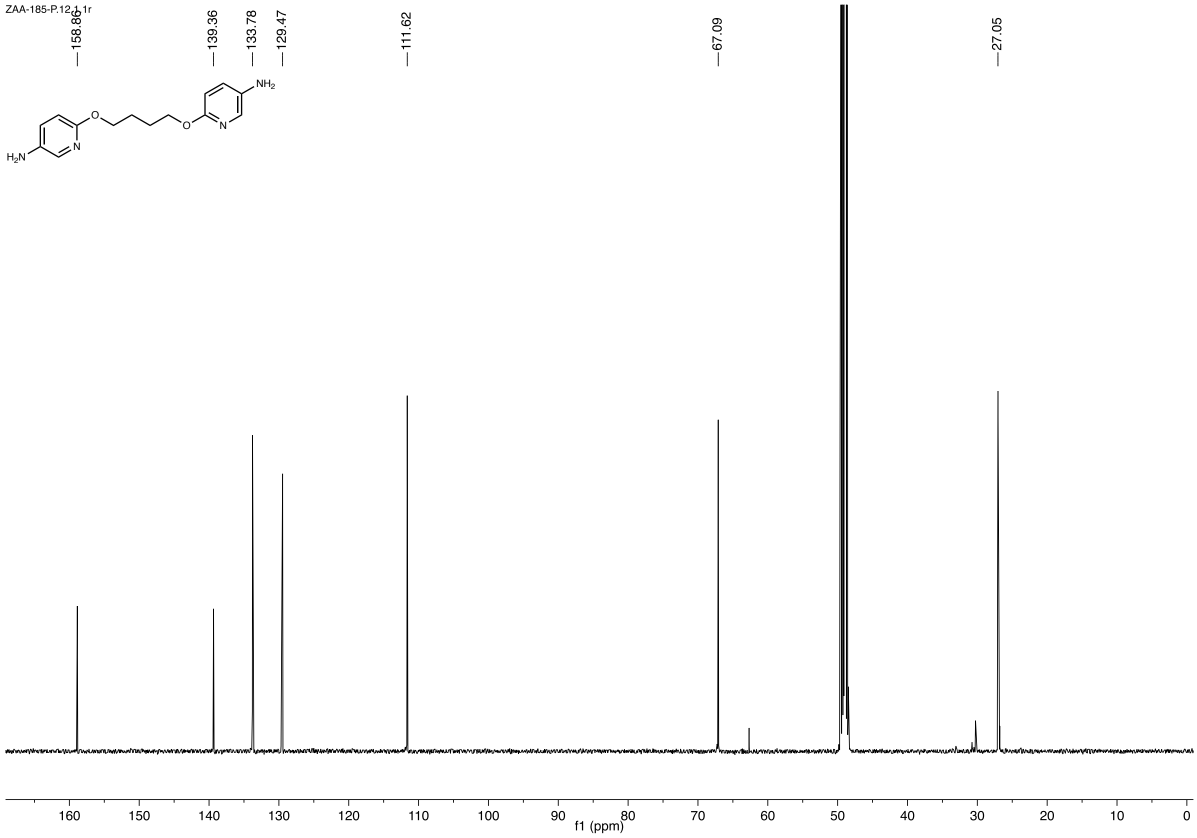

^{1}H and ^{13}C NMR of **J**

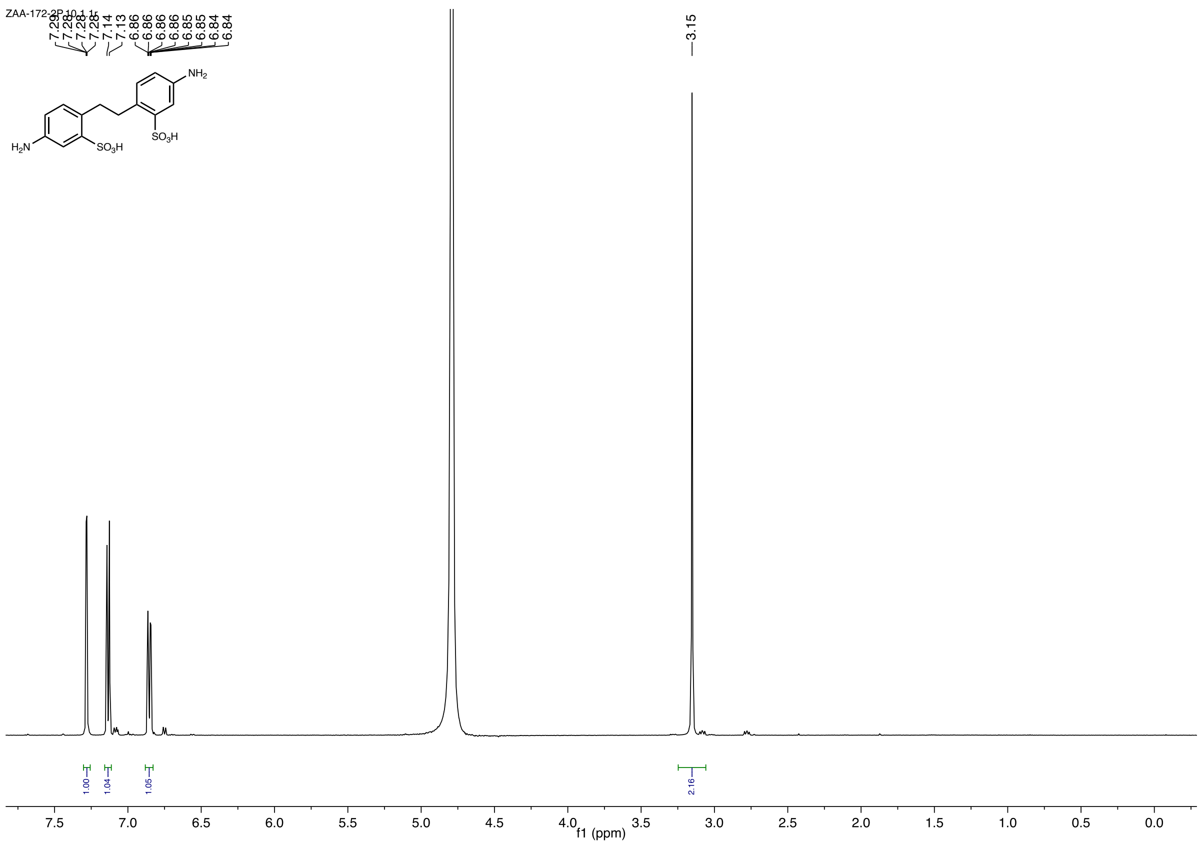

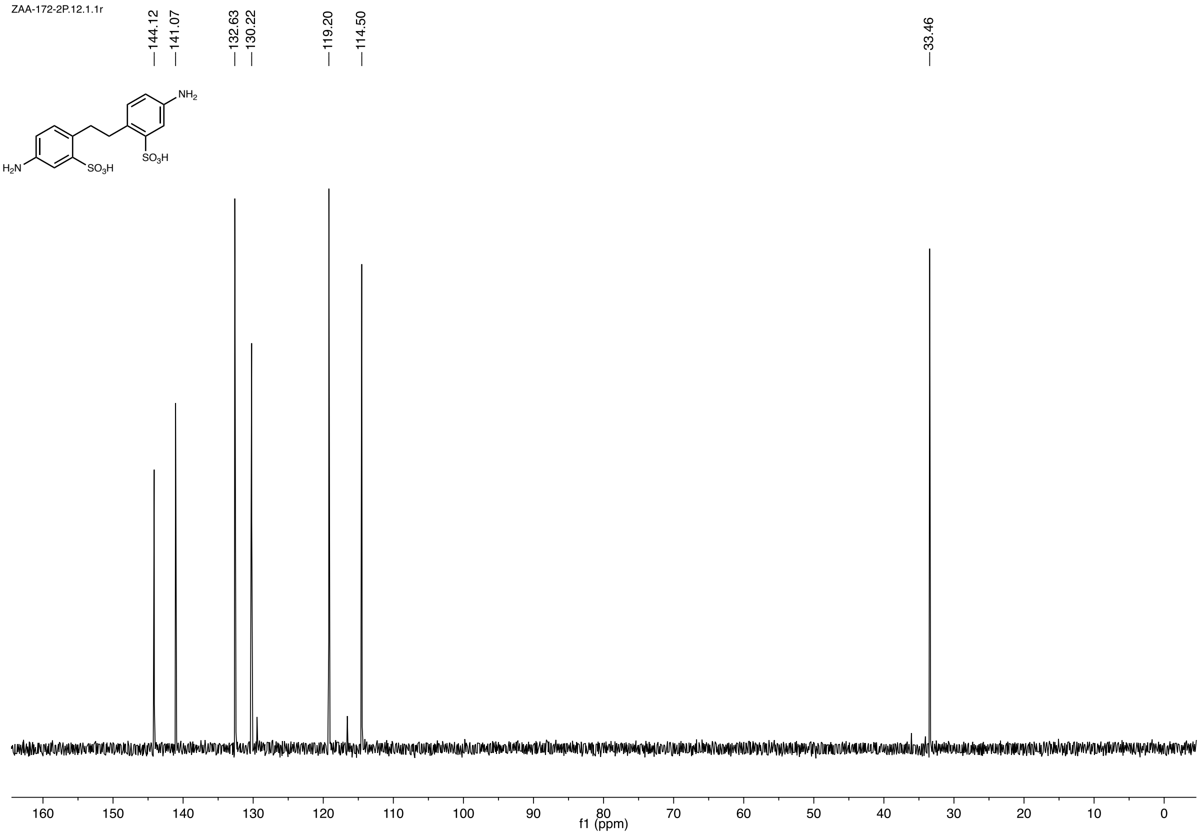

^{1}H and ^{13}C NMR of **L**

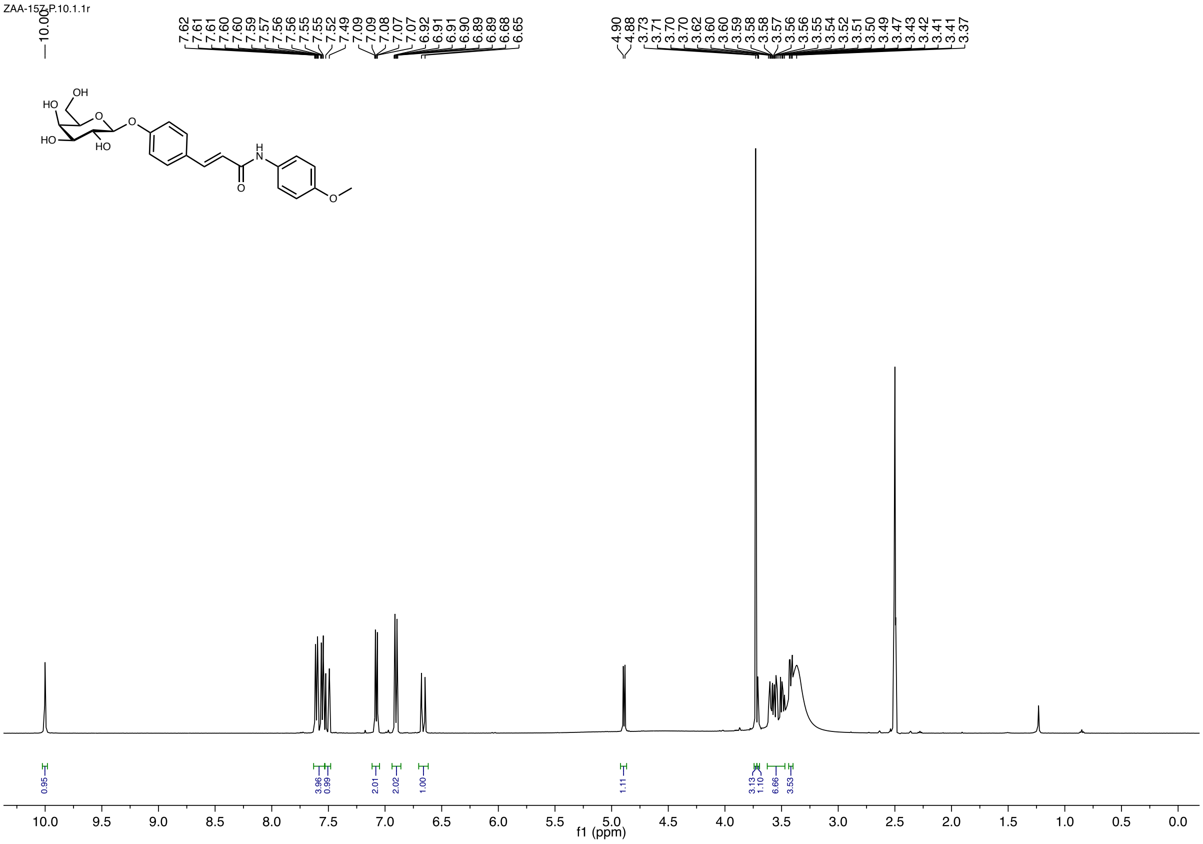

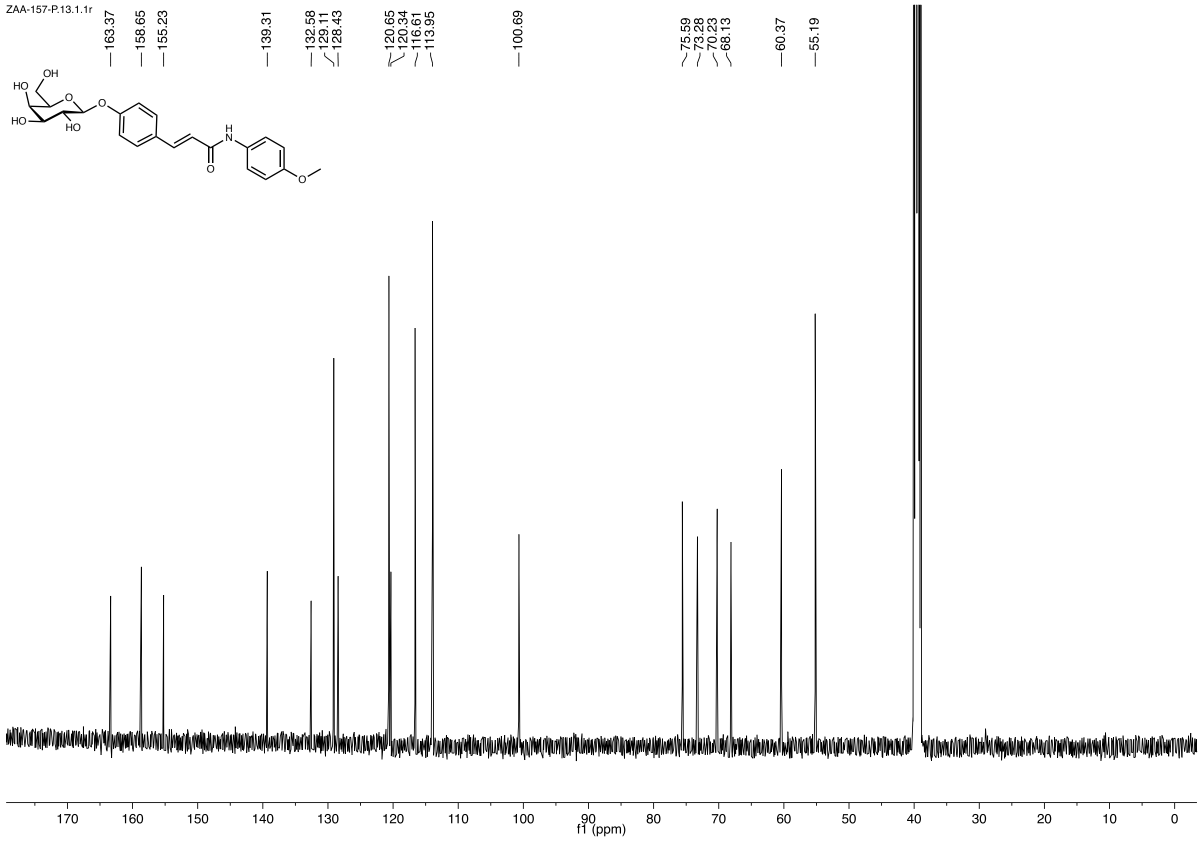

^{1}H and ^{13}C NMR of **A1**

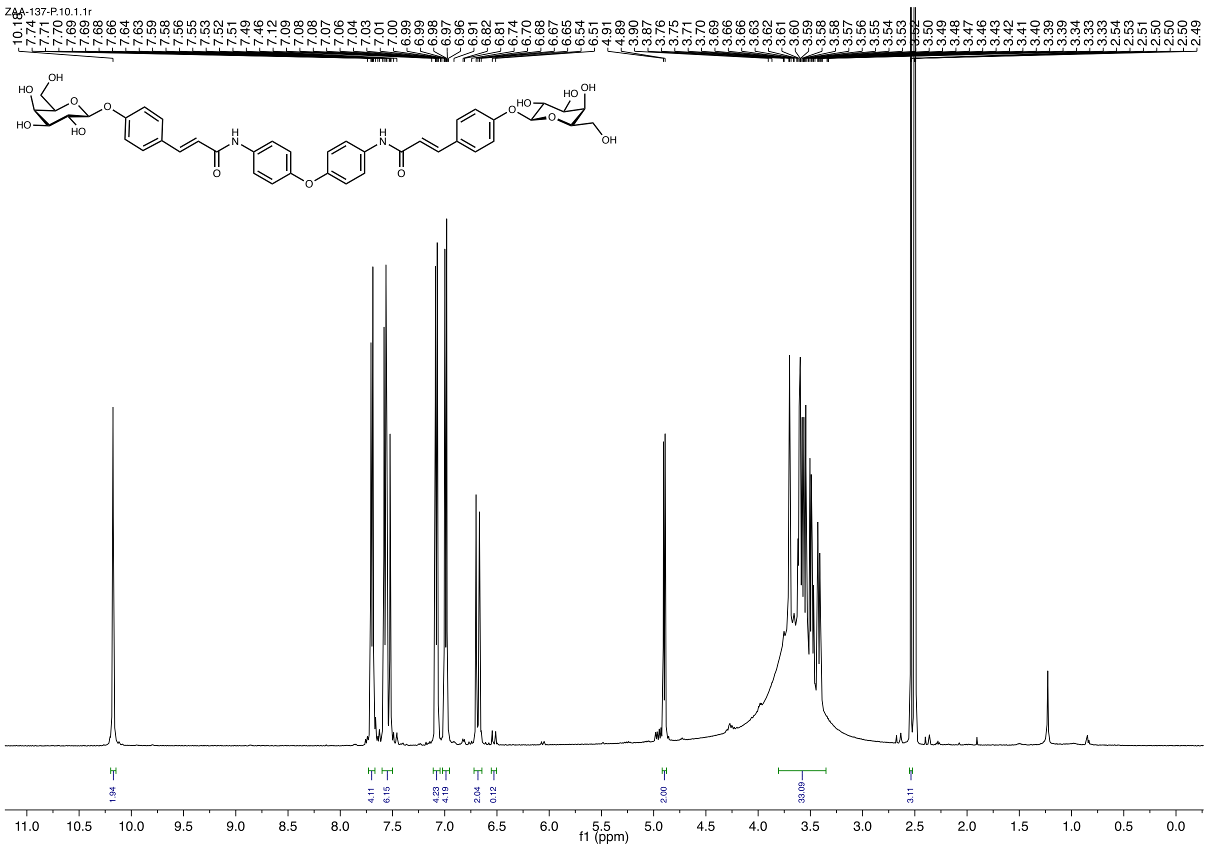

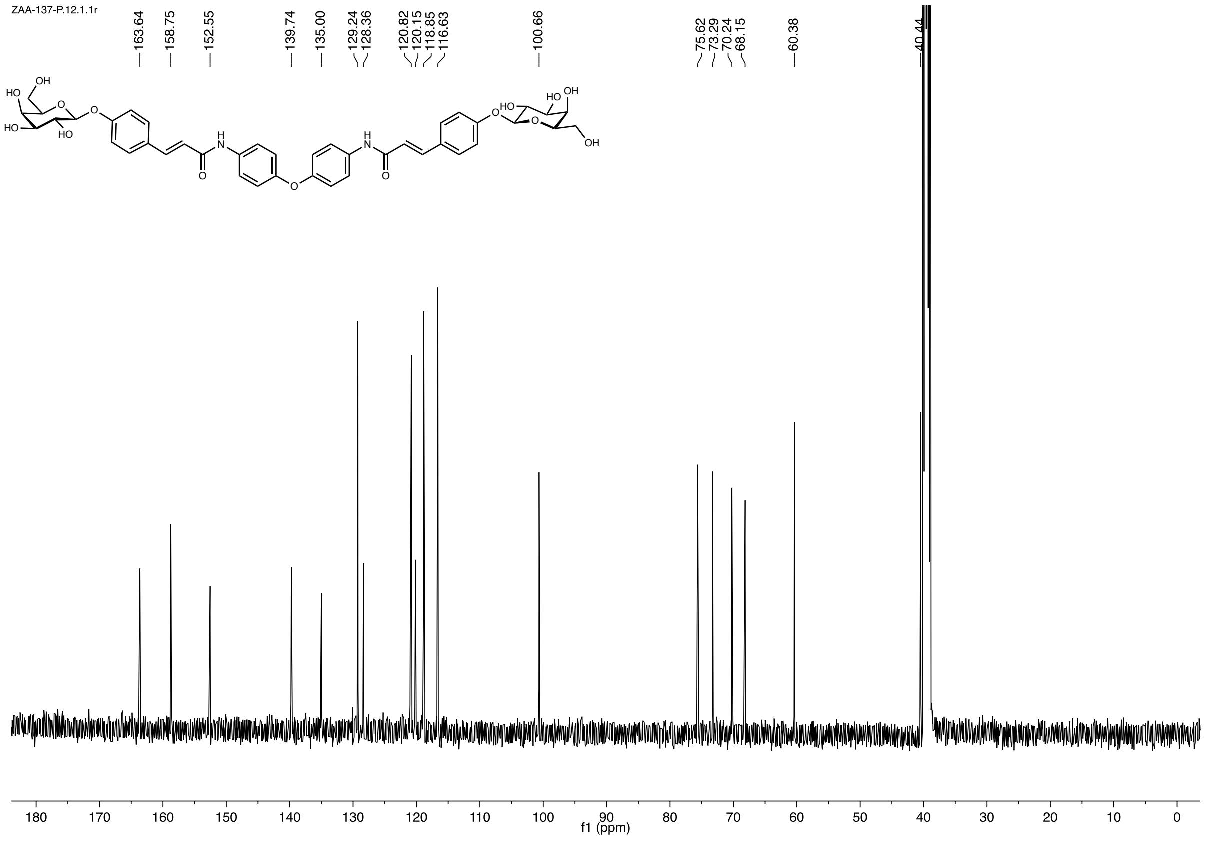

^{1}H and ^{13}C NMR of **B1**

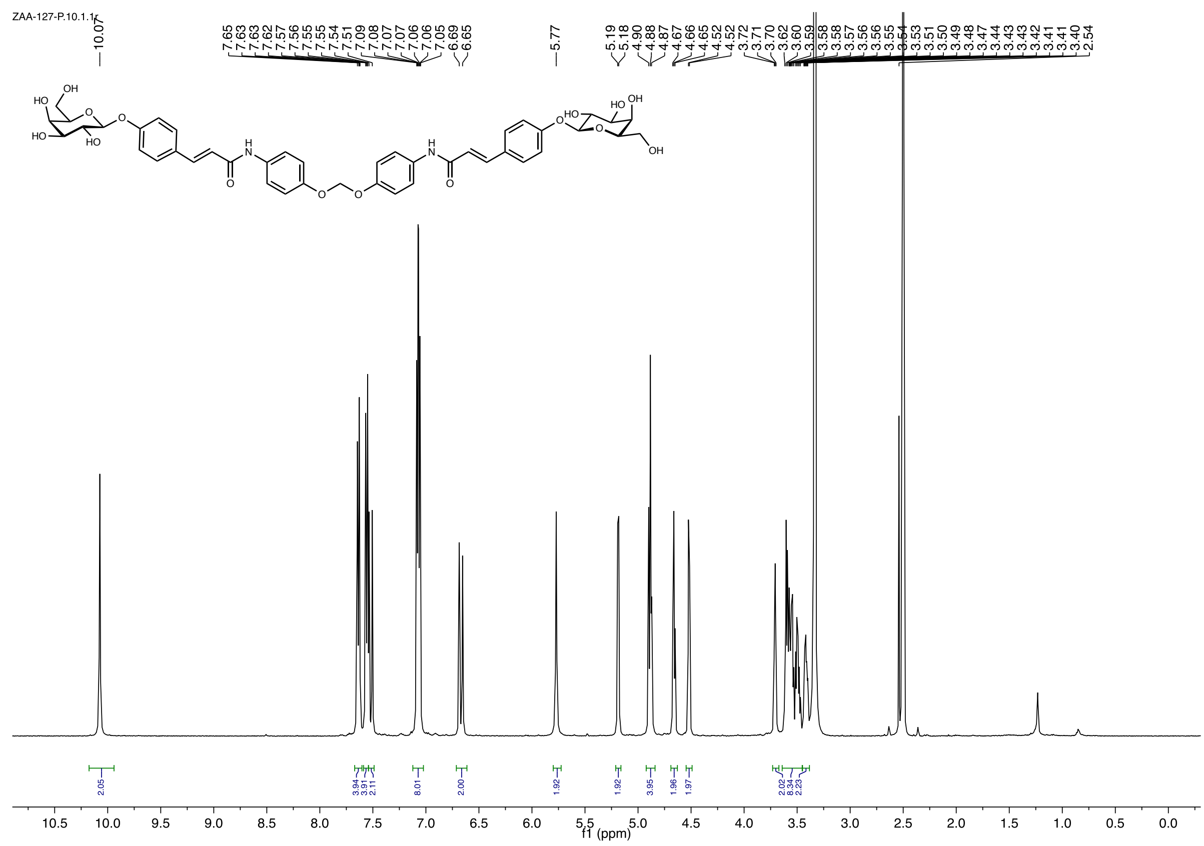

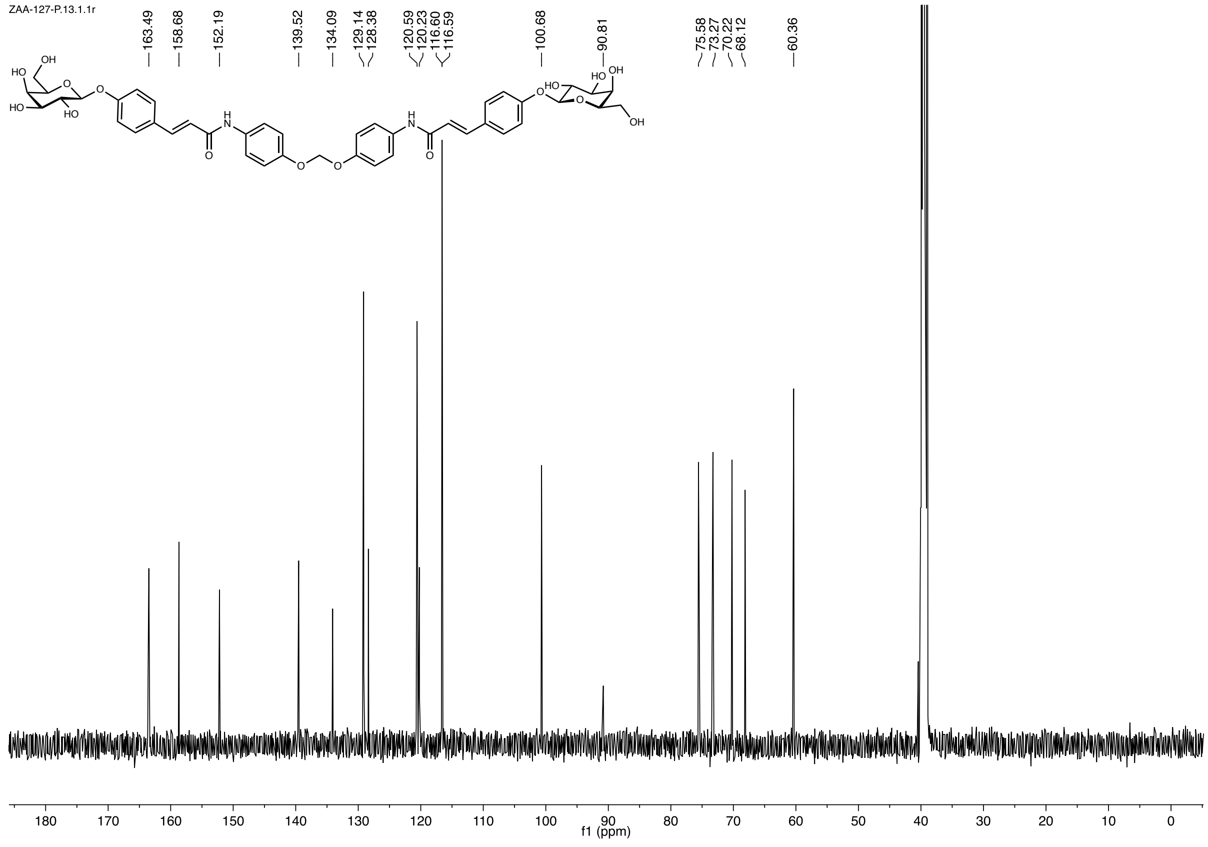

^{1}H and ^{13}C NMR of **C1**

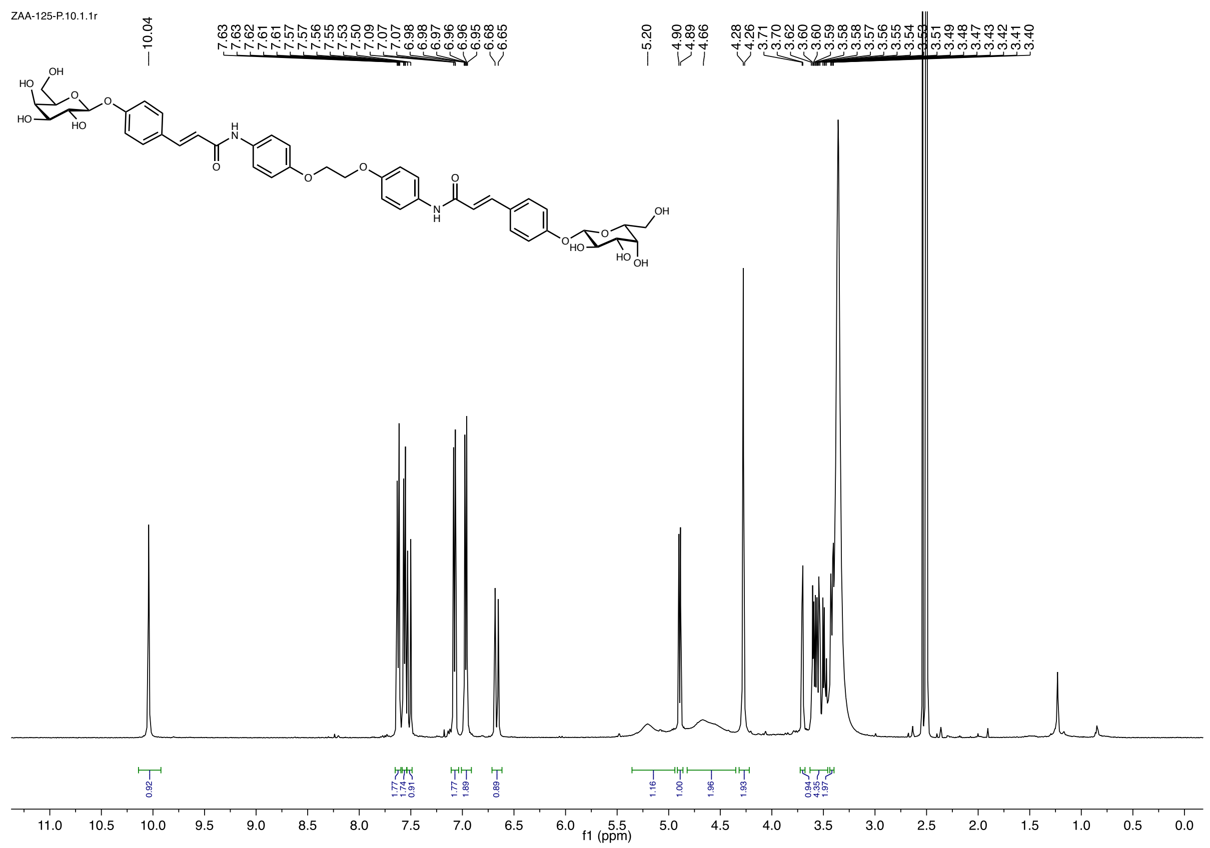

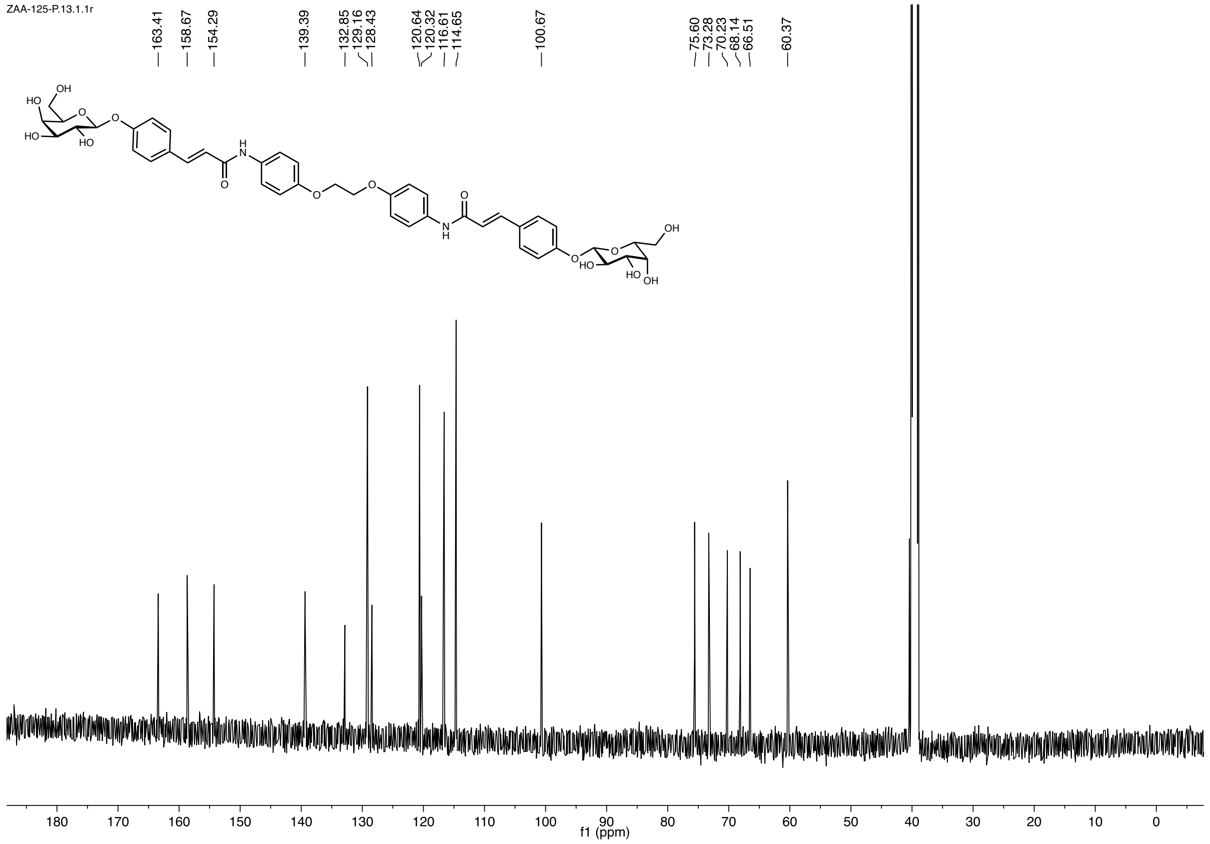

^{1}H and ^{13}C NMR of **D1**

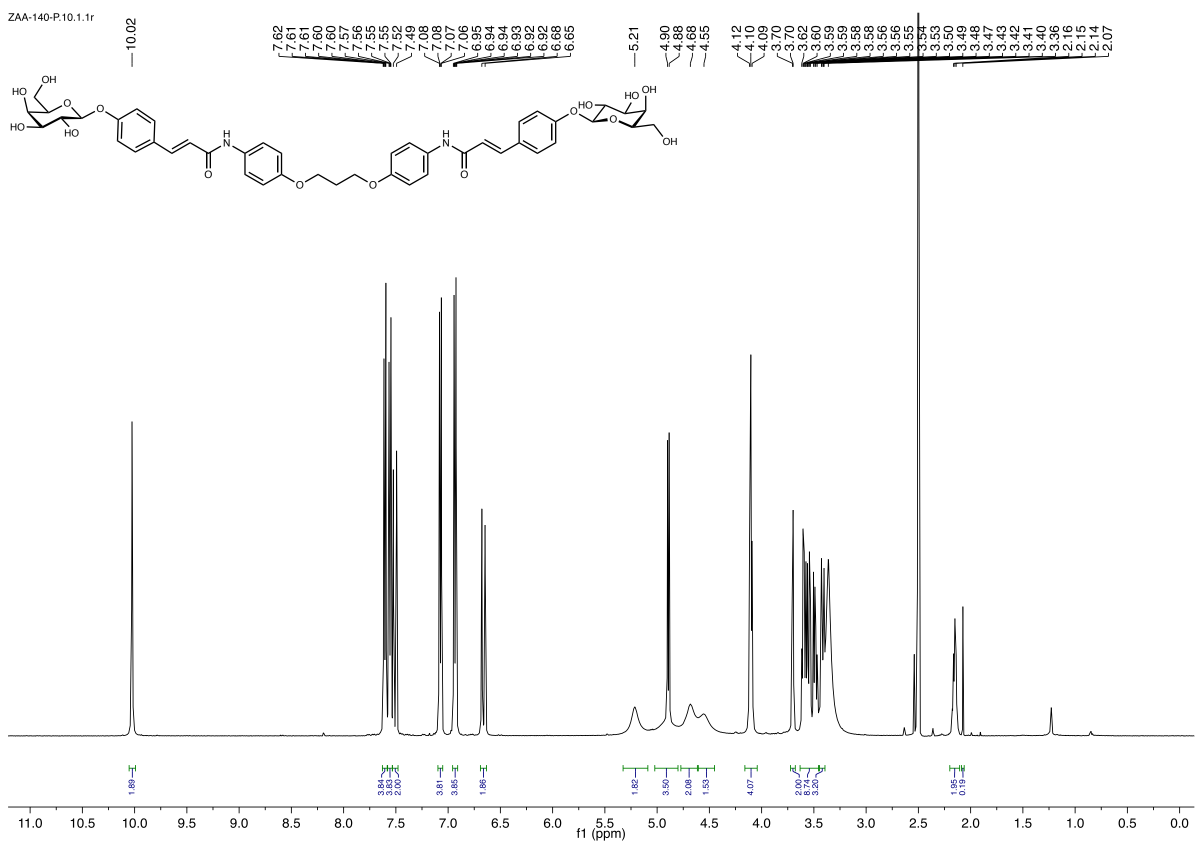

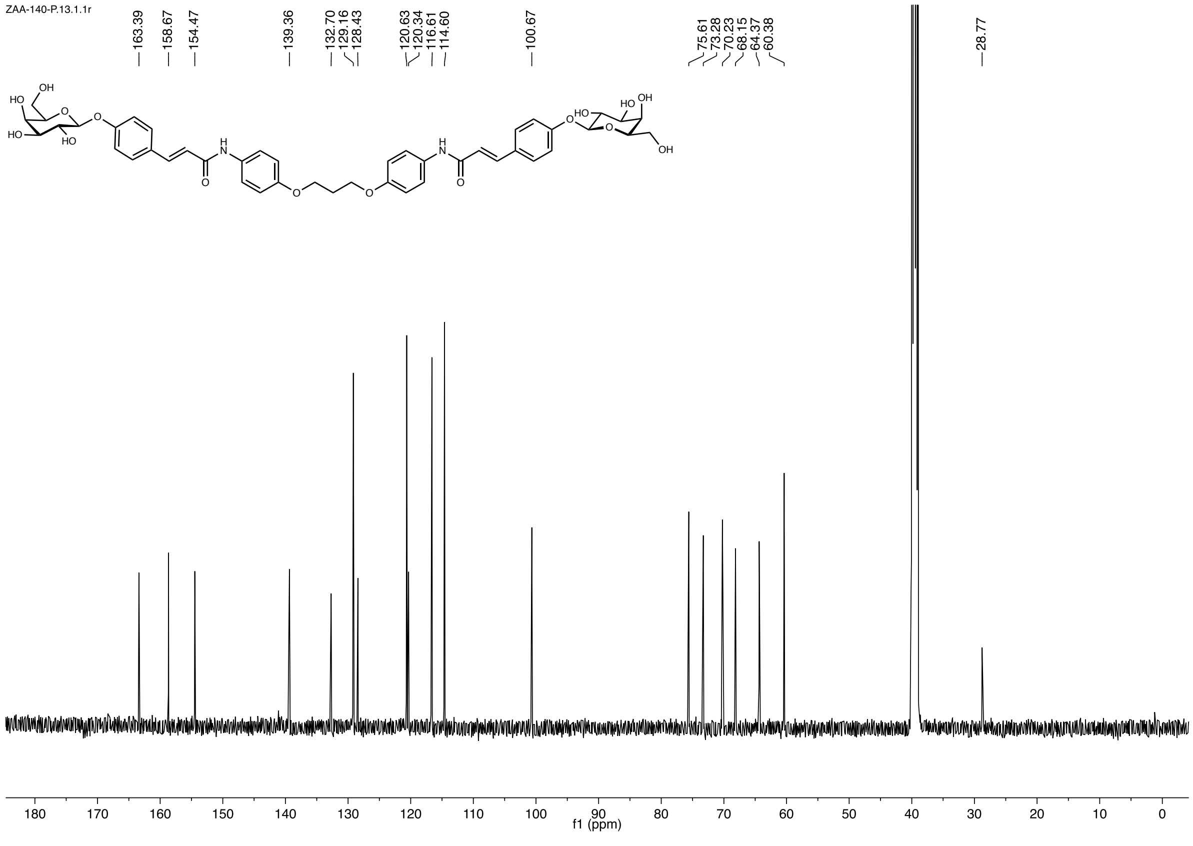

^{1}H and ^{13}C NMR of **E1**

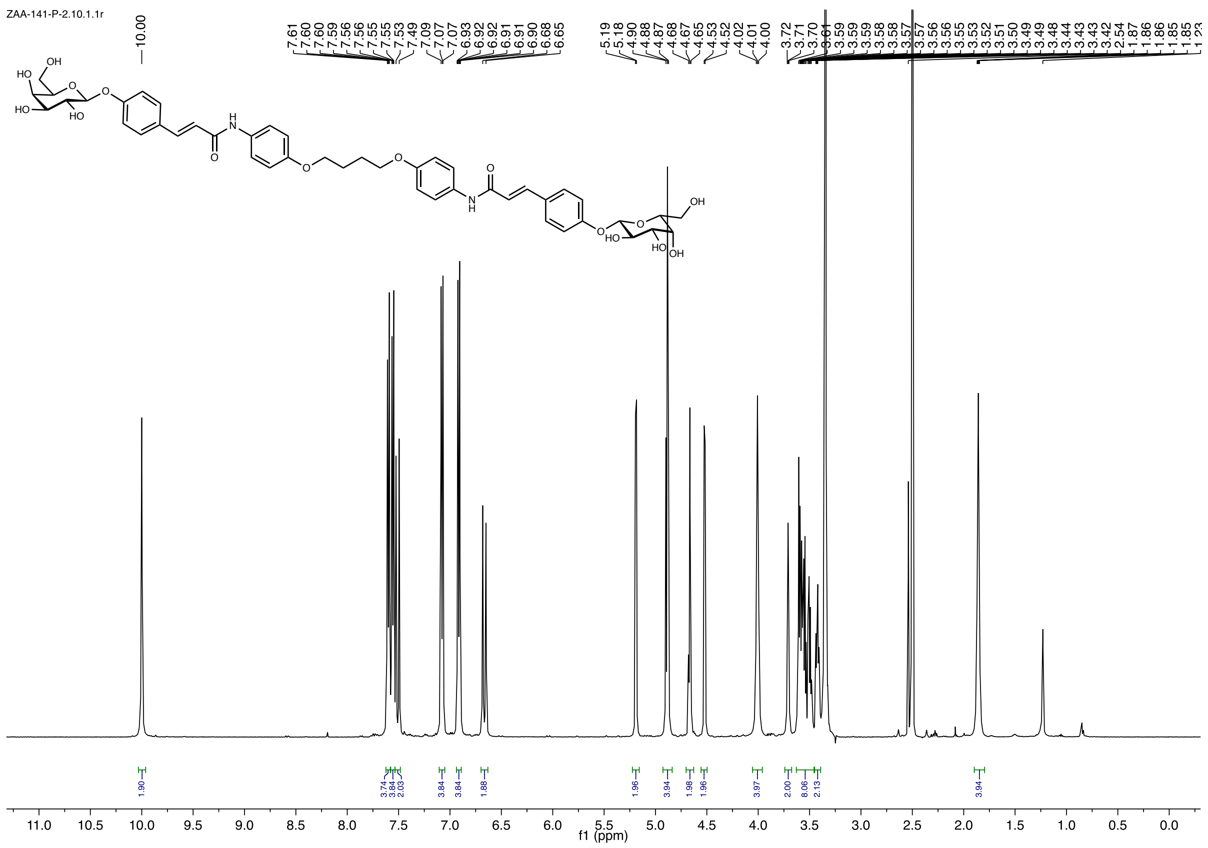

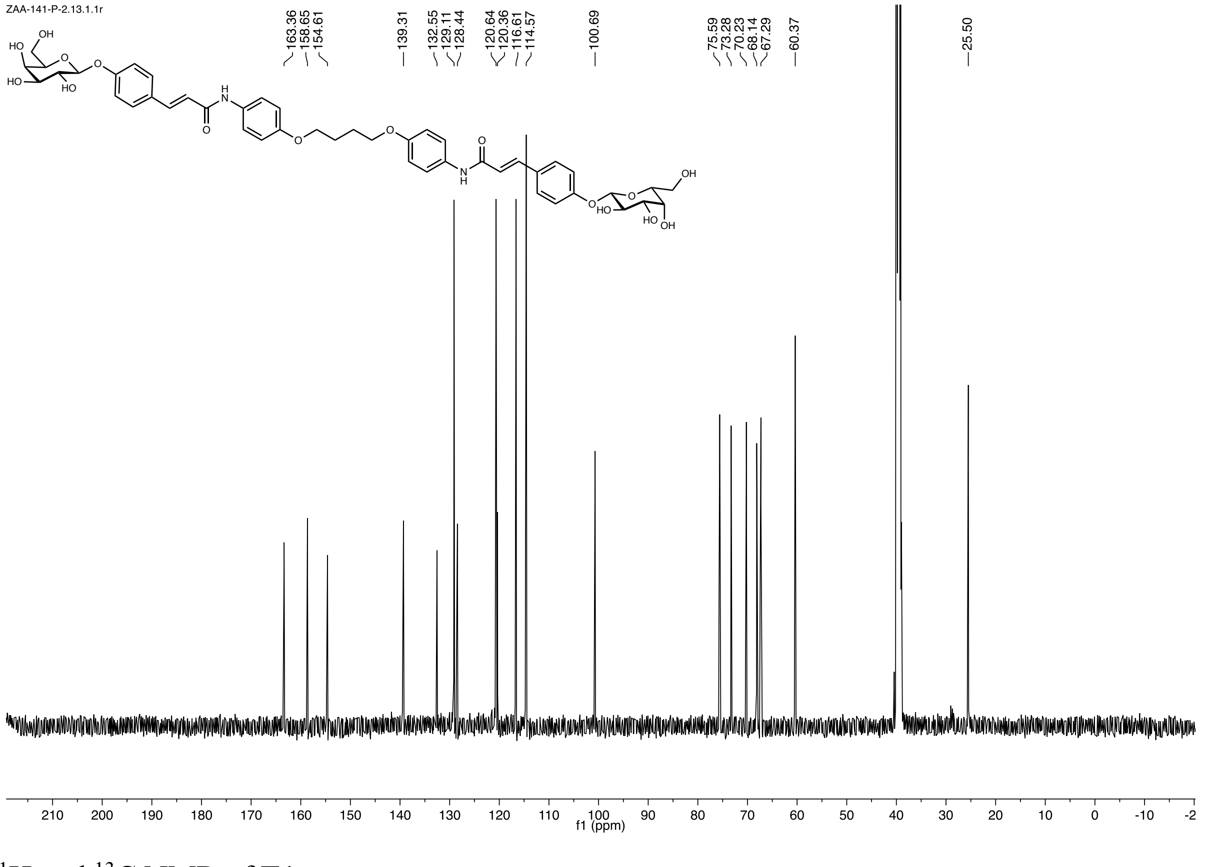

^{1}H and ^{13}C NMR of **F1**

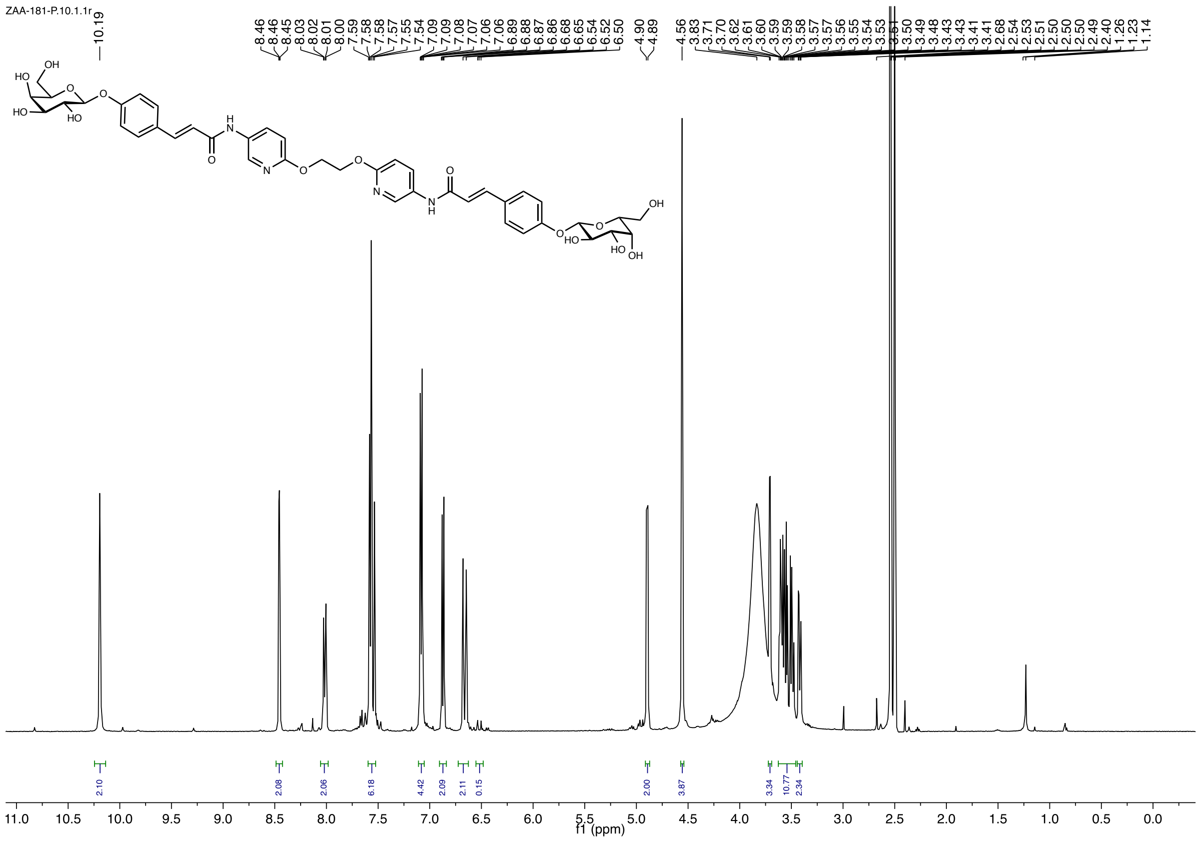

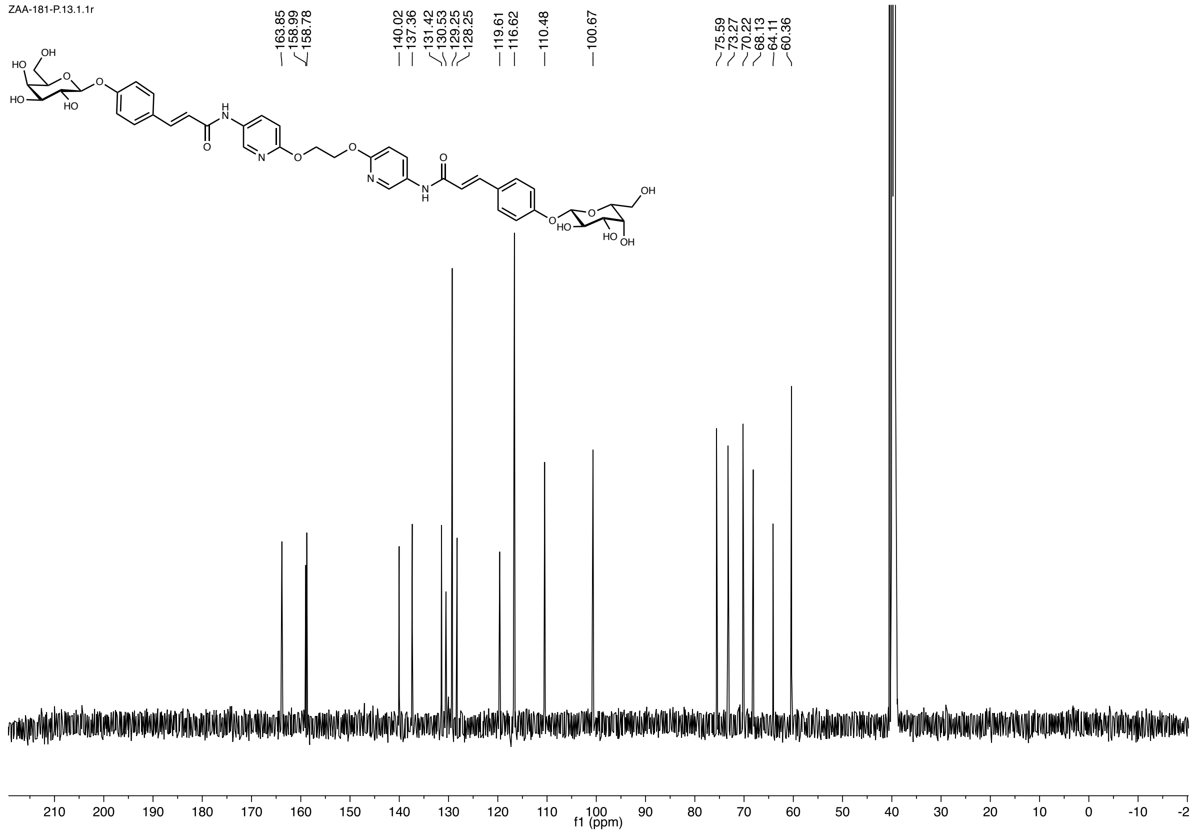

^{1}H and ^{13}C NMR of **H1**

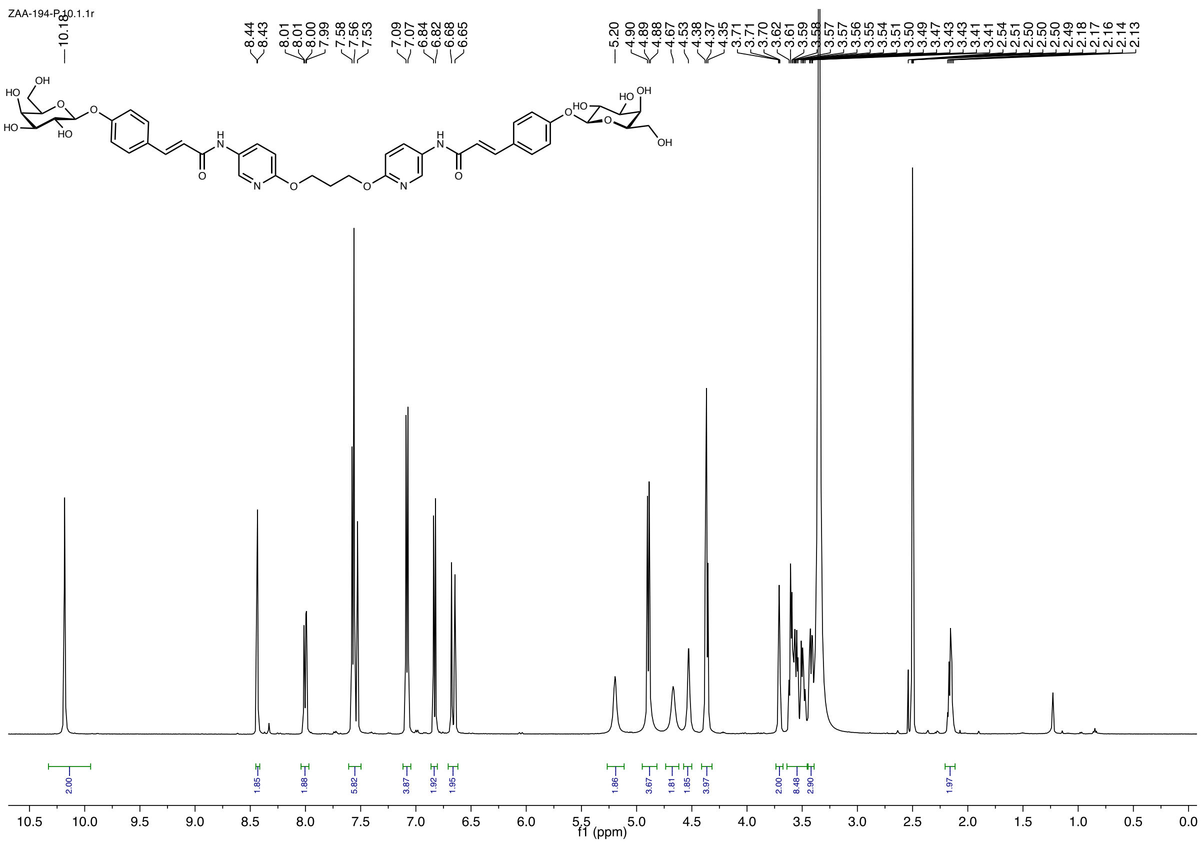

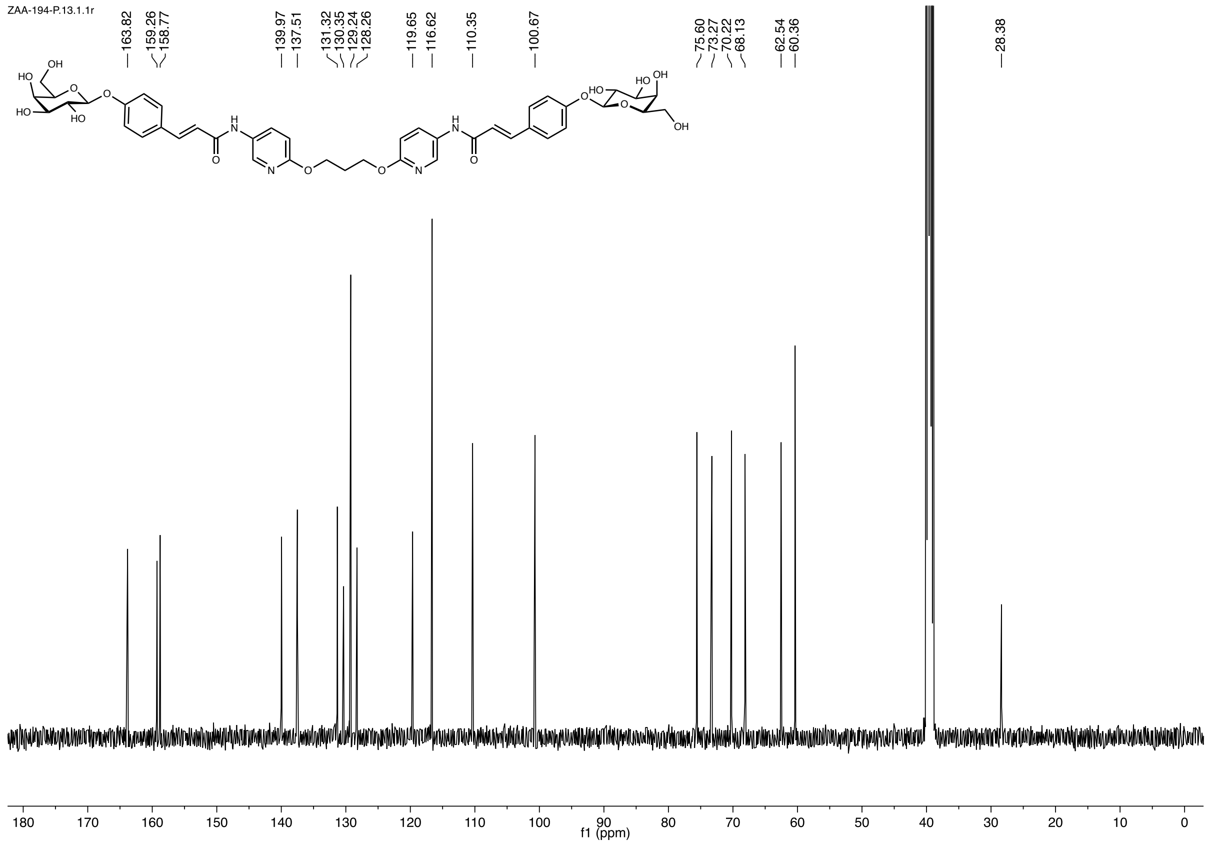

^{1}H and ^{13}C NMR of **I1**

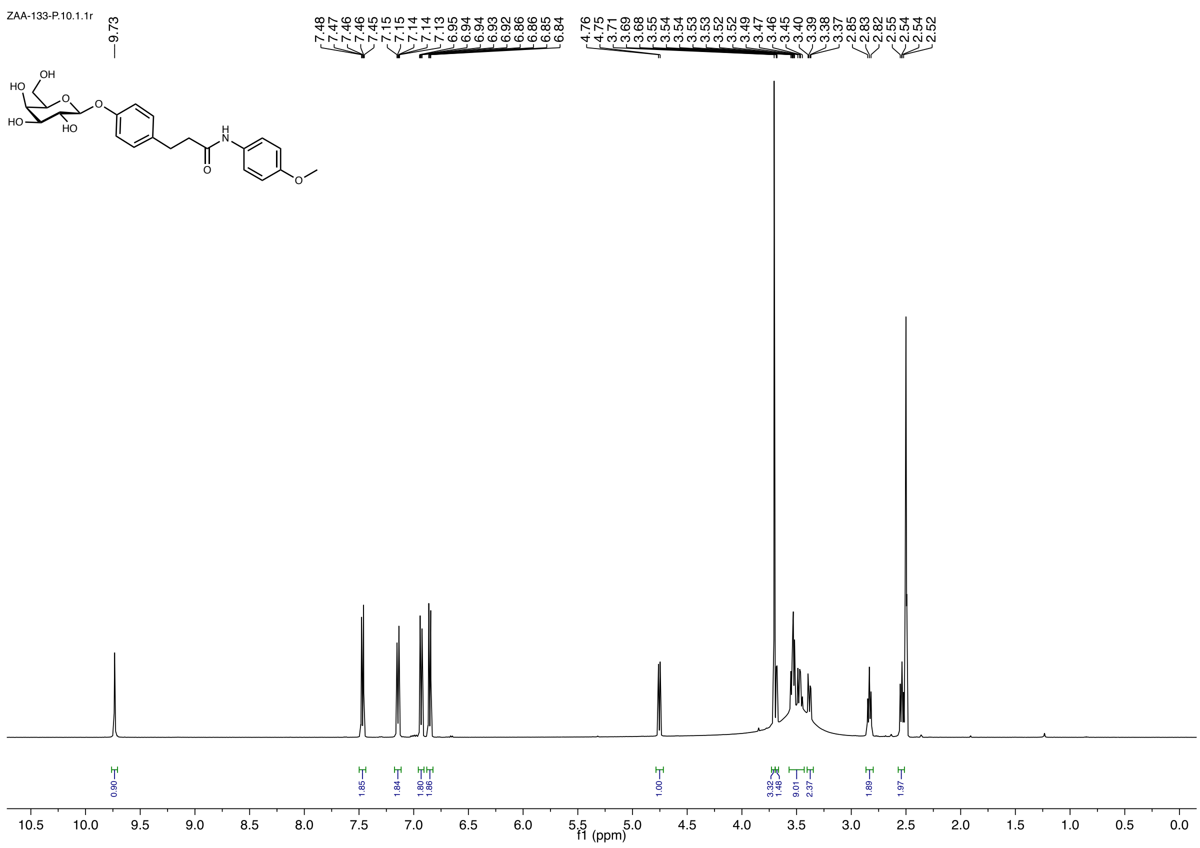

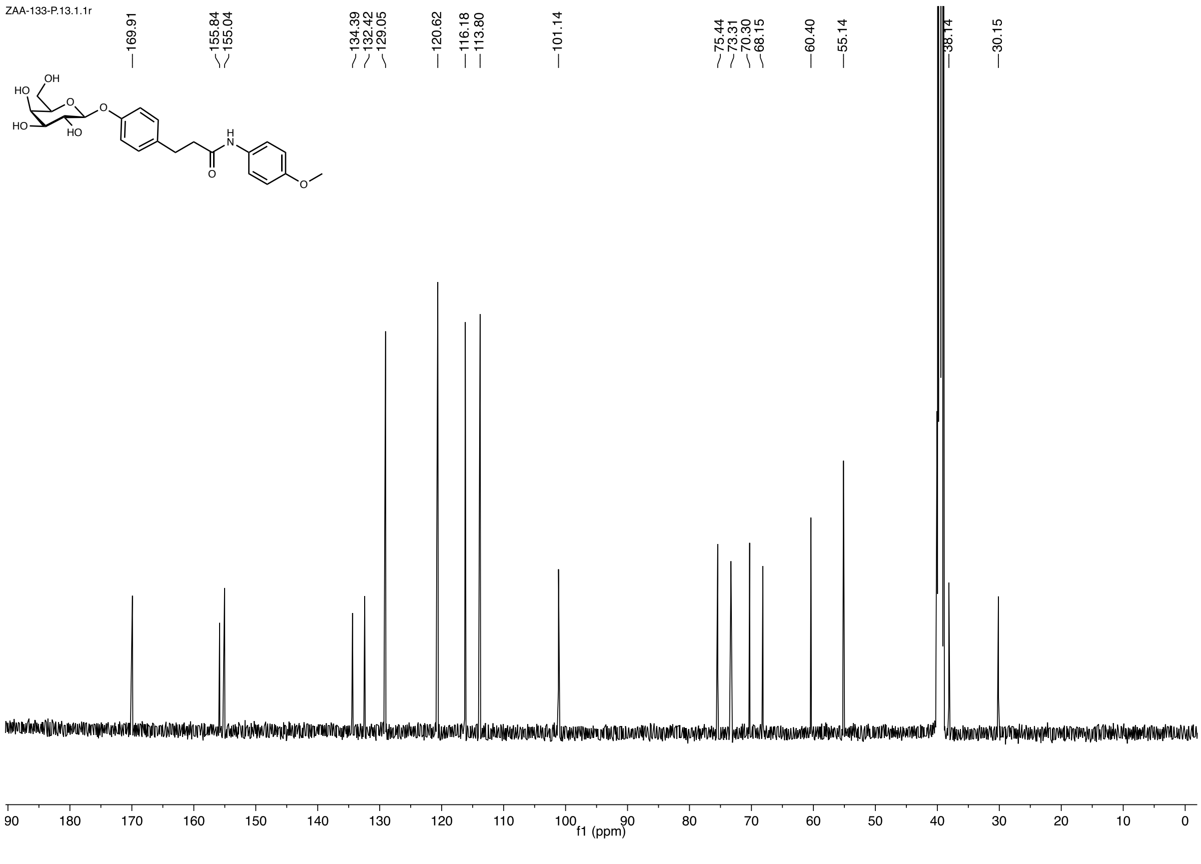

^{1}H and ^{13}C NMR of **A2**

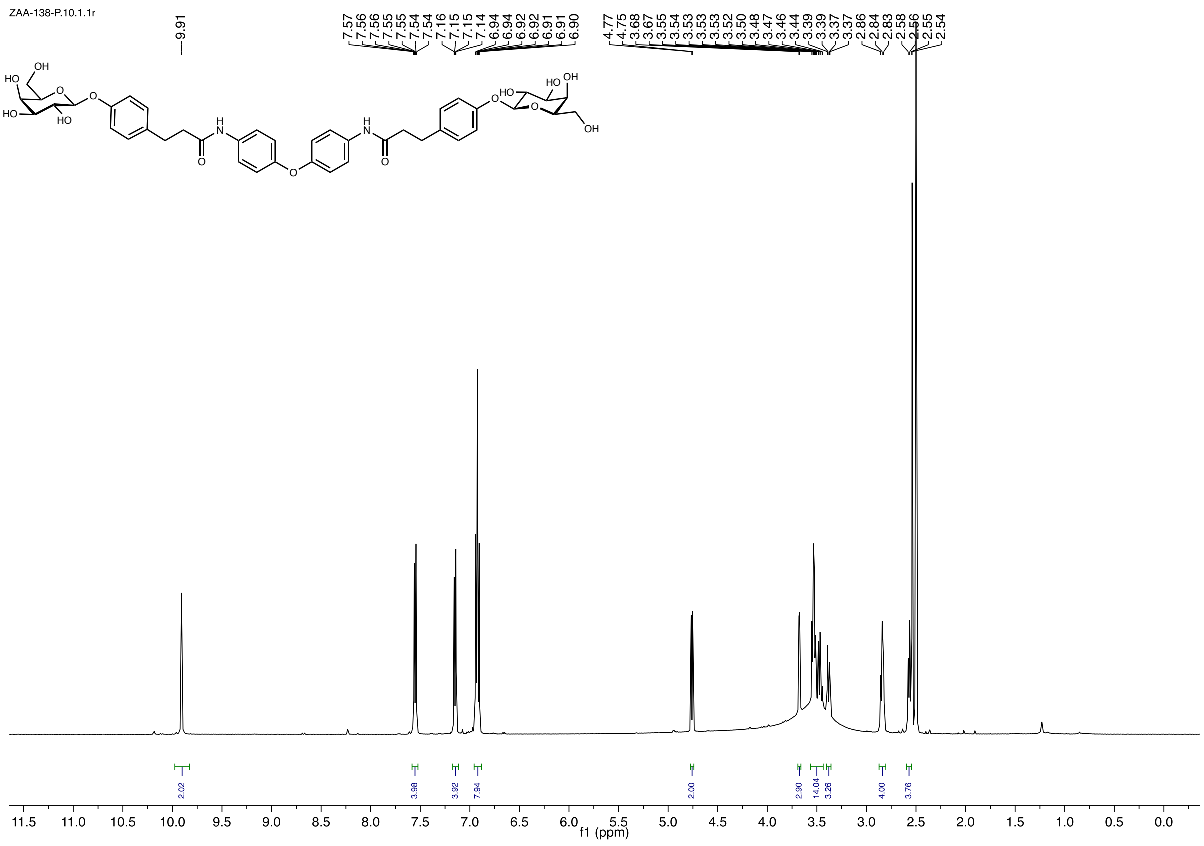

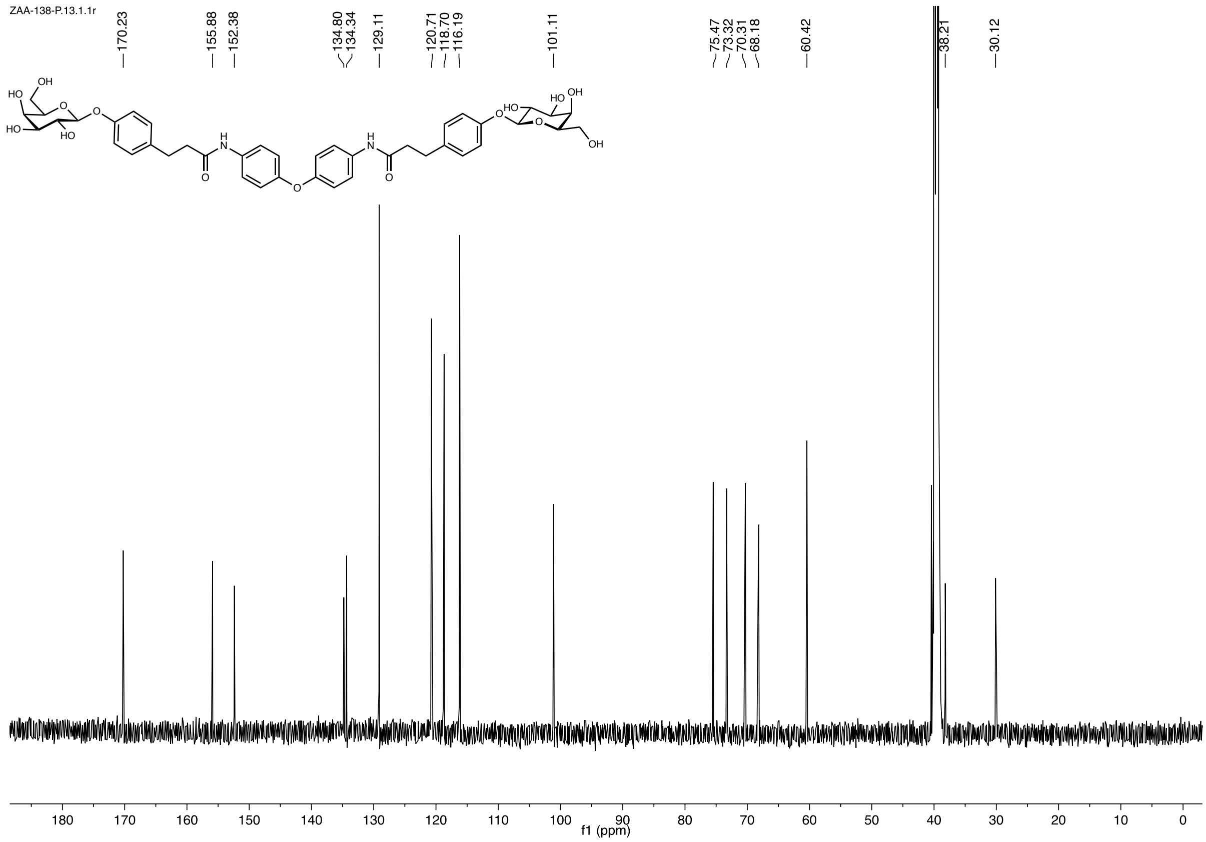

^{1}H and ^{13}C NMR of **B2**

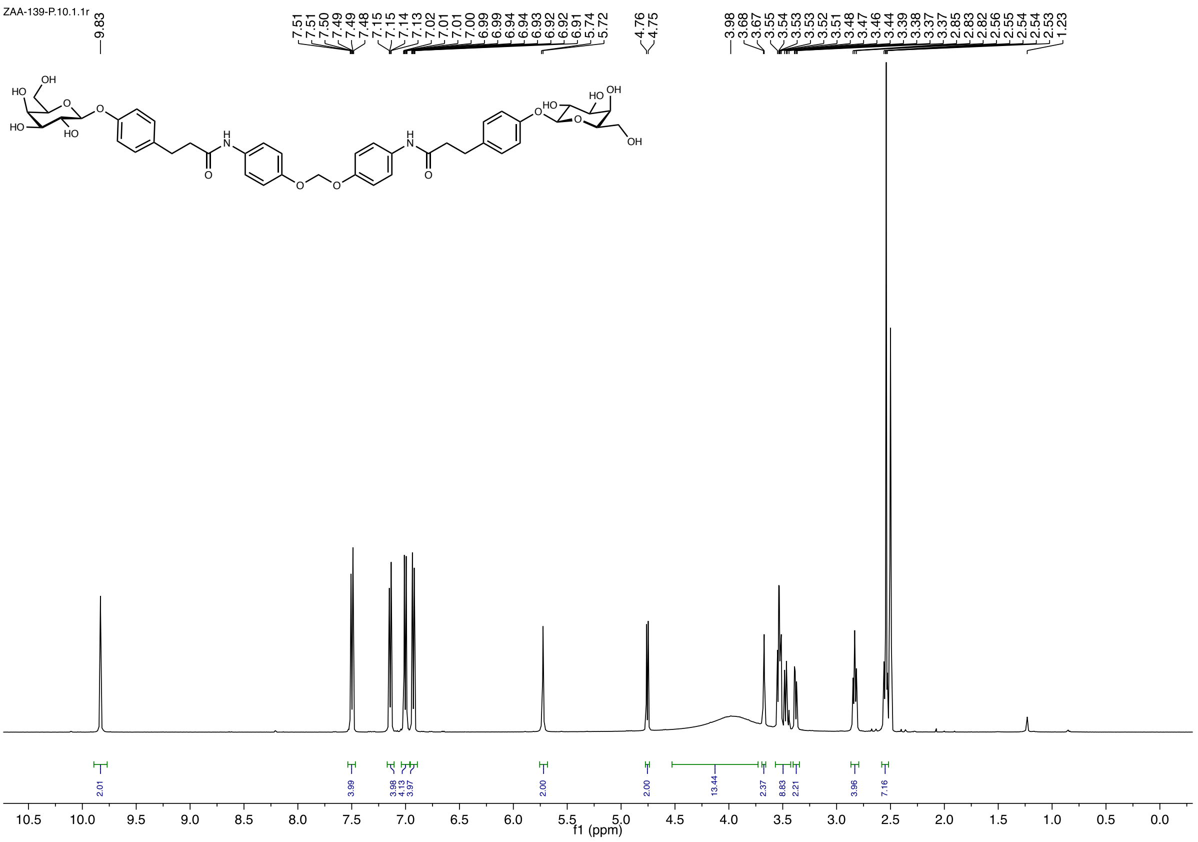

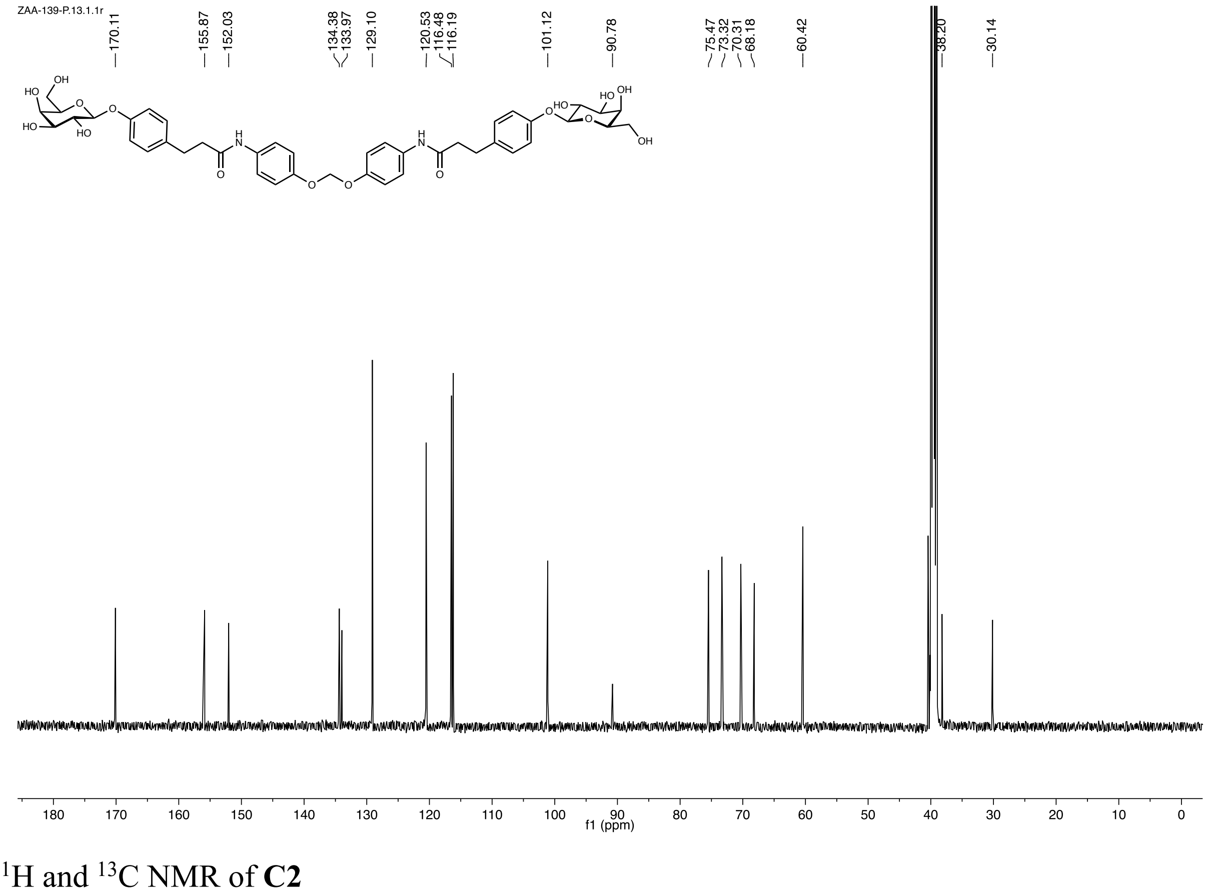

^{1}H and ^{13}C NMR of **C2**

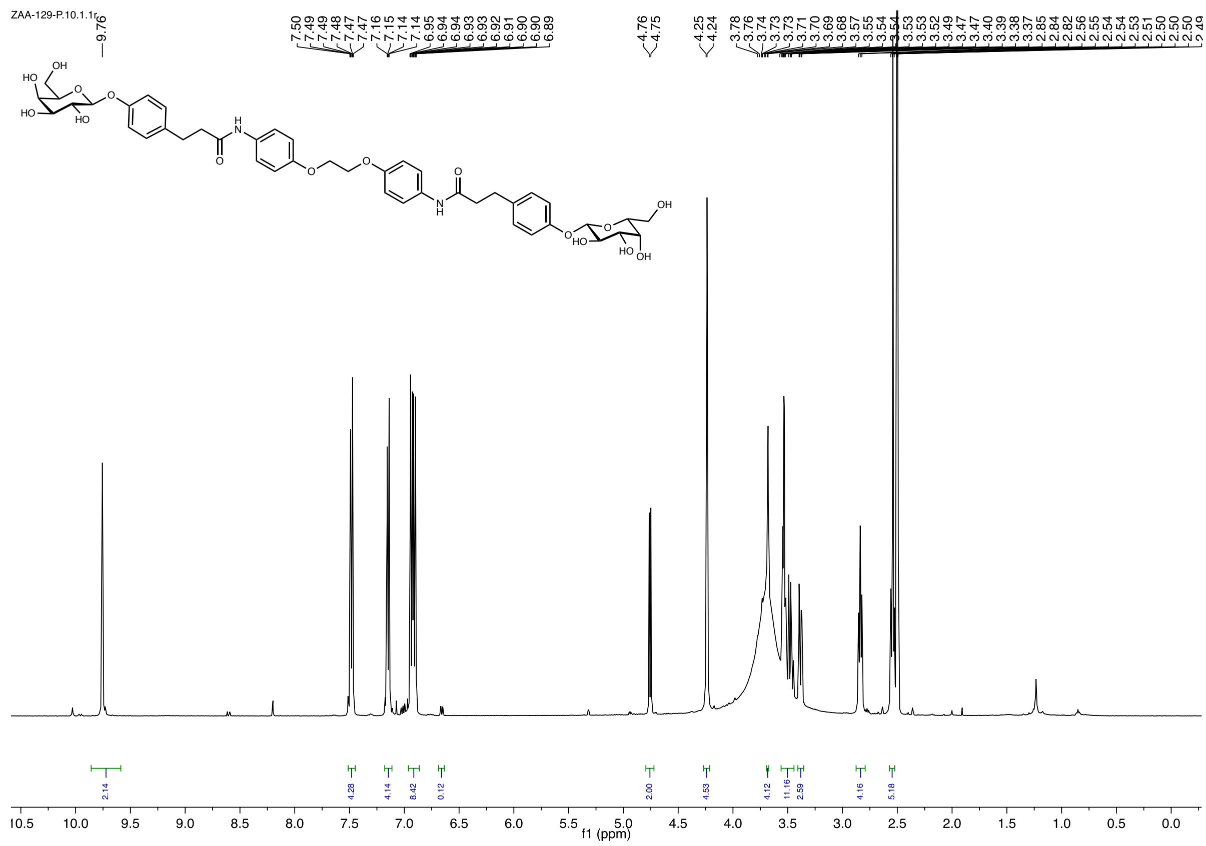

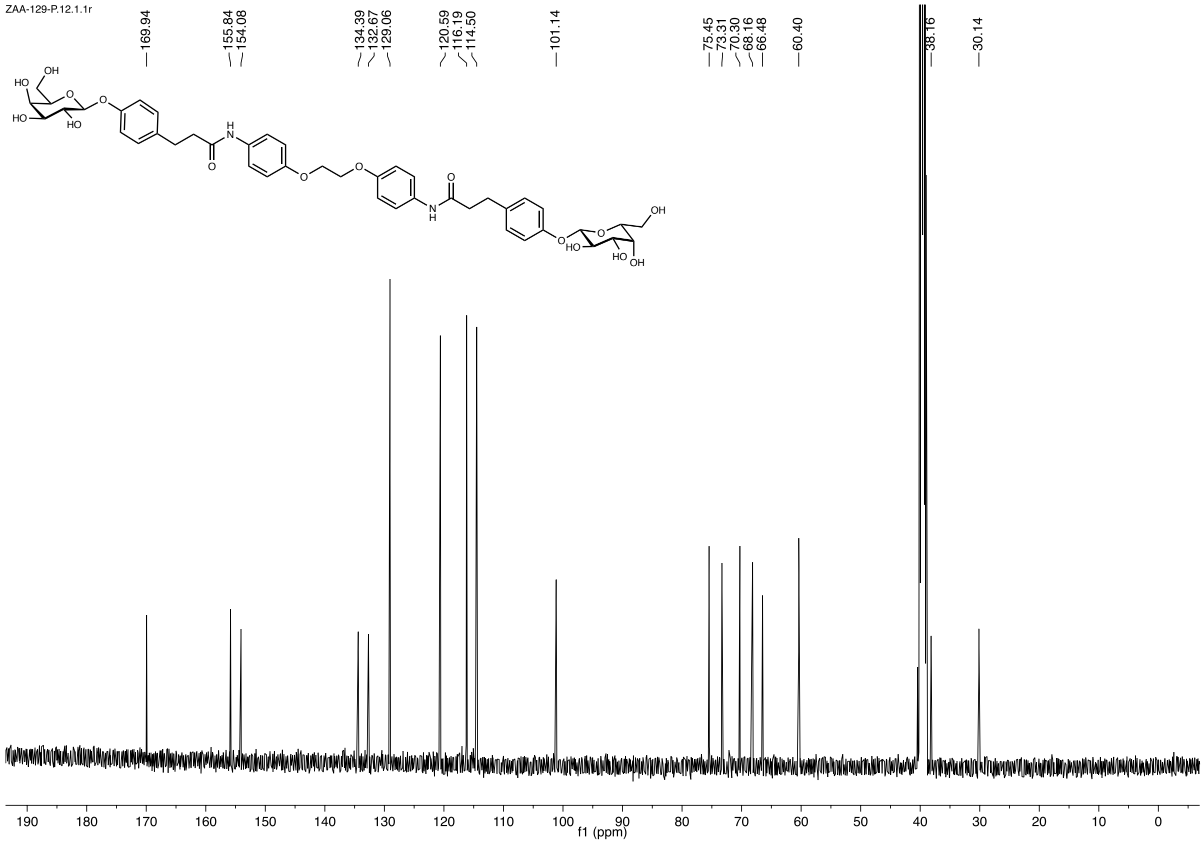

^{1}H and ^{13}C NMR of **D2**

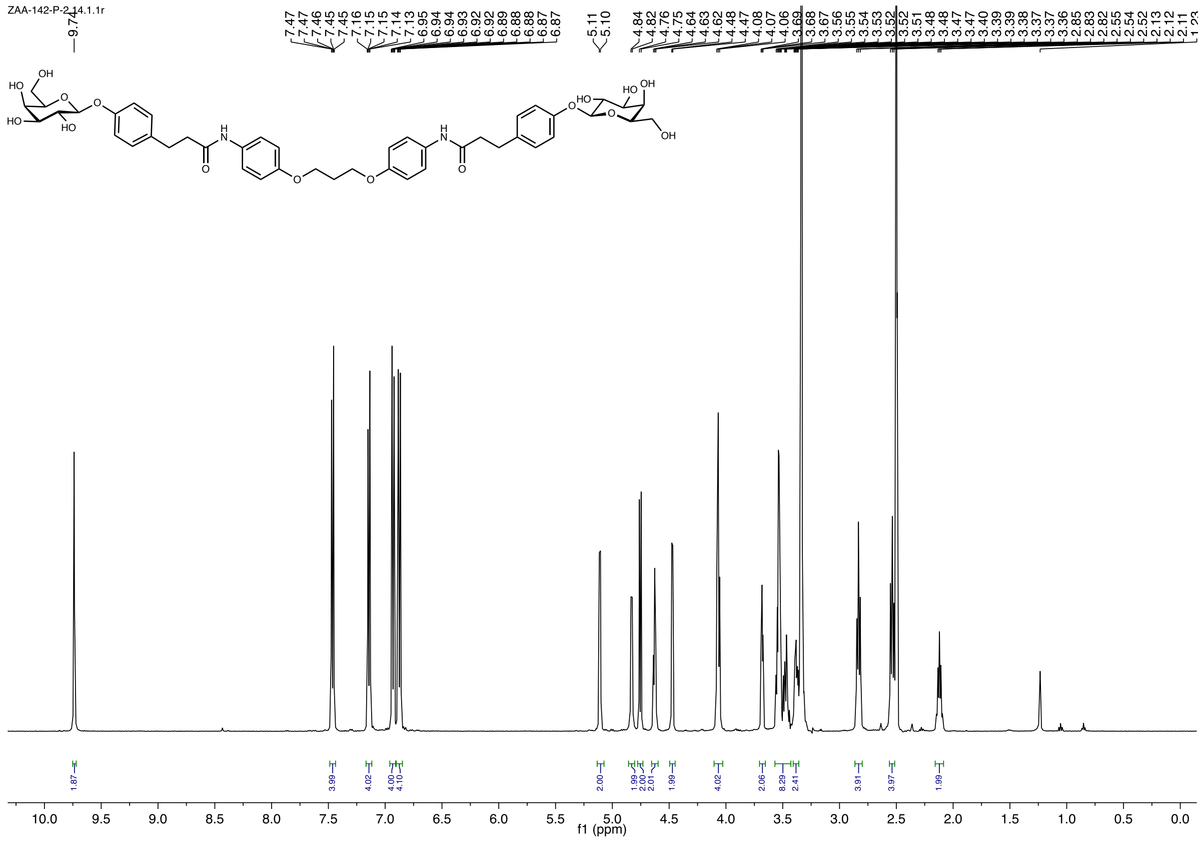

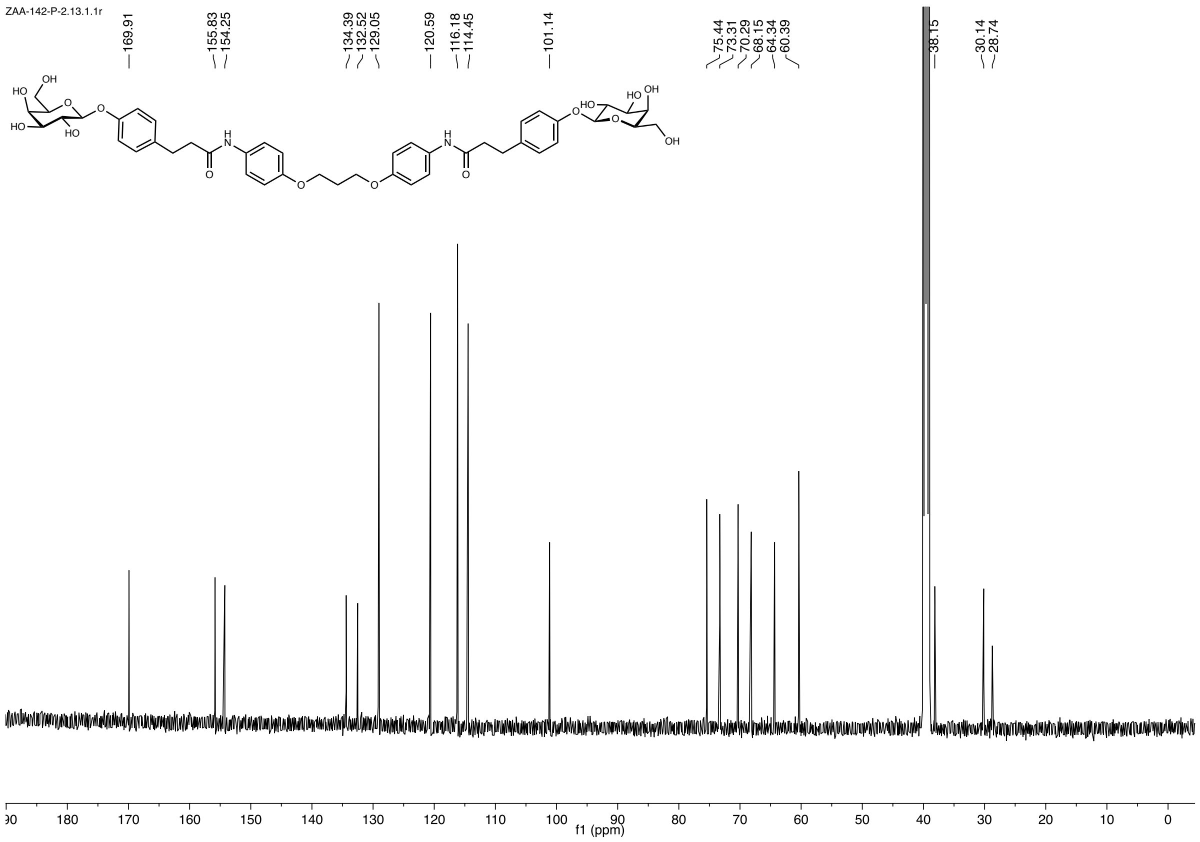

^{1}H and ^{13}C NMR of **E2**

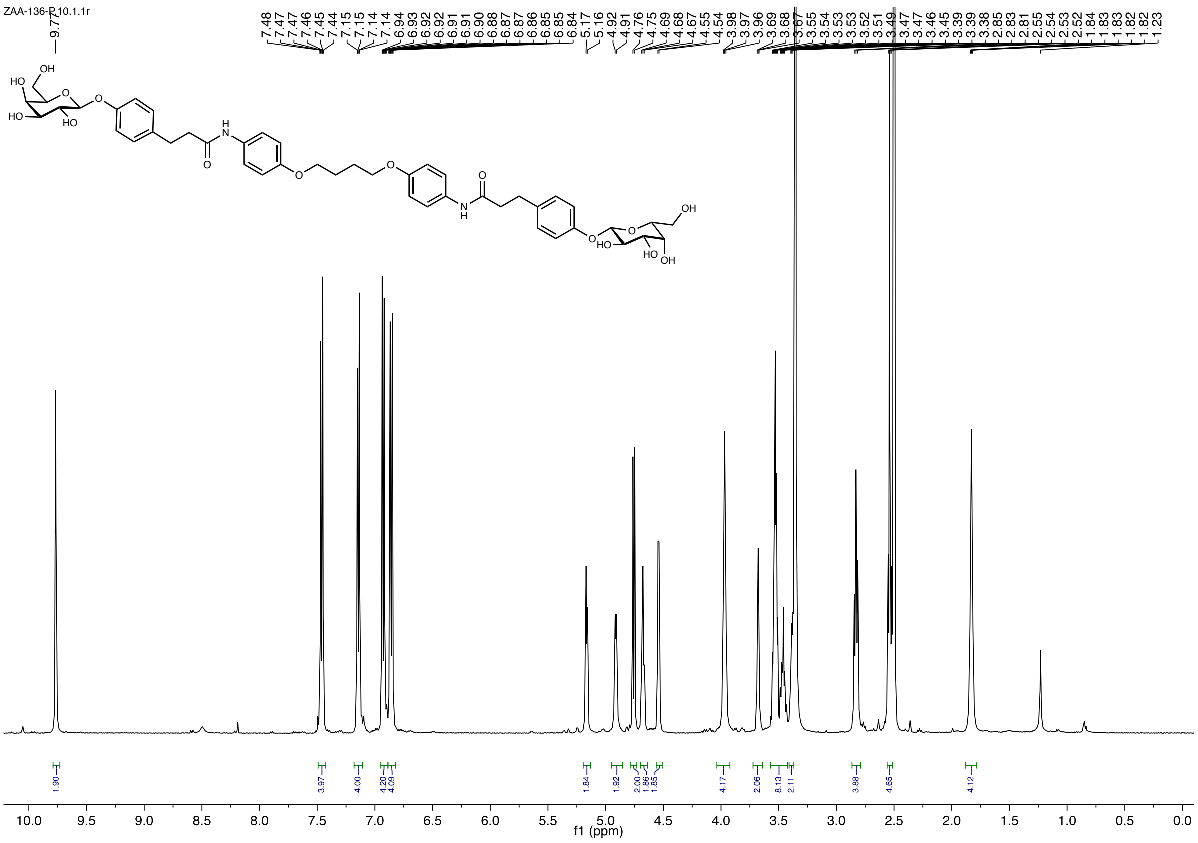

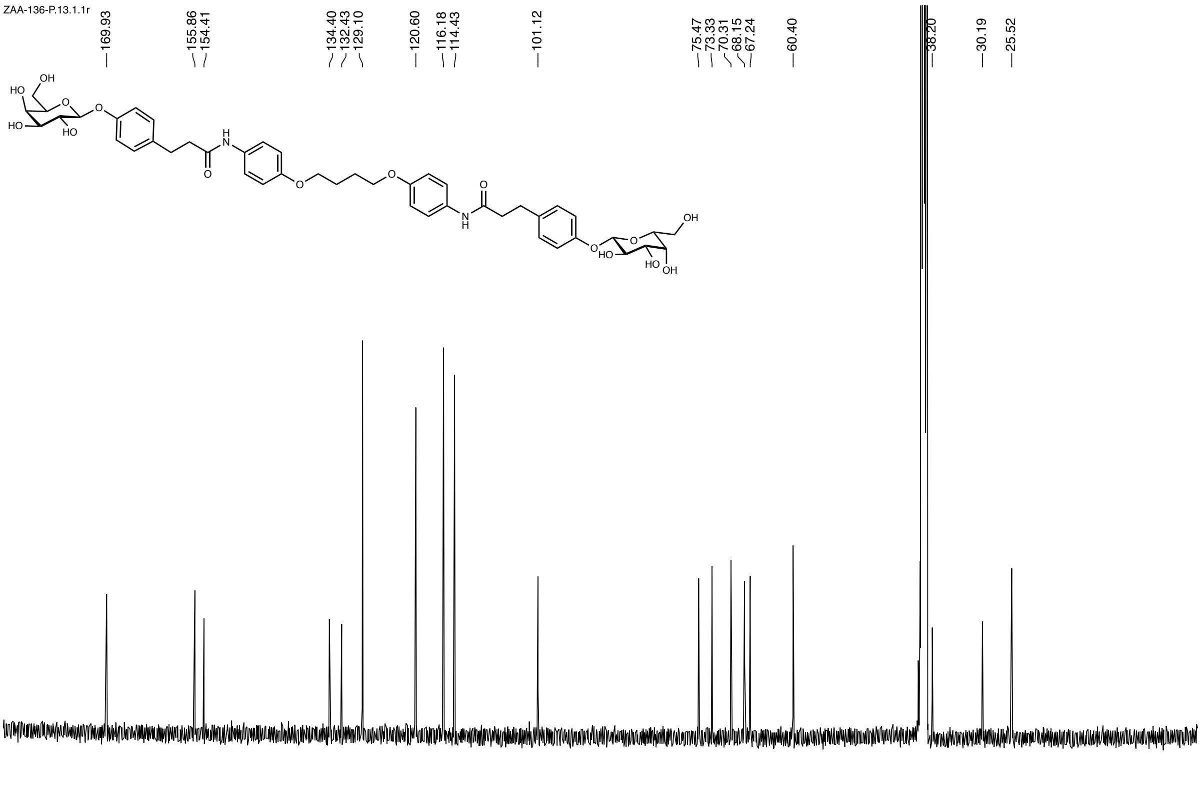

^{1}H and ^{13}C NMR of **F2**

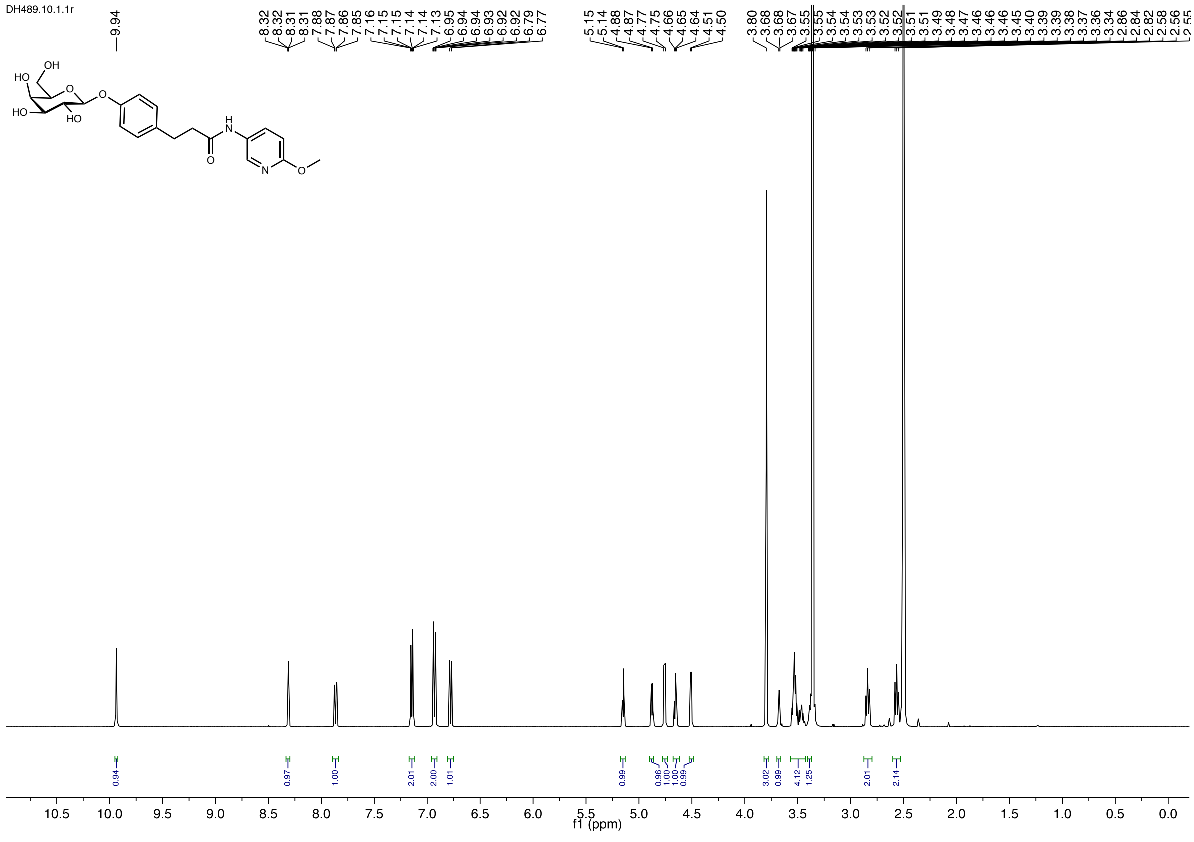

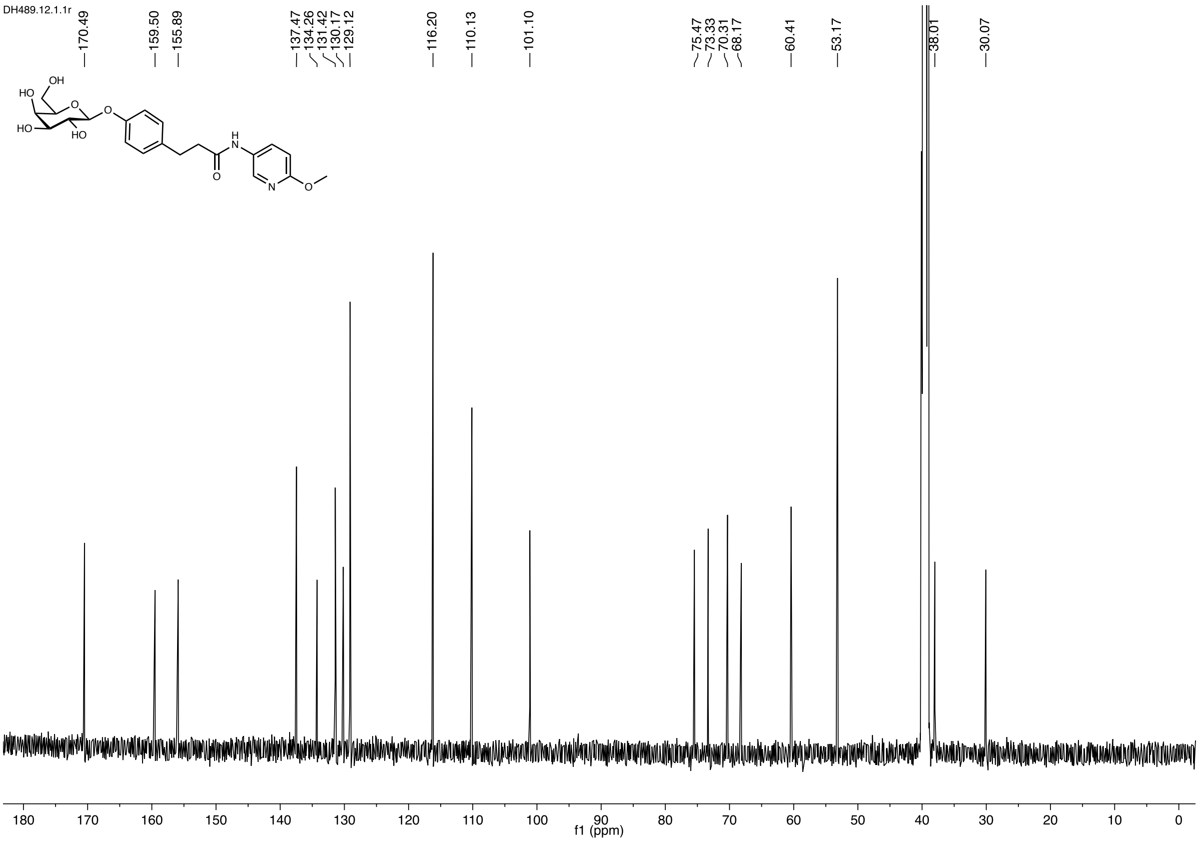

^{1}H and ^{13}C NMR of **G2**

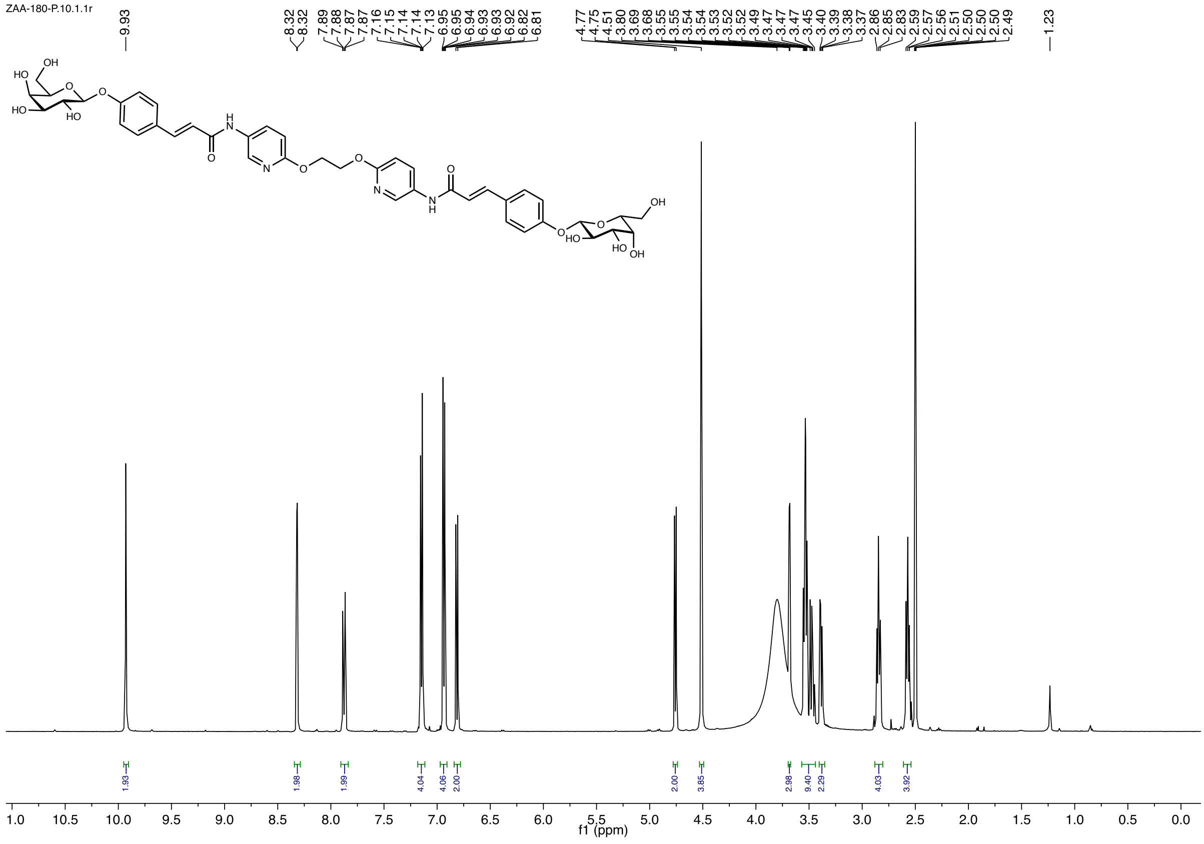

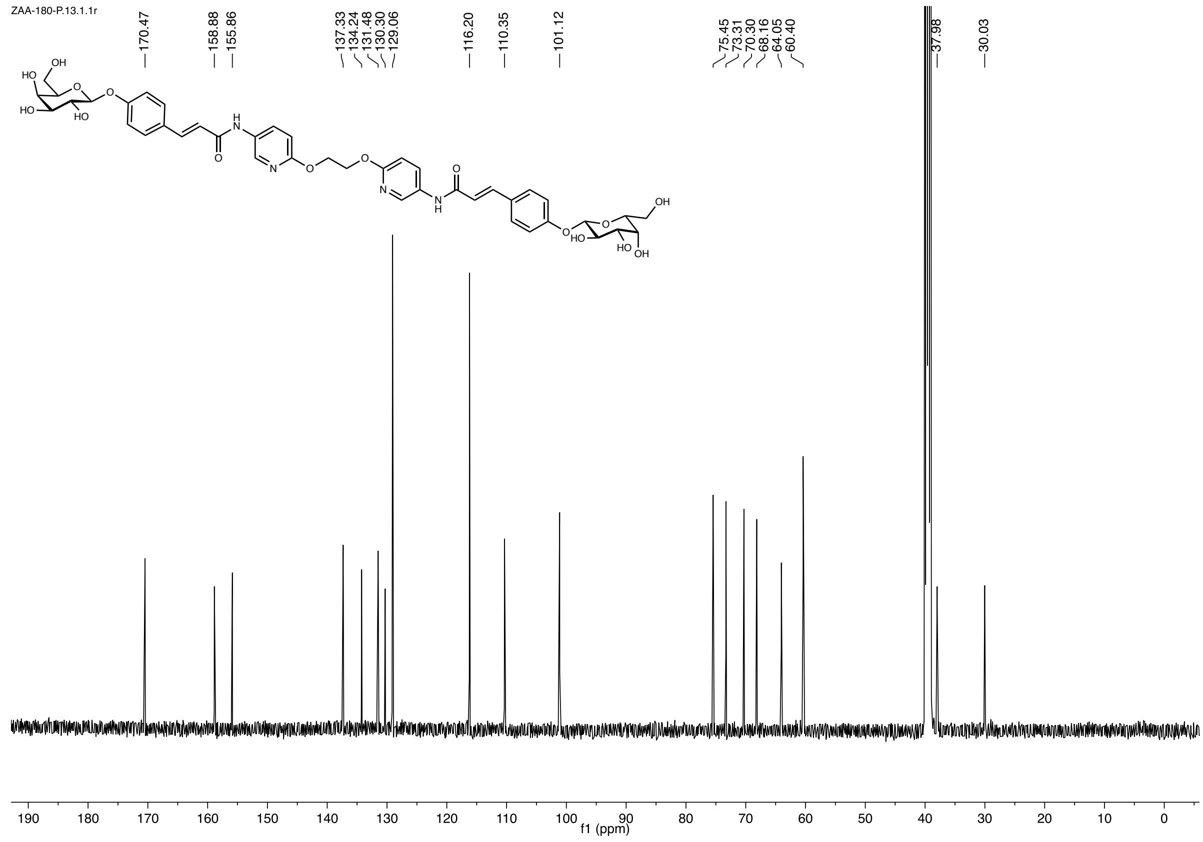

^{1}H and ^{13}C NMR of **H2**

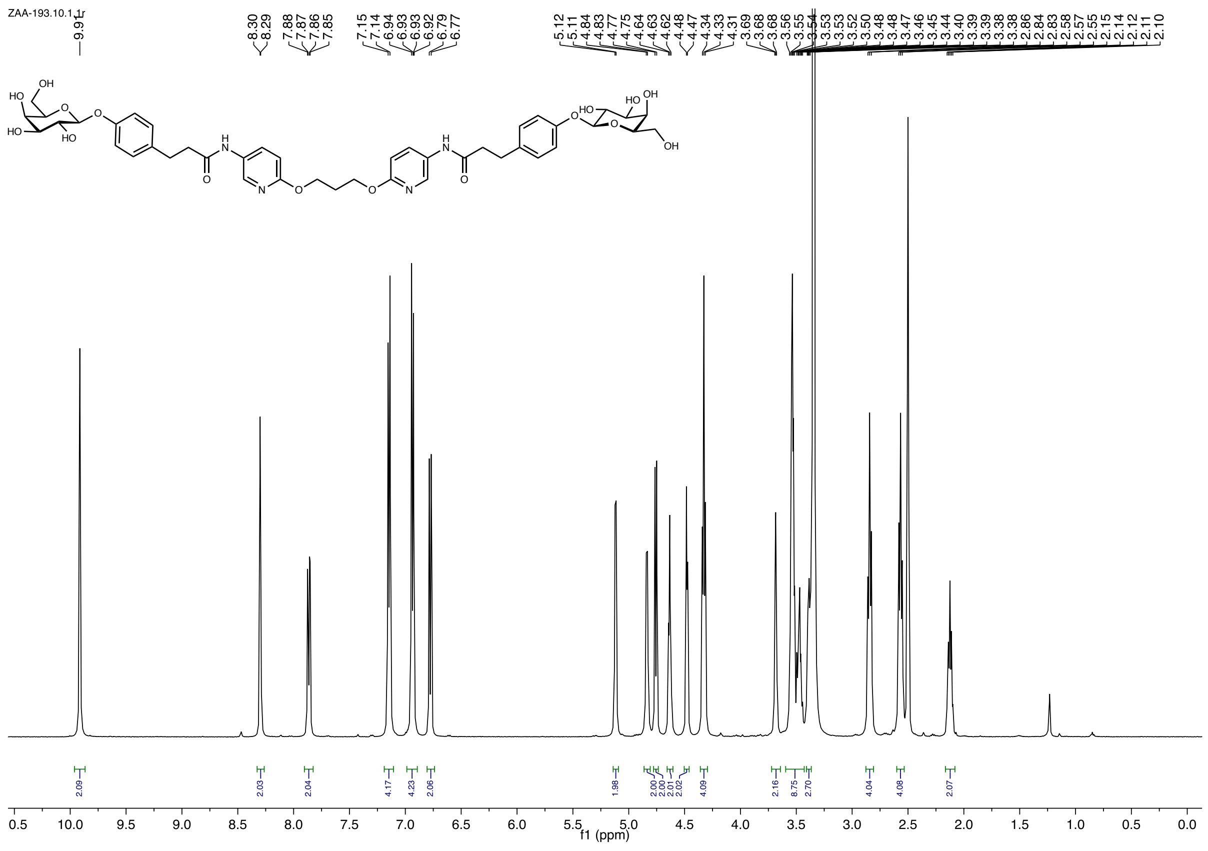

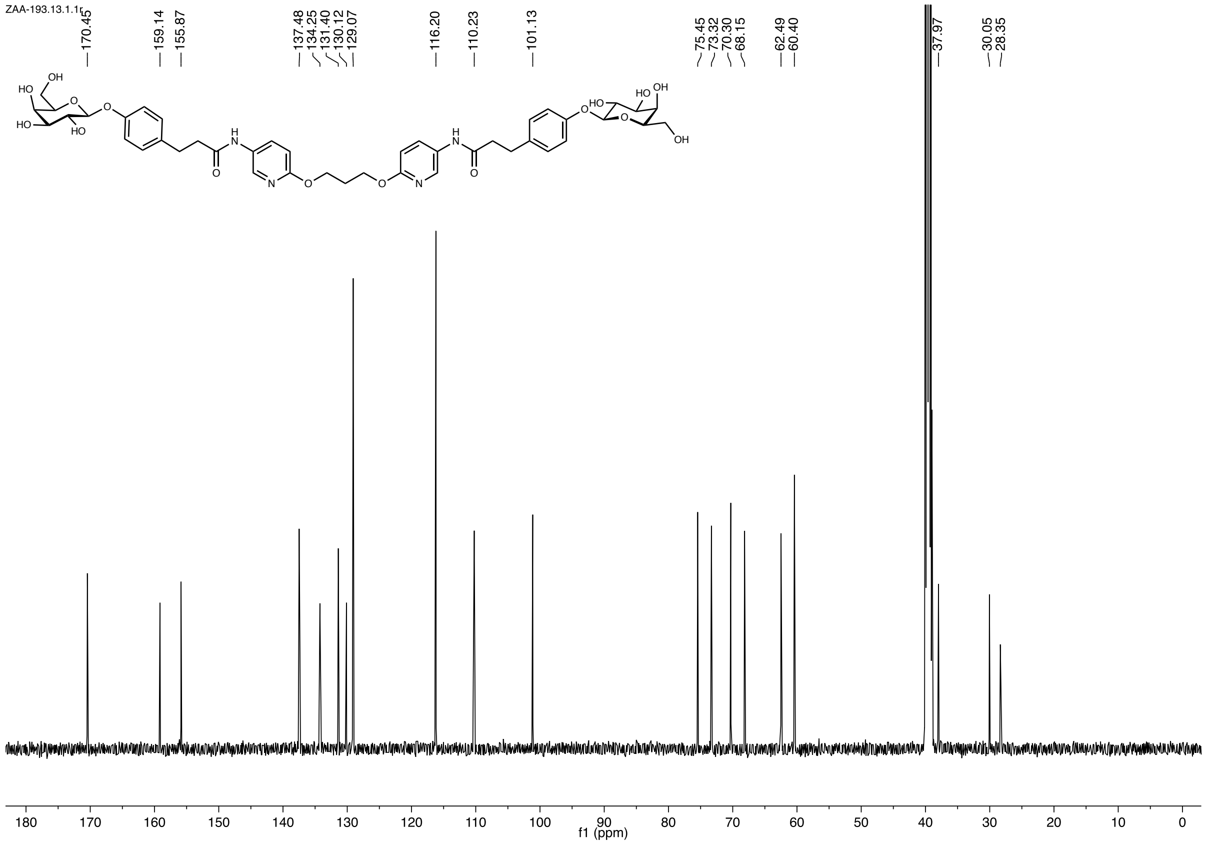

^{1}H and ^{13}C NMR of **I2**

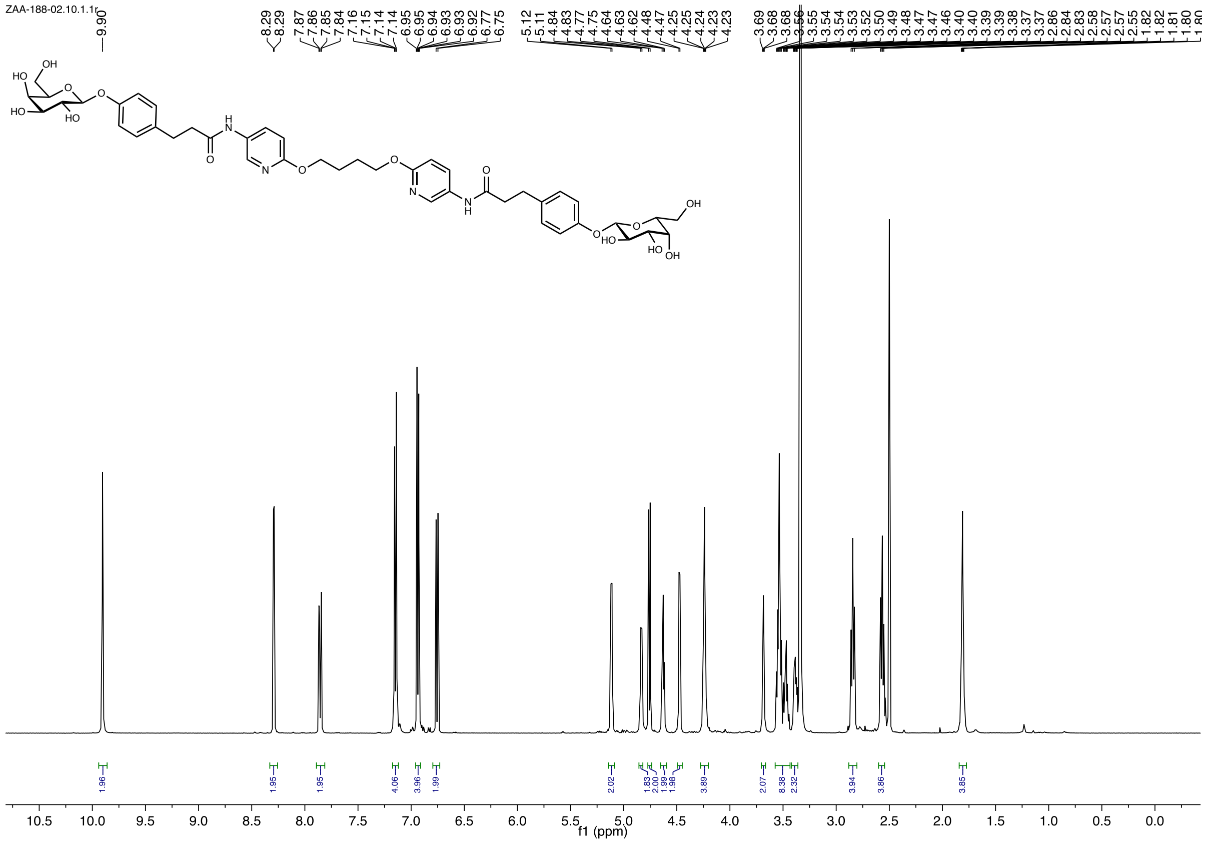

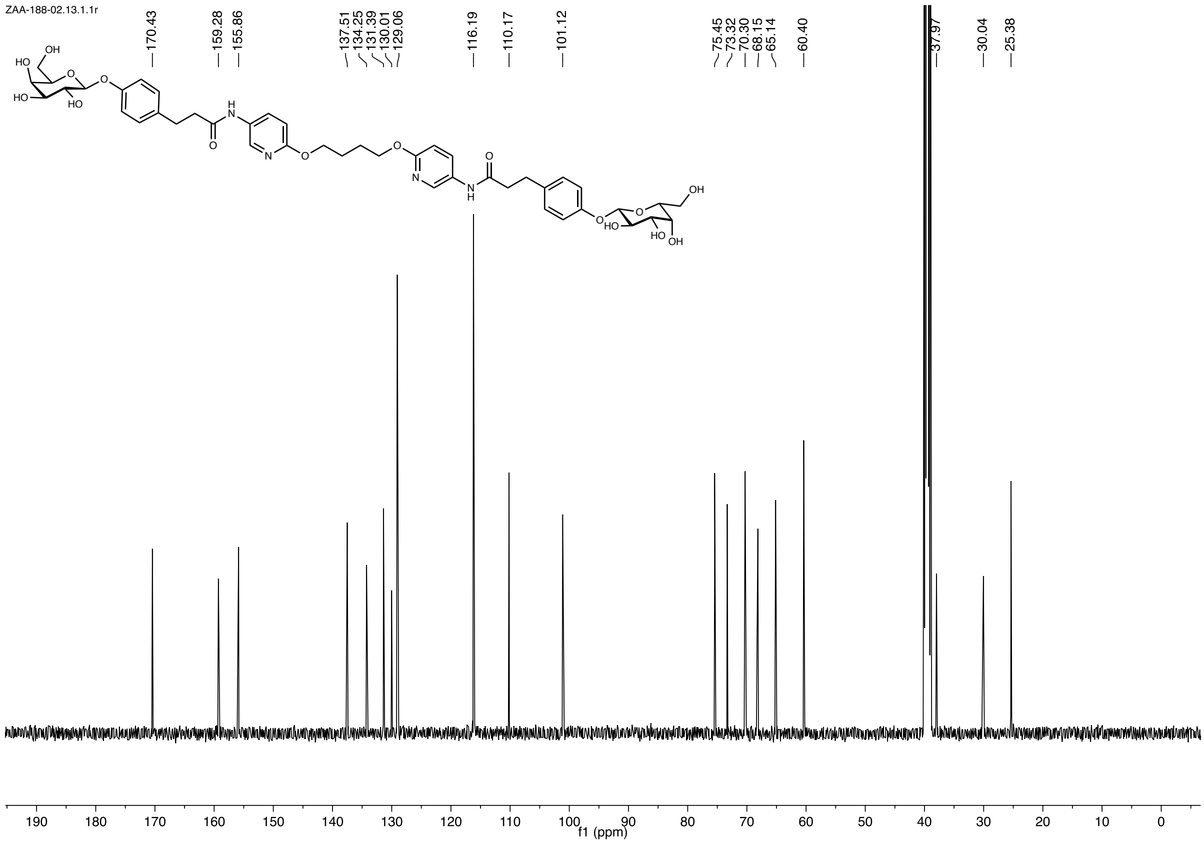

^{1}H and ^{13}C NMR of **J2**

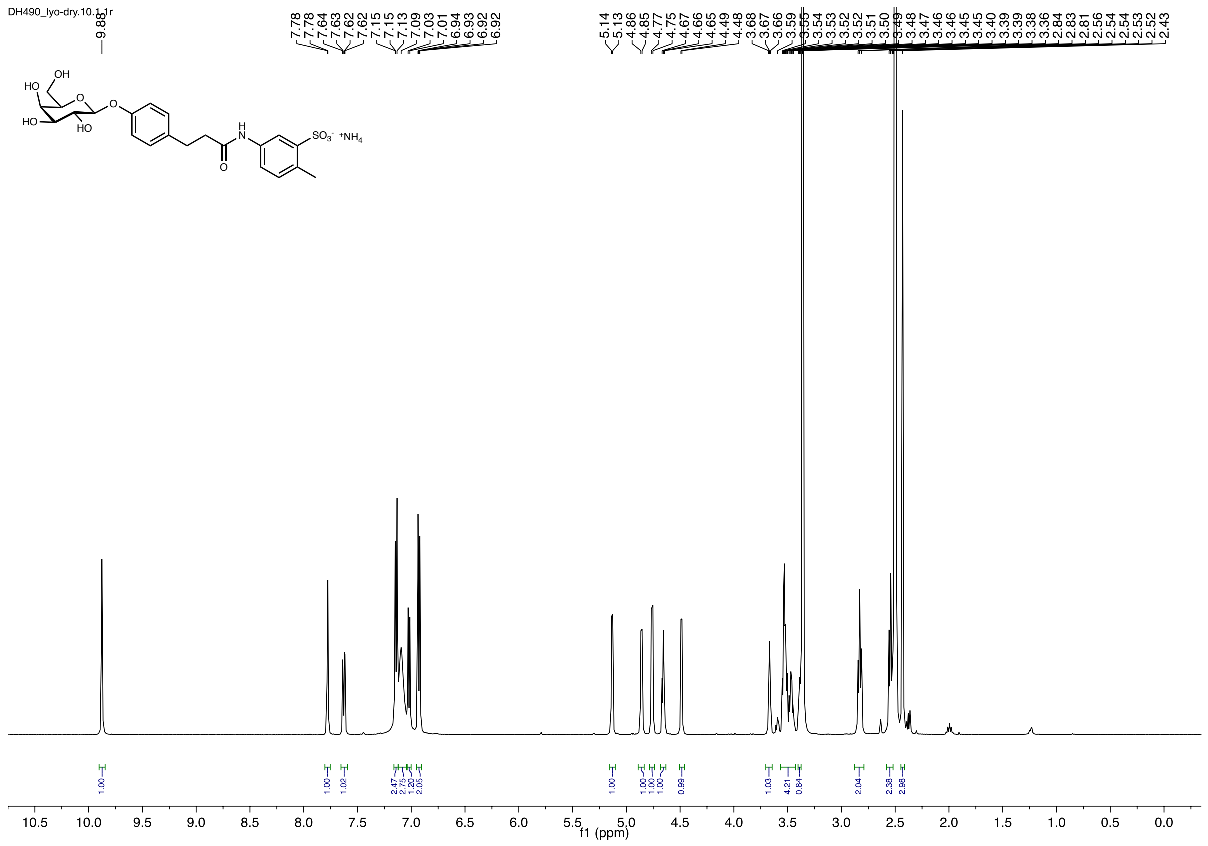

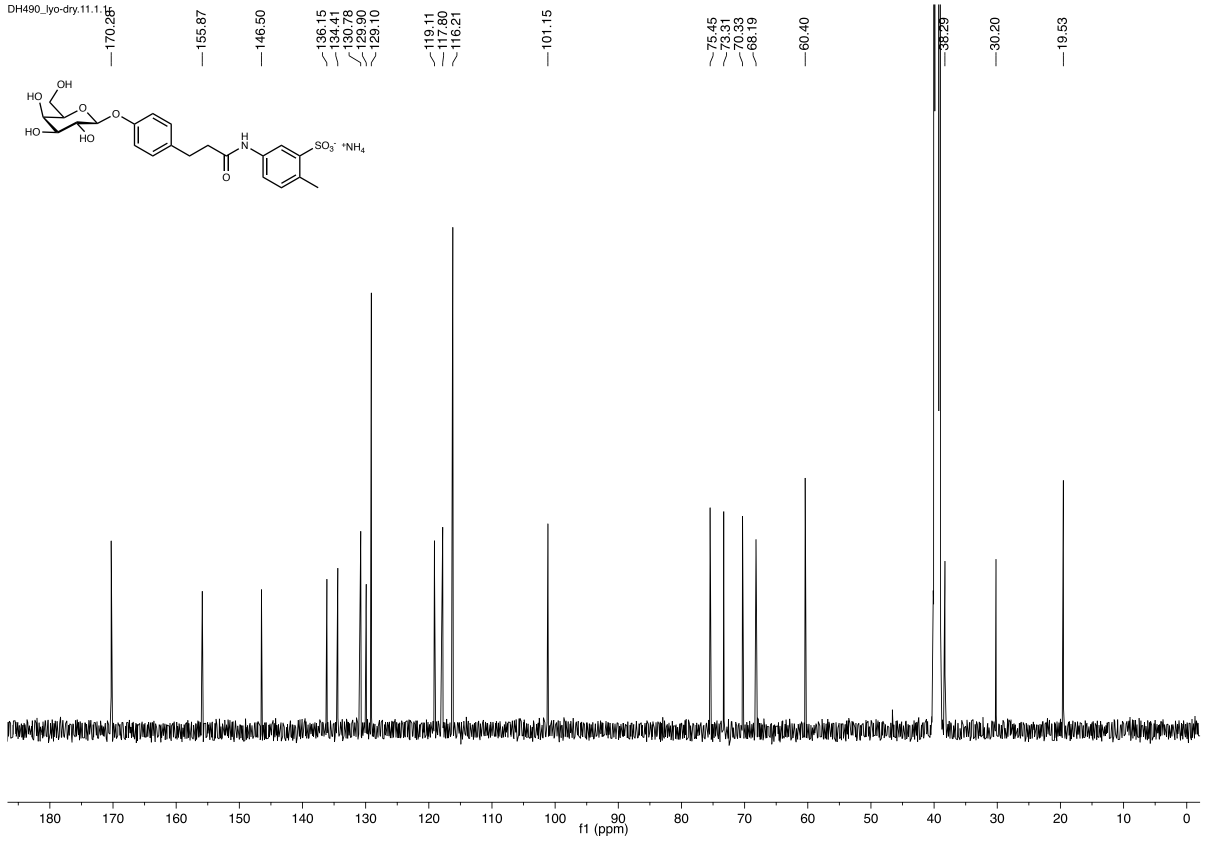

^{1}H and ^{13}C NMR of **K2**

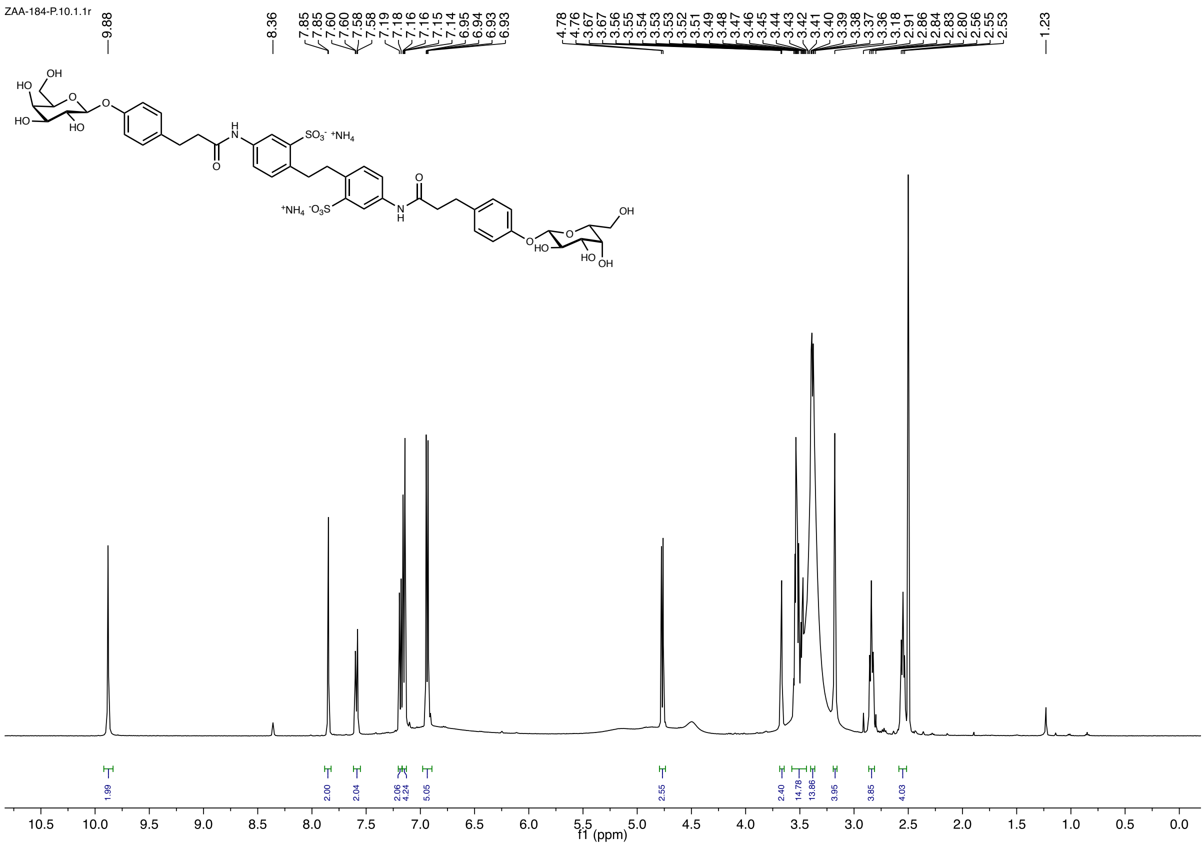

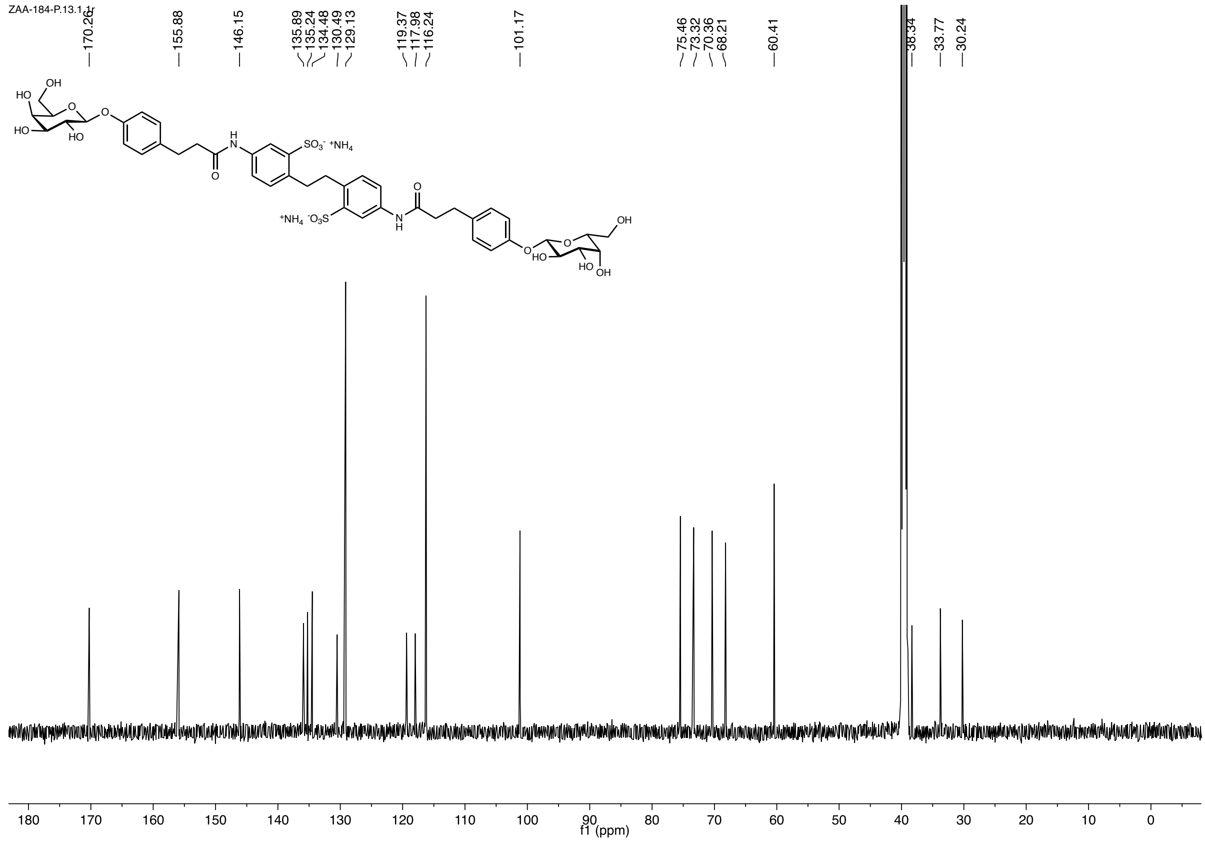

^{1}H and ^{13}C NMR of **L2**

REFERENCES

[1] H. E. Gottlieb, V. Kotlyar, A. Nudelman, *J. Org. Chem.* **1997**, *62*, 7512–7515.

[2] W. Guo, J. Li, N. Fan, W. Wu, P. Zhou, C. Xia, *Synth. Commun.* **2005**, *35*, 145–152.

[3] N. Takada, E. Kato, K. Ueda, S. Yamamura, M. Ueda, *Tetrahedron Lett.* **2002**, *43*, 7655–7658.

[4] S. Akimoto, D. Kato, M. Jikei, M. A. Kakimoto, *J. Photopolym. Sci. Technol.* **1999**, *12*, 245–248.

[5] M. S. Butt, Z. Akhtar, M. Zafar-Uz-Zaman, A. Munir, *Eur. Polym. J.* **2005**, *41*, 1638–1646.

[6] A. Shiotani, M. Kohda, *J. Appl. Polym. Sci.* **1999**, *74*, 2404–2413.

[7] I. N. Bazanova, N. V. Kholodkova, V. P. Gostikin, *Russ. J. Appl. Chem.* **2002**, *75*, 436–440.

[8] E. Zahorska, S. Kuhaudomlarp, S. Minervini, S. Yousaf, M. Lepsik, T. Kinsinger, A. K. H. Hirsch, A. Imberty, A. Titz, *Chem. Commun.* **2020**, *56*, 8822–8825.

[9] I. Joachim, S. Rikker, D. Hauck, D. Ponader, S. Boden, R. Sommer, L. Hartmann, A. Titz, *Org. Biomol. Chem.* **2016**, *14*, 7933–7948.

[10] W. B. Turnbull, A. H. Daranas, *J. Am. Chem. Soc.* **2003**, *125*, 14859–14866.

[11] Y. Zhang, M. Huo, J. Zhou, S. Xie, *Comput. Methods Programs Biomed.* **2010**, *99*, 306–314.

[12] C. Chemani, A. Imberty, S. De Bentzmann, M. Pierre, M. Wimmerová, B. P. Guery, K. Faure, *Infect. Immun.* **2009**, *77*, 2065–2075.

[13] T. Eierhoff, B. Bastian, R. Thuenauer, J. Madl, A. Audfray, S. Aigal, S. Juillot, G. E. Rydell, S. Muller, S. de Bentzmann, et al., *Proc. Natl. Acad. Sci.* **2014**, *111*, 12895–12900.
